# Enumerative Discovery of Noncanonical Polypeptide Secondary Structures

**DOI:** 10.1021/jacs.4c04991

**Published:** 2024-09-04

**Authors:** Adam P. Moyer, Theresa A. Ramelot, Mariano Curti, Margaret A. Eastman, Alex Kang, Asim K. Bera, Roberto Tejero, Patrick J. Salveson, Carles Curutchet, Elisabet Romero, Gaetano T. Montelione, David Baker

**Affiliations:** † Department of Biochemistry and Institute for Protein Design, 552407 University of Washington, Seattle 98195, Washington, United States; ‡ Department of Chemistry and Chemical Biology, Center for Biotechnology and Interdisciplinary Studies, 8024 Rensselaer Polytechnic Institute, Troy 12180, New York, United States; § Institute of Chemical Research of Catalonia (ICIQ-CERCA), Barcelona Institute of Science and Technology, Av. Països Catalans 16, Tarragona 43007, Spain; ∥ Department of Chemistry, 7618 Oklahoma State University, Stillwater, Oklahoma 07478, United States; ⊥ Departament de Farmàcia i Tecnologia Farmacèutica, i Fisicoquímica, Facultat de Farmàcia i Ciències de l’Alimentació, Universitat de Barcelona (UB), Av. Joan XXIII 27-31, Barcelona 08028, Spain; # Institut de Química Teòrica i Computacional (IQTCUB), Universitat de Barcelona (UB), Martí i Franqués 1, Barcelona 08028, Spain

## Abstract

Energetically favorable local interactions can overcome the entropic cost of chain ordering and cause otherwise flexible polymers to adopt regularly repeating backbone conformations. A prominent example is the α helix present in many protein structures, which is stabilized by *i*, *i* + 4 hydrogen bonds between backbone peptide units. With the increased chemical diversity offered by unnatural amino acids and backbones, it has been possible to identify regularly repeating structures not present in proteins, but to date, there has been no systematic approach for identifying new polymers likely to have such structures despite their considerable potential for molecular engineering. Here we describe a systematic approach to search through dipeptide combinations of 130 chemically diverse amino acids to identify those predicted to populate unique low-energy states. We characterize ten newly identified dipeptide repeating structures using circular dichroism spectroscopy and comparison with calculated spectra. NMR and X-ray crystallographic structures of two of these dipeptide-repeat polymers are similar to the computational models. Our approach is readily generalizable to identify low-energy repeating structures for a wide variety of polymers, and our ordered dipeptide repeats provide new building blocks for molecular engineering.

## Graphical abstract

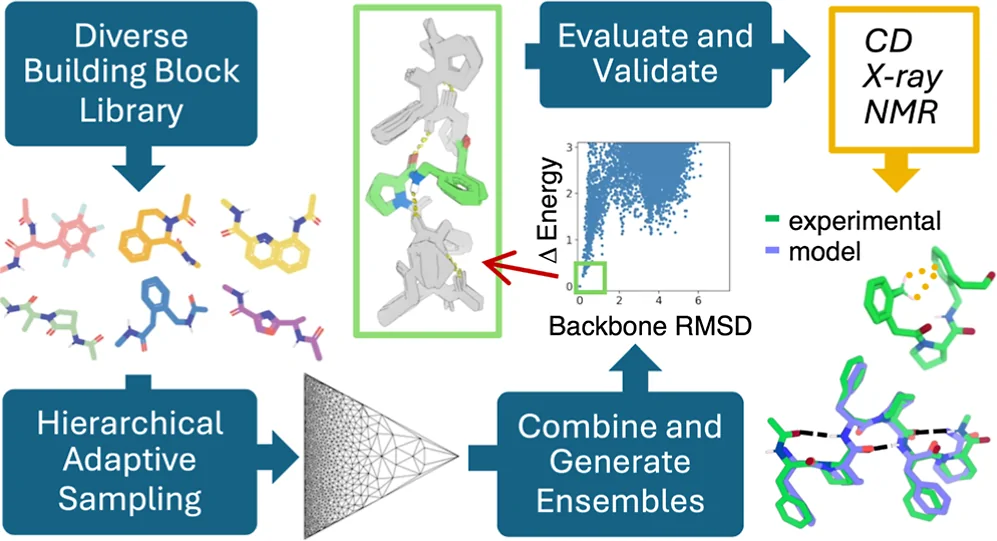

## Introduction

Linus Pauling discovered the α helix by building models of polypeptide chains that maximize hydrogen bonds between backbone NH and CO groups without introducing backbone strain.^1^ Synthetic and computational peptide chemists have discovered hundreds of additional repeating secondary structures that are stabilized by noncanonical amino acids (for selected reviews, see Refs 2–11 ); several of these novel secondary structures have higher thermodynamic stability compared to the native helices, and higher biological stability as they are not susceptible to natural protein degradation processes (for examples see Refs 4,9,12,13 ). Such abiotic secondary structures provide new avenues for macromolecular design, as illustrated by designed β peptide helical bundles,^9,12,14^ and a comprehensive set of these structures would have considerable utility. In principle, it should be possible to identify many more regular repeating arrangements of nonprotein polymers that have low energy compared to alternative conformations, and hence be highly populated at equilibrium, by direct calculation. Perhaps because of the magnitude of the chemical and conformational spaces that must be covered, few systematic computational searches for abiotic secondary structures have been described. Conformational searching has been used to identify relatively low-energy helices with extended hydrogen-bonded backbones including α/β, α/γ, β/γ, and α/δ dipeptide-repeating polymers.^8,15–18^ However, side-chain−side-chain and side-chain−backbone interactions (closed cycles, tertiary amides, hydrophobic contacts, and hydrogen bonds), which can play critical roles in stabilizing peptide conformations (see examples in Refs 8,18–20 ), have not been considered in large scale enumeration studies due to the combinatoric explosion of sequences and side-chain conformations.

Computational approaches originally developed for protein design have in recent years been applied to polypeptide macrocycles containing unnatural amino acids with considerable success, enabling systematic identification of 7–12 residue cyclic peptides that adopt single stable and membrane permeable structures.^21,22^ We reasoned that large-scale computational enumeration could similarly be used to systematically identify abiotic secondary structures stabilized by unnatural amino acids. Such an approach should be able to screen far more residue compositions than previous low-throughput efforts, and include costly or synthetically challenging residues that would typically be excluded from a large-scale experimental effort. We set out to develop an unbiased energy-based method to identify repeating dipeptide sequences likely to adopt low-energy states with structurally repetitive backbone conformations, which has not been done at this scale previously.^6,15–18,23,24^

## Results and Discussion

The regular repeating structures in proteins, the α helix and β sheet, have a single residue repeat unit; i.e., all residues in an α helix, for example, have backbone ϕ and ψ values in the same range. Because of the greater chemical diversity of unnatural amino acids, we hypothesized that repeating structures with dipeptide structural repeat units could also, in some cases, be stable ground states. Therefore, we sought to develop a computational method that, given a set of unnatural amino acids AA_*i*_, could determine which repeating dipeptide combinations (AA_*i*_–AA_*j*_)_*n*_ have regular repeating structures as conformational low-energy states. We reasoned that four repeat units should be sufficient to capture the majority of sequential and longer-range secondary structure interactions, and for computational tractability, we decided to focus on identifying octapeptides with structurally repeating ground states [labeled from here on as (AB)_4_].

The considerable chemical diversity of unnatural amino acids complicates use of traditional classical force fields for accurate energy evaluation, as parameters must be obtained for all functional groups. To avoid the need for force field parametrization and to achieve higher accuracy, we instead chose to use the AIMNet neural network for energy evaluations,^25^ which rapidly and accurately estimates the energies of small molecules otherwise obtained with costly density functional theory (DFT) calculations.

Given the above choices to focus on (AB)_4_ peptides and use AIMNet for energy evaluation, in principle, one could, for each (AB)_4_ peptide, systematically sample conformational space and identify those peptides for which regular repeating structures had the lowest energies. However, such a direct approach is not currently feasible. There are hundreds of unnatural amino acids of interest, with 2 to 6 internal dihedral degrees of freedom and three internal degrees of freedom on average. Sampling the dihedrals on a relatively coarse 10-degree grid gives 36 possible values for each degree of freedom, or around 36^3^ conformers to evaluate per monomer, and 36^3^ × 36^3^ per dipeptide (since the two monomers in the dipeptide repeat (DPR) unit can have different conformations). In total, this approach would require about 45 trillion energy evaluations for the ∼20,000 dipeptide building blocks we explored in this study (20,475 × 36^3^ × 36^3^ = 44.6 × 10^12^); and given that a single AIMNet energy calculation on an octapeptide takes 5 s, performing the direct calculation would require ∼7.1 million CPU years (See Table S1 for a complete residue list and calculation of unique dipeptide combinations).

To make the search tractable, we developed a hierarchical approach based on the assumption that for a DPR to have a low-energy ground state, both monomer conformations must have relatively low energy. This is plausible because the interactions between adjacent amino acids are unlikely to be significantly stronger than those within amino acids and, hence, are unlikely to be able to compensate for locally strained monomeric conformers. With this assumption, we can divide the search into three steps: first, calculate the potential energy surface for each of the residues as monomeric units; second, use these landscapes to guide sampling of low energy dipeptide conformations; and third, for each of the low energy dipeptide conformations, generate short helical structures consisting of four repeats, and evaluate whether they are lower in energy than other sampled conformations. Related buildup approaches have been used to compute low-energy conformations of natural polypeptides.^26^ We describe these steps in turn in the following paragraphs and schematically in Figure 1.

**Figure 1:**
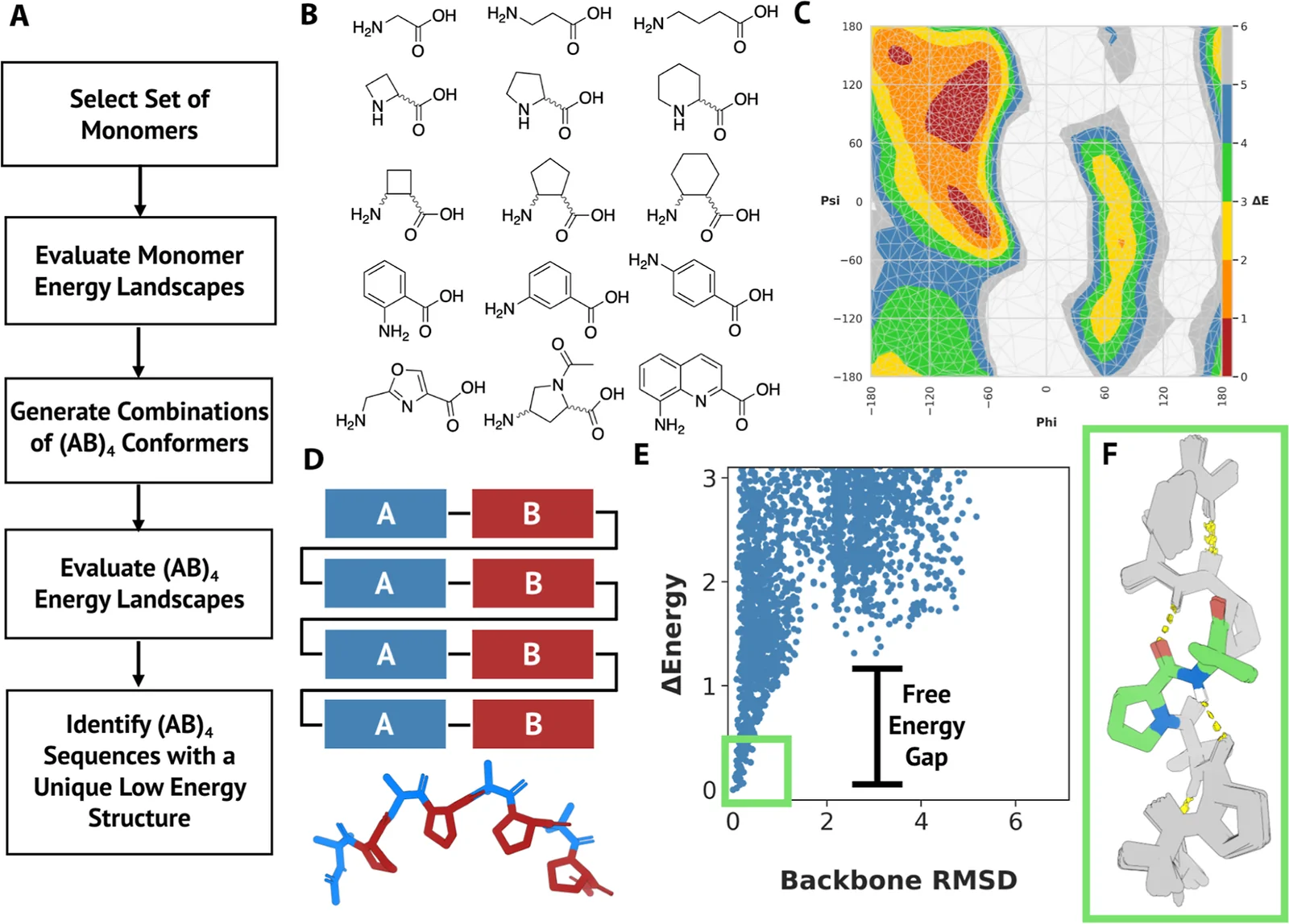
Computational pipeline for novel secondary structure discovery. (A) Process flow diagram for computational discovery of (AB)_*n*_ secondary structures from noncanonical amino acids. (B) Representative examples of noncanonical amino acids considered in this study. The full list of residues that were considered in this study is in Table S1. (C) Example potential energy surface of the monomeric l-Alanine residue sampled with the VABLAS protocol. (D) Schematic representation of (AB)_*n*_ repeating peptide and the chemical structure of a representative example. (E) Example of a computational conformational ensemble shown as the delta energy to the lowest energy state versus the RMSD with respect to the backbone atoms of the lowest energy state. The free energy gap between the lowest energy state and the next lowest energy state is highlighted with a black bar, and the 100 lowest energy conformers from the ensemble are enclosed in a green box and shown in (F) aligned by their backbone atoms. The repeat unit is highlighted in color.

### Monomer Energy Landscape Calculation

Carrying out even the first step, the full determination of the energy landscape of monomeric building blocks, is challenging when there are large numbers of blocks since noncanonical residues can have many continuous degrees of freedom when considering the torsions of both the backbone and the side chain. There are also discrete degrees of freedom, such as ring flips, for which the continuous degrees of freedom must be independently sampled. We developed an efficient hierarchical approach to determining the potential energy surfaces of these multidimensional systems. We evaluated the energy of the noncanonical residues with AIMNet­(SMD)-D4, a neural network trained on millions of DFT calculations and fine-tuned with the SMD solvation correction,^27^ including the D4 dispersion in the computed energies. To sample low-energy conformers, we developed a multiresolution adaptive sampling technique, which we call Volume and Boltzmann Loss Adaptive Sampling (VABLAS). VABLAS is an iterative process that utilizes a Delaunay triangulation where each vertex is a previously sampled point. At each optimization stage, simplices with low average energy and large volume are selected and subdivided into new smaller simplices; this process is iterated until convergence. VABLAS enables identification of low-energy regions of the potential energy surface of the noncanonical residues without a priori knowledge of the location of the minima. Details of VABLAS are provided in the Supporting Information (See Monomer sampling protocol, Figures S1–S3, and Codes S1 and S2).

We used this computational pipeline to generate energy landscapes for 130 noncanonical amino acids, most of which are readily available from commercial vendors. These noncanonical amino acids have 25 different backbone chemical structures, for example, β, γ, and δ amino acids, in addition to the standard protein peptide backbone. The additional chemical variation in the set consists of cyclic groups and other side chains likely to bias backbone geometry, such as ortho, meta, and para substitutions of amino-benzoic acid and cyclization patterns, as well as alternate chirality at stereocenters. Of the 130 residues, 92 have a chiral center, and 40 residues are proline-like with a secondary amide at the peptide ligation site (Figure S4).

### Repeat Conformer Generation

We focused our efforts on finding repeating low-energy secondary structures built from a dipeptide-like repeat unit where the two monomers can have distinct conformations (unlike the α helix, for example, where all residues have roughly the same backbone conformation). Taking into account the chirality of the building blocks, the 130 monomers yielded 20,475 combinations of unique dipeptide building blocks. Conformational ensembles for each of these building blocks were generated with a bias from the monomer energy landscapes. Monomer conformations were sampled based on their Boltzmann probability with a kT of 1.2 kcal/mol, which allowed for inclusion of higher energy states while still focusing the sampling toward lower energy monomer conformations. Dipeptide building block structures were then propagated to generate an octapeptide with helical symmetry consisting of four identical dipeptide units. Conformations with internal clashes were discarded, and the remaining octapeptides were evaluated using AIMNet­(SMD)-D4. A second stage of sampling was carried out around the low-energy polymer conformations biased by Boltzmann probability; a lower kT of 0.6 kcal/mol/residue was used. During this second stage, the ten closest (in dihedral angles) monomeric conformers were selected for each of the two original constituent monomers, and all 100 combinations of these conformers were similarly evaluated in polymeric form. This two-stage process enabled both (1) wide sampling to discover rare low-energy states and (2) narrow sampling to fully explore the conformations around the rare low-energy states. For each low energy state, we evaluated the difference in energy with the lowest energy conformation sampled for the same chemical structure greater than 2.0 Å RMSD away and selected those for which this difference was greater than 0.66 kcal/mol/residue (see Polymer Sampling Protocol and Code S3). The identification of previously characterized DPRs (Figure S5) suggested that this selection threshold was reasonable. Ten chemically and structurally diverse designs that were predicted to be global energy minima, readily synthesizable, and not previously described in literature were selected for experimental synthesis and characterization.

### Conformations of Computationally Predicted Secondary Structures

Helical parameters, backbone dihedral angles, and other structural features of the ten selected novel secondary structures are reported in Tables 1 and S2 and S3, alongside canonical 3_10_, α, and π helices. Six selected designs are shown as schematic representations along with the corresponding energy diagrams for their computed conformational ensembles in Figure 2. These new dipeptide-repeat secondary structures (referred to here as DPRs) have diverse sizes and shapes: some are tall and narrow with a large pitch, while others are short and wide with a radius twice the size of an α helix. They incorporate amino acids with different backbone lengths, from α to δ amino acids, allowing for exploration of new conformations. A common feature is repeating hydrogen-bonded interactions, ranging from short to long-range (2 to 6 residues), including ‘mixed’ helices with hydrogen bonds running in opposite directions along the helical axis. Noncanonical side chains with hydrogen bonding functional groups form in some cases complex networks of backbone–side-chain–backbone hydrogen bonds; the diversity in hydrogen bonding patterns among the designs is highlighted in Figures 2 and S6A,B. Many designs feature mixed chirality backbone and side-chain conformations and cis-peptide bond geometries. Conformationally constrained residues, including proline-like chemistries, aromatic rings, and other closed cycles, favor distinct global minima by limiting the accessible conformations, and internal hydrophobic contacts, involving both backbone and side chains, also stabilize the designed states.

**Table 1:** Helical Parameters for Ideal and Design Models

helices	3_10_ helix	α helix	π helix	DPR1	DPR2	DPR3	DPR4
sequence (AB)_*n*_				TYR PIP	AMPA^a^ PRO	ALA NIP	ACHC^a^ NIP
chirality A	L	L	L	*S*	achiral	*S*	*S*, *R*
chirality B				*R*	*S*	*S*	*S*
amino acid types A,B	α	α	α	α, α	δ, α	α, β	β, β
helix handedness	right	right	right	right	right	right	right
# residues per turn (RPT)	3.0	3.6	4.2	3.7	2.7	4.6	3.2
radius	1.9 Å	2.3 Å	2.8 Å	∼2 Å	∼2 Å	∼3 Å	∼2 Å
pitch (rise per turn)	5.8 Å	5.4 Å	4.1 Å	9.0 Å	6.3 Å	5.5 Å	6.6 Å
rotation angle per repeat (twist)^b^	120°	100°	85°	195°	270°	160°	225°
hydrogen bond pattern (CO, NH)	*i*, *i* + 3	*i*, *i* + 4	*i*, *i* + 5	*i*, *i* + 3	*i*, *i* + 3	*i*, *i* + 5	*i*, *i* + 3
# atoms in ring formed by hbond	10	13	16	10	13	18	12
peptide bond conformation *w* A–B	trans	trans	trans	trans	trans	cis	cis

a AMPA is called AMACBEN2 and ACHC is ACH12C in the SMILES residue list (Table S1).

b The rotation angle per repeat (twist) is provided for each repeating unit. For 3_10_, α, and π helices, each with a single residue repeating unit, twist = 360°/RPT. For DPR helices with a dipeptide repeating unit, twist = 2 × 360°/RPT, denoting the rotation angle per repeating dipeptide unit. All twist angles are rounded to the nearest 5°.

**Figure 2:**
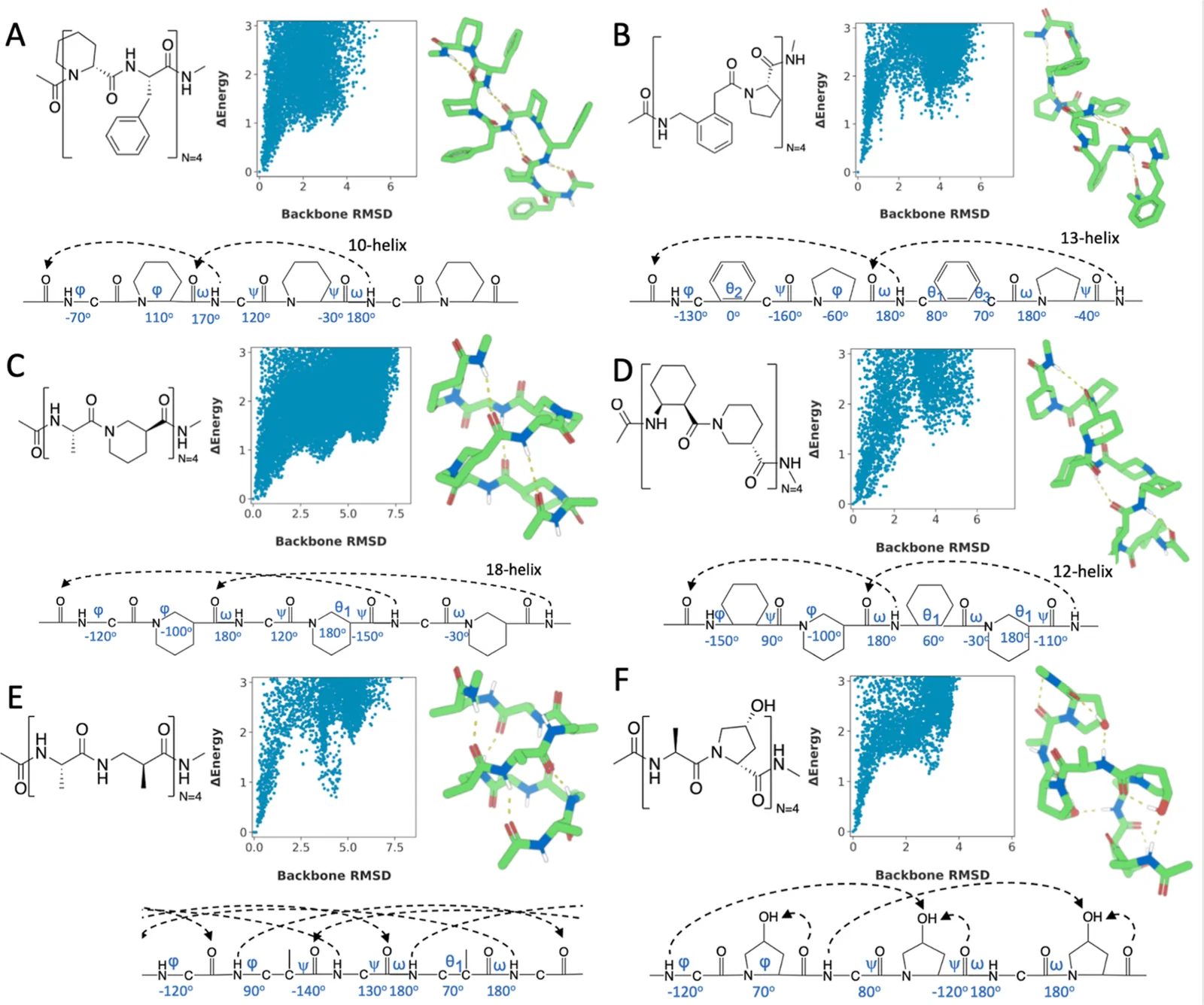
Designed (AB)_4_ secondary structures. (A–F) Chemical structure of the DPR unit, conformational ensemble, and predicted low energy structure for six computationally predicted examples of (AB)_4_ secondary structures, DPR1–DPR6. Nonpolar hydrogens were omitted for clarity, and hydrogen bonds are depicted as dashed lines. Some side chains were excluded for clarity from linear schematic. Backbone dihedral angles are rounded to the nearest 5°.

A variety of side-chain−side-chain and side-chain−backbone interactions stabilize the noncanonical repeating polymer structures. DPR6 and DPR8 have no backbone hydrogen bonds but are stabilized by two sets of backbone−side-chain hydrogen bonds per repeat unit. DPR7 and DPR10 each have a single backbone hydrogen bond per repeat unit and are further stabilized by side-chain−backbone hydrogen bonds (DPR7) or aromatic π-stacking interactions (DPR10). DPR9 has no hydrogen bonds and is solely stabilized by apolar interactions. Backbone tertiary amides provide rigidity, and designs with both cis and trans peptide bonds prior to the tertiary amide were favored if they resulted in favorable apolar or polar interactions. In contrast, DPR5 forms a mixed helix with backbone hydrogen bonds facing both directions (resembling an 18/16 backbone hydrogen-bonded conformation previously computed by Baldauf et al.^15^).

### Structural Characterization by Circular Dichroism, X-Ray Crystallography, and NMR Spectroscopy

The ten selected dipeptide-repeat polypeptides were synthesized with 3 or 4 repeating dipeptide units. We refer to these as 3×, 3.5×, or 4× DPRs, where “*n*×” denotes the number *n* of (AB)_*n*_ repeats in the polypeptide; in this notation, 1× denotes the simplest dipeptide fragment of 2 residues, 3.5× DPRs have 7 residues, and 10× DPRs have 20 residues. To screen for the presence of secondary structure in solution, circular dichroism (CD) spectra were collected on the 3×, 3.5×, or 4× DPRs in either water or CH_3_CN at low (5 °C) and high (95 or 75 °C) temperature (Figure S7). For 8 of these ten DPRs, these spectra indicated some solvent and/or temperature-dependent ordered structure. We attempted to crystallize all ten designs for X-ray structure determination, and obtained a structure for DPR1.

We next compared the CD spectra of the ten DPRs to theoretical spectra computed from the design model ensembles (Figure S7). The relatively small size of these polypeptides made spectral computation tractable at the full time-dependent density functional theory (TD-DFT) level, as shown, for example, for 4× DPR4 in Figure S8. Previous studies suggest that this method can correctly capture the overall CD signal of polypeptides.^28,29^ CD spectra were computed for selected low-energy and high-energy ensembles to simulate the effect of temperature-induced unfolding (shown, for example, in Figure S9). This approach, using AIMNet­(SMD)-D4 to generate structural ensembles, proved sufficient for accurate spectral calculations, eliminating the need for time-intensive MD simulations or the inclusion of explicit solvent molecules.^19,20^ For some of these DPR polypeptides, there is a good correlation between features of the calculated CD spectra of the low-energy ensembles and the experimental CD spectra (Figures 3 and S7). For these DPRs, the computed spectra for the high-energy “disordered” structure ensembles also have weaker intensities than the low-energy ensembles (Figure S9) and resemble the experimental spectra of the corresponding 1× DPRs (Figure S10).

**Figure 3:**
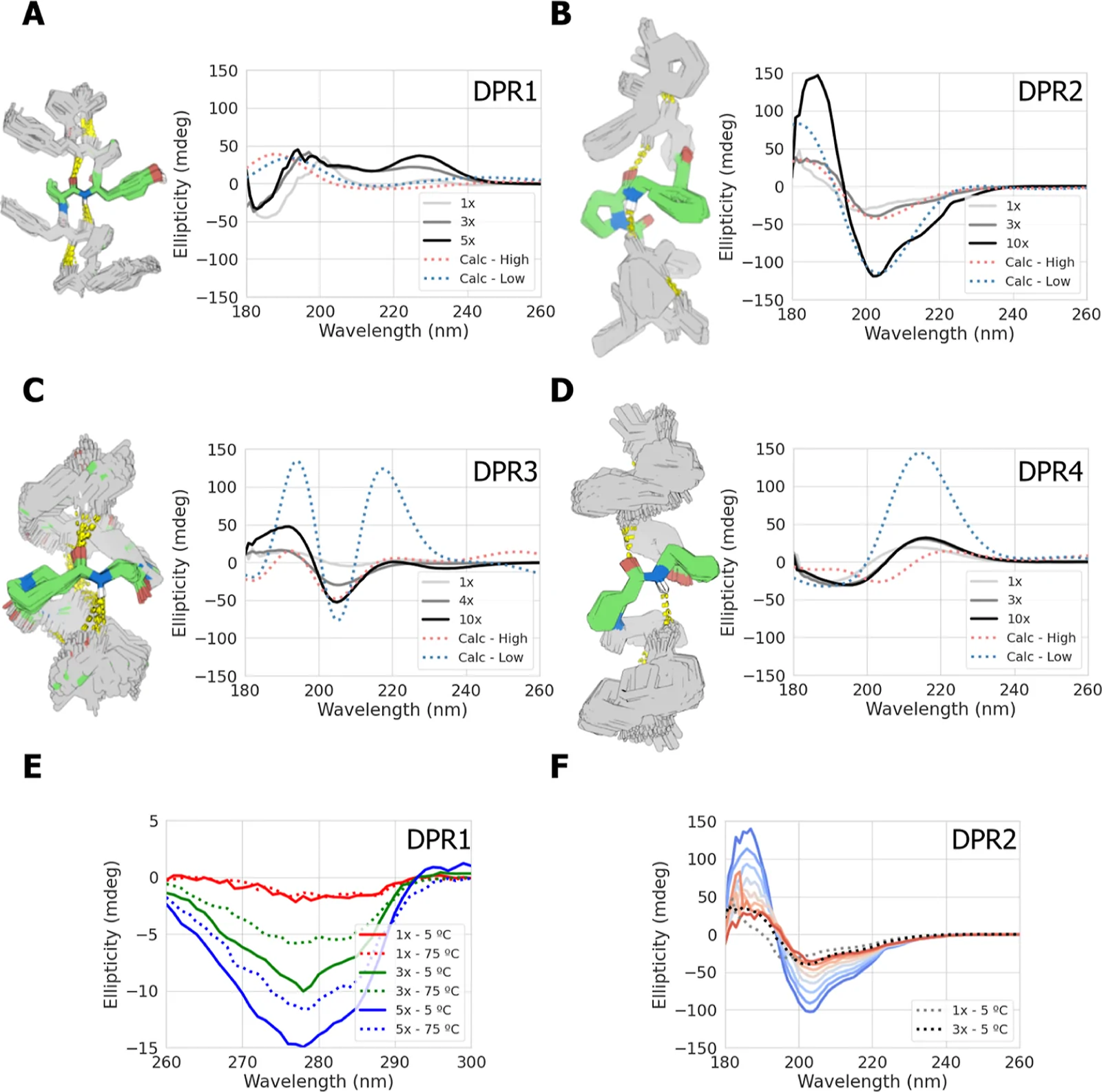
Experimental and calculated CD spectra. Low energy predicted ensemble (with nonpolar hydrogens excluded for clarity), experimental CD spectra (solid lines) at 5 °C in 2:1 ACN/H_2_O (A–C) and 100% ACN (D), and average theoretical spectra (dotted lines), with low energy spectra in blue and high energy spectra in red for (A) DPR1, (B) DPR2, (C) DPR3, and (D) DPR4. (E) Near UV CD spectrum of DPR1 1× (red), 3× (green), 5× (blue) at 5 °C (solid lines) and 75 °C (dotted lines). (F) Far UV spectra of 10× DPR2 at temperatures from 5 to 75 °C at 10 °C intervals (colored blue to red), showing increased unfolding at high temperatures, together with spectra of largely disordered 1× (gray dotted lines) and 3× (black dotted line) at 5 °C.

Based on the X-ray crystallography and CD screening results of DPRs in different solvents and at various temperatures, as well as comparisons between experimental and computed spectra, and between CD spectra computed for high vs low energy ensembles, we selected four designs (DPR1–4) for more detailed structural characterization. For these, we also synthesized the 1× dipeptide as a negative control that cannot form cooperative secondary structure and longer 10× polypeptides aimed at increasing the amount of ordered structure by helical cooperativity (for DPR1, we used instead a 5× polypeptide due to synthesis difficulties). Comparison of the far UV amide CD spectra (180–260 nm) of 1×, 3×, and 10× (or 5× for DPR1) revealed length-dependent CD features consistent with secondary structure formation (Figure 3A–D). These effects were particularly pronounced for DPR2 (Figure 3B). DPR2 and DPR3 display a positive band, and DPR1 and DPR4 show a negative band in the 180–190 nm region (ascribed to π → π* transitions), and DPR1, DPR3, and DPR4 show a positive band while DPR2 has a negative band at ∼220 nm (*n* → π*) (Figure 3A–D), all signatures of at least a subpopulation of molecules with conformational order. Additional length- and temperature-dependent near-UV aromatic CD (260–300 nm) studies of DPR1 and additional temperature-dependent far-UV CD studies (180–260 nm) of 10× DPR2 provide further evidence for secondary structure formation in these two polypeptides (Figure 3E,F).

We next characterized the structures of the four DPRs in more detail using solution NMR spectroscopy. NMR studies of repeating peptides are challenging due to the significant overlap of resonance signals from chemically identical residues, except where conformational features, such as end effects, lead to distinct magnetic environments. Chemical shift assignments of the 3×, 5×, and 10× DPRs were made using standard homonuclear and natural-abundance heteronuclear experiments and were aided by assignments for the 1× dipeptides used as unstructured negative controls. For the 5× and 10× DPRs, resonances could be assigned to specific residue and atom types (e.g., Tyr H^N^s or Pro Hδs), but the sequence-specific assignment to the particular repeat (e.g., the third vs the fourth repeated unit) was still not possible due to degeneracy and spectral overlap. For two of the designs, we were able to identify NOEs that were consistent with the characteristic repeating structures. Details of these NMR studies are presented in Supporting Information (Figures S11-S29 and Tables S5–S9), and the overall NMR and crystallography results are outlined in the following sections.

### DPR1

DPR1 is composed of alternating (heterochiral) l-Tyr and d-pipecolic acid (Pip) residues (Figure 4A). The design model is a right-handed helical conformation stabilized by a repeating pattern of backbone (*i*, *i* + 3) hydrogen bonds (Figures 2A and 4B–E), creating a series of alternating type II β-turns defined by characteristic backbone dihedral angles in the central *i* + 1 and *i* + 2 residues: ϕ = −60°, ψ = 120°, and ϕ = 80°, ψ = 0°.^30^ The resulting helical structure is further stabilized by (*i*, *i* + 1) CH−π polar interactions between the methylene Hδ2 proton of d-Pip and π electrons of the l-Tyr aromatic ring (Figure 4F). DPR1 is a “10-helix” with ten atoms making up the pseudo hydrogen-bonded ring, like the classical 3_10_ helix of α amino acids but with very different radius, pitch, and twist angles (Table 1). As a result, DPR1 forms a much longer and flatter helix compared to a 3_10_ helix with the same number of residues.

**Figure 4:**
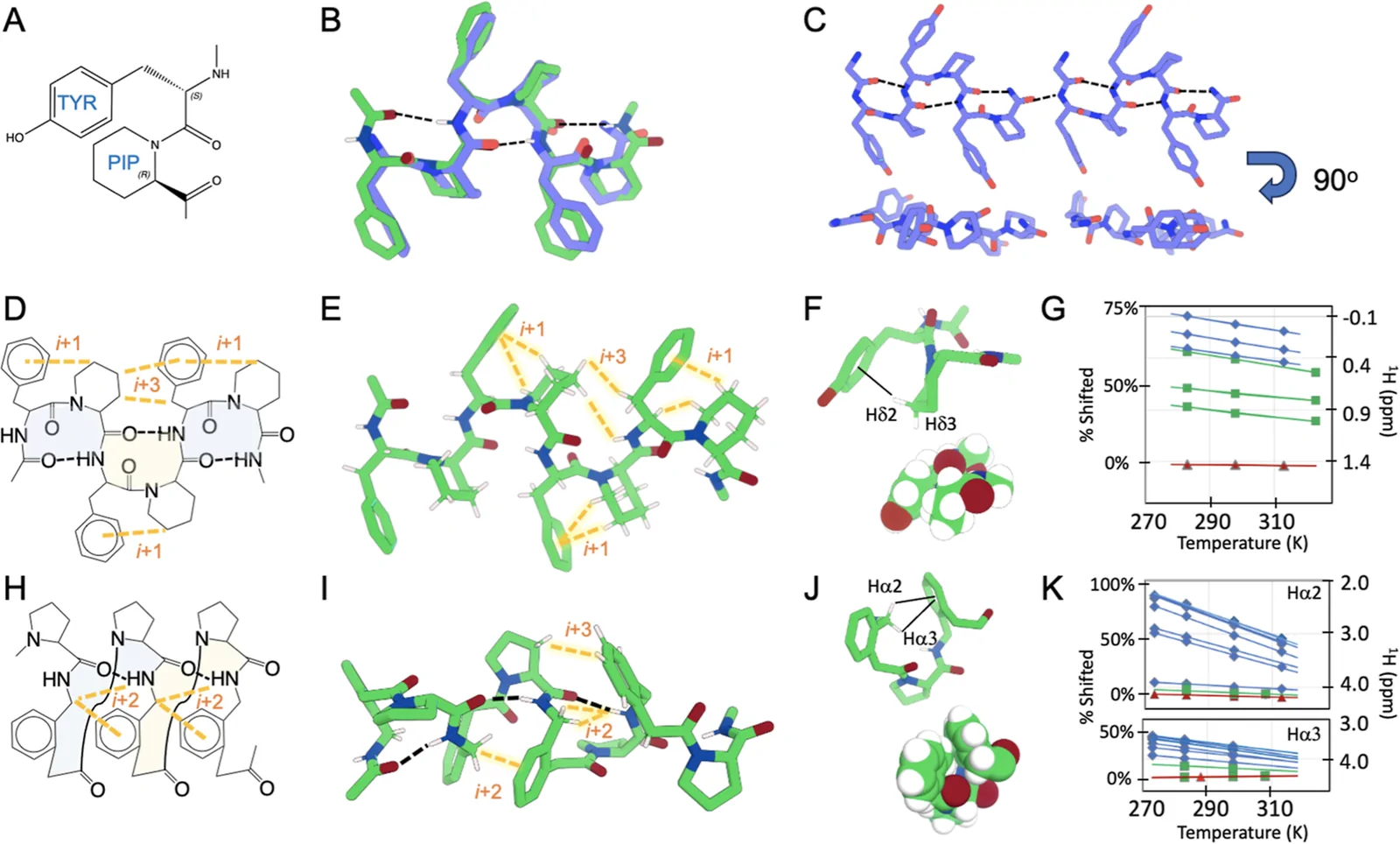
3D structure determination. (A) DPR1 dipeptide representation with l-Tyr and d-Pip. (B) The 0.9 Å crystal structure of 3× DPR1 (purple) overlaid with the design model (green, Cα RMSD of 0.46 Å). (C) Two symmetry-related helices from the crystal lattice in an extended head-to-tail arrangement resulting from the screw axis of symmetry (top) and rotated 90° to show the flat geometry of this secondary structure. (D) Schematic of DPR1 with repeating β-turns. (E) Design model of 4× DPR1 in stick representation with selected hydrogens shown. Key NOEs (*i*, *i* + 1 and *i*, *i* + 3) observed in the DPR1 5× 2D ^1^H–^1^H NOESY data at 10 °C shown as dotted yellow lines. Tyr OHs are excluded for clarity in (B,E). (F) Single dipeptide segment of the model structure shown as sticks (top) and as space filling models (bottom). The polar CH−aromatic interaction identified by the ring current shifts in NMR is shown as a solid black line. (G) Temperature and length-dependence of Pip Hδ2 chemical shifts in DPR1, color-coded by polymer length as in Figure 3E (i.e., 1× – red, 3× – green, 5× – blue). In the 5× ^1^H–^13^C HSQC NMR spectrum (Figure S13), some Pip Cδ−Hδ2 cross peaks overlap and therefore the proton counts for each peak are given in parentheses (Figure S15B). The percent shifted (*y*-axis) was calculated for each resolved proton by subtracting the 1× shift and dividing by the maximum expected ring current shift of 2.0 ppm. (H–K) are the corresponding data for DPR2. (I) NOEs of 10× DPR2 at 10 °C. (K) Temperature and length-dependence of AMPA Hα2 and Hα3 shifts in DPR2.

The 0.9 Å X-ray crystal structure of the mirror image enantiomer of 3× DPR1 (d-Tyr l-Pip) was determined in the *P*2_1_ space group (Figure 4B,C and Table S4); only the left-handed helical structure crystallized from a racemic mixture. The crystal structure closely matches the computational model with a Cα RMSD of 0.46 Å, including the designed hydrophobic side-chain interactions between the adjacent d-Tyr (*i*) and l-Pip (*i* + 1). Within the crystal lattice, there is an extensive network of water molecules and minimal intermolecular contacts, suggesting that the crystallographic structure was not influenced by crystal packing. Due to the screw axis of symmetry, the head-to-tail repeating arrangement of the helical peptides mimics a continuous helix (Figure 4C), complete with hydrogen bonds between their terminal ends, as would be observed in a longer continuous helix.

Solution NMR studies in acetonitrile and 2:1 acetonitrile/water solvents revealed increasing amounts of ordered structure as the number of DPR1 repeats is increased from 1× to 3× and 5× (Figure S11C,D), consistent with the length- and temperature-dependent CD study of Figure 3E. Detailed NMR studies of 5× DPR1 at the lowest temperature (10 °C) indicate the presence of a substantial population of the predicted helical structure in solution. This conclusion is supported by nine sequential (*i* + 1) NOEs, including five identified between the aromatic ring and Pip side chain, along with four nonoverlapping (*i* + 3) NOEs, characteristic of the designed helix (shown in Figure 4E, with NOESY data shown in Figure S14). These NOEs were either weak or absent in 3× DPR1 and became significantly stronger in 5× DPR1, particularly upon cooling from 25 to 10 °C (Table S5, Figure S14A,B).

Chemical shift and ^3^ *J*(HN-Hα) scalar coupling data also indicate cooperative helical structure formation at lower temperatures. In the unstructured 1× control, there is a mix of cis and trans Tyr-Pip peptide bond conformations (47% cis, 53% trans, Figures S11B and S12), whereas 5× DPR1 has exclusively trans d-Pip conformations (Figure S11E and S13). In addition, Tyr ^3^ *J*(HN-Hα) scalar coupling constants are smaller in the 5× compared to the 3× and 1× (trans conformation) DPR1s, and are even smaller in 5× DPR1 at lower temperatures (Table S6). The smallest scalar coupling constants for 5× DPR1 at 10 °C indicate some Tyr φ dihedral angles consistent with the predicted helical structure and characteristic of the repeating type II β-turn structural motif, although with a fraying at the N-terminal end of the molecule. Comparing the temperature dependence (ΔδHN/Δ*T*) of amide proton resonances in 5× and 1× DPR1 (Table S7), which reflects intramolecular hydrogen-bond formation, also indicates an equilibrium shift to more ordered conformations at low temperature (discussed further in Table S7). Analysis of the upfield shifts of axial Pip Hδ2 resonances resulting from ring-current shifts from the (*i*, *i* + 1) Tyr (Figure 4F), assuming 2.0 ppm as the maximum ring current shift for the Pip Hδ2, provides estimates of ∼40 and ∼65% ordered structure for 3× DPR1 and 5× DPR1, respectively, at 10 °C (Figures 4G and S15). These NMR data indicate a length- and temperature-dependent stabilization of conformation(s) that are fully consistent with the DPR1 design model.

The DPR1 poly­(II β-turn) helix was not identified in previous computational studies because repeating α/α amino acids with opposite chirality were not considered. DPR1 belongs to a class of helices sometimes called β-bend ribbon spirals, that are made up of repeating β-turns.^31^ Our crystal structure, to our knowledge, is the first with a poly­(II β-turn) helix and demonstrates the value of systematic consideration of side-chain interactions for overall stability (a related helical structure (d-Ala–l-Pro)_4_ was validated by NMR analysis in aprotic solvent).^32^ In DPR1, the turn conformations are stabilized by closely packed hydrophobic side-chain interactions and the CH−π polar interaction between the neighboring l-Tyr and d-Pip side chains. The selected piperidine ring, rather than a proline pyrrolidine ring, provides distinct torsional preferences, resulting in an increased pitch compared to proline analogs. The stability of the designed DPR1 poly­(II β-turn) 3.7_10_ helix in acetonitrile–water mixtures suggests that it may be more stable than previously determined repeating poly­(II β-turn) helices.^32^

### DPR2

DPR2 is a repeat of the δ/α dipeptide AMPA–l-Pro, where AMPA represents the noncanonical δ amino acid ortho­(aminomethyl)­phenylacetic acid. AMPA is an achiral aromatic amino acid with constrained backbone structure. The DPR structure is an *i*, *i* + 3 hydrogen bonded, 13-helix (Figures 2B and 4H–J). AMPA has an unusual cis-constrained backbone conformation (θ_2_ = 0°) due to the Cα and Cδ backbone atom’s connection to the ortho positions of its aromatic ring. The resulting backbone kink results in a 2.7_13_ helical structure with a significant backbone twist to accommodate its hydrogen bonds, enabling the formation of packing interactions and burial of nonpolar surface areas. The DPR2 design has stabilizing CH−π interactions between AMPA CαH_2_ protons and the aromatic ring of the AMPA in the *i* + 2 position, which are ∼3 Å apart (Figure 4I,J), resulting in significant buried nonpolar surface area.

Solution NMR studies of DPR2 in acetonitrile and 2:1 acetonitrile/water solvent show increasing amounts of resonance dispersion between 1×, 3×, and 10× due to increasing amounts of structure (Figures S16 and S17), and consistent with the length- and temperature-dependence observed in the corresponding CD study (Figure 3B,F). Detailed NMR studies of 10× DPR2 at the lowest temperature (10 °C), including four NOEs (Figures 4I and S18, and Table S8), indicate the presence of a substantial population of the designed model conformation in solution. Ring-current shifts on AMPA methylene Hα2 and Hα3 protons provide dispersion between these resonances of different AMPA residues, allowing the assignment of NOEs involving these resonances. For all DPR2 lengths, the intraresidue distances H^N^ (*i*) to Hα2 (*i*) and H^N^ (*i*) to Hα3 (*i*) are short (2.5 and 3.0 Å, respectively) as they are only four bonds apart, while the key conformation-dependent H^N^ (*i* + 2) to Hα2/Hα3 (*i*) distances are also short (4.0 and 2.6 Å, respectively) in the 2.7_13_ model helix. For 10× DPR2, overlaying the TOCSY with the NOESY spectra allowed for identification of NOESY cross peaks assigned to these (*i* + 2) conformation-dependent NOEs (Figure S18 and Table S8). Additionally, we identified *i* + 2 and *i* + 3 NOEs from the AMPA aromatic ring protons that are characteristic of the predicted DPR2 helical structure (Figure 4I and Table S8).

The temperature dependence of ring current shifts and amide NH chemical shifts for DPR2 also indicate increased population of the helical structure at lower temperatures (Table S9). Although both 1× and 10× DPR2 have both cis and trans AMPA-Pro peptide bond conformations in the same ratio at 25 °C (18% cis, 82% trans, Figure S16), in 10× polymer, this increased to 92% trans upon cooling (Figure S19). The two AMPA CαH_2_ protons are shifted significantly upfield compared to 1× at the same temperature with larger upfield shifts at lower temperatures (Figures 4K and S17, S20, and S21C), consistent with length- and temperature-dependent stabilization of conformational states. In the predicted helical DPR2 structure, both AMPA methylene CαH_2_ atoms are positioned above the neighboring AMPA (*i* + 2) aromatic ring (Figures 4J and S21D), resulting in the observed ring current shifts on these protons. We estimate the average ordered structure across the 10× DPR2 sequence to be 75% at 0 °C in 2:1 acetonitrile/water, based on ten different AMPA Hα2 peaks which range from 11 to 93% shifted (discussed in Figure S21).

The δ/α (AMPA/l-Pro)_*n*_ sequence of DPR2 has *i*, *i* + 3 hydrogen bonding, resembling a previously predicted (but not experimentally characterized) δ/α 13-helix,^17^ yet has distinctive features. DPR2 has a proline that lacks the HN required for sequential repeating *i*, *i* + 3 hydrogen bonds and has the unusual δ-amino acid AMPA with a cis-constrained peptide bond conformation, resulting in a kinked backbone structure. The DPR2 helix has a smaller radius and a longer pitch than the predicted α/δ 13-helix, which has backbone dihedral angles for the δ amino acid that are analogous to two α residues in an α helix.^17^ Besides DPR2, we are not aware of any previously determined structures that incorporate the sterically hindered AMPA building block into a δ/α DPR, although there is a structure of polymeric AMPA that forms a distinctly different 10-helix structure with *i*, *i* + 1 hydrogen bonds.^33^

### DPR3 and DPR4

DPR3 is an α/β DPR of alanine and nipecotic acid (Nip), a pipecolic acid isomer with a tertiary amine and β-amino acid backbone, with an unusual cis Ala–Nip peptide bond. The predicted helical structure has long-range repeating hydrogen bonds (18-helix) between Ala H^N^ (*i* + 5) and Nip CO (*i*), with a larger diameter helix than the standard α helix (Figure 2C). There are stabilizing hydrophobic contacts between the nonaromatic rings of the Nip side chains in positions *i* and *i* + 4, resulting in stacking of these Nip rings along the length of the helix. DPR4 is a β/β DPR of ACHC–Nip, where ACHC designates (1*S*,2*R*) 2-aminocyclohexane carboxylic acid. The DPR4 secondary structure also has cis X-Nip peptide bonds and is stabilized by repeating backbone hydrogen bonds between ACHC H^N^ (*i* + 3) and Nip CO (*i*) (Figure 2D). With two β amino acids, there are 12 atoms in the ring formed by the hydrogen bond, and the backbone forms a helix with an inner diameter that is smaller than the standard α helix. DPR4 is stabilized by hydrophobic contacts between the rings of ACHC (*i*) and Nip (*i* + 3) that form a stack of alternating rings along three sides of the helix.

Although the experimental CD data for 10× DPR3 and DPR4 are each consistent with theoretical CD spectra of the low energy ensemble (as outlined above), using solution NMR, we were not able to observe any *i* + 2 or *i* + 3 NOEs distinct from intraresidue and sequential peaks. Unfortunately, neither DPR3 nor DPR4 have aromatic residues to allow for stabilizing CH−π polar interactions or spectroscopically helpful ring current shifts, as were observed in DPR1 and DPR2. Both 1× DPR3 and 1× DPR4 had a mixture of cis and trans X-Nip populations, with just over 50% cis for both DPR3 (Figures S22 and S23) and DPR4 (Figures S26 and S27). Using these assignments as a guide, we determined that there is also a mix of both cis and trans conformations in the longer 10× peptides (and 20× DPR3), along with ROESY cross peaks between the cis and trans resonances indicating slow conformational exchange (Figures S25 and S29). The H^N^ resonances for the 10× of both DPR3 and DPR4 were downfield-shifted compared to the 1× at the same temperature, indicating increased hydrogen bond propensities in the polymers. However, due to the complexity and overlap, we were unable to determine whether the conformations with cis peptide bonds observed for 10× or 20× DPR3 (Figures S24 and S25) or for 10× DPR4 (Figures S28 and S29) correspond to the predicted helical structures with cis peptide bonds.

The DPR3 and DPR4 designs both have unusual cis-peptide bond configurations prior to the constrained tertiary amide of nipecotic acid. As for DPR1 and DPR2, there is only one backbone hydrogen bond for each DPR unit, and additional stabilization comes from apolar interactions of the side chains along the outside of the helices. Neither the 4.6_18_-helix of α/β DPR3 nor the 3.2_12_-helix for the β/β DPR4 have been previously described to our knowledge.

## Conclusions

We describe the systematic search for folded repeating structures stabilized by the backbone and side-chain interactions of noncanonical amino acids. Our large-scale search for DPR sequences predicted to have single global energy minima revealed a diverse set of new structures stabilized by combinations of backbone and side-chain hydrogen bonding, conformational restriction using cyclic side chains, and inter-repeat polar and hydrophobic contacts (Figure 2, Tables 1, and S2 and S3). Our approach systematically explores all of the repeating structures for which AIMNet calculations of the monomer configurations are low in energy (e.g., Table S11). Our results go beyond previous backbone-centric approaches^8,15–17,23,24,34,35^ by including the energetics of a wide array of possible side-chain−backbone and side-chain−side-chain interactions. Our hierarchical design approach can be extended to other large chemical spaces such as cyclic poly amides and peptides.^36^ Our approach does have limitations: first, we would miss repeating structures for which intraresidue interactions are sufficiently strong to stabilize the monomer in relatively high energy conformations, second, we would miss low-energy conformations that do not have perfect repeating, helical symmetry, and third our implicit solvent calculations do not model the solvation shell explicitly.

Likely because side-chain−side-chain and side-chain−backbone interactions are considered explicitly in our investigation, four of the ten experimentally characterized DPRs have some ordered structure consistent with the designed models as assessed by comparison of experimentally measured and computed CD spectra. Further characterization of two DPRs revealed structures that were nearly identical to the design models, supported by X-ray crystallography and NMR data analysis. NMR data analysis also demonstrated a significant dynamic population of the design models in solution that became more ordered at longer lengths and lower temperatures. Studies of the length dependence of our new structures suggest that, like the α-helix, a critical minimal length is required to overcome the nucleation barrier.

Taken together, our results support the hypothesis that repeating structures built up from low-energy monomers can be effectively stabilized by side-chain conformational biases arising from cyclic geometries, side-chain-backbone steric interactions, side-chain−side-chain hydrophobic packing, and hydrogen bonding along the polypeptide chain. These noncanonical amino acid-based DPR structures provide useful new and robust structural motifs for the construction of new materials and macromolecules, with applications such as scaffold for catalysis, atomically precise molecular wires, and high-strength, elastic polymer materials.

## Materials and Methods

### Computational Methods

#### Monomer Sampling and Convergence

The performance of the monomer sampling protocol was monitored by evaluating the Boltzmann-weighted Kullback–Leibler (KL) divergence of the potential energy surfaces generated from a linear interpolation of the Delaunay triangulation (Figure S2). The VABLAS protocol consistently achieved convergence of the potential energy surface of the monomeric units with less sampling compared to random sampling. The relative rate of convergence between the two methods was increased with the number of dimensions and conformational rigidity. The rate of convergence for the adaptive sampling technique was approximately constant with respect to conformational rigidity and 10^*N*^, where *N* is the number of dimensions. However, the scaling of the process with the number of dimensions is limiting. We find that the practical limit for this technique is 5 to 6 dimensions (Figure S3) due to the increased time needed to update the triangularization in higher dimensions. That is why this technique was not used to sample the multiresidue secondary structures directly. The distribution of sampled points shows that the adaptive sampling technique successfully focused sampling on the lower energy and more relevant points. Additional details are presented in the Supplemental Monomer Sampling Protocol.

#### Polymer Sampling and Selection

For the 20,475 combinations of dipeptides, we calculated the free energy gap between the overall lowest energy conformation of the polymer and the lowest energy conformation of the polymer with RMSD > 2.0 Å with the overall lowest energy conformation (See Figure 1 for a graphical explanation). Initial analysis of this free energy gap for previously characterized and validated DPR peptides that were sampled in our large-scale screening, suggested that a gap of 0.66 kcal/mol/residue was a reasonable threshold for proceeding to experimental validation (See Figure S5). This binary filter was more stringent than statistical mechanical partition function analysis because it is not sensitive to undersampling of alternative low energy states. Approximately 10% of potential combinations passed this filter; however, half of these passing designs are combinations with only α amino acids (side-chain and chirality variations) because they were highly represented in the monomer building block library. The final selection of dipeptide sequences for experimental testing was evaluated for novelty of sequence and structure compared to the previous literature, as well as among the selection. After experimentation, we suggest a more stringent threshold of 0.8 kcal/mol/residue and 1.5 Å RMSD, which is approximately 5% of the sequences. The lowest energy structures are provided as PDB files, along with CSV files of the delta energy vs RMSD for all 20,475 calculated sequences, including the most promising 918 designs. These are available in a Zenodo data deposition (DOI 10.5281/zenodo.12510622) to facilitate further research. Note that a significant false positive rate, similar to this study, should be expected from these designs. Additional details are presented in the Supplemental Polymer Sampling Protocol.

#### Computational Prediction of CD Spectra

CD spectra were calculated through the TD-DFT formalism. Peptide geometries were those obtained from AIMNet­(SMD)-D4 for 4× lengths (i.e., (AB)_4_) without further optimization. For each peptide, calculations were performed on 40 and 30 low- and high-energy conformers that were sampled from the population of polymers with delta energy ≤1 kcal/mol/residue and between 1 and 6 kcal/mol/residue, respectively. Two final high- and low-energy CD spectra were calculated by averaging the sets of spectra to take conformational heterogeneity into account.

Calculations were performed at the TD-CAM-B3LYP/6-31G* level, including solvent effects through the Polarizable Continuum Model (PCM), as implemented in Gaussian 16. Benchmark calculations were performed with other functionals or basis sets, although all of them produced qualitatively identical results (see Figure S7). Acetonitrile was employed as the solvent in the PCM model; using water as the solvent yielded identical spectra. For peptides with aromatic side chains, 64 excited states were calculated, while 32 states were calculated for those without aromatic side chains.

To facilitate comparison and better match experimental CD spectra, calculated stick spectra were red-shifted by 0.6–1.0 eV and broadened using Gaussian line shapes with half widths at half height between 1750 and 3000 cm^–1^ (the specific values were adjusted for each peptide). The calculated intensities were scaled to approximately match the experimental ones. The corresponding data for these calculations has been deposited in the ioChem-BD database^37^ and is accessible through the DOI 10.19061/iochem-bd-6–339.

## Experimental Methods

### Synthesis of Selected DPRs

Distinct lengths of dipeptide-repeat polymers were synthesized using standard solid-phase peptide synthesis by WuXi AppTec. In order to reduce potential electrostatic interactions resulting from N- and C-terminal end effects, DPRs were synthesized with (1) either N-terminal acetylation (CH_3_–CO−) or an N-terminal glycine ([NH_3_^+^]–CH_2_–CO−) and (2) C-terminal amidation (−NH_2_). The series of 3× peptides were preemptively synthesized with an N-terminal glycine to promote solubility while placing the charged ammonium further along the tail of the molecule. Following synthesis, all peptides underwent purification and characterization using HPLC and LC–MS, achieving purities greater than 95%. Full characterization of compound purities can be found in Table S10.

### CD Data Collection

The Far-UV CD experiments were performed on a Jasco J-1500 at 0.17 mg/mL from 1 to 10 mg/mL stocks of 50:50 water/acetonitrile with 1 mm path length cuvettes for all samples. Solvents tested include 100% deionized water, 100% acetonitrile, and the mixture of 2:1 acetonitrile/water. Spectra were averaged three times. All temperature changes were equilibrated for 5 min. Experiments were repeated with consistent results. The near-UV CD experiments (260 to 300 nm) were collected on 1×, 3×, and 5× DPR1 at 5 and 75 °C and 2 mg/mL in 2:1 acetonitrile/water.

### Crystallization and Diffraction

3× DPR1 crystallized from a 0.5 mL solution (20 mg/mL) by slow evaporation in a humidity-controlled room with a 50:50 acetonitrile/water mixture. Diffracting crystals, composed of the left-handed helix formed by d-Tyr and l-Pip enantiomer repeats, developed over three months. Crystal diffraction data was collected from a single crystal using APS beamline 24ID-C at 100 K. Unit cell refinement and data reduction were performed using XDS and CCP4 suites.^38,39^ The structure was identified by direct methods and refined by full-matrix least-squares on *F* ^2^ with anisotropic displacement parameters for the non-H atoms using SHELXL-2018/3.^40,41^ Structure analysis was aided by using Coot/ShelXle.^42,43^ The hydrogen atoms on heavy atoms were calculated in ideal positions with isotropic displacement parameters set to 1.2 × *U*_eq_ of the attached atoms. The structure has been deposited in the Cambridge Structural Database, CSD (Reference code 2338589), and the refinement statistics are given in Table S4.

### NMR Data Collection

NMR samples were prepared by dissolving 2 to 10 mg of the polymers in acetonitrile-*d*_3_ or acetonitrile-*d*_3_/water mixtures using Milli-Q filtered water. 5× DPR1 and 10× DPR2 were dissolved in a 2:1 mixture of acetonitrile-*d*_3_/water with sonication to aid in dissolution and increase solubility. Volumes of 600 to 800 mL were loaded in 5 mm tubes for NMR data collection; a few samples were cloudy due to solubility limitations.

NMR spectra were collected at several temperatures ranging from 0 to 75 °C with a 5 mm TCI CryoProbe on either a Bruker Avance III 600 MHz or Bruker Avance Neo 800 MHz spectrometer. Spectra included 1D ^1^H spectra and 2D NOESY (200 ms), ROESY (200 ms, 6000 Hz), TOCSY (80 ms), COSY, ^1^H–^15^N HSQC, ^1^H–^13^C HSQC, H–^13^C TOCSY-HSQC (60 ms), and ^1^H–^13^C HMBC. All spectra were referenced to internal TMS present in the acetonitrile-*d*_3_ at 0.03% (v/v). ^3^J­(HN-Hα) scalar couplings were measured in 1D spectra when not prohibited by overlap. The temperature dependence of amide proton chemical shifts was measured from 1D and 2D ^1^H–^15^N HSQC or TOCSY spectra at variable temperatures (0 to 75 °C). This analysis excluded overlapped peaks. Temperature coefficients, ΔδH^N^/Δ*T* (slope), were derived through linear regression using Microsoft Excel. Spectra were processed using TopSpin 4.0 (Bruker) or NMRPipe software and visualized using NMRFAM-SPARKY.^44^ The NMR validation data and models have been deposited in PDB_Dev database with accession codes 00000375 for DPR1 and 00000376 for DPR2.

## Supplementary Material



## Notes

Computational method support (schematics, figures, and code). Experimental data (spectra, biophysical data, X-ray crystal statistic) and analysis (methods, figures, and tables) (PDF)
