# Supplementary material for: Enumerative Discovery of Noncanonical Polypeptide Secondary Structures
Source: J Am Chem Soc. 2024 Sep 4;146(37):25501–12. doi: 10.1021/jacs.4c04991 (PMC11421003; doi:10.1021/jacs.4c04991)
Supplement: Supplementary file 1 [file ja4c04991_si_001.pdf]

Enumerative Discovery of Non-Canonical Polypeptide Secondary Structures

Adam P Moyer[1,‡], Theresa A Ramelot[2,‡], Mariano Curti[3], Margaret A Eastman[4], Alex Kang[1], Asim K Bera[1], Roberto Tejero[2], Patrick J Salveson[1], Carles Curutchet[5, 6], Elisabet Romero[3], Gaetano T Montelione[2,*], David Baker[1,*]

[1] Department of Biochemistry and Institute for Protein Design, University of Washington, Seattle, WA, 98195, USA

[2] Department of Chemistry and Chemical Biology, Center for Biotechnology and Interdisciplinary Studies, Rensselaer Polytechnic Institute, Troy, New York, 12180 USA

[3] Institute of Chemical Research of Catalonia (ICIQ-CERCA), Barcelona Institute of Science and Technology, Av. Països Catalans 16, Tarragona 43007, Spain

[4] Department of Chemistry, Oklahoma State University, Stillwater, OK 07478 USA

[5] Departament de Farmàcia i Tecnologia Farmacèutica, i Fisicoquímica, Facultat de Farmàcia i Ciències de l'Alimentació, Universitat de Barcelona (UB), Av. Joan XXIII 27-31, 08028 Barcelona, Spain

[6] Institut de Química Teòrica i Computacional (IQTCUB), Universitat de Barcelona (UB), Martí i Franqués 1, 08028 Barcelona, Spain

‡ Co first authors

* To whom correspondence should be addressed: monteg3@rpi.edu; dabaker@uw.edu

Supporting Information - Table of Contents

Supplemental Computational protocols

- Monomer sampling protocol.. 4
- Polymer sampling protocol... 6

Supplemental Discussion

- Expanded discussion of methodology limitations and future work…………………. 7

Supplemental Figures

S1 Volume and Boltzmann Loss Adaptive Sampler (VABLAS) flowchart 8
S2 Performance of Delaunay Triangulation with number of dimensions 9
S3 Convergence of monomer sampling 10
S4 Distribution of properties of the monomeric residues 12
S5 Comparison of computational predictions to known helices 13
S6 Helix schematics for DPR1-DPR10 models 21
S7 Experimental CD spectra of 10 DPRs 23
S8 CD spectra calculated with different functionals or basis sets 25
S9 Calculated CD spectra for low- and high-energy ensembles 26
S10 Calculated CD spectra for different polymer lengths 27
S11 DPR1 1D 1H spectra and ^{1}H-^{15}N HSQCs 28
S12 1x DPR1 2D ^{1}H-^{13}C HSQC and Pip chemical shift assignments 29
S13 1x and 5x DPR1 chemical shifts change with temperature 30
S14 5x DPR1 5x NOESY data at 298 and 283 K 31
S15 DPR1 NMR ring current shifts 32
S16 DPR2 1D spectra and ^{1}H-^{15}N HSQCs 33
S17 1x and 10x DPR2 ^{1}H-^{13}C HSQC spectra 34
S18 10x DPR2 ^{1}H-^{1}H NOESY and TOCSY 35
S19 10x DPR2 ^{1}H-^{13}C HSQCs showing percentage of cis at decreasing temps. 36
S20 10x DPR2 changes with temperature 37
S21 10x DPR2 ^{1}H-^{1}H TOCSY and AMPA peak shifts with temperature 38
S22 1x DPR3 1D ^{1}H NMR 39
S23 1x DPR3 COSY, ROESY, HSQC NMR 40
S24 10x/20x DPR3 COSY NMR 42
S25 20x DPR3 COSY, ROESY, HSQC NMR 44
S26 1x DPR4 1D ^{1}H NMR 46
S27 1x DPR4 COSY, ROESY, HSQC NMR 47
S28 10x DPR4 COSY NMR 49
S29 10x DPR4 COSY, ROESY, HSQC NMR 51

Supplemental Tables:

Table S1 Complete residue list (SMILES format) and calculation of unique dipeptide combinations 53
Table S2 Helical parameters for DPR5 - DPR10 models 86
Table S3 Dihedral angle parameters for ideal models DPR1 - DPR 10 87
Table S4 X-ray data collection and refinement statistics 88
Table S5 DPR1 NOE summary table 89
Table S6 DPR1 $^{3}J(HN\text{-}H\alpha)$ scalar coupling constants at 298 and 283 K 90
Table S7 DPR1 amide temperature coefficients 91
Table S8 DPR2 NOE summary 92
Table S9 DPR2 amide temperature coefficients 93
Table S10 HPLC and Mass Spectrometry validation 94
Table S11 Calculated relative energies for DPR1 and DPR2........ 110

Supplemental Code

Code S1 boltzmann_adaptive_sampler.py 111
Code S2 run_residue_sampling.py 115
Code S3 run_polymer_sampling.py 119

Supplemental References 124

Supplemental Computational Protocols

Supplemental Monomer sampling protocol

1. Identify all significant continuous degrees of freedom.
    a. For example, the phi and psi torsions are the significant degrees of freedom of α-amino acids.
    b. For residues with sidechains, the sidechain torsions are also considered to be significant degrees of freedom.
2. Generate an initial conformer for each amino acid.
    a. We used the Cambridge Structural Database conformer generator, ConfGen, which leverages data from small molecule crystal structures.
    b. If the residue has discrete degrees of freedom such as ring puckering, each discrete degree of freedom must be processed independently.
    c. All residues were parameterized with an N-terminal acetylation
    d. All residues were parameterized with two different C-terminal modifications: C-terminal methyl-amidation and C-terminal dimethyl-amidation.
    e. Residues with C-terminal dimethyl-amidations are designed to mimic the context of pre-proline
3. Initialize the adaptive sampling process by sampling and recording the energy of the residues on a sparse grid of the degrees of freedom.
    a. Energy was recorded after minimizing (AIMNet(SMD)-D4) the residue to convergence (0.1 kcal/mol) with constraints on the significant degrees of freedom. To clarify, all bond lengths and bond angles were free to optimize.
4. Calculate a Delaunay Triangulation using SciPy of all sampled points.
    a. The N-dimensional values of the degrees of freedom are the coordinates for the points in the Delaunay Triangulation
5. Calculate the Volume Adjusted Boltzmann Probability (VABP) of each simplex of the Delaunay Triangulation
    a. The Volume Adjusted Boltzmann Probability (VABP) is defined as:
        i. $Loss_i = V_i * mean(exp(\Delta E_{0\text{->}N+1} / -kT))$
        ii. $V_i = det(X_{0\text{->}N} - X_{N+1}) / N!$
    b. This function calculates the Boltzmann probability of each simplex weighted by the “volume” of the simplex.
    c. Intuitively, simplices with high volume will have a high loss and simplices with low energy/high Boltzmann probability will have a high loss.
6. Select N simplices with the highest loss, select a point within each simplex, and evaluate the energy of the residue at those points.

a. Intuitively, simplices with the lowest energy will be selected, but that selection is balanced by the reduction of volume of the simplices by sampling those regions.
b. The number of points evaluated on each loop is a balance between the time to re-calculate the Delaunay Triangulation, and the quality of the adaptive sampling. In the extremes: If you select 1 point per loop, you will sample the optimal point each round, but you will need to recalculate the Delaunay Triangulation every time, which is time consuming. If you select every simplex, you will be uniformly sampling.
c. The number of points sampled each round should be determined by how fast you can evaluate the molecular function and how fast you are calculating the Delaunay Triangulation. I dynamically adjusted this number to maintain 99% of the runtime evaluating the molecular functions.

7. If the total number of sampled points is less than 10*10^N, where N is the total number of degrees of freedom of the residue, return to step 4, else stop. Empirically, Evaluated the KL Divergence of the potential energy surface. If the potential energy surface is not converged, return to step 4, else stop.

Supplemental Polymer sampling protocol

1. Generate the list of all combinations of unique dipeptide sequences taking into account chirality and permutation of sequence (AB vs BA is identical with helical symmetry)
2. Sampling Stage 1 - Naive Sampling
    a. Load the database of both monomer ensembles/potential energy surfaces
    b. Randomly select N conformations for each monomer biased by the Boltzmann probability given the energy of the monomer unit.
        i. KT was 1.2 kcal/mol
    c. Generate the helically symmetric structure for N conformations by assembling the $(AB)_4$ polymer
    d. Calculate a crude energy to detect clashing of the polymer conformation
    e. Discard conformations with clashes
    f. Evaluate the energy of the remaining conformations
    g. Record the energy, degrees of freedom, and coordinates of the evaluated conformations
3. Sampling Stage 2 - Biased Sampling
    a. Load the database of previously sampled polymer conformations
    b. Randomly select N/10 conformations of the polymer that have already been sampled biased by the energy of the polymer conformations
        i. KT was 0.6 kcal/mol/residue
    c. Select the 10 nearest neighboring points/dihedral angles from the monomer ensembles
    d. Generate the helically symmetric structure for N conformations by assembling the $(AB)_4$ polymer
    e. Calculate a crude energy to detect clashing of the polymer conformation
    f. Discard conformations with clashes
    g. Evaluate the energy of the remaining conformations
    h. Record the energy, degrees of freedom, and coordinates of the evaluated conformations
4. Perform steps 2 and 3 iteratively until 5,000 - 50,000 conformations have been evaluated

Supplemental Discussion

Expanded discussion of methodology limitations and future work

By comparing known structures that have previously been solved by crystallography with computationally predicted structures using our protocol, we can investigate the false negative and identify limitations. Fundamentally, this study attempts to predict the equilibrium structure of non-canonical residues, addressing two core challenges: exhaustive sampling of the conformational space and accurate evaluation of energetic contributions. Both tasks are practically impossible to do without simplifications and approximations that reduce the complexity of the simulations.

For sampling, we adopted a hierarchical approach with high-resolution sampling density at the monomer level and progressively sparser sampling at the polymer level. This hierarchy focuses the sampling into the most promising low energy regions. However, interactions in the global context of the polymer chain can theoretically overcome local monomeric conformations. Also, sampling included strict helical symmetry which does not model slightly irregular helices. Sampling these would exponentially increase the configuration space and significantly more sampling.

Accurately and quickly scoring the energetics of molecular systems is a grand challenge in molecular design. Functions like DFT predict molecular energies accurately but are time-consuming for large systems (such as the ones in this study which can reach over 100 atoms). These slow score functions will proportionally limit the amount of sampling that can be achieved with the same amount of computational resources. Fast score functions like molecular mechanics do not account for many important weak interactions and rely on tuning parameters, limiting their scope. This constraint on using efficient score functions also prohibits accounting for other important energetic contributions such as vibrational modes and explicit solvent interactions which can have significant contributions to the energetics of folding.

Deep learning methods for sampling and scoring of arbitrary molecular systems are a promising direction, but are currently limited by the need for relevant, high-quality training data. However, these approaches offer an attractive compromise between speed and accuracy.

Supplemental Figures

Figure S1. Volume and Boltzmann Loss Adaptive Sampler (VABLAS) flowchart

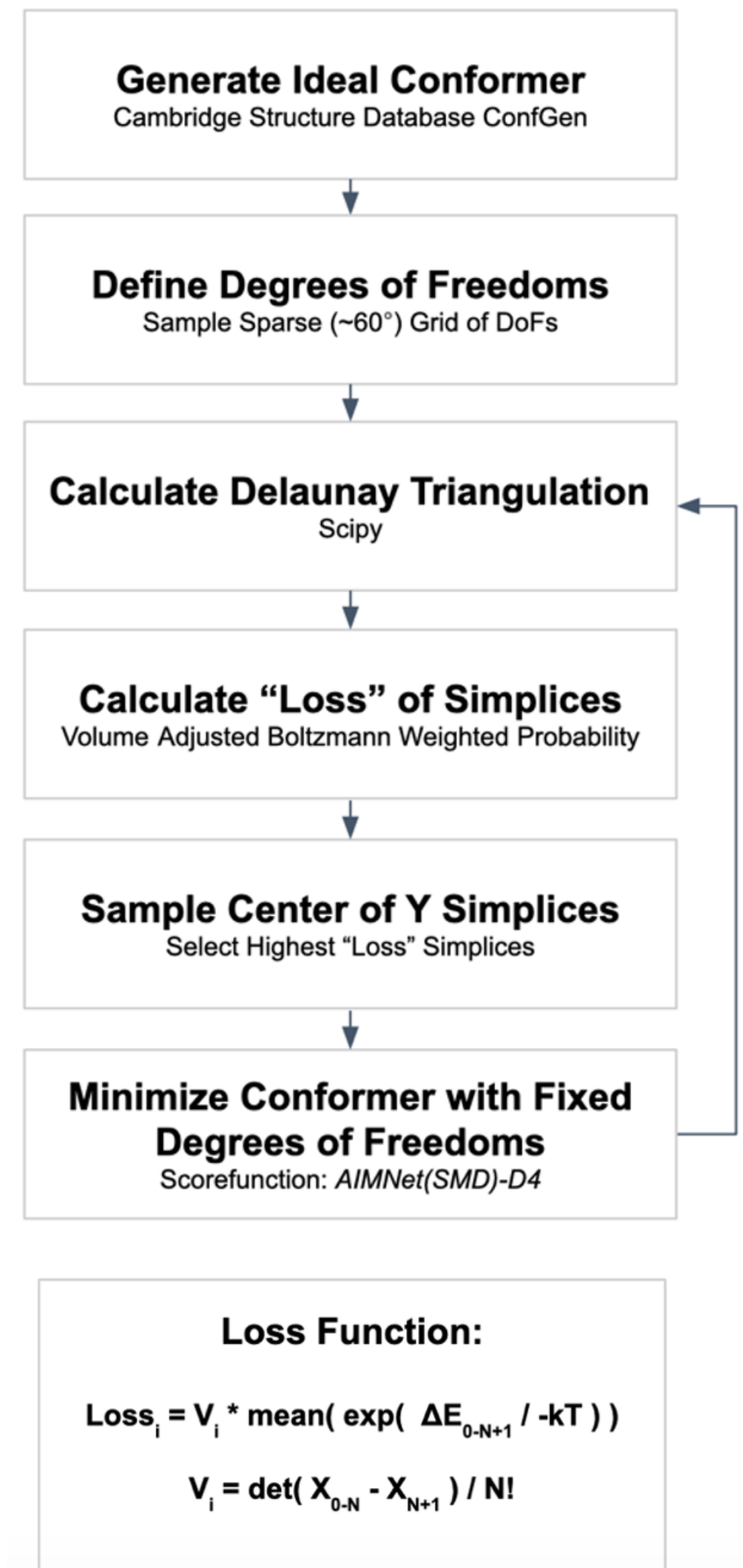

Process flow diagram for Boltzmann Adaptive Sampling (VABLAS) protocol for monomeric residues. The process iterates indefinitely until sufficient sampling is achieved, which is checked periodically. Heuristically, we found that $10*10^N$ samples are sufficient where N is the total number of degrees of freedom. The number of centers sampled per iteration (Y) should be dynamically controlled to balance the time calculating the Delaunay triangulation and evaluating the energy of molecules. The loss function is the Volume Adjust Boltzmann Weighted Probability, which is calculated by multiplying the volume of a simplex by the mean of the unnormalized Boltzmann probability for each vertex of the simplex.

Figure S2. Performance of Delaunay Triangulation with number of dimensions

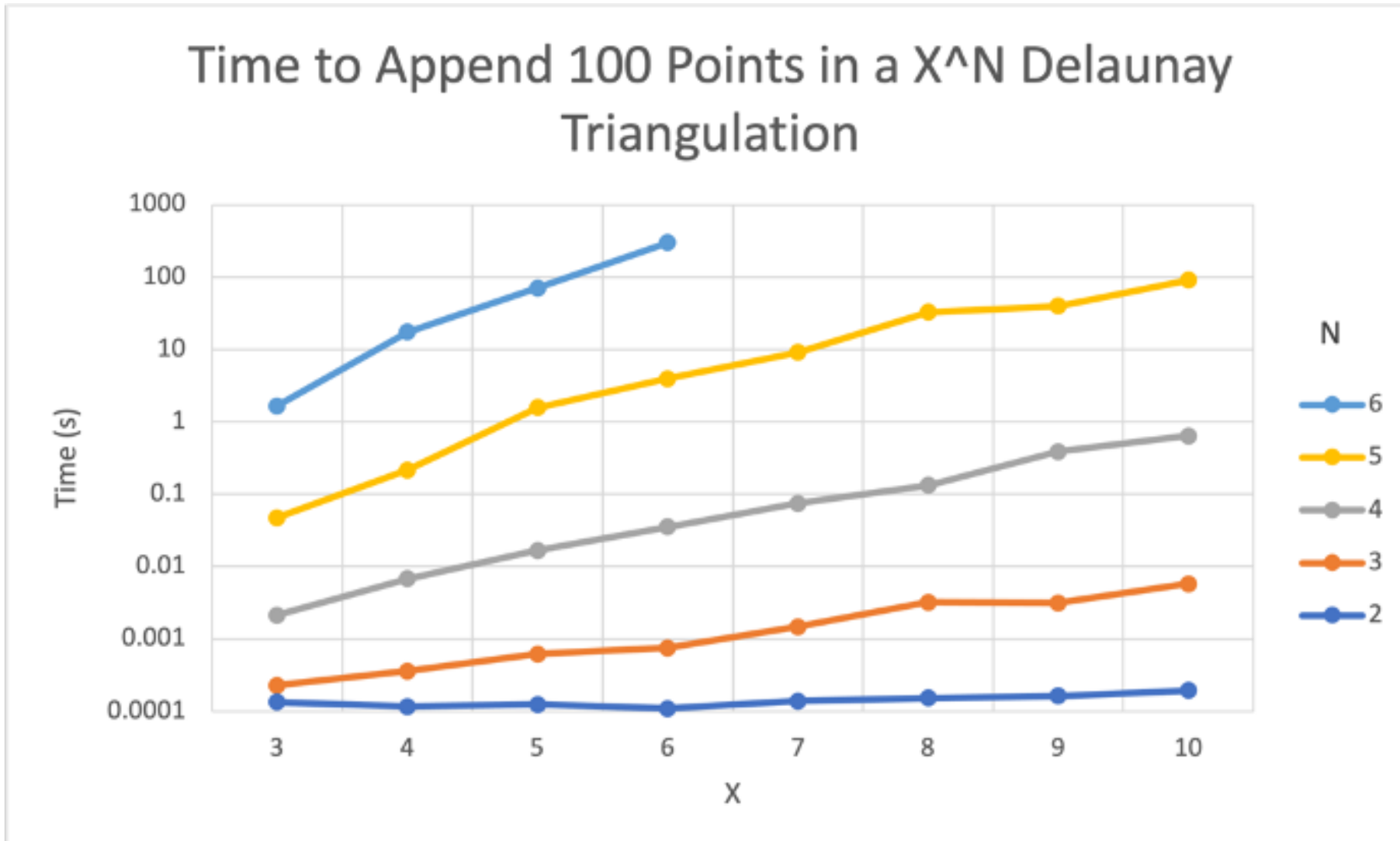

The time required to append 100 points into a Delaunay triangulation while varying the number of points in the set and the dimension of the points. X represents the size of the table, and N represents the dimension of the table. For the sixth dimension, the calculation was stopped when the size of the table reached 1,000,000 points. Time is in seconds, and the axis is plotted with logarithmic scaling.

Figure S3. Convergence of monomer sampling

A

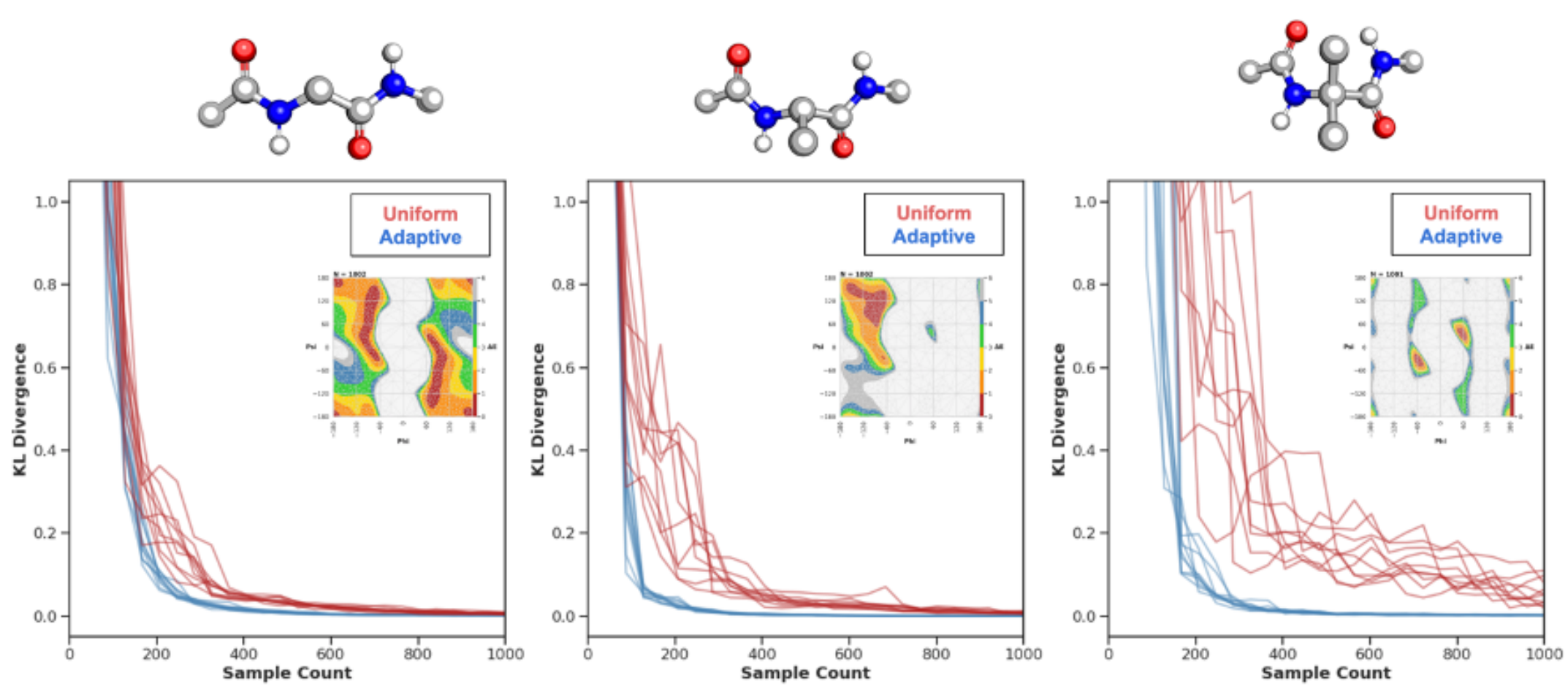

B

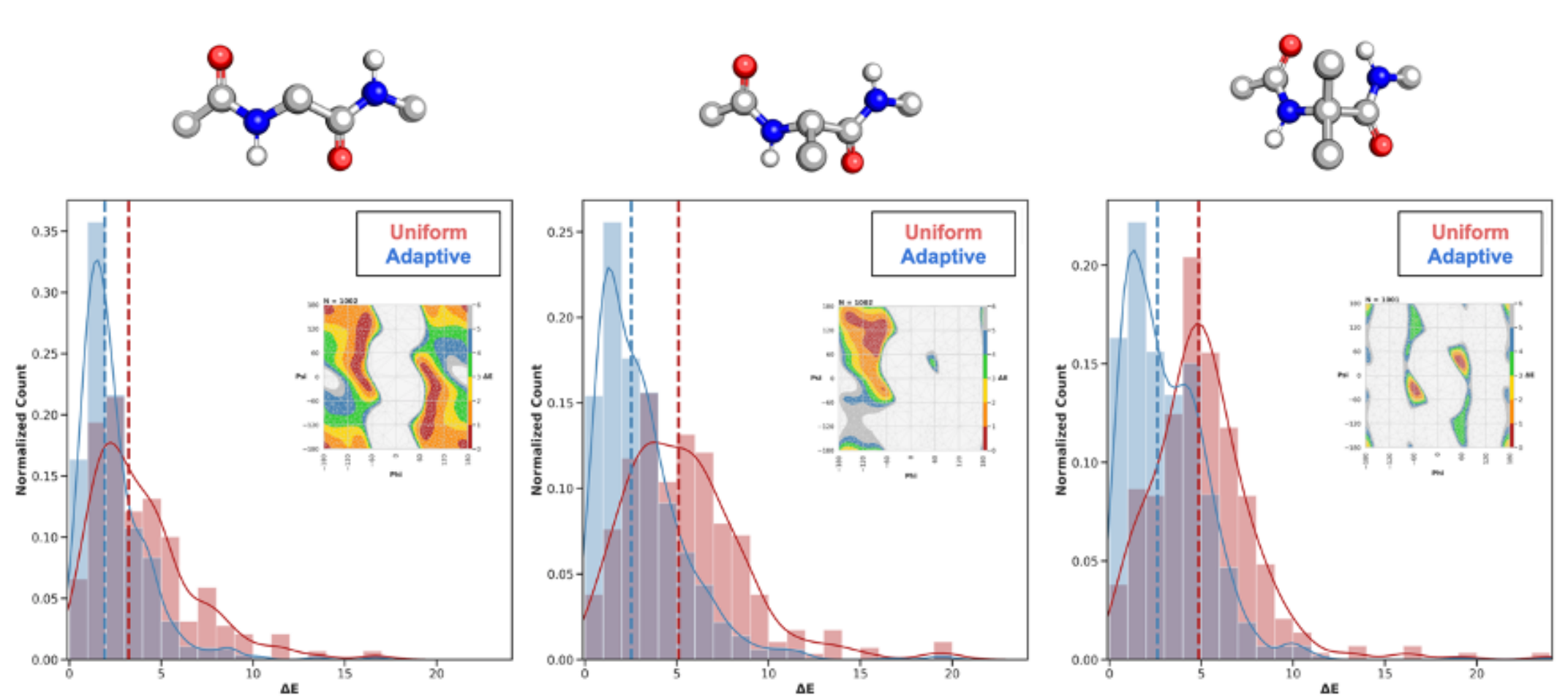

C

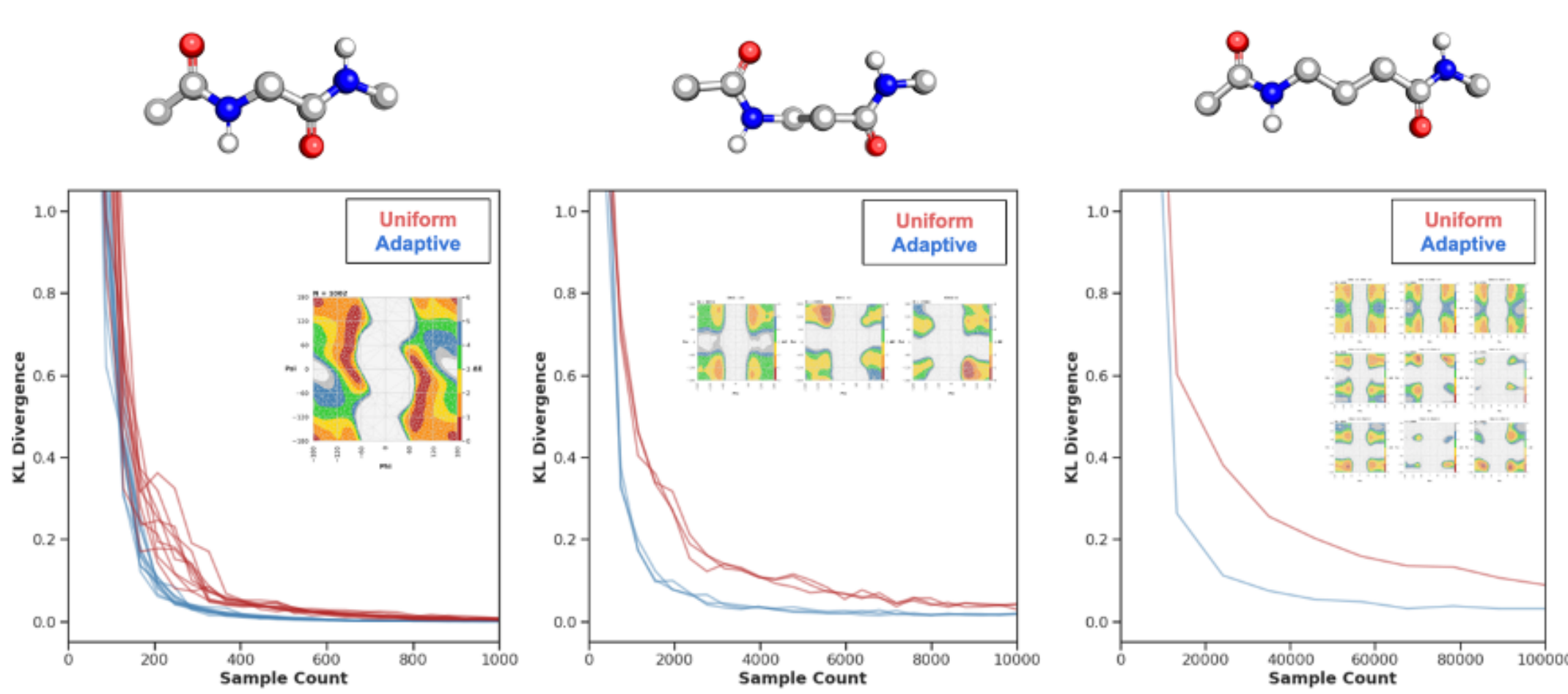

(A) A plot of Kullback-Leibler (KL) Divergence versus sample count for ten trajectories of conformer ensemble sampling with (blue) and without (red) adaptive sample for glycine (left), alanine (center), and α-aminoisobutyric acid (right). The divergence of the ensemble is calculated between checkpoints during sampling and the final exhaustive ensemble. The potential energy surfaces for each residue are shown as an insert within each respective plot. (B) A plot of the normalized distribution of points with respect to energy in kcal/mol after conformer ensemble sampling with (blue) and without (red) adaptive sampling for glycine (left), alanine (center), and α-aminoisobutyric acid (right). The vertical line depicts the median value of the energy from the distribution. The potential energy surfaces for each residue are shown as an insert within each respective plot. (C) A plot of Kullback-Leibler (KL) Divergence versus sample count for 10, 3, and 1 trajectories, respectively, of conformer ensemble sampling with (blue) and without (red) adaptive sample for glycine (left), β-glycine (center), and γ-glycine (right). The divergence of the ensemble is calculated between checkpoints during sampling and the final exhaustive ensemble. The potential energy surfaces for each residue are shown as an insert within each respective plot.

Figure S4. Distribution of properties of the monomeric residues

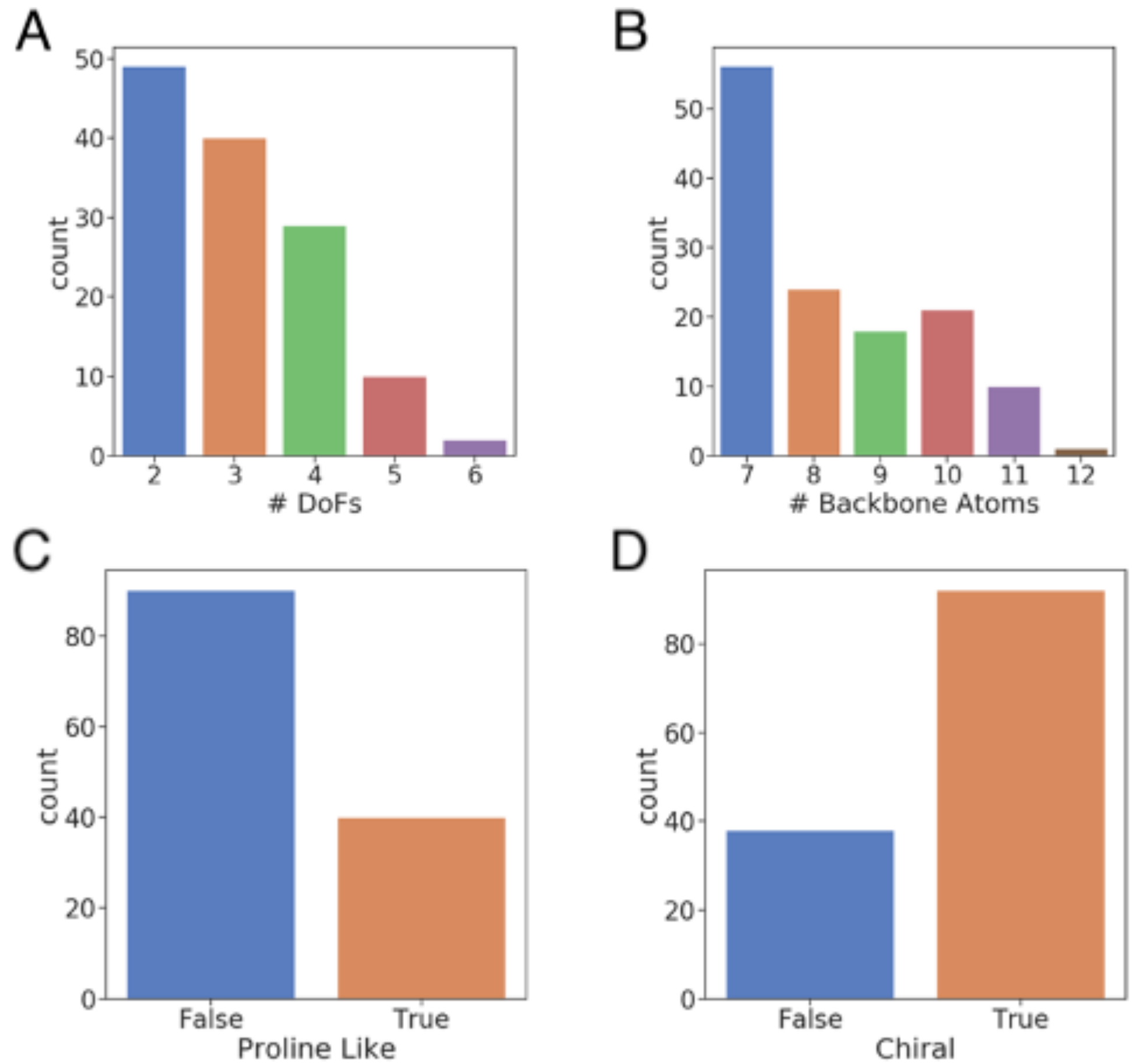

(A) The number of residues given the number of major degrees of freedom in the backbone and sidechains. (B) The number of residues given the number of atoms in the backbone. (C) The number of residues that are proline-like, containing a secondary amine. (D) The number of residues that contain a chiral center.

Figure S5. Comparison of computational predictions to known helices.

Validation of design methodology by comparison of low energy design models with previously described abiotic secondary structures with reported crystal structures.

In order to compare our design models to previously identified DPRs, we searched the literature and the CSD crystallography database. In many cases, we purposefully exclude some examples from the literature for the following reasons:
1) the chemical functional groups differ from the calculated molecules in a critical way,
2) the length of the crystallized peptide is insufficient for conclusively determining the presence of subunit repeats with helical symmetry, and
3) the crystal structure does not exhibit helical symmetry within a dipeptide subunit.

We've focused our comparison on a diverse set of backbone chemistries and discussed some successes and limitations of our method.

Summary Table:

	Type	Design seq	CSD ID	Description	Reference
A)	α/α	Ala/Ala		3_{10} helix	
B)	α/α	Aib/L-Pro	1227627	β bend ribbon	Di Blasio et al.[1]
C)	α/β	Aib/ACPC	640724	11-helix	Choi et al.[2]
D)	α/γ	Aib/γ^4(R)Ala	881177	12-helix	Basuroy, et al.[3]
E)	α/β	Ala/β2Ala		18/16-helix	Yoon, et al.[4]
F)	γ/γ	Quin/Pyri	666084	Herringbone	Delsuc, et al.[5]
G)	α/β	BEN2/Pro	687326	Aromatic	Prabhakaran, et al.[6]

A) α/α Poly-Alanine Helix Example

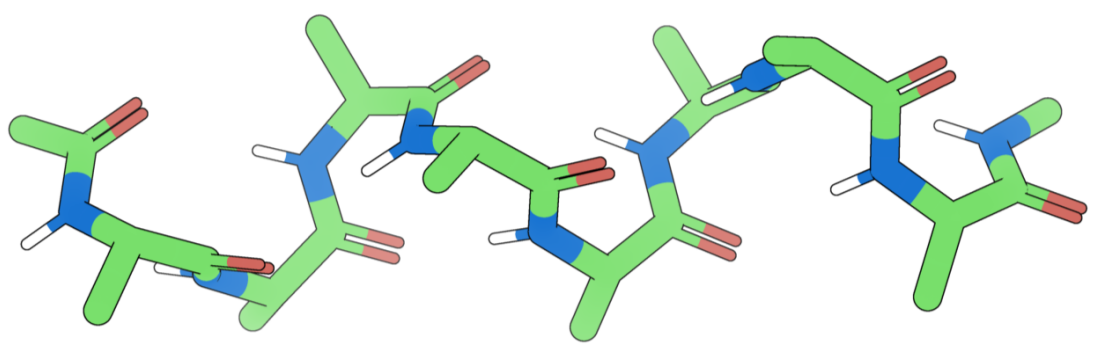

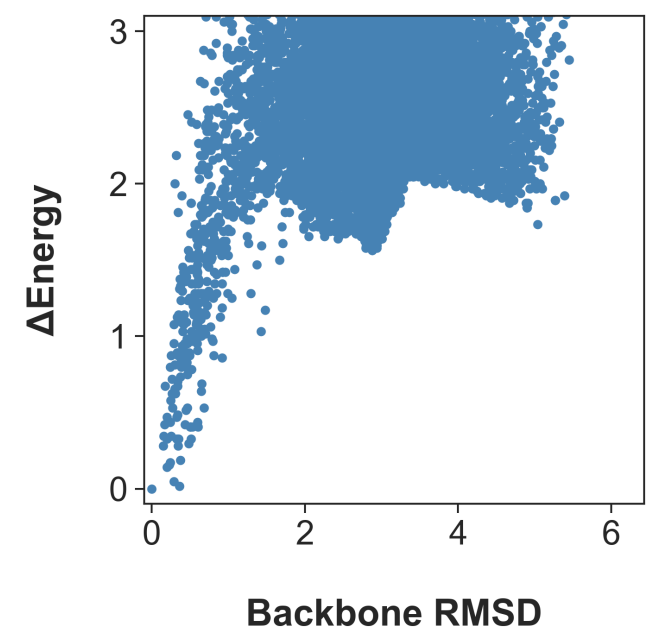

All combinations of the residues were calculated, which included dipeptides composed of identical residues as well. Therefore, we were able to retrospectively evaluate the calculations for an α L-amino acid helix with the Ala-Ala DPR. While the structure of L-α amino acid helices is well documented in proteins and long peptides, the structures of short helices in proteins and peptide fragments has been shown to have a bias toward the 3_{10} helix.[7]
Interestingly, our study unambiguously identified the 3_{10} as the predicted lowest energy structure with a phi of -67/-62 and psi of -22/-21 (the two monomers of the DPR had slightly different conformations). The closest confirmation to an ideal α helix was sampled at phi -57/-57 and psi -49/-49. Visually this conformer formed the *i*, *i*+4 hydrogen bond pattern, and it had a delta energy of 1.17 kcal/mol/residue and 1.48 Å RMSD to the lowest energy structure.

There are several potential reasons that our method identifies the 3_{10} helix:
1) there is a helical symmetric constraint during sampling, and the α helix may require slight irregularities along the helix for stabilization which we would fail to identify
2) neural network score function was trained on small molecules and peptide fragments; the contributions of long range interactions could be undertuned (in the monomer energy landscape of ALA [Figure 1C], the phi/psi of the 3_{10} is preferred over the α helix),
3) Experimental studies and DFT calculations have shown that the 3_{10} helix is preferred in gas/vacuum and the α helix is preferred with high dielectrics; the solvation that was trained into the AIMNet(SMD) model may be less accurate with large molecules outside of the training distribution.[8,9]
4) Vibrational mode distribution affects the entropic contributions to helical stability; however, they were not considered in this study.[10]

B) α/α DPR Helix Example

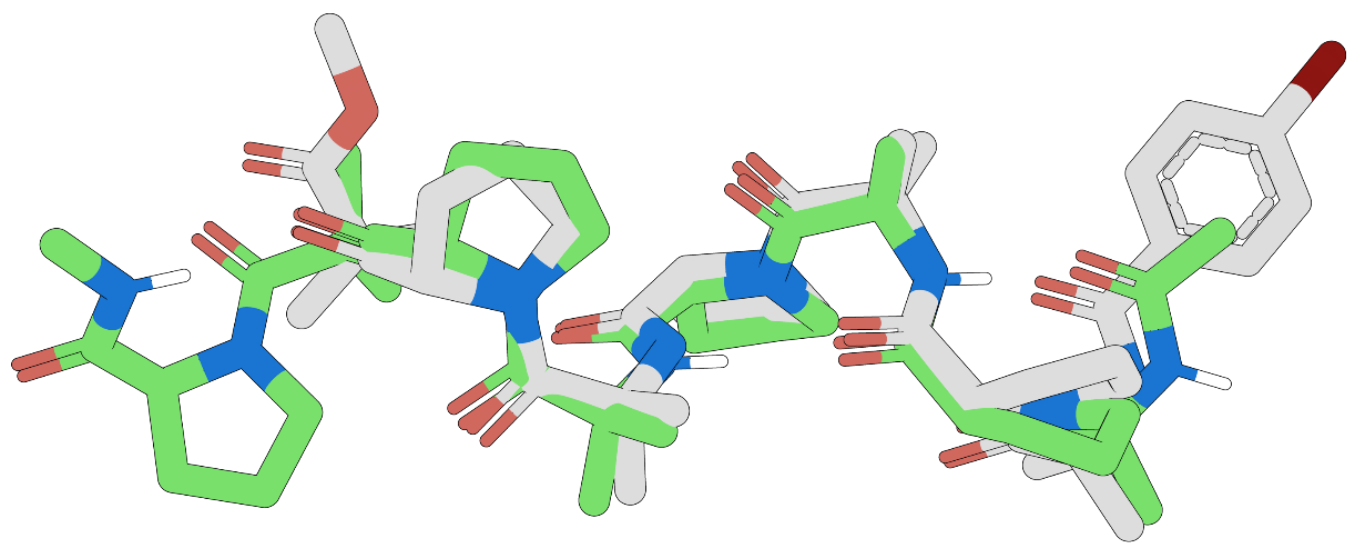

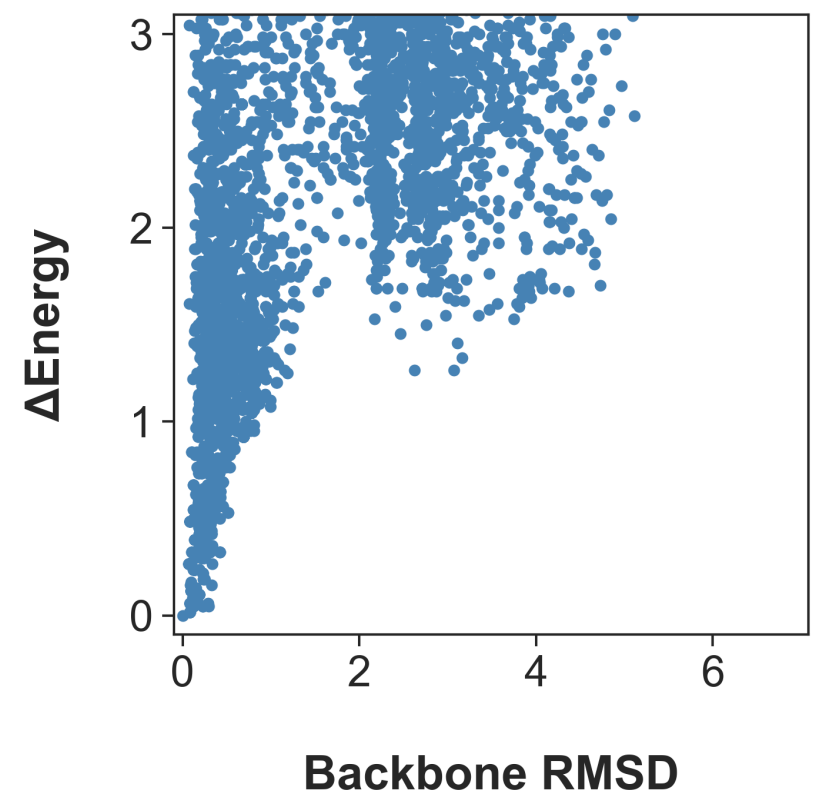

A representative example of an α/α X-ray crystal structure (gray) with a repeating dipeptide unit of α-aminoisobutyric acid (Aib) and proline and the structure is described as a "β-bend ribbon spiral", characterized by intramolecular backbone *i*, *i*+3 hydrogen bonds.[1] This aligns well with our predicted lowest energy model with dihedral angles close to a type I' β turn or a 3_{10} helix (repeating type III' β turn). This structure has one of the largest delta energies observed in our screening. The crystal structure was obtained from the CSD (Reference code 1227627).

C) α/β DPR Helix Example

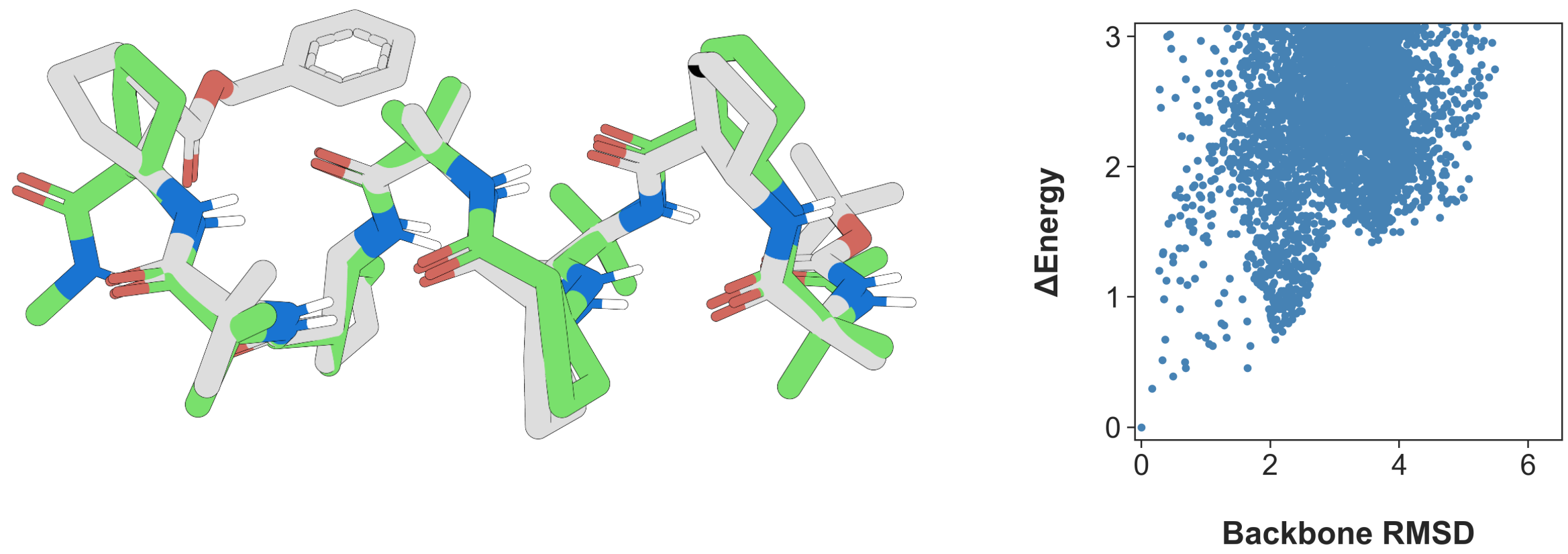

An example of a predicted α/β secondary structure (green) with the corresponding crystal structure (gray) from the work of Choi et al. and the Gellman lab.[2] They found that at shorter lengths the 11-helix is stabilized in the context of (*S,S*)- *trans*-1,2-ACPC and α-aminoisobutyric acid (Aib). Our predictions also find that the 11 helix is the lowest energy state. However, the ensemble for this example has a relatively small energy gap as well as relatively poor sampling density compared to the alternative state. These computational indications of weak folding could hint at why the 11 helix and 14/15 helix stability are dependent on length. The crystal structure was obtained from the CSD (Reference code 640724).

D) α/γ DPR Helix Example

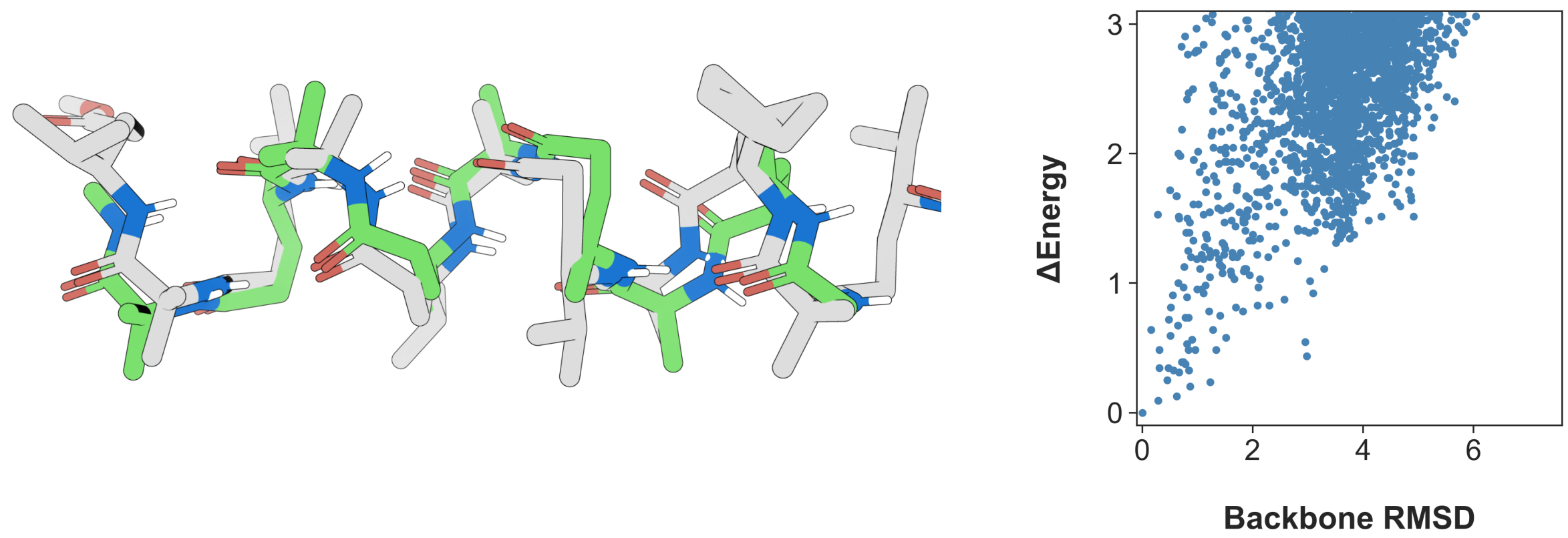

Basuroy et al. solved the crystal structure of a mixed α/γ polypeptide (gray) forming a *i*, *i*+3 hydrogen bonded C12-helix without residues with cyclic constraints in the backbone,[3] which is compared to our lowest energy design model (green). We did not explicitly model the β-branched variant of the γ^4 amino acid, which could account for the alternative low energy states at higher RMSD (~3 Å). Those points correspond to a β-turn like structure with an unsatisfied backbone NH exposed to solvent. The crystal structure was obtained from the CSD (Reference code 881177).

Both examples C and D from this section highlight that the selection of the energy cuttoff threshold is not obvious. While the lowest energy state was correctly identified for both molecules, the funnels are quite broad in RMSD and the energy gap is narrow.

In the context of our study, the false positive and false negative rate is dependent on the threshold. By tightening the threshold, the false positive rate will increase, but conversely, the false negative rate will decrease. For molecular design, a low false positive rate is preferred because it reduces the number of costly experiments. Practically, this means using a stricter threshold to ensure only the best predictions are considered.

There is a limit to tighten the threshold. Score functions are not perfect, so it has been seen in drug discovery that the best tip of the distribution is enriched for molecules that exploit errors in the score function.[11] The ideal threshold would be selected by maximizing the average precision, but that requires many positive examples and negative examples.

E) α/β DPR Helix Example

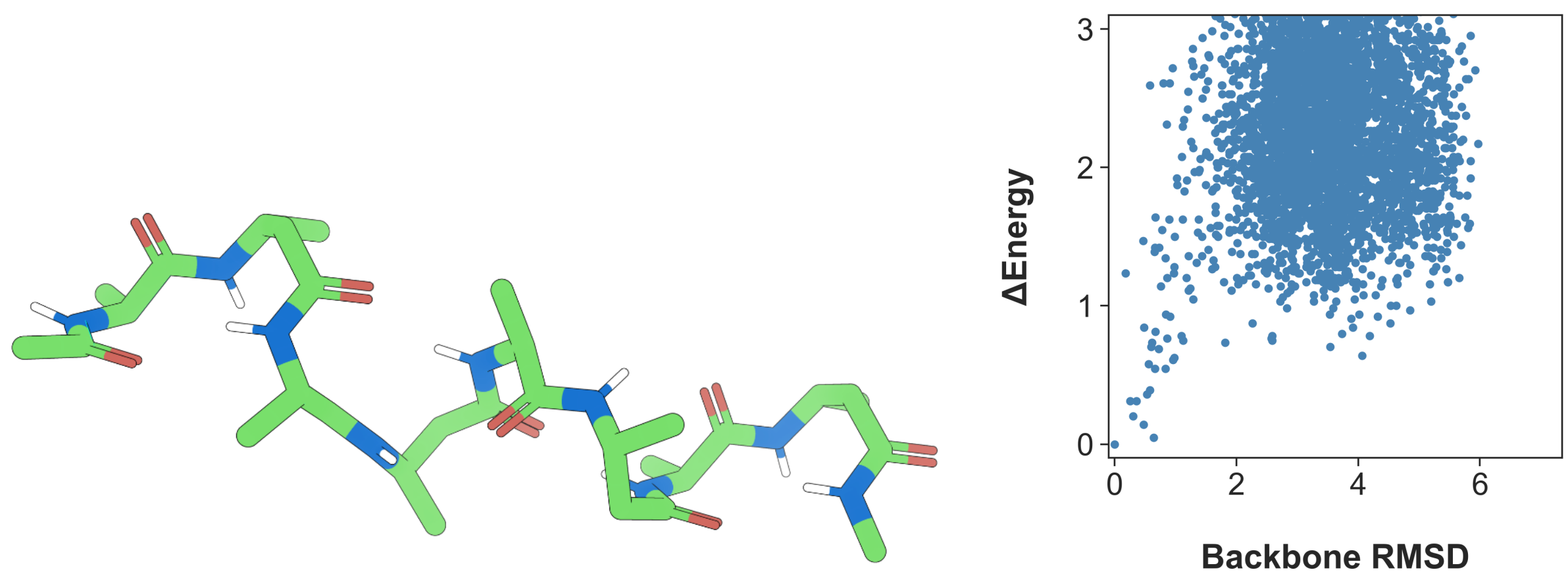

DPR5 is an α/β DPR with repeats of ALA and (*S*)-β^2-homoalanine. The lowest energy calculated conformation of DPR5 forms a mixed helix with backbone hydrogen bonds facing both directions and matches the 18/16 backbone hydrogen bonded conformation previously computed by Baldauf, et al.[12] However, it does not match the recently described 11/9 helical structure for the short 3x DPR5 that was supported by NMR data.[4] It has been observed that these two types of helices can easily interconvert with a single backbone structural rearrangement, specifically the rotation of the φ angle of the α residue by about 90°.[13] Both structures have stabilizing apolar methyl-methyl contacts, showing no clear preference for one over the other. The low signal for both experimental and predicted CD spectra we obtained for DPR5 led to its exclusion from further experimental analysis (SI Fig. S6); although signal remained low, it was found to increase when using n-propanol solvent.[4] This suggests that the interconversion may be influenced by length and solvent, similar to the 3_{10} to α-helix equilibriums.[14] It is possible that the 18/16 helix structure observed in our calculations will be favored over the 11/9 helix in the case of longer DPR5 polypeptides.

F) Herringbone DPR Helix Example

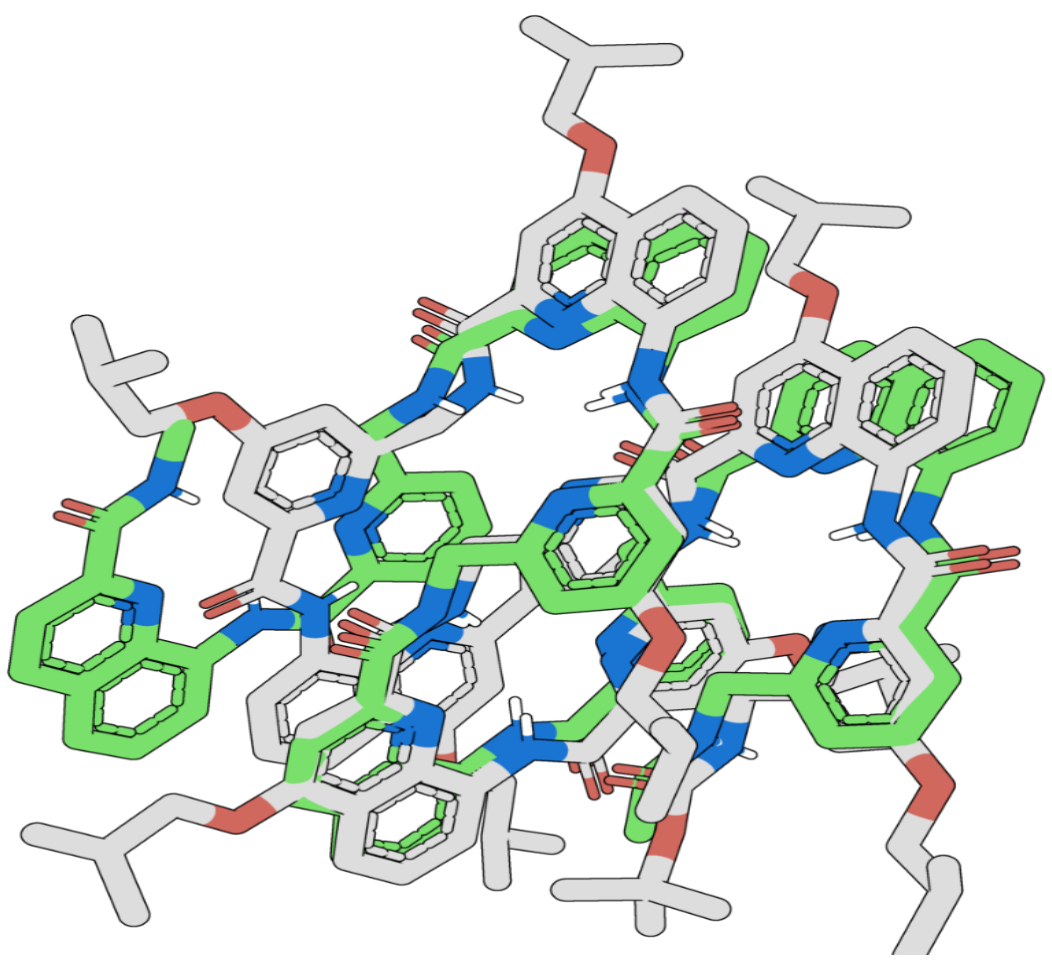

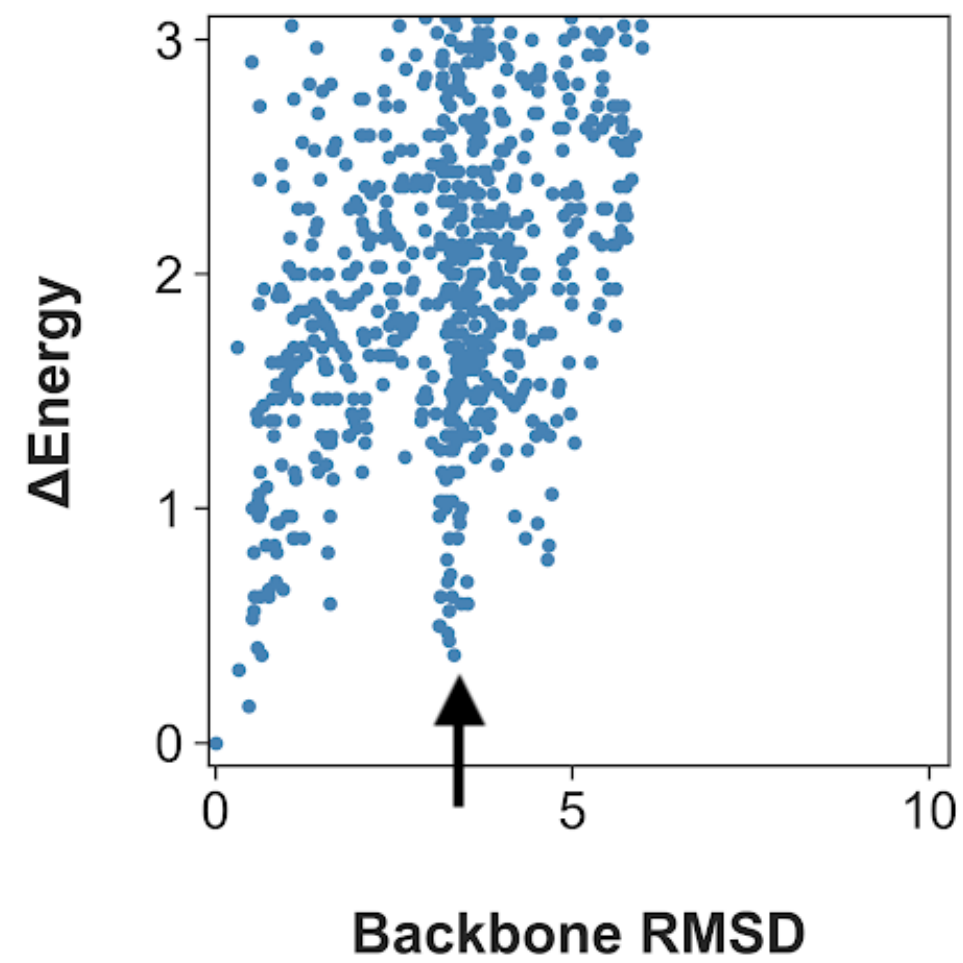

Looking forward to using this protocol for designing organic electronics, evaluating our prediction against the experimental work of Delsuc et al. in the Huc lab[5] was of significant interest. Here we compare the crystal structure of the achiral herringbone helix (gray) made up of similar quinoline and pyridine derivative γ/γ amino acids to the lowest energy structure (green). Coincidentally, the same chirality of the helix was computationally found to be the lowest energy structure, but the opposite chirality was also found at 3.5 Å RMSD, which is shown in the energy landscape at +0.4 kcal/mol/residue. From the report of Delsuc et al., this odd helix was not expected at the onset of their work, but it is an example of how computation can go beyond the intuition of humans. The crystal structure was obtained from the CSD (Reference code 666084).

G) Aromatic-backbone DPR Helix Example

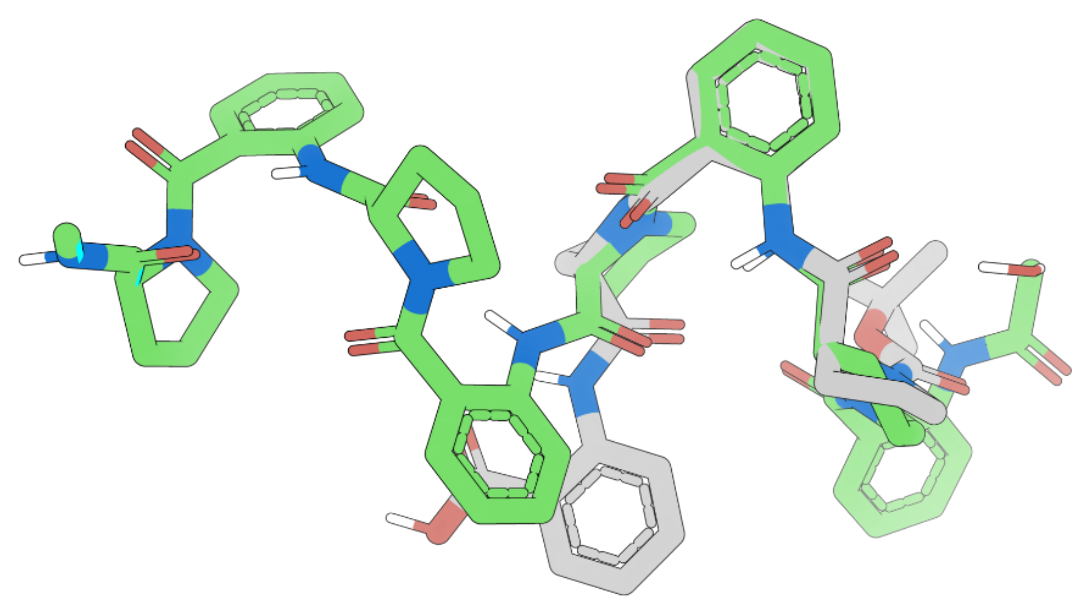

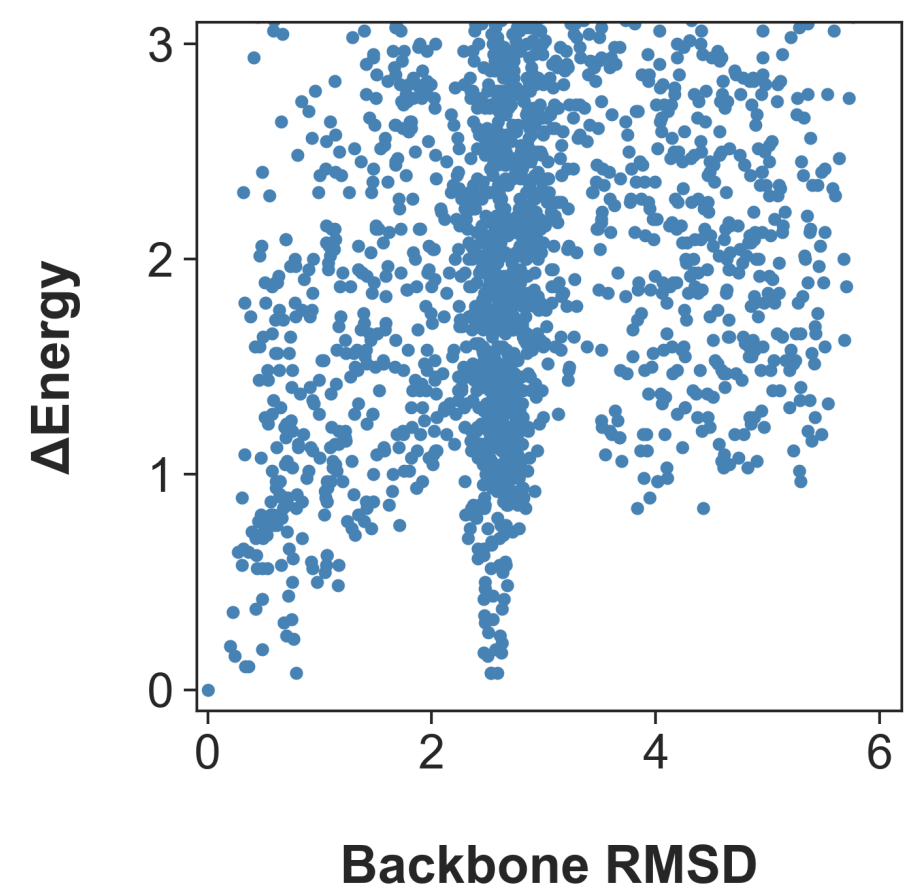

Second low energy state:

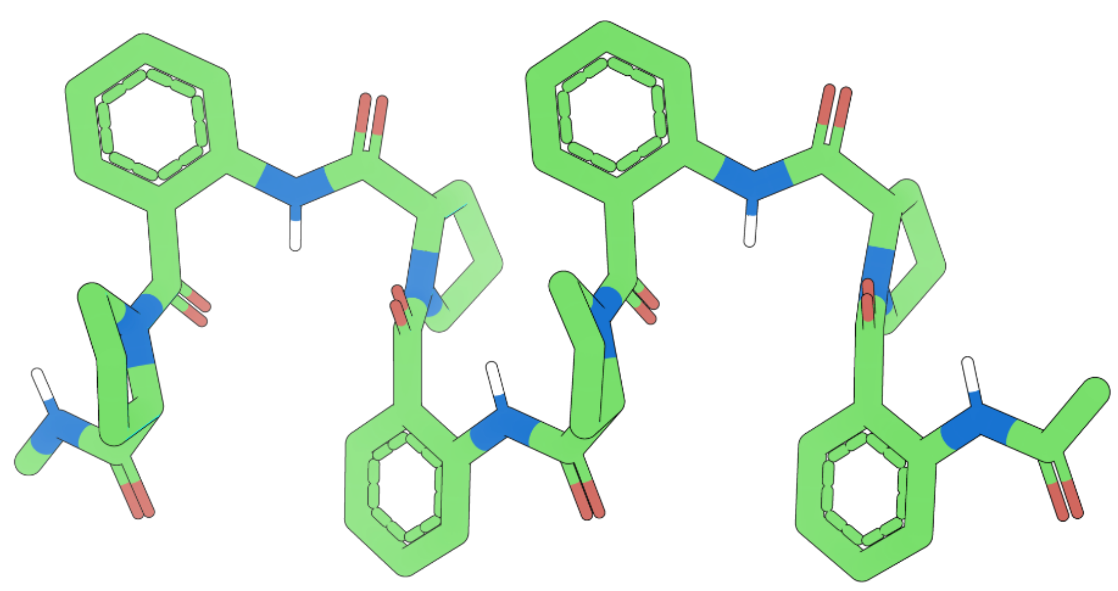

The BEN2/PRO prediction is an example of a false negative in comparison given the literature precedence. Our funnel identifies two distinct low energy states given this sequence. The lowest energy state matches the crystal structure of the 2x BEN2/PRO molecule (Reference code 687326).[6] There is a slight deviation from the design model at the termini of the crystal structure, which could be attributed to crystal packing. Ideally, we would compare at least a 3x version of the crystal structure to evaluate the middle of the helix.

Also, there is a very small gap in energy compared to the second low energy state shown above. Our predictions would suggest that these two conformations would be in thermal equilibrium as thermal energy at 298 K is 0.59 kcal/mol which is sig higher than the predicted energy gap. Interestingly, this second low energy state was observed in other crystal structures of the same study when the proline was replaced with AIB. This highlights the difficult task of accurately comparing the energetics of reasonable low energy structures.

Figure S6. Helix schematics for DPR1-DPR10 models

(A) Schematic diagram of DPR1 - DPR4 indicating hydrogen bonds and dihedral angles rounded to the nearest 10°. Some side chains that were not in hydrogen bonds have been removed for clarity. A head-on view of each helix is shown (right). (B) Schematics for DPR6 - DPR10.

A

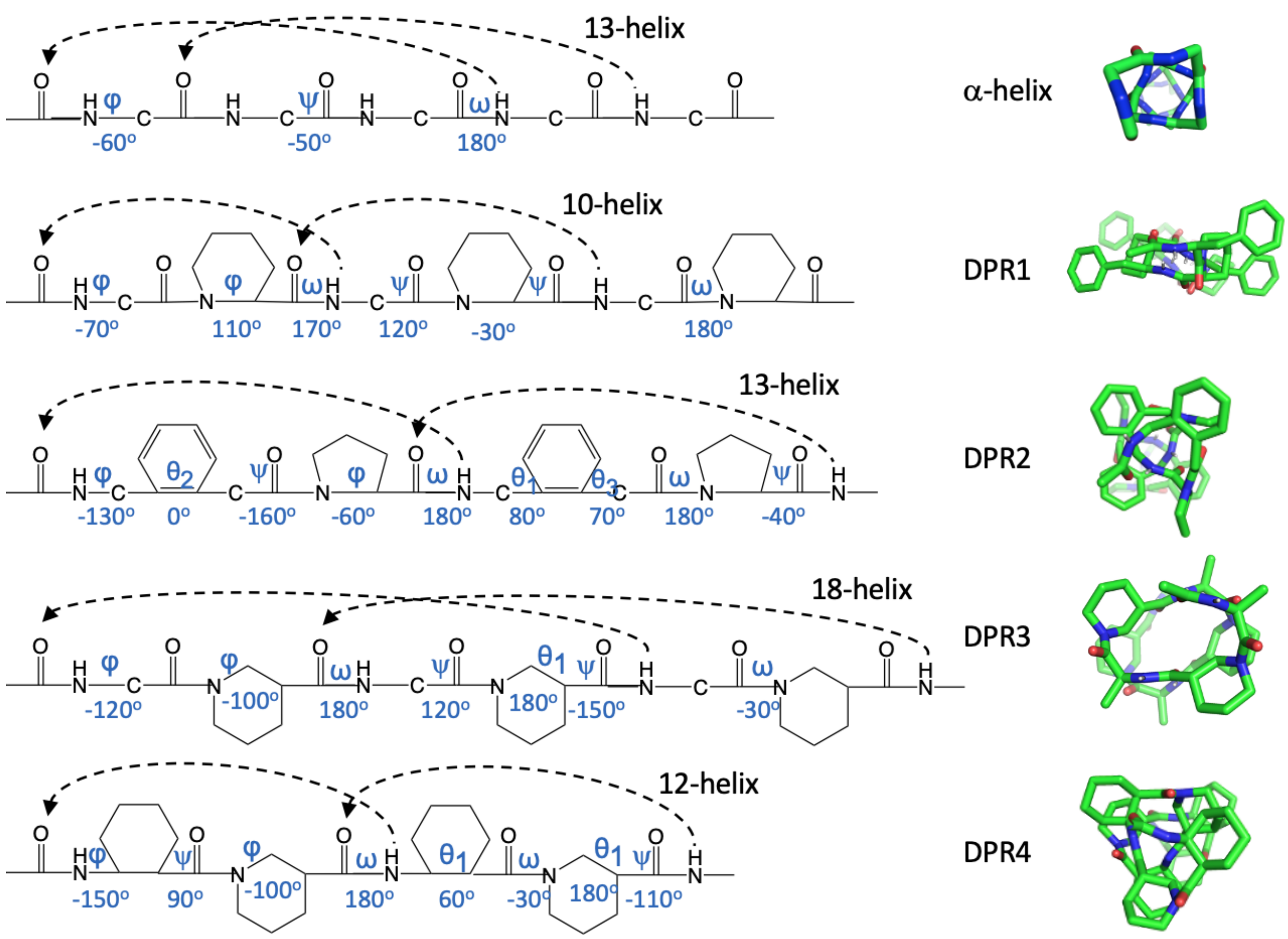

B

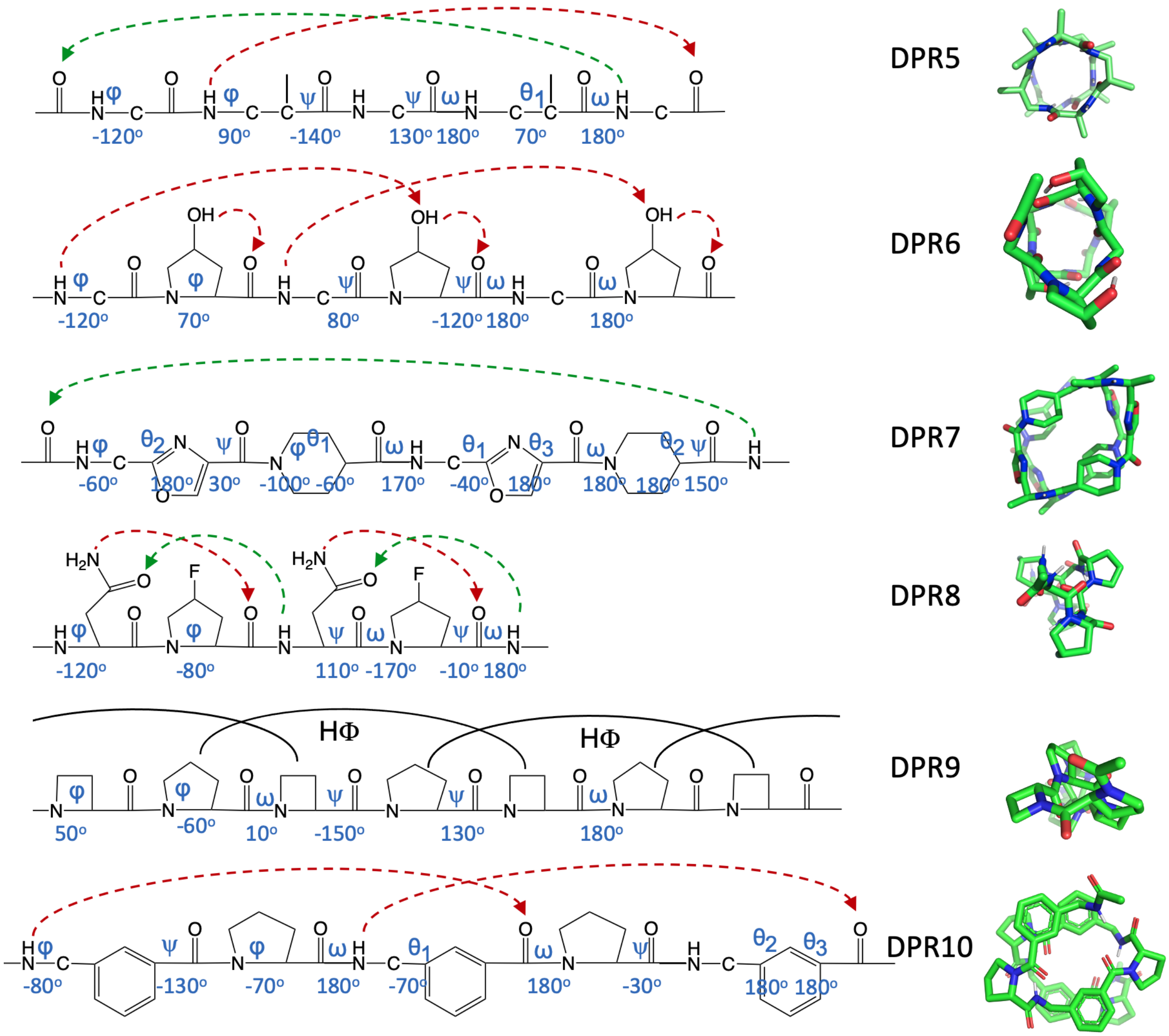

Figure S7. Experimental CD spectra of 10 DPRs

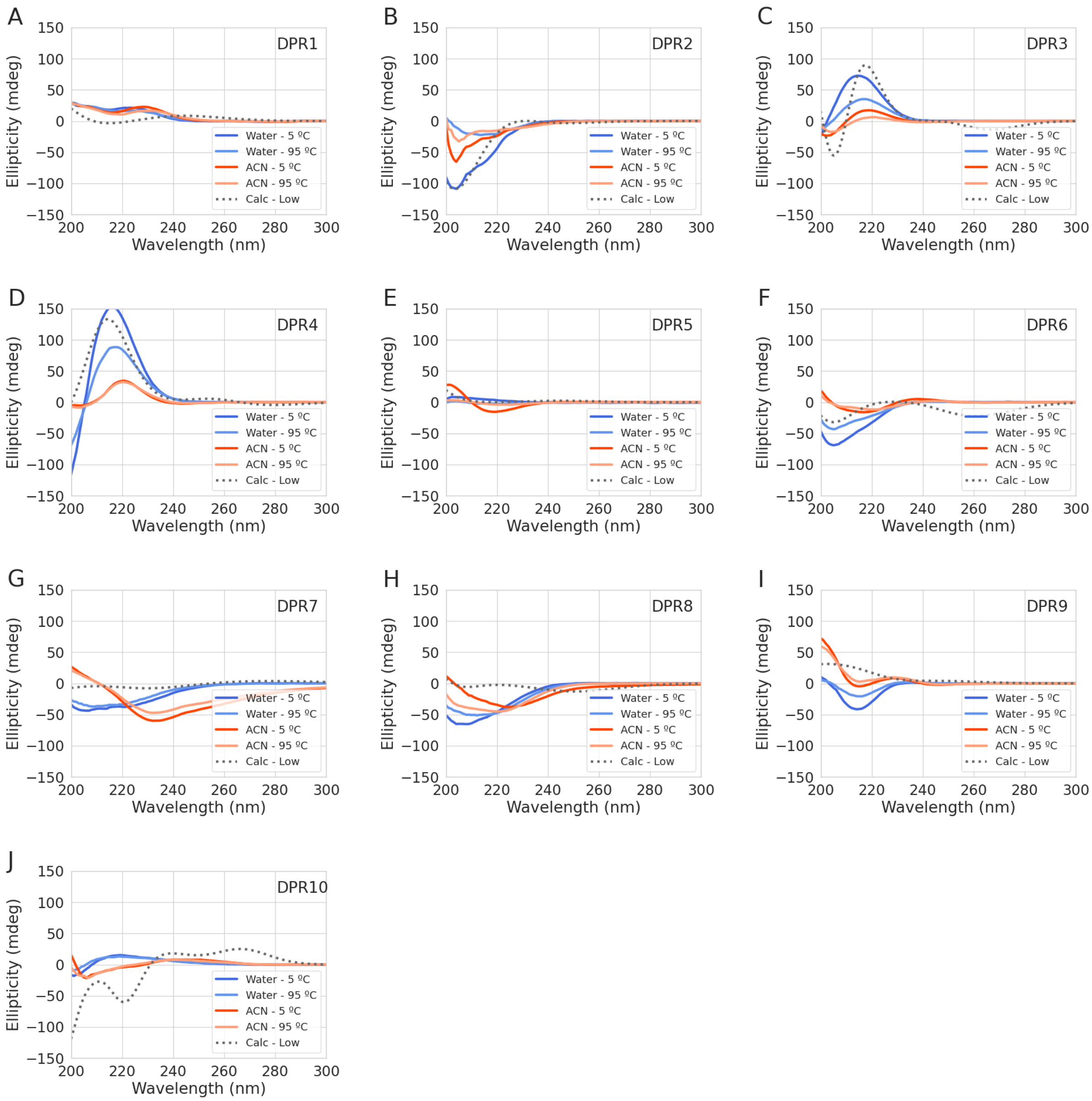

Experimental CD spectra for 10 DPRs in 100% H_2O (blue) or 100% ACN (red), at 5 ºC (light) or 95 ºC (dark), together with the average calculated theoretical spectra (dotted gray) for the low energy ensemble. The length of the polymers is 3x for DPR1, DPR2, DPR4, and DPR9; 3.5x for DPR10 (7 residues); and 4x for DPR3 and DPR5 - DPR 8. Higher CD intensities in the UV region, typically indicating increased secondary structural ordering,[15] were observed for seven DPRs (DPR2 - DPR4, DPR6 - DPR9). Six DPRs (DPR2 - DPR6, DPR9) displayed significant temperature-dependent intensity changes. DPR2-DPR4 best matched the calculated spectra from the helical design models, which take into account contributions from complex structural factors. DPR1

had only a weak CD signal (both experimental and calculated) but was pursued further after the crystal structure was obtained. Subsequent near UV CD (Figure 3E) and NMR studies on 5x DPR1 gave support for ordered helical secondary structure in solution. Taken together, 8 of the DPRs have spectra that suggest some solvent-dependent and/or temperature-dependent ordered structure. DPRs 1 through 4 were selected for further structural analysis based on their agreement with the calculated CD spectra.

Figure S8. CD spectra calculated with different functionals or basis sets

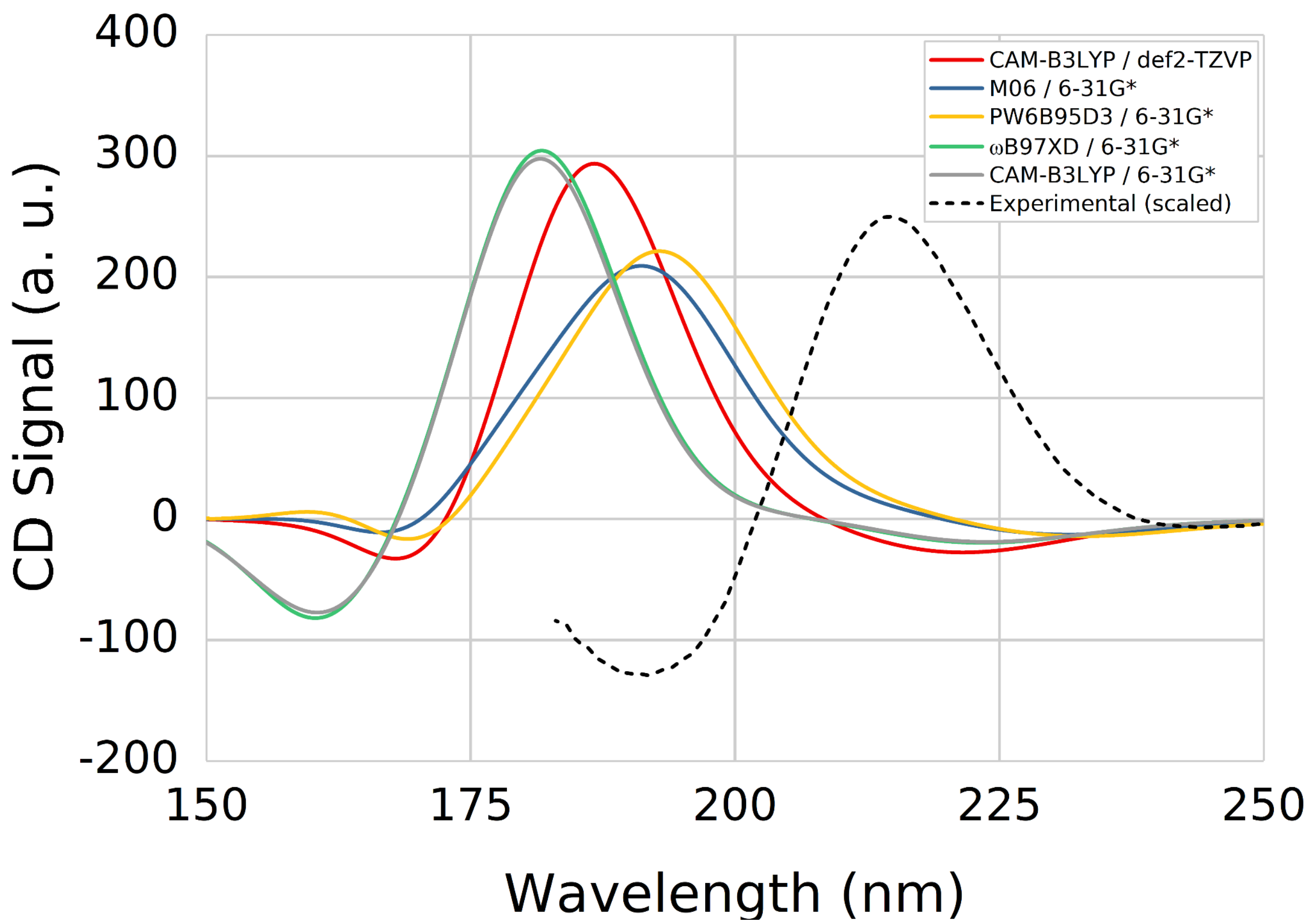

Theoretical circular dichroism spectra for a low-energy conformer of 4x DPR4, using various functionals and basis sets. For the M06 (blue) and PW6B95D3 (yellow) functionals, 96 excited states were included in the calculation, while for the rest, 32 states were sufficient. All calculations produced a qualitatively similar result; among these, CAM-B3LYP / 6-31G* (gray) provides a good compromise between accuracy and computational cost. The experimental spectrum for this peptide (Figure 3C), arbitrarily scaled (black dotted line), is shifted but retains a similar profile to the predicted spectra. Prediction of CD patterns for the DPR helices was necessary to compare with and interpret the complex experimental results. Spectral patterns include contributions from several types of overlapping transitions in the UV region. These include not only those related to the amide backbone and charge-transfer transitions between neighboring amide groups[16] but also contributions from the aromatic side chains[17] influenced by their electronic coupling,[18] and from tertiary structure effects.[19]

Figure S9. Calculated CD spectra for low- and high-energy ensembles

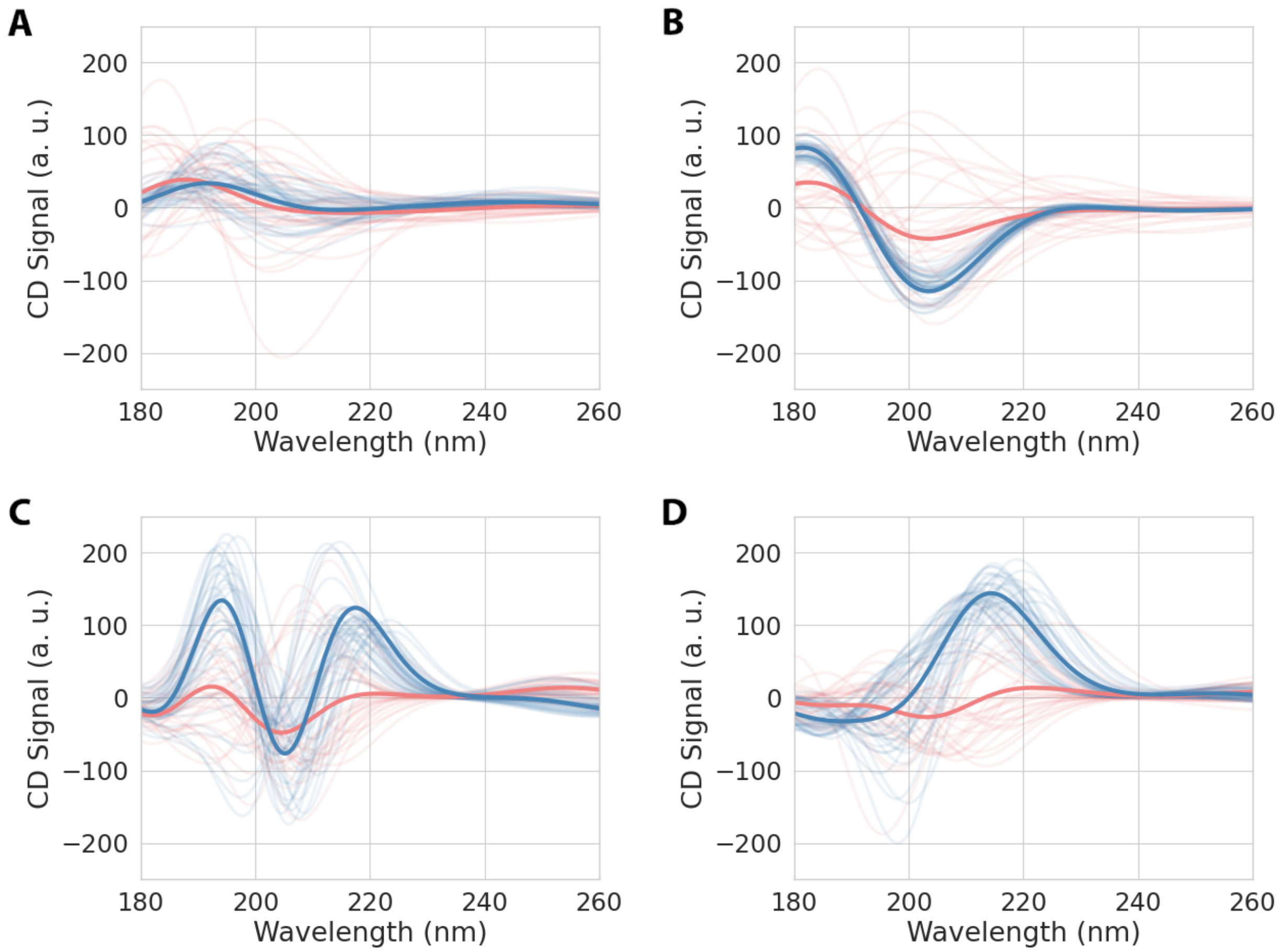

(A-D) Calculated circular dichroism spectra (thin lines) for 40 low-energy (blue) and 30 high-energy (red) conformers of 4x polymers. Thick lines correspond to the low-energy (blue) and high-energy (red) averaged spectra for each set of calculated spectra. The spectra correspond to (A) DPR1, (B) DPR2, (C) DPR3, and (D) DPR4.

Figure S10. Calculated CD spectra for different polymer lengths

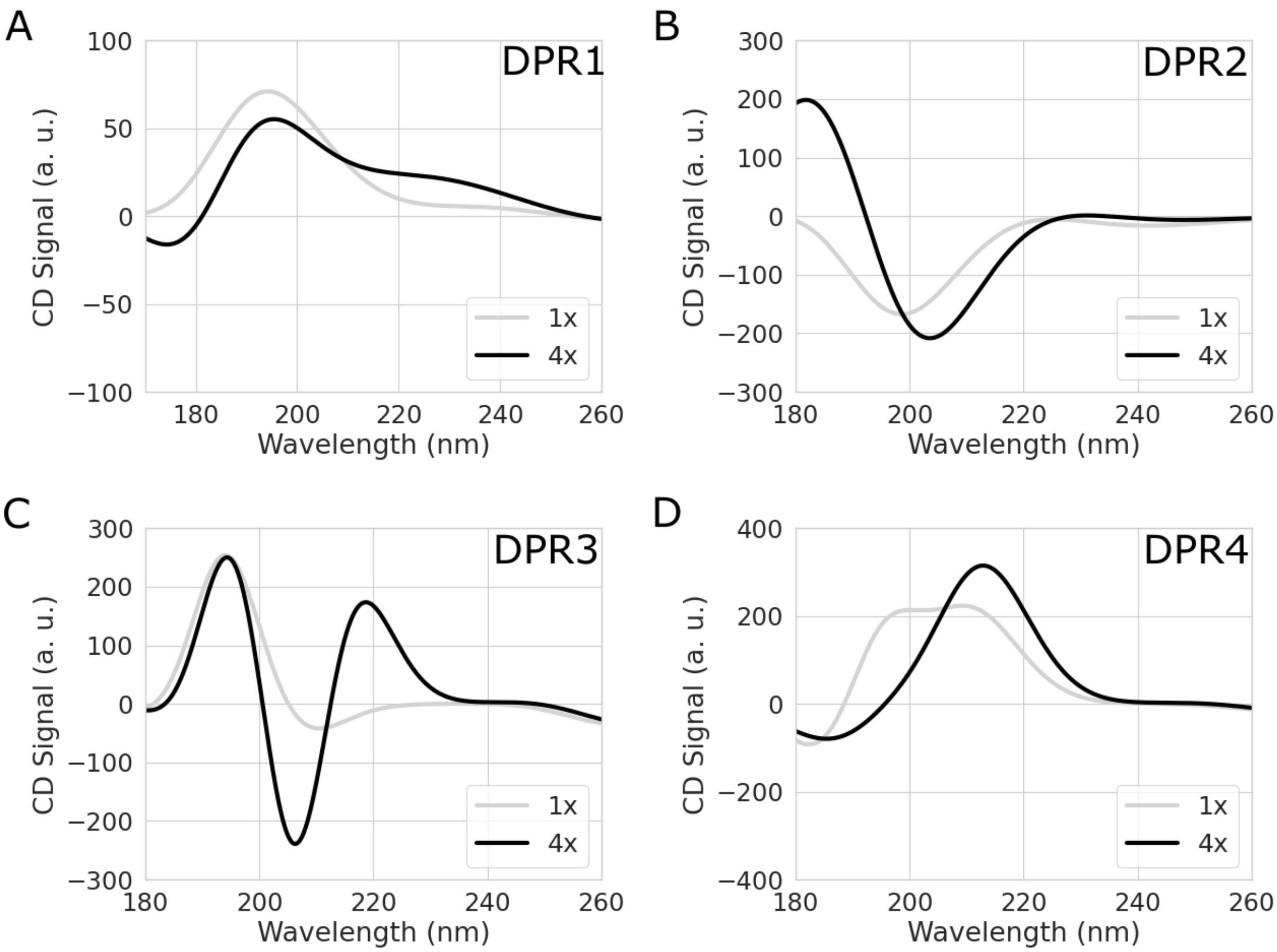

(A-D) Calculated circular dichroism spectra for single low-energy conformers of 1x or 4x polymers. The spectra correspond to (A) DPR1, (B) DPR2, (C) DPR3, and (D) DPR4. While the 1x models mainly probe local contributions to CD spectra, the 4x models also include long-range contributions. Thus, the difference between the 1x and 4x spectra can be ascribed to the secondary structure contributions. The 4x spectra are, in most cases, significantly different from those for the 1x polymers, particularly around 210 - 220 nm, where they show higher intensities. Therefore, CD can be used to assess secondary structuring in solution for these polymers. Notably, the calculated differences between 1x and 4x polymers follow similar patterns to those of the experimental spectra (*cf.* 1x and 10x spectra in Figure 3), providing additional confirmation that the structural models are representative of the actual polymers.

Figure S11. DPR1 1D ^{1}H spectra and ^{1}H-^{15}N HSQCs

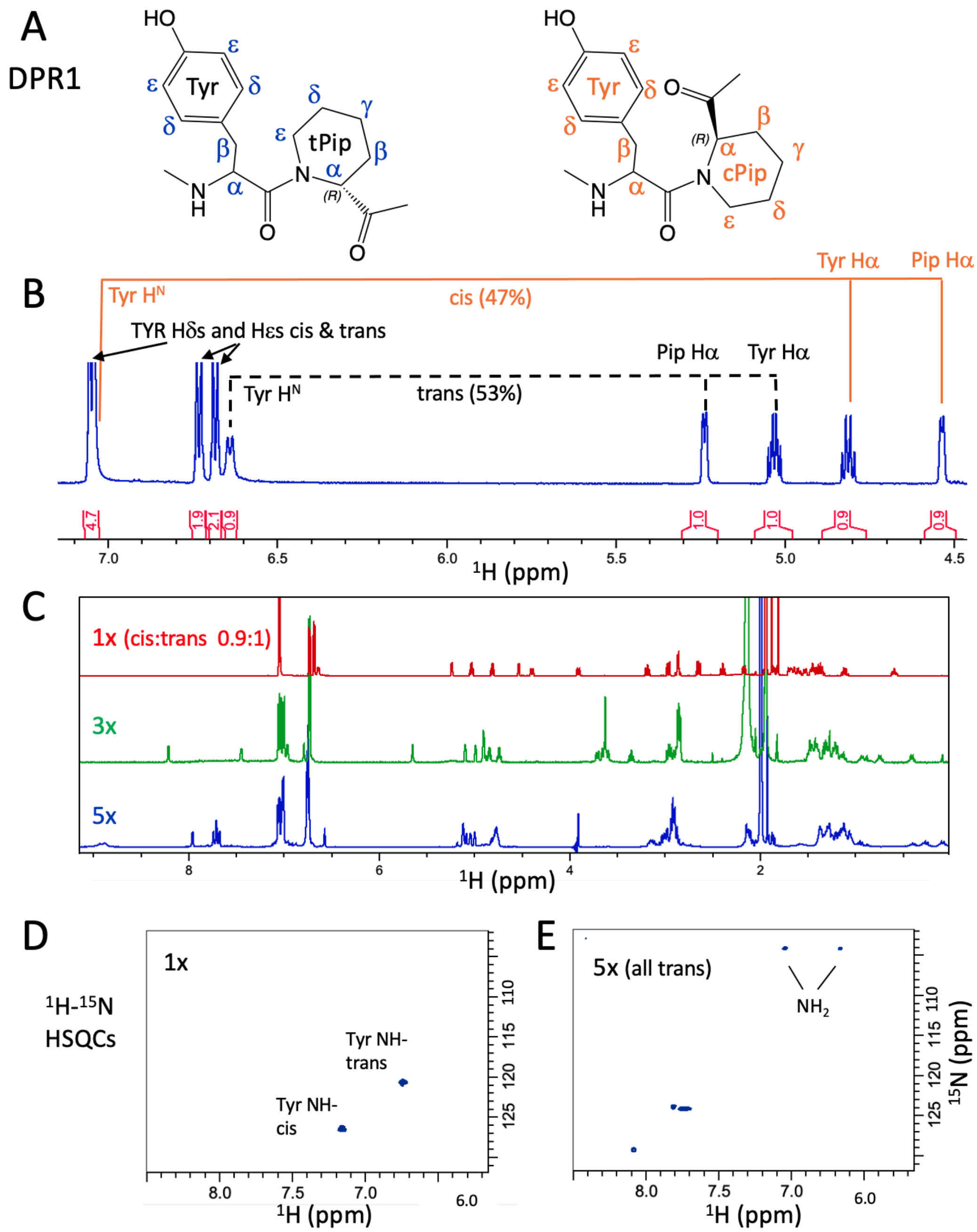

(A) Chemical structures of *trans* Tyr-tPip and *cis* Tyr-cPip. (B) 1D ^{1}H NMR spectra of 1x DPR1 in CD_3CN at 298 K with integrations of 0.9:1 of cis : trans Tyr-Pip peptide bond conformations. (C) 1D ^{1}H spectra of 1x, 3x, and 5x DPR1 at 283 K. (D-E) 2D ^{1}H-^{15}N HSQC spectra of 1x (D) and 5x (E) DPR1. The 5x DPR1 exclusively showed the *trans* D-Pip conformation, with downfield chemical shifts of H^N resonances indicating increased intramolecular hydrogen bonding propensity.

Figure S12. DPR1 1x 2D ^{1}H-^{13}C HSQC and Pip chemical shift assignments

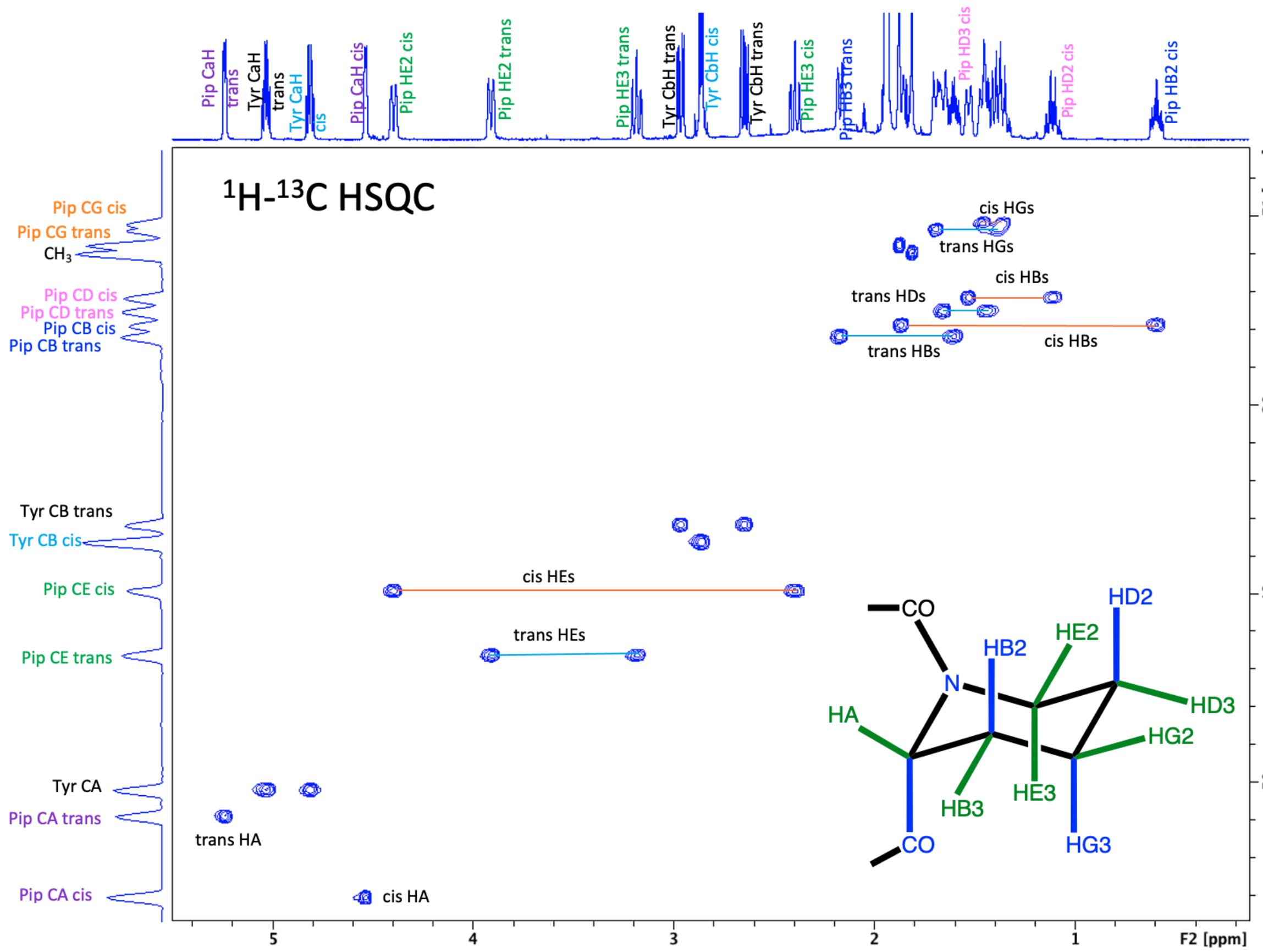

2D ^{1}H-^{13}C HSQC spectra of 1x DPR1 in CD_3CN at 298 K with X-pipecolic acid *cis* and *trans* side chain peaks labeled. Piperidine ring (inset) with stereospecific labels: blue lines are axial, and green are equatorial. Chemical shift assignments for both the cis and trans conformers were determined (Table S2) using standard 2D COSY, TOCSY, and NOESY experiments and confirmed by observation of characteristic NOEs. The cis conformation had a strong NOE between the Hα of Pip and the Hα of the preceding Tyr. The trans had strong NOEs between the Hεs of Pip and the Hα of the preceding Tyr. Stereospecific assignments for all D-Pip hydrogens were determined, establishing its chair conformation with axial CO placement in the trans conformation. The 5x data (not shown) was consistent with this conformation as well and matches with the design model.

Figure S13. DPR1 1x and 5x chemical shifts change with temperature

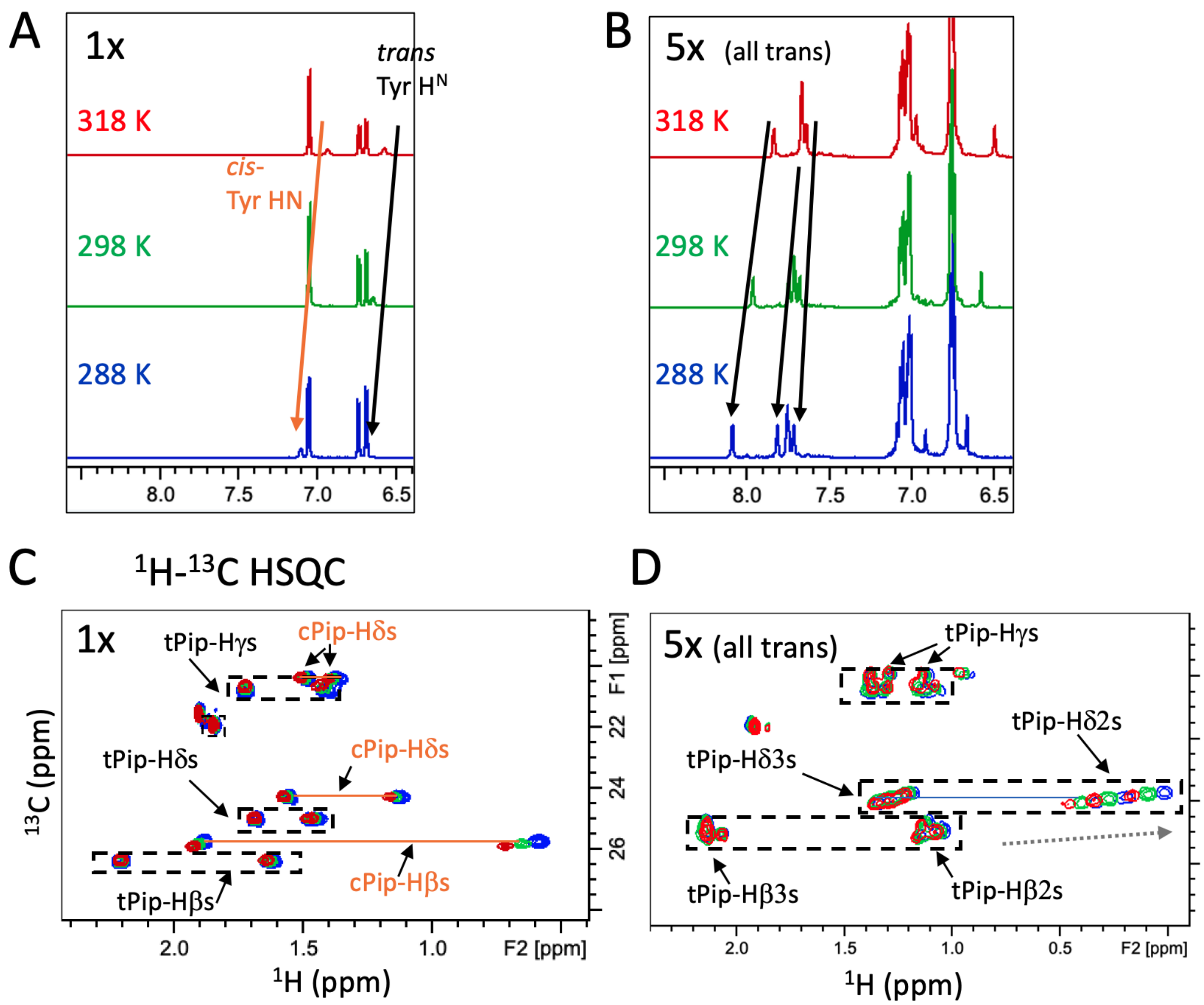

(A-B) 1D ^{1}H spectra of 1x (A) and 5x (B) DPR1 at three temperatures (288, 298, and 318 K) reveal downfield-shifted H^N resonances at lower temperatures. (C-D) 2D ^{1}H-^{13}C HSQC spectra of DPR1 (C) 1x and (D) 5x vs. temperature. Selected trans Pip, tPip, peaks that showed temperature-dependent shifts are marked with dashed boxes, and the corresponding cis, cPip, peaks in the 1x are labeled in orange. The 5x spectra have only trans X-Pip peaks due to stabilization of the trans peptide bond conformations by secondary structure formation. The largest temperature-dependent ring current shifts were observed for tPip Hδ2s.

Figure S14. DPR1 5x NOESY data at 298 and 283 K

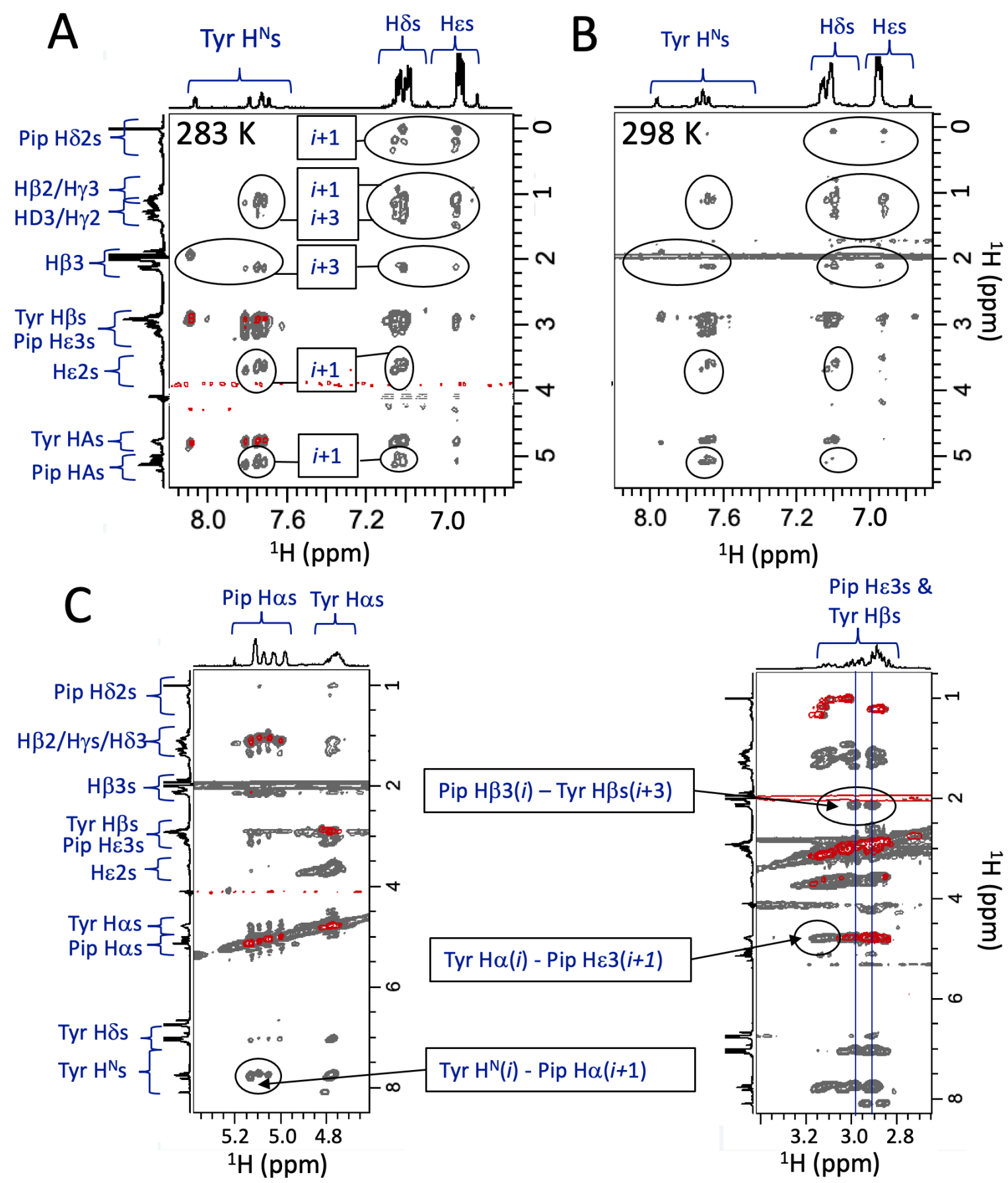

(A-B) 2D ^{1}H-^{1}H NOESY of DPR1 5x in 2:1 CD_3CN : H_2O at (A and C) 283 K and (B) 298 K. Cross peaks in the black circles are more intense at lower temperatures. These are (*i*+1) NOEs between Tyr ring protons and Pip Hβ2, Hδ2, and Hε2s protons, and (*i*+3) NOEs to Hβ3 and Hγ3. (A and C) NOESY (gray) and TOCSY (red) spectra at 283 K show the characteristic *trans* NOEs from Pip Hε2/3s(*i*+1) to Tyr Hαs(*i*) and Pip Hβ3(*i*) to Tyr Hβs(*i*+3). (See Table S5 for a complete list of supporting NOEs). NOEs between Tyr Hβs and Pip Hε3s are overlapped with those between Pip Hε3s and Hβ2/Hγs. These NOESY data were validated against the models using a customized version of PDBstat (v5.23)[20] that includes D-Pip to generate a simulated peak list with the simPL (simulate peak list) command and a defined distance cutoff (< 5.0 Å). The input data were BMRB-formatted chemical shifts and PDB-formatted model coordinates. The output was a file with simulated peaks and corresponding assignments that were read into Sparky (rp - read peaks command). There were no NOESY peaks that were inconsistent with the models, and no simulated NOESY peaks were absent for interproton distances < 4 Å in the model.

Figure S15. DPR1 NMR ring current shifts

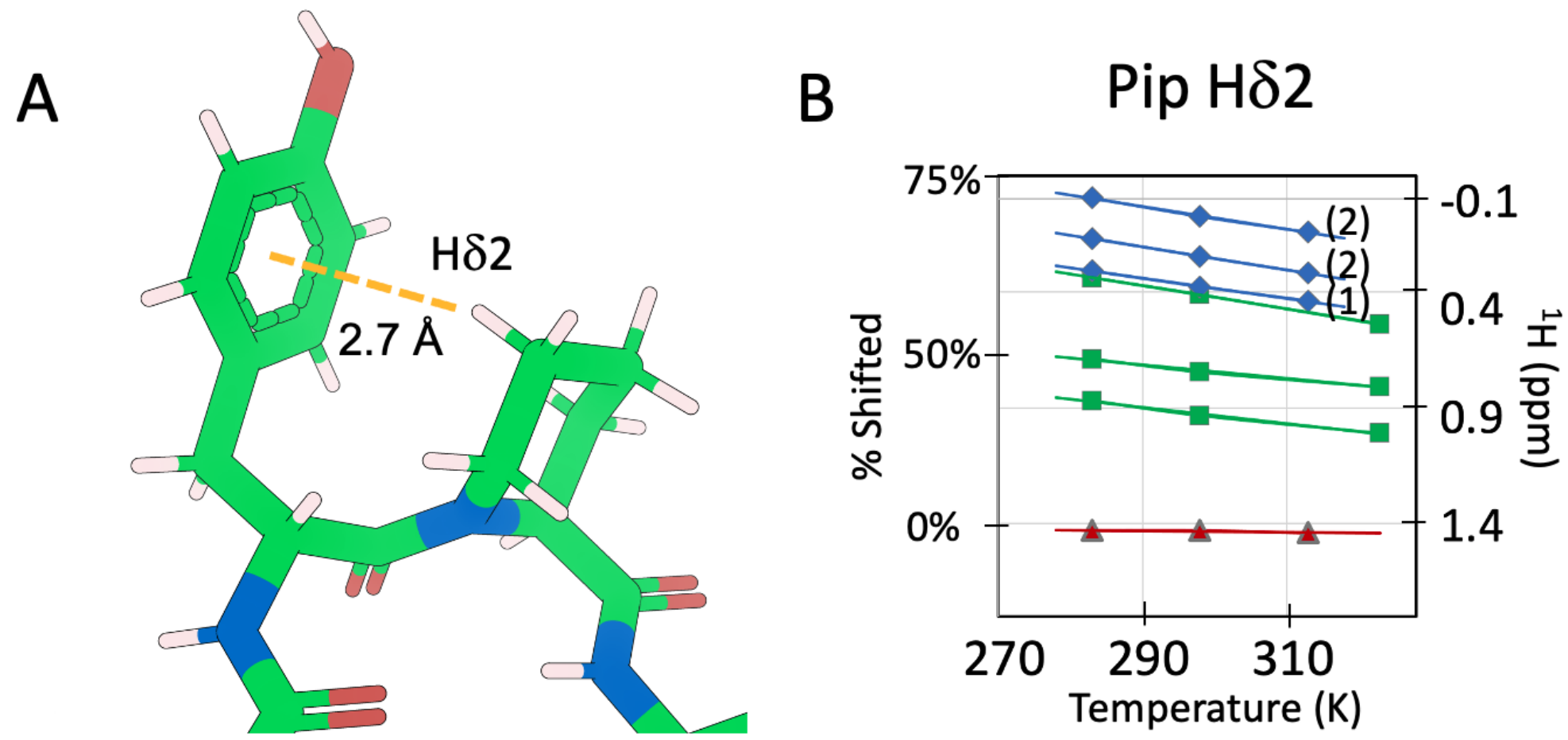

(A) Design model of a single DPR1 dipeptide subunit showing the axial Pip Hδ2 proton - Tyr ring distance is 2.7 Å with an azimuthal angle of 12°, which would create an upfield ring current shift. (B) Pip Hδ2 shifts vs. temperature, color-coded by polymer length with 1x (red), 3x (green), and 5x (blue). The percent shifted at 283 K (left side y-axis) was calculated for each resolved proton by subtracting the 1x shift and dividing by the maximum expected ring current shift of 2.0 ppm. These shifts act as probes for local polymer structure. In the 5x DPR1 ^{1}H-^{13}C HSQC spectrum, two of the Pip Cδ-Hδ2 cross peaks overlap, and therefore, the proton counts for each peak are given in parentheses. **Quantitation of ring current shifts.** Analysis of these shifts estimates a 65% average helical structure across the sequence for 5x DPR1 at 10° C. The percent shifted for each resolved Hδ2 resonance was determined by comparing its shift to the 1x control. It was reported as a percentage relative to a maximum expected upfield shift resulting from ring-current effects. For the three resolved Pip Cδ-Hδ2 cross peaks, this yielded helical estimations of 72% (for two subunits), 63% (2), and 56% (1) for the five repeat shifting peaks (Figure 4G). This indicates that the helical structure is not uniformly distributed across the polymer. A similar analysis of the more disordered 3x DPR1 gives estimations of 55%, 37%, and 28% with a weighted average of ~40% at 283 K. These estimates are a lower bound for the percent ordered based on a maximum expected ring current shift of 2.0 ppm.

Figure S16. DPR2 1D spectra and ^{1}H-^{15}N HSQCs

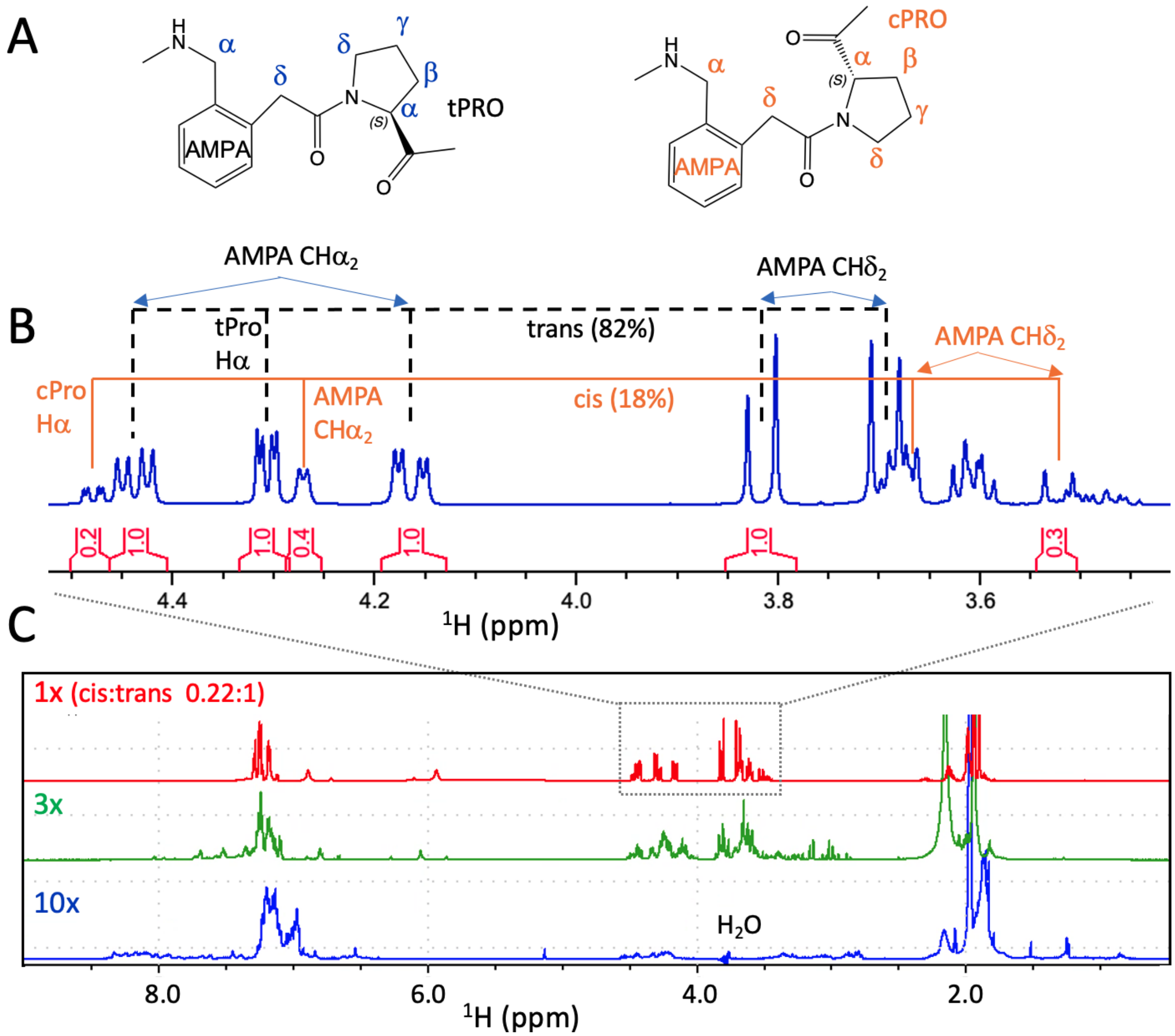

(A) Chemical drawing of *trans* and *cis* AMPA-Pro. (B) 1D ^{1}H NMR spectra of 1x DPR2 in CD_3CN at 298 K. Cis and trans peak integrations are shown. The average ratio of cis : trans populations was 0.22 : 1. (B) 1x, 3x DPR2 in CD_3CN, and 10x DPR2 in 2 : 1 CD_3CN : H_2O. The 10x DPR2 showed characteristic downfield shifts in multiple NH signals indicative of intramolecular hydrogen bonding. The upfield shifts of some aliphatic resonances are attributed to ring current shifts from more ordered folded conformations.

Figure S17. 1x and 10x DPR2 ^{1}H-^{13}C HSQC spectra

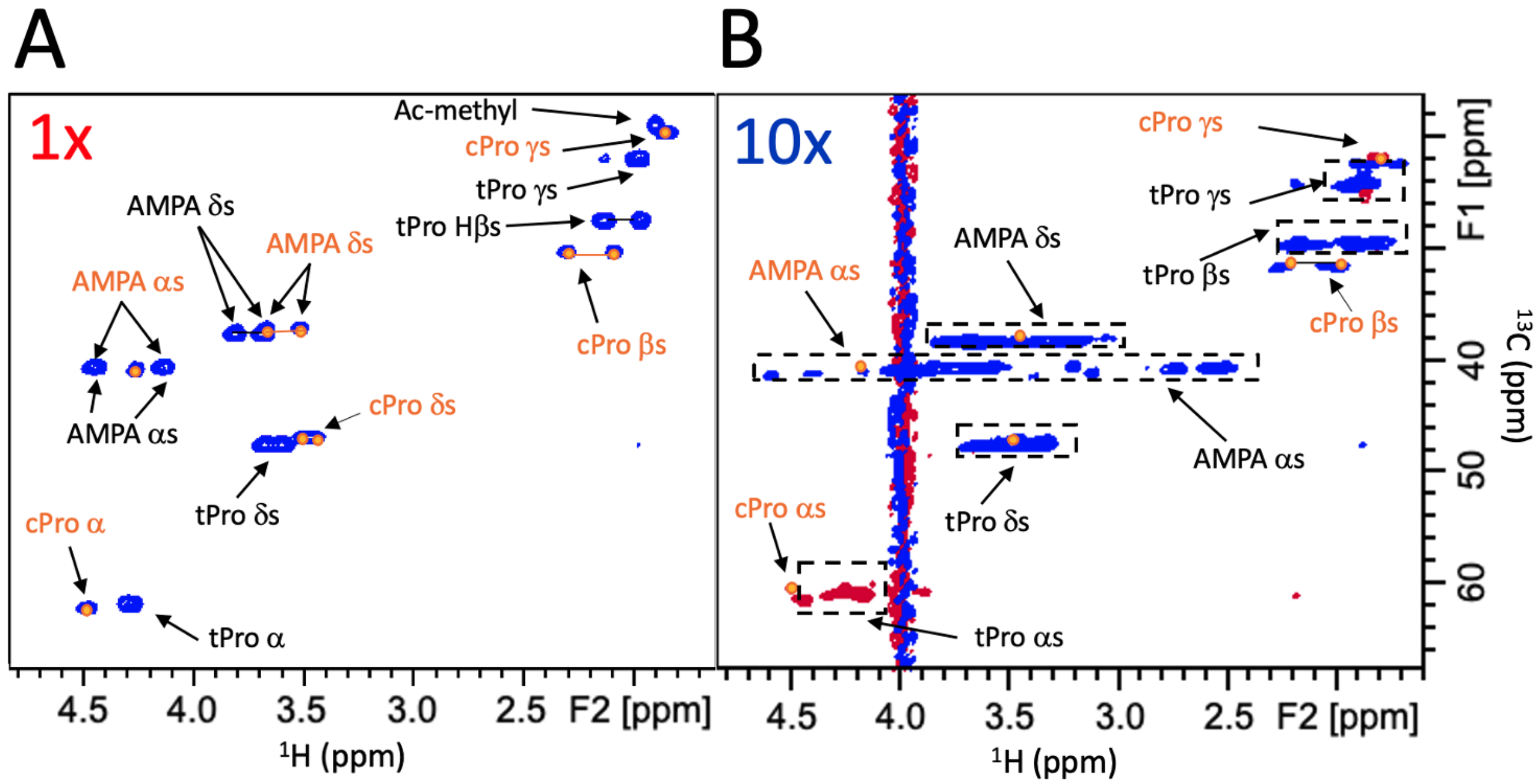

(A) 2D ^{1}H-^{13}C HSQC spectra of 1x DPR2 in CD_3CN and (B) 10x DPR2 in 2:1 CD_3CN : H_2O recorded at 283 K with (C) Schematic of cis (top) and trans (bottom) dipeptide units. Chemical shift assignments for the cis and trans conformers were determined by the identification of characteristic NOEs. Specifically, the trans conformations displayed strong NOEs between the AMPA $C^{\delta}H_2$s and Pro Hδs, while the cis conformations had strong NOEs between the AB $C^{\delta}H_2$s and Pro Hα. Peak positions from cis dipeptide (AMPA-cPro) in the 1x spectrum are marked with orange circles in both the 1x and 10x spectra and labeled (orange text). In the 10x spectrum, peaks belonging to trans AMPA-tPro repeats are outlined by a black dashed box (10% *cis*, 90% *trans*). The AMPA Hα2/3 protons were shifted significantly upfield compared to the shorter 1x at the same temperature due to ring current shifts. The length-dependent shifts give support for the designed model, where both AMPA methylene Hα2 and Hα3 atoms are positioned above the neighboring AMPA (*i*+2) residue's aromatic ring, leading to shielding effects.

Figure S18. 10x DPR2 ^{1}H-^{1}H NOESY and TOCSY.

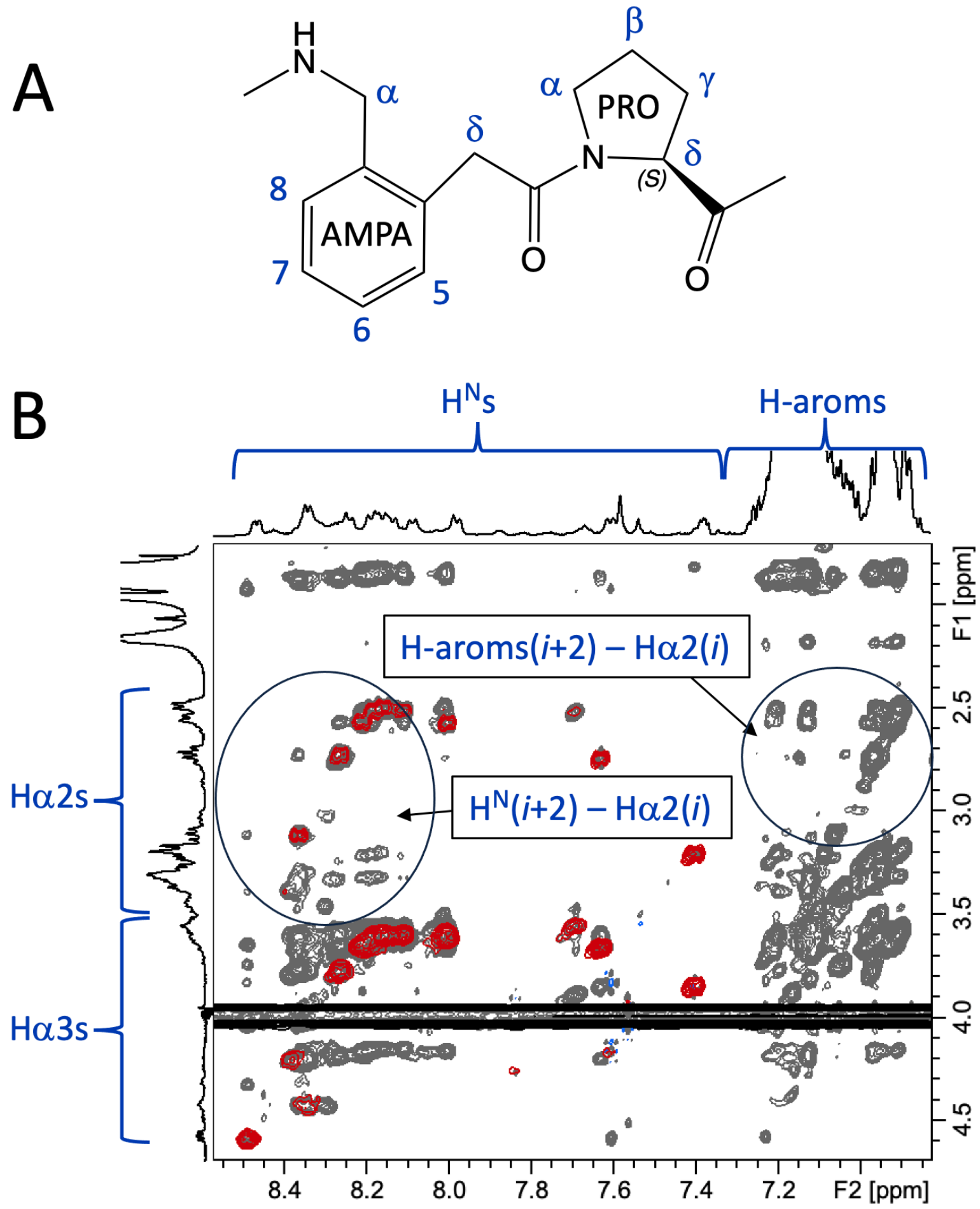

(A) Labeled schematic of DPR2 (B) 2D ^{1}H-^{1}H TOCSY (red) and (B) NOESY (black) spectra of 10x DPR2 in 2:1 CD_3CN : H_2O at 283 K with AMPA protons labeled. The upfield shifted AMPA Hα2 protons from 2.4 - 3.2 ppm do not overlap with any other protons. Circled NOEs not overlaid by TOCSY peaks can be attributed to *i* to *i*+2 NOEs that support the structure (with boxed labels). These include NOEs from the shifted AMPA methylene Hα2/3s to both (*i*+2) H^N and aromatic ring protons.

Figure S19. 10x DPR2 ^{1}H-^{13}C HSQCs with percentage of cis at decreasing temperature

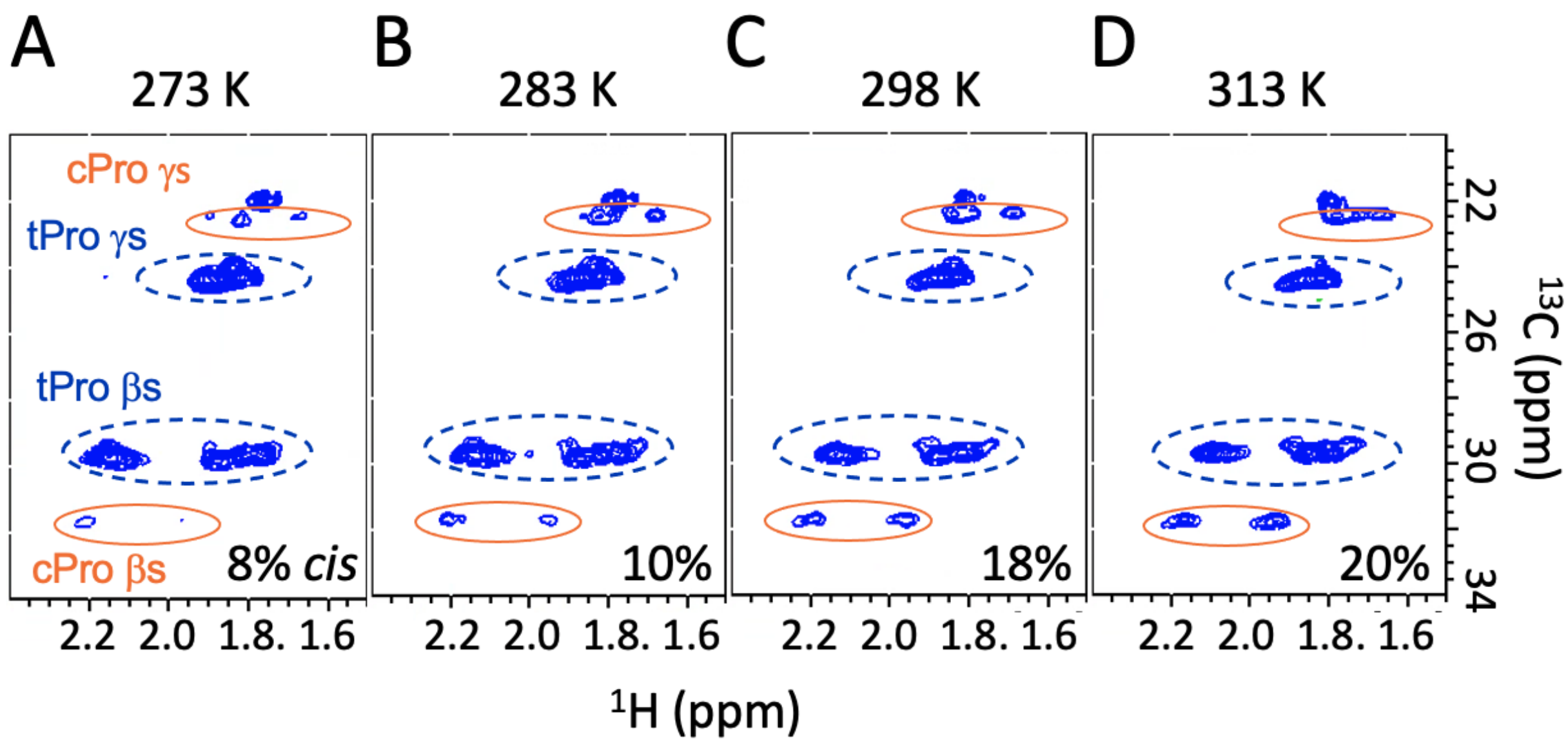

^{1}H-^{13}C HSQC spectra of 10x DPR2 recorded at four temperatures, 273 283, 298, and 313 K. The percentage of *cis* and *trans* conformations were obtained by integration of cPro (orange circles) and tPro (blue dashed circles) $C^{\beta}H\beta2$ and $H\beta3$ cross peaks for each of the conformations using ellipses in Sparky[21]. Determination of cis/trans ratios from 1D NMR spectra was not possible due to spectral overlap, however in these 2D spectra, the percent of the low energy trans population increases from 80 to 92% upon cooling to 0 °C, consistent with stabilization of the predicted all-trans helical conformation.

Figure S20. 10x DPR2 changes with temperature

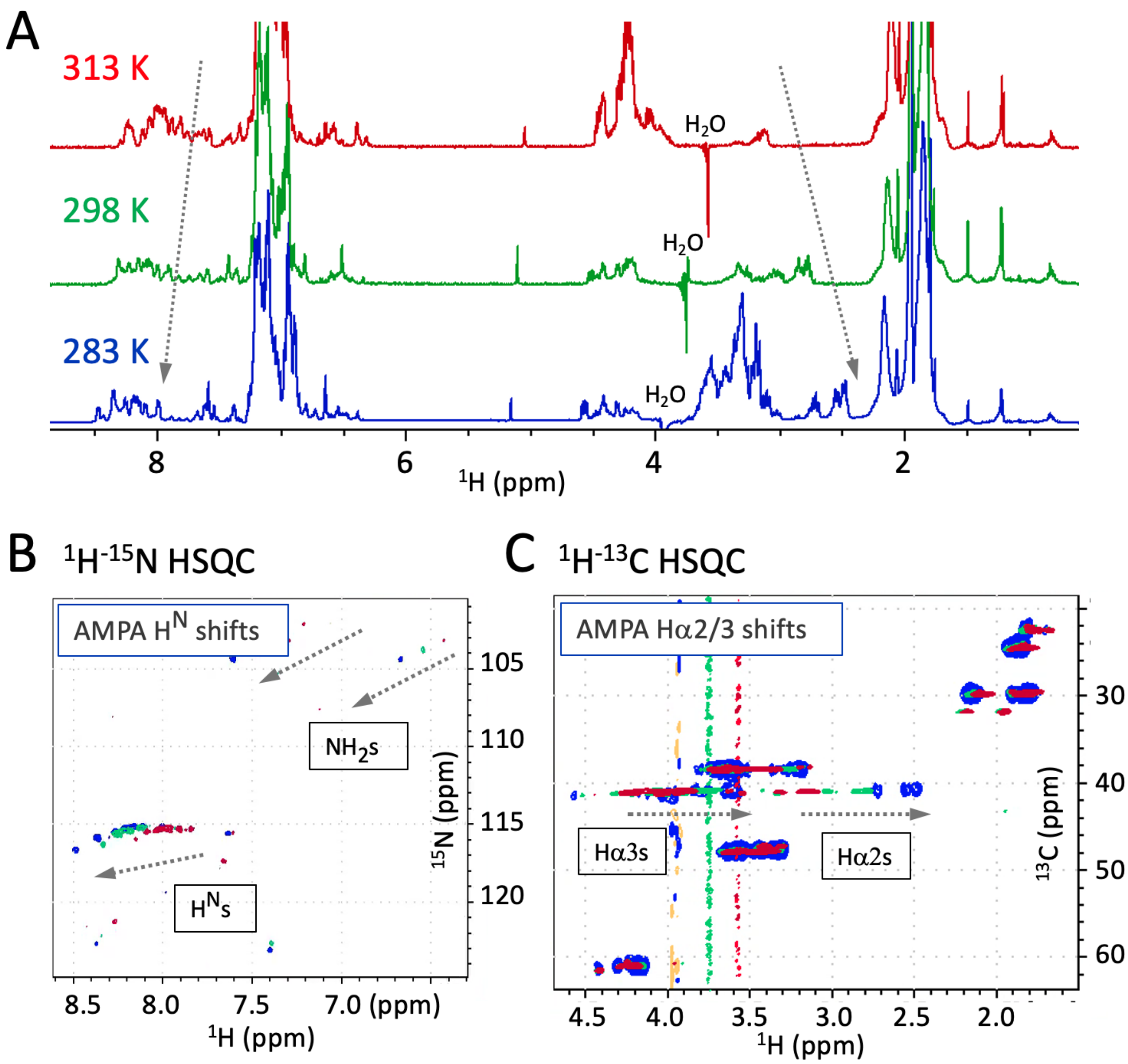

(A) 1D ^{1}H-NMR, (B), 2D ^{1}H-^{15}N HSQC, and (C) ^{1}H-^{13}C HSQC spectra recorded on 10x DPR2 at three temperatures: 283 (blue), 298 (green), and 313 K (red). In the (A) 1D ^{1}H-NMR and (B) 2D ^{1}H-^{15}N HSQC spectra, the H^N peaks shift downfield as the temperature is lowered from 313 to 283 K (gray dashed arrows). In (A) 1D and (C) 2D ^{1}H-^{13}C HSQC spectra, the AMPA $C^{\alpha}H_2$ protons from the *trans* conformations shifted upfield as the temperature was lowered (gray dashed arrows), consistent with increased helical structure.

Figure S21. 10x DPR2 ^{1}H-^{1}H TOCSY and AMPA peak shifts with temperature

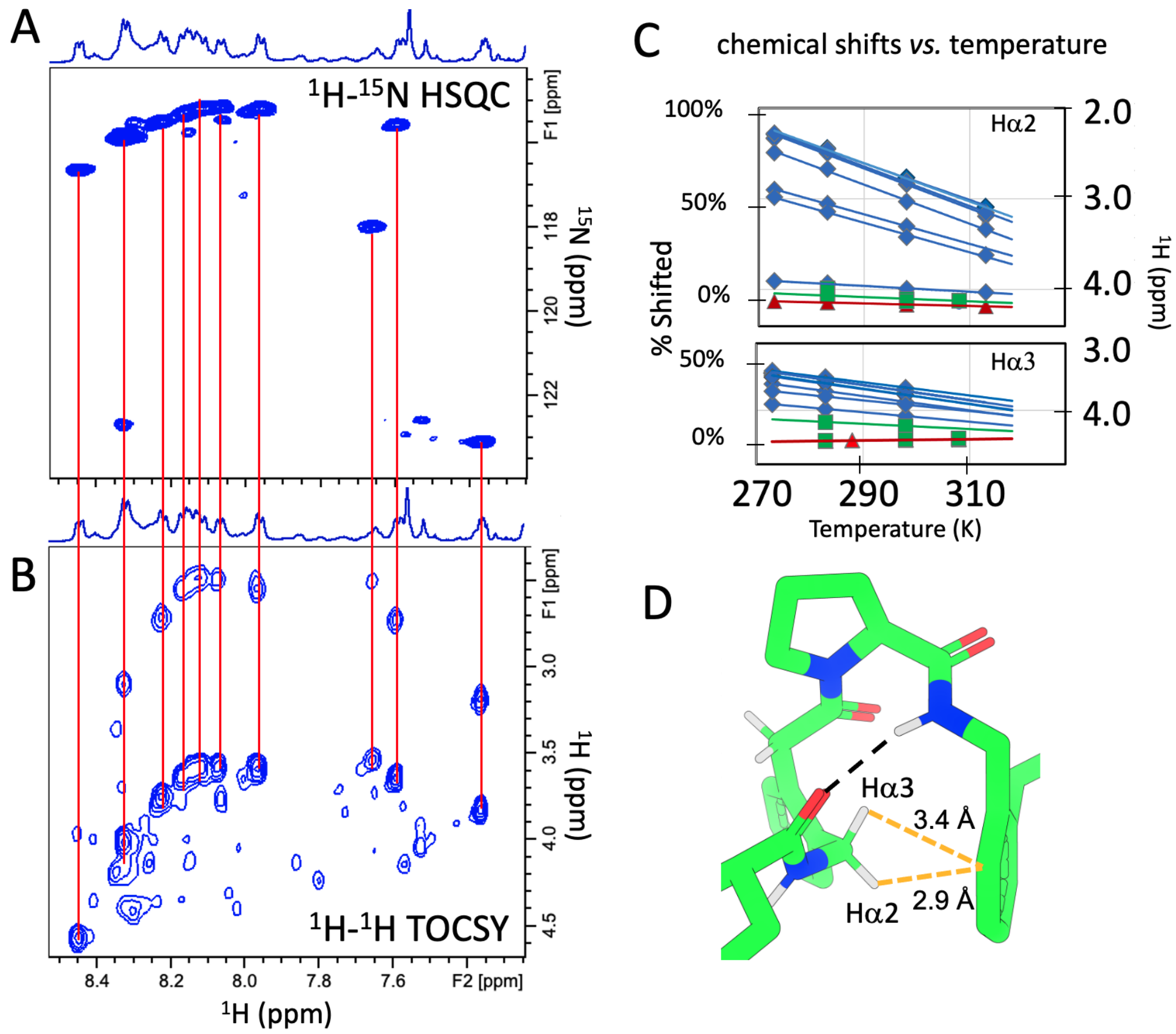

(A) 2D ^{1}H-^{15}N HSQC and (B) ^{1}H-^{1}H TOCSY spectra of 10x DPR2 in 2:1 CD_3CN : H_2O. Intraresidue AMPA H^N to Hα2 and Hα3 cross peaks are indicated for the ten strongest H^N peaks. (C) AMPA methylene Hα2/3 shifts from 1D or TOCSY spectra, color-coded by polymer length (1x in red, 3x in green, and 10x in blue), are plotted vs. temperature. Stereospecific assignments were confirmed via NOEs to (*i*) H^N and (*i*+2) H^Ns. The observed results are consistent with the model, and a smaller maximum expected ring-current shift for Hα3 compared to the closer Hα2. Percent ordered structure (% shifted) was calculated for each proton by subtracting the 1x shift and dividing by the maximum expected ring current shift of 2.0 ppm. The ring-current shifts indicate ordered populations of 11% to 93% (average ~75%) using the ten different AMPA Hα2 and 27 to 52% with the Hα3 protons, assuming a maximum 2.0 ppm shift for both sites. Since the maximum shift for Hα3 is expected to be less than Hα2 in the designed structure, the population estimates from Hα3 are conservative. Minimal Hα2 and Hα3 ring current shifts (0% and 11%) at the helix N-terminus suggest fraying. The estimated average helical structure for 3x at 283 K is ~6%. (D) Expanded view of the DPR2 model helical structure, indicating polar contacts between AMPA (*i*) methylene Hα2 and Hα3 atoms located 2.9 and 3.4 Å above the AMPA (*i*+2) aromatic ring, with azimuthal angles of 16° and 21°, respectively.

Figure S22. 1x DPR3 1D ^{1}H NMR

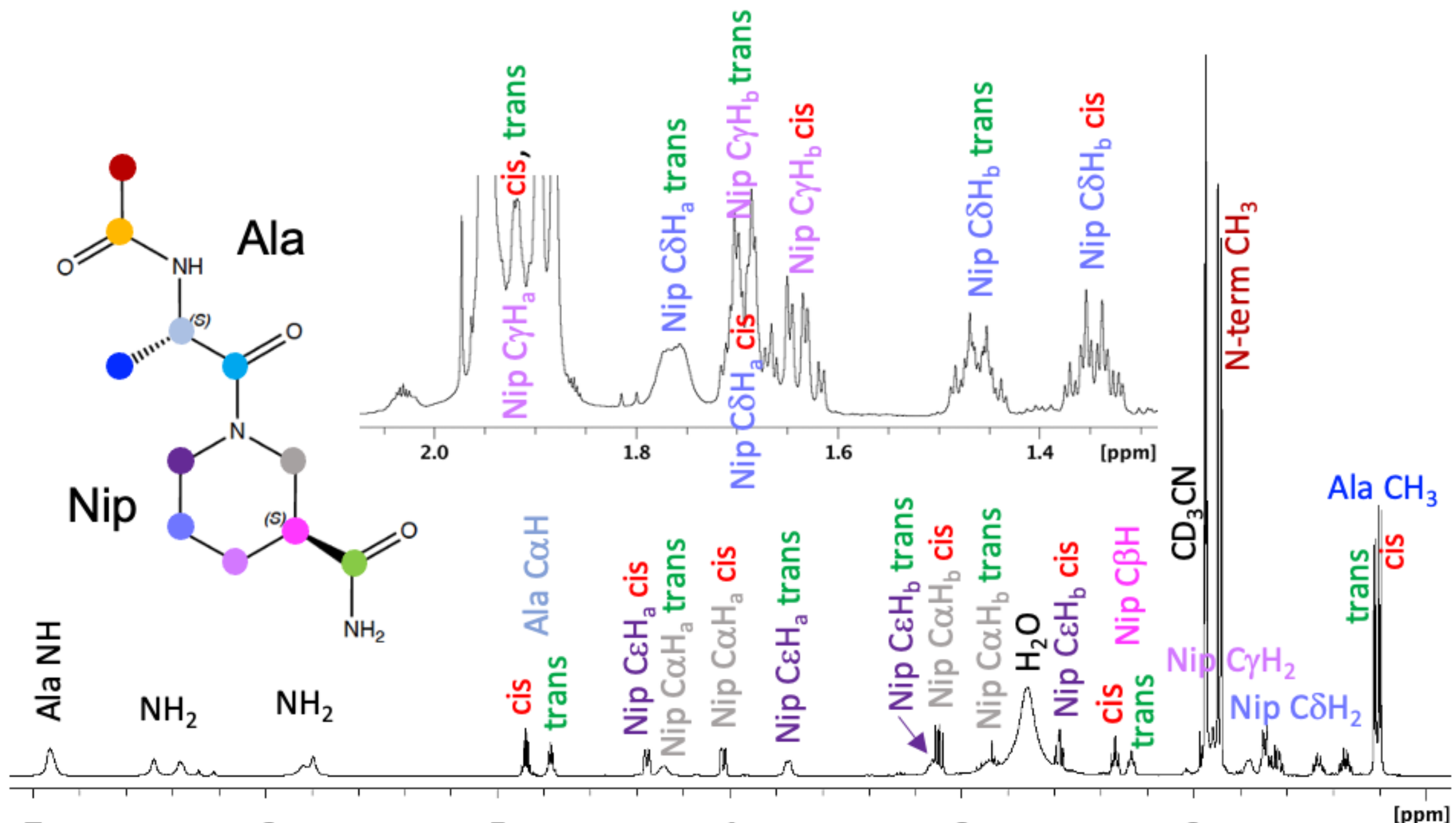

1D ^{1}H NMR spectrum of 1x DPR3 in CD_3CN at 283 K, with assignments. 1x DPR3 shows both *cis* and *trans* conformations about the peptide bond between the Ala and Nip residues in a cis:trans ratio of 1.1:1, measured by integration of the resolved resonances. Assignments were determined from COSY, TOCSY, ROESY, HSQC, HSQC-TOCSY, and HMBC spectra.

Figure S23. 1x DPR3 COSY, ROESY, HSQC NMR

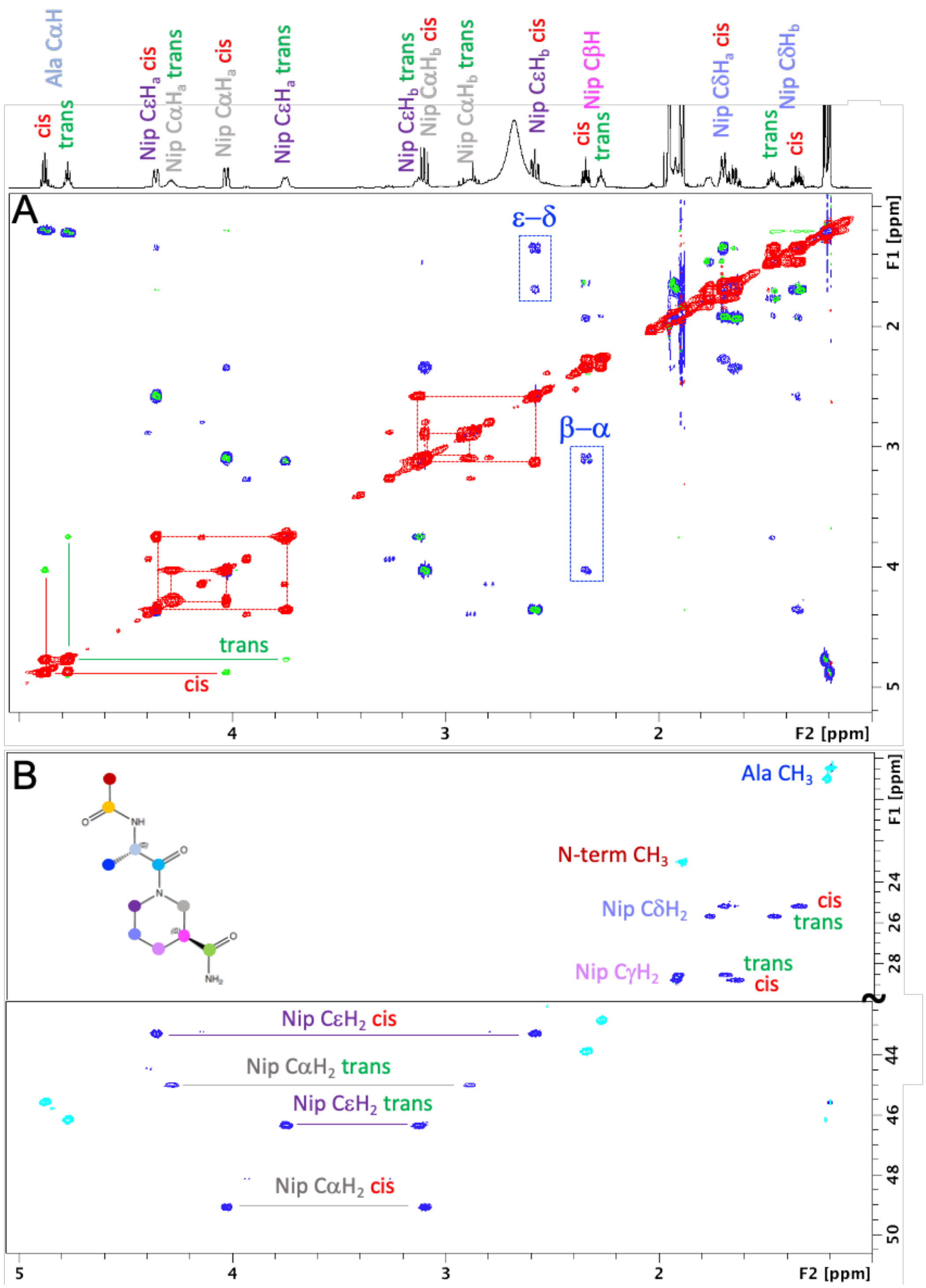

NMR spectra of 1x DPR3 in CD_3CN at 283 K. (A) Assignment of *cis* and *trans* conformational states through ROEs between Ala CαH and Nip $C\varepsilon H_2$ (*trans*) or $C\alpha H_2$ (*cis*). The Nip $C\varepsilon H_2$ and $C\alpha H_2$ were assigned by COSY connections to the more upfield $C\delta H_2$ or more downfield CβH resonances, respectively. The COSY spectrum (blue) is overlaid with the ROESY spectrum (red, light green). Red dashed rectangles connect *cis-trans* conformational exchange peaks. Blue dashed rectangles enclose Nip Hα-Hβ and Hε-Hδ COSY cross peaks that assign cis Hα and Hε. Exchange peaks along with COSY cross-peaks between resonances designated H_a and H_b allow for the remaining assignments of Hα and Hε. Designations H_a and H_b are arbitrary and not stereospecific assignments. ROESY cross peaks defining the *cis* and *trans* molecules are shown with red (*cis*) and green (*trans*) solid lines. (B) HSQC spectrum aligned along the ^1H dimension with the COSY/ROESY spectra of panel A. The Nip assignment patterns in the carbon dimension for *cis* and *trans* Nip $C\alpha H_2$ and $C\varepsilon H_2$ were used to interpret the spectra for longer DPR3 and also for DPR4, which also has cis and trans Nip conformations.

Figure S24. 10x/20x DPR3 COSY NMR

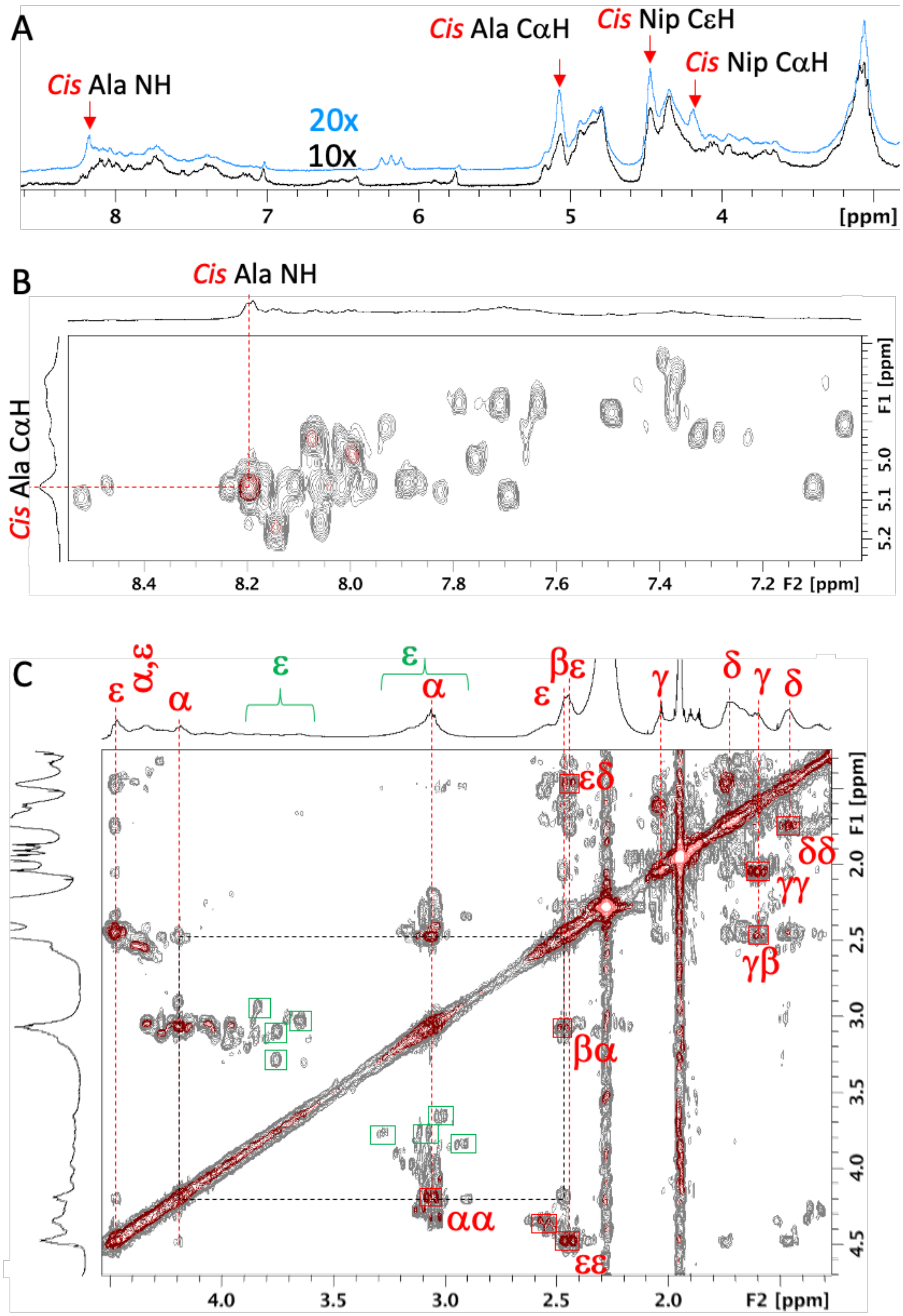

NMR spectra of 10x and 20x DPR3 in CD_3CN at 283 K. Both 10x and 20x DPR3 show conformational states with both *cis* and *trans* conformations of the peptide bond

between the Ala and Nip residues, with conformational exchange between these *cis* and *trans* ensembles. ^{1}H resonances are broad in the 1D spectrum, representing multiple protons with a range of chemical shifts, and the resonances for Nip $C\alpha H_2$ and $C\varepsilon H_2$ overlap, making detailed assignments difficult. Spectra of 10x and 20x are similar, except that for the 20x, some *cis* conformers appear to increase. (A) Comparison of 1D ^{1}H NMR spectra of 10x DPR3 (black) and 20x DPR3 (blue) in CD_3CN at 273 K, with key *cis* resonances labeled. (B,C) COSY spectrum (gray) overlaid with COSY spectrum with a higher threshold (red) of 20x DPR3 in CD_3CN at 283 K, illustrating the dominance of some cis conformers. (B) The Ala NH-CαH region shows more than 20 cross peaks of different intensities, potentially representing both sequentially different Ala residues and different conformations. The strongest NH-CαH cross peak, assigned to *cis* conformer(s), is labeled. (C) Higher intensity peaks for Nip resonances are labeled with *cis* in red and *trans* in green, showing again that one or a close group of *cis* conformations is slightly favored; the large number of smaller Nip $C\alpha H_2$ and $C\varepsilon H_2$ resonances are challenging to assign due to spectral overlap.

Figure S25. 20x DPR3 COSY, ROESY, HSQC NMR

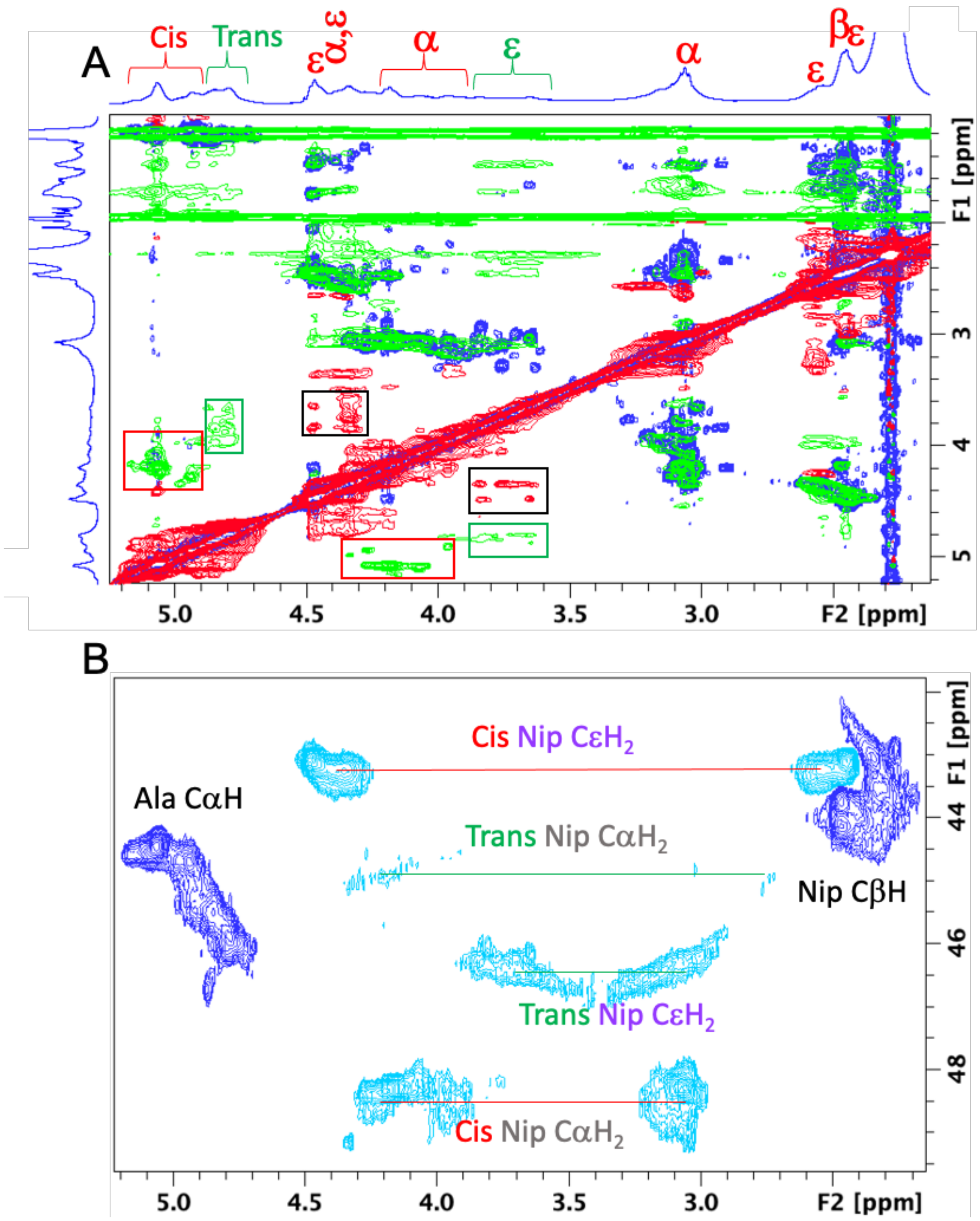

NMR spectra of 20x DPR3 in CD_3CN at 283 K. Both *cis* and *trans* configurations are present in comparable amounts and exchange with each other. (A) Ala CαH and Nip $C\alpha H_2$ - $C\varepsilon H_2$ region of COSY spectrum (blue) overlaid with ROESY spectrum (red, light green). Exchange peaks between *cis* and *trans* Nip CεH are enclosed in black rectangles. Exchange peaks between *cis* and *trans* Nip CαH are not observed because

trans Nip CαH resonances are too weak to identify, or the cross-peaks are very close to the diagonal due to close overlap between *cis* and *trans* Nip CαH resonances. Exchange between the *cis* and *trans* configurations indicates that despite longer lengths (20x) and lower temperatures (273 K, Figure S23A), the predicted low-energy *cis* conformation was not significantly stabilized. ROESY cross peaks defining the *cis* and *trans* conformational states are enclosed in red (*cis*) and green (*trans*) rectangles. The approximate ratio of *cis/trans* from integration of the Ala CαH peaks is 1.1:1, the same as observed for the 1x DPR3 sample. However, the *trans* Nip $C\varepsilon H_2$, and more markedly, $C\alpha H_2$, are weaker than expected based upon this ratio. (B) HSQC spectrum aligned along ^1H dimension with COSY/ROESY in A, showing the same pattern of carbon chemical shifts as for 1x DPR3, but different overlapped proton shifts, and weak *trans* Nip $C\alpha H_2$ peaks.

Figure S26. 1x DPR4 1D ^{1}H NMR

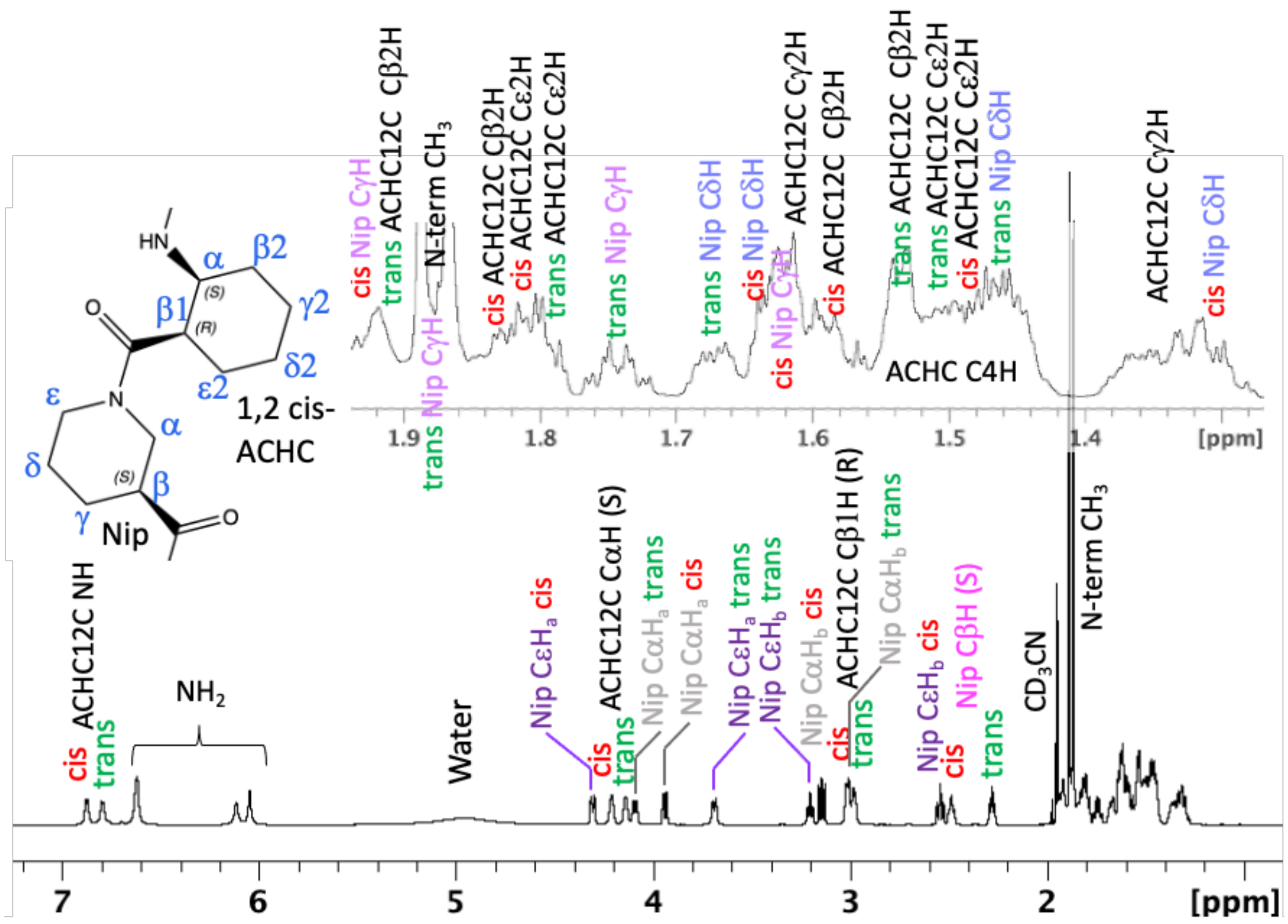

1D ^{1}H NMR spectrum of 1x DPR4 in CD_3CN at 283 K, with assignments. 1x DPR4 shows both *cis* and *trans* conformations about the peptide bond between the 1,2-cis-ACHC and Nip residues in a cis:trans ratio of ~ 1.0:1.0, measured by integration of the resolved resonances. Assignments were determined from COSY, TOCSY, ROESY, HSQC, HSQC-TOCSY, and HMBC spectra.

Figure S27. 1x DPR4 COSY, ROESY, HSQC NMR

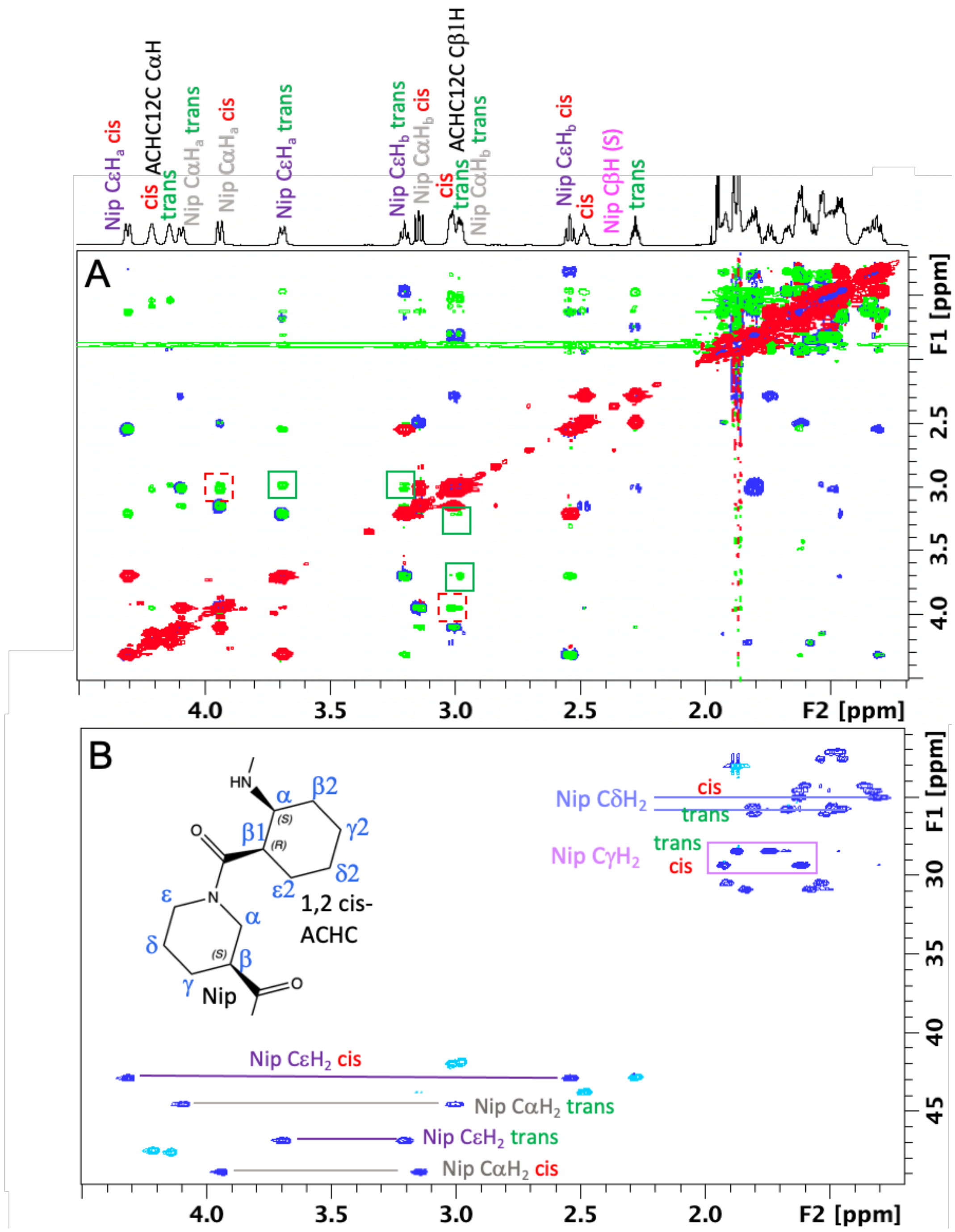

NMR spectra of 1x DPR4 in CD_3CN at 283 K, showing the assignment of *cis* and *trans* conformational states through ROEs between 1,2-cis-ACHC Cβ1H and Nip $C\varepsilon H_2$ (*trans*) or $C\alpha H_2$ (*cis*), with Nip $C\varepsilon H_2$ and $C\alpha H_2$ defined by COSY connections to the more upfield $C\delta H_2$ or more downfield CβH, respectively. (A) COSY spectrum (blue) overlaid with ROESY spectrum (red, light green). Designations H_a and H_b are arbitrary and not

stereospecific assignments. Exchange cross peaks in red connect all corresponding *cis-trans* pairs in the 2 – 4.5 ppm region. ROESY cross peaks defining the *trans* molecules are enclosed in green solid squares. The *cis* ROESY cross peaks enclosed in red dashed squares, between 1,2-cis-ACHC Cβ1H and Nip CαH, overlap the Nip *cis* $C\alpha H_a$ - *trans* CaH_b peak arising due to exchange. Other ROE cross peaks that would define *cis* are either too close to the diagonal for exchange peaks to be observed. (B) HSQC spectrum at 283 K aligned along ^1H dimension with the COSY/ROESY in panel A. The assignments for 1x cis and trans DPR4 peaks were used as a guide for interpreting NMR data for the longer-length DPR4 polymers.

Figure S28. 10x DPR4 COSY NMR

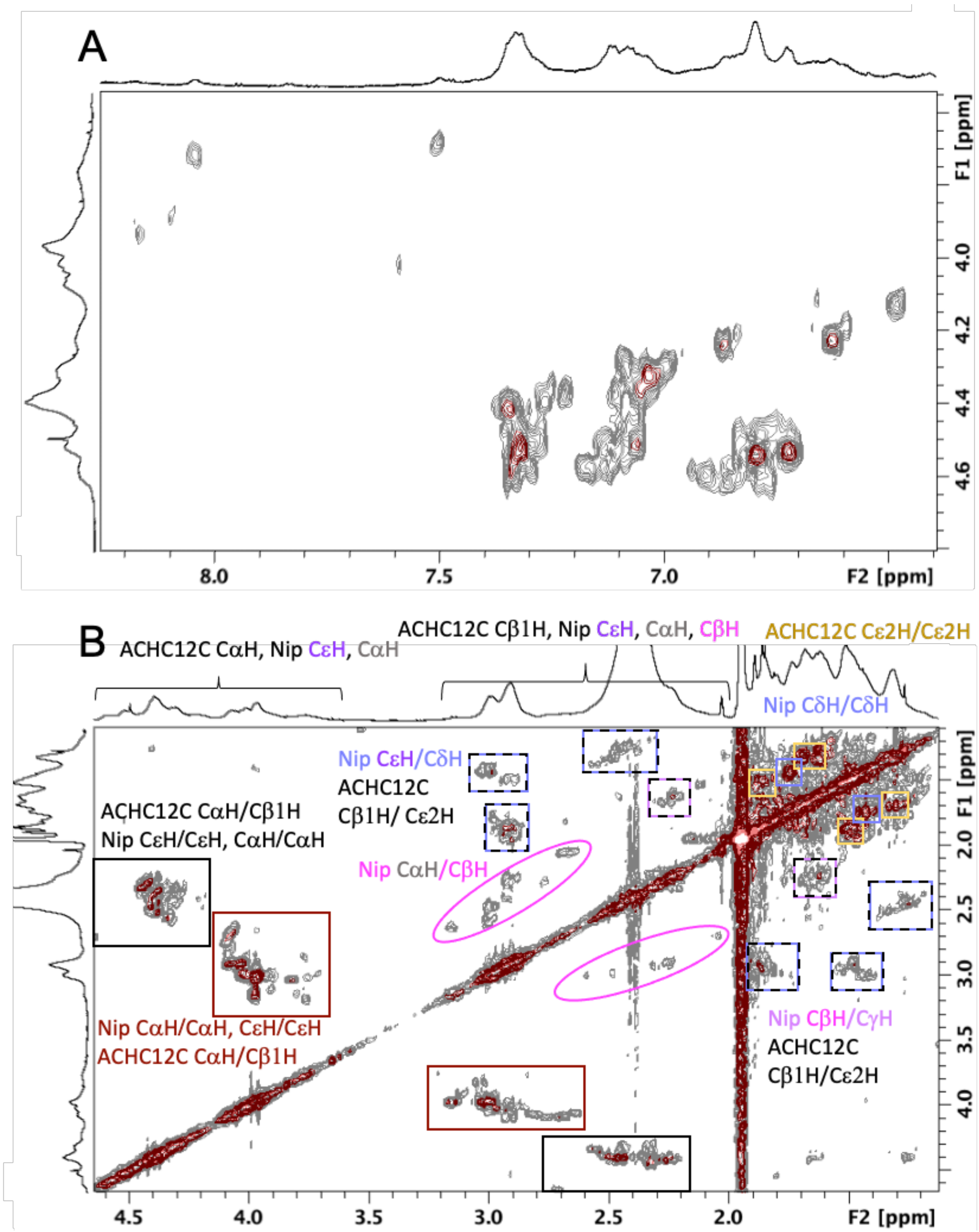

(A) COSY spectrum (gray) of 10x DPR4 in CD_3CN at 283 K, overlaid with the same COSY spectrum with a higher threshold (red), shows that in contrast to 20x DPR3 (Figure S23), there are multiple prominent 1,2 cis-ACHC CαH. (A) In the NH-CαH region, more than 20 cross peaks of different intensities are present, potentially representing both sequentially different 1,2 cis-ACHC residues and different slowly

exchanging conformational states. (B) Tentative resonance assignments. Extensive overlap of resonances of the aliphatic ring structures of 1,2 cis-ACHC and Nip makes more detailed resonance assignments too difficult.

Figure S29. 10x DPR4 COSY, ROESY, HSQC NMR

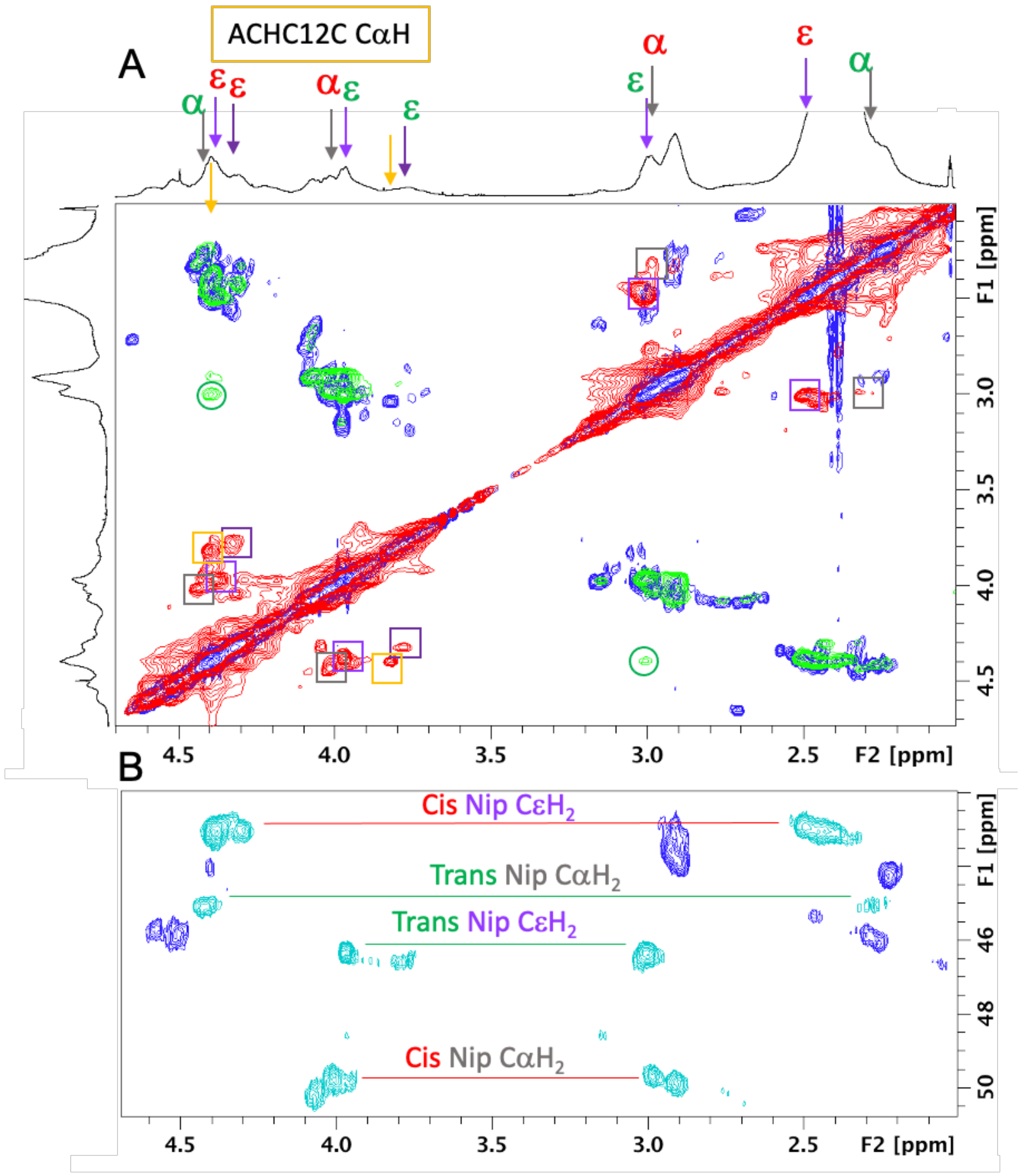

NMR spectra of 10x DPR4 in CD_3CN at 283 K. As for DPR3 (Figure S24), both *cis* and *trans* configurations are present in comparable amounts and are exchanging with each other. (A) Nip $C\alpha H_2$ - $C\varepsilon H_2$ region of COSY spectrum (blue) overlaid with ROESY spectrum (red, light green). *Cis-trans* exchange peaks are enclosed in squares: gray for Nip $C\alpha H_2$, purple for Nip $C\varepsilon H_2$, and gold for 1,2 cis-ACHC CαH. ROESY cross peaks in green circles probably indicate the proximity of 1,2 cis-ACHC CαH (*i*) and Nip $C\varepsilon H_2$ (i+1) in the *trans* conformation. The extensive overlap and tentative nature of the assignments make it impossible to distinguish *cis* and *trans* by the method of identifying Nip CαH and CεH in the COSY followed by analysis of ROESY cross peaks, which here

would be between 1,2-cis-ACHC Cβ1H and Nip CαH (*cis*) or CεH (*trans*). However, there are *cis*-*trans* exchange peaks in the ROESY spectrum for all four pairs tentatively assigned to resonances of Nip CαH_2 and CεH_2. (B) HSQC spectrum aligned along ^1H dimension with the COSY/ROESY in *A*. The presence of about four distinct groupings of $^{13}CH_2$ peaks between 42 and 51 ppm in the 10x DPR4 HSQC spectrum indicates both *cis* and *trans* configurations are present, and this pattern resembles that of 1x DPR3 (Figure S22B), 20x DPR3 (Figure S24B), and 1x DPR4 (Figure S26B). By analogy to these other HSQC spectra, the *cis* and *trans* Nip CαH_2 and CεH_2 of 10x DPR4 can be tentatively assigned, and these assignments are consistent with tentative assignments of the COSY spectrum (Figure S27B). Overlay of the HSQC-TOCSY (not shown) and HSQC was helpful in distinguishing the 1,2-cis-ACHC and Nip resonances.

Supplemental Tables

Table S1. Residue list (SMILES format and structural drawings) and calculation of unique dipeptide combinations

SMILES chemical format for 130 amino acids includes N-terminal acetylation and C-terminal N-methylation, which were included in the energy simulation of the monomers to better represent the environment in a polypeptide. Amino acids starting with “m” are mirror images and have the opposite chirality of the version without “m”. Note that each residue was additionally calculated as a preproline variant. In those cases, the C-terminal cap was adjusted to a di-methyl amidation, which approximates the effects of proline-like residues, which is well documented to affect the torsional preferences of the residue preceding it in canonical α-amino acids.[22]

This study considered 130 unique residues in total. 92 of the residues were chiral, and both chiralities of the residues were considered, yielding a total of 222 residues. Therefore, after filtering for redundant pairs of dipeptides that happen due to sequential permutation (AGLY-BGLY vs BGLY-AGLY) and complete mirror symmetry (ALA-B3ALA vs mALA-mB3ALA), there are a total of 20,475 unique dipeptide combinations.

The snippet for this calculation is provided (where monomers.txt is a line delimited file of the monomers in the below table):

```
monomers = open('monomers.txt', 'r').readlines()
pairs = [frozenset(pair) for pair in itertools.product(monomers, repeat=2)]
pairs_no_mirrors = [pair for pair in pairs if not all([res.startswith('m') for res in pair])]
unique_pairs_no_mirrors = set(pairs_no_mirrors)
print(len(unique_pairs_no_mirrors))
> 20475
```

1	AGLY	CC(=O)NCC(=O)NC	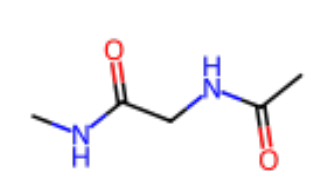
2	BGLY	CC(=O)NCCC(=O)NC	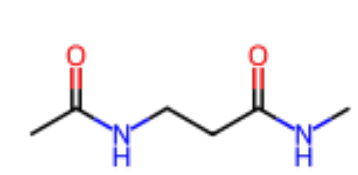

3	GGLY	CC(=O)NCCCC(=O)NC	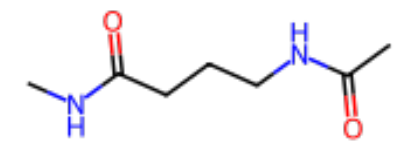
4	DGLY	CC(=O)NCCCCC(=O)NC	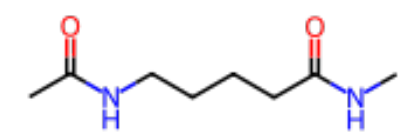
5	EGLY	CC(=O)NCCCCCC(=O)NC	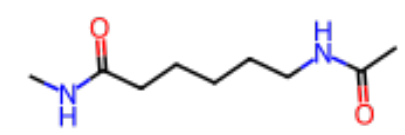
6	ALA	CC(=O)N[C@@H](C)C(=O)NC	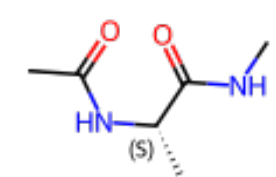
7	ABU	CC(=O)N[C@@H](CC)C(=O)NC	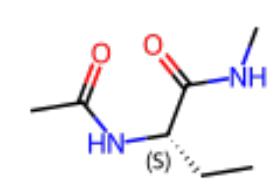
8	SER	CC(=O)N[C@@H](C[OH])C(=O)NC	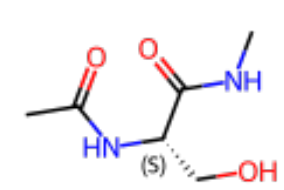
9	ASN	CC(=O)N[C@@H](CC(=O)N)C(=O)NC	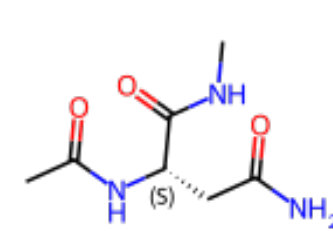

10	CLA	CC(=O)N[C@@H](C[Cl])C(=O)NC	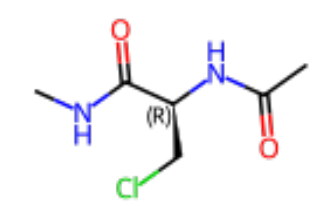
11	VAL	CC(=O)N[C@@H](C(C)C)C(=O)NC	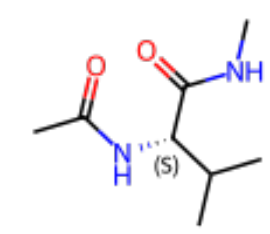
12	TBG	CC(=O)N[C@@H](C(C)(C)C)C(=O)NC	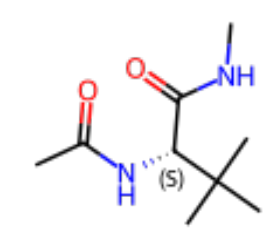
13	NVL	CC(=O)N[C@@H](CCC)C(=O)NC	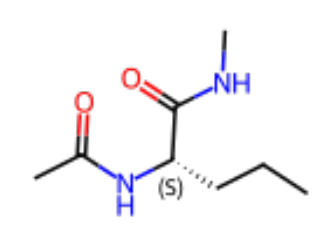
14	LEU	CC(=O)N[C@@H](CC(C)C)C(=O)NC	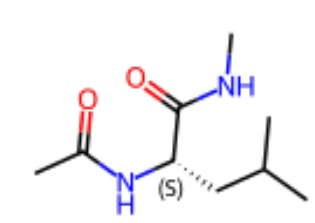
15	TBA	CC(=O)N[C@@H](CC(C)(C)C)C(=O)NC	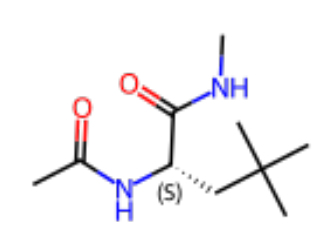
16	PHG	CC(=O)N[C@@H](c1ccccc1)C(=O)NC	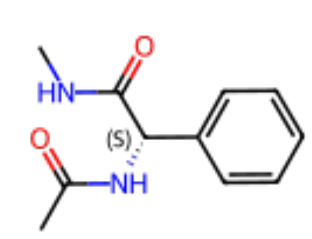

17	PHE	CC(=O)N[C@@H](Cc1ccccc1)C(=O)NC	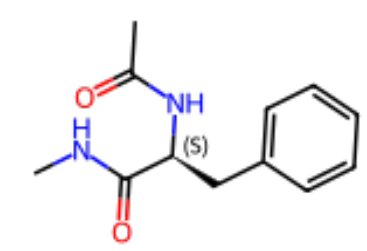
18	FPA	CC(=O)N[C@@H](Cc1c(F)c(F)c(F)c(F)c1(F))C(=O)NC	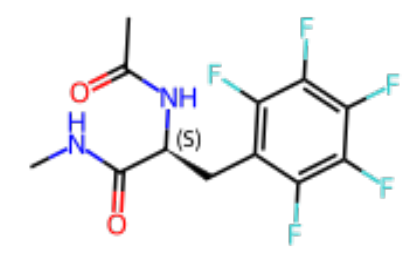
19	NAP1	CC(=O)N[C@@H](Cc1cccc(cccc2)c12)C(=O)NC	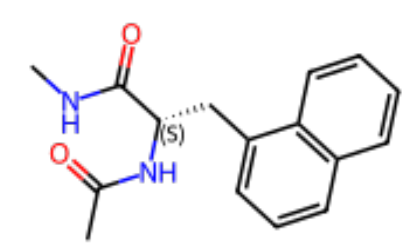
20	NAP2	CC(=O)N[C@@H](Cc1ccc(cccc2)c2c1)C(=O)NC	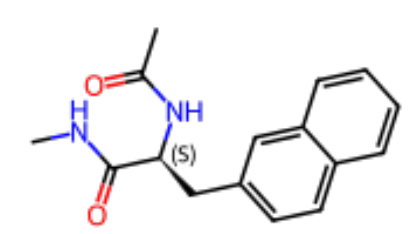
21	ANT9	CC(=O)N[C@@H](Cc1c(cccc2)c2cc(cccc2)c12)C(=O)NC	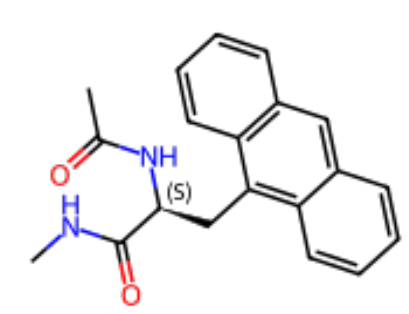
22	PYR3	CC(=O)N[C@@H](Cc1ccc(ccc3(ccc4))c(c3c4cc2)c12)C(=O)NC	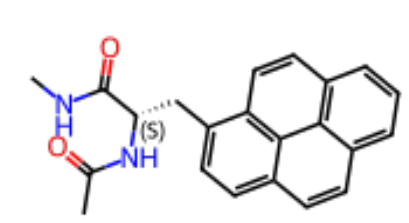
23	DHA	CC(=O)NC(=C)C(=O)NC	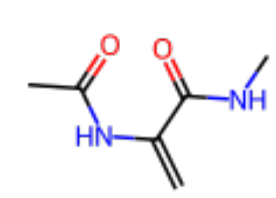

24	AIB	CC(=O)NC(C)(C)C(=O)NC	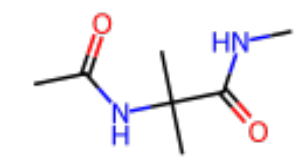
25	ACPC	CC(=O)NC(C1)(C1)C(=O)NC	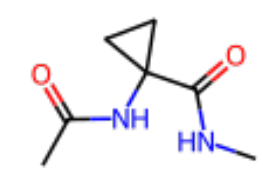
26	ACBC	CC(=O)NC(CC1)(C1)C(=O)NC	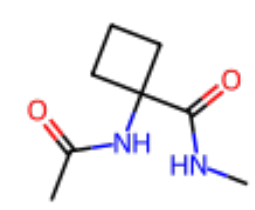
27	ACPenC	CC(=O)NC(CCC1)(C1)C(=O)NC	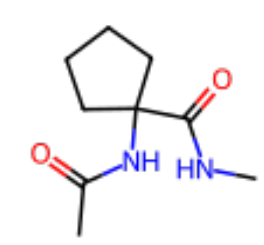
28	ACPhenC	CC(=O)NC(Cc(cccc2)c12)(C1)C(=O)NC	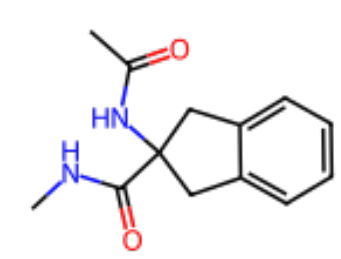
29	ACHC	CC(=O)NC(CCCC1)(C1)C(=O)NC	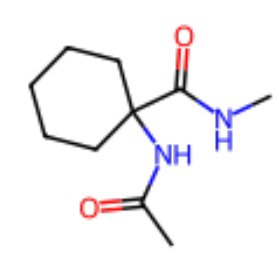
30	SAR	CC(=O)N(C)CC(=O)NC	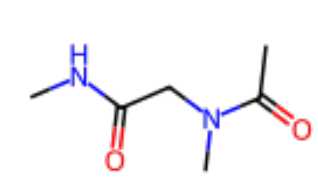

31	PTABU	CC(=O)N(CC)CC(=O)NC	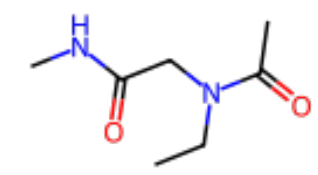
32	PTIPA	CC(=O)N(C(C)C)CC(=O)NC	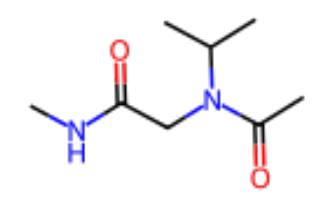
33	PTTBA	CC(=O)N(C(C)(C)C)CC(=O)NC	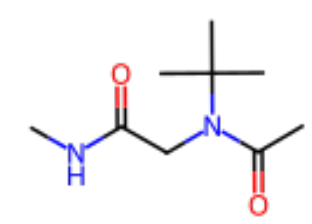
34	PTANI	CC(=O)N(c1ccccc1)CC(=O)NC	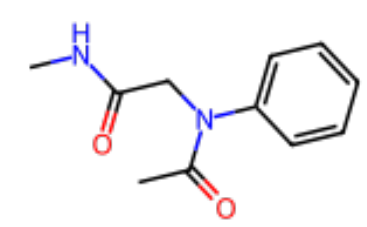
35	PTAMBA	CC(=O)N([C@H](c1ccccc1)C)CC(=O)NC	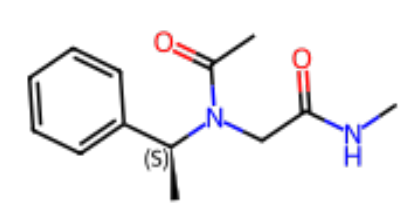
36	NMALA	CC(=O)N(C)[C@@H](C)C(=O)NC	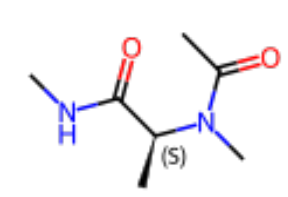
37	NMABU	CC(=O)N(C)[C@@H](CC)C(=O)NC	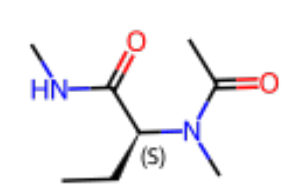

38	NMVAL	CC(=O)N(C)[C@@H](C(C)C)C(=O)NC	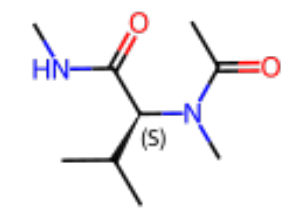
39	NMPHG	CC(=O)N(C)[C@@H](c1ccccc1)C(=O)NC	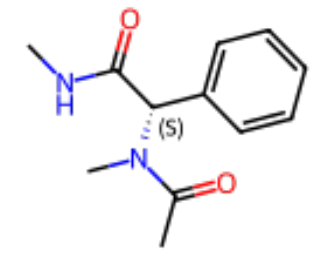
40	NMLEU	CC(=O)N(C)[C@@H](CC(C)C)C(=O)NC	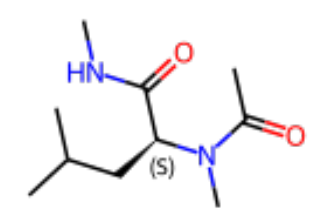
41	NMPHE	CC(=O)N(C)[C@@H](Cc1ccccc1)C(=O)NC	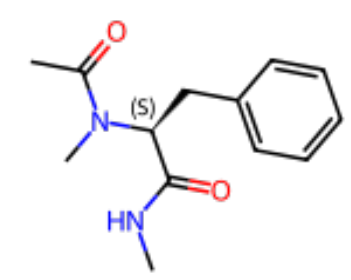
42	NMSER	CC(=O)N(C)[C@@H](C[OH])C(=O)NC	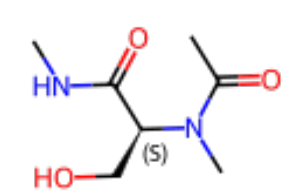
43	NMASN	CC(=O)N(C)[C@@H](CC(=O)N)C(=O)NC	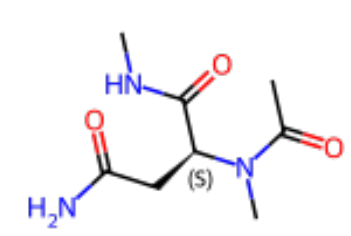
44	AZE	CC(=O)N1[C@@H](CC1)C(=O)NC	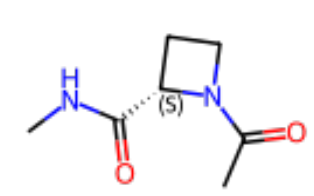

45	PRO	CC(=O)N1[C@@H](CCC1)C(=O)NC	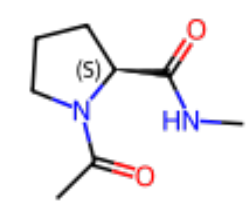
46	PIP	CC(=O)N1[C@@H](CCCC1)C(=O)NC	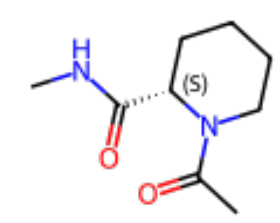
47	TIQ	CC(=O)N1[C@@H](c(cccc2)c2CC1)C(=O)NC	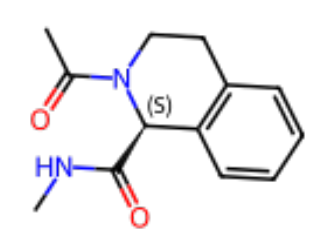
48	TIC	CC(=O)N1[C@@H](Cc(cccc2)c2C1)C(=O)NC	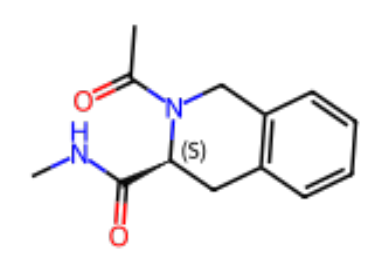
49	NHM	CC(=O)N1[C@@H](Cc2c3ccccc3[nH]c2C1)C(=O)NC	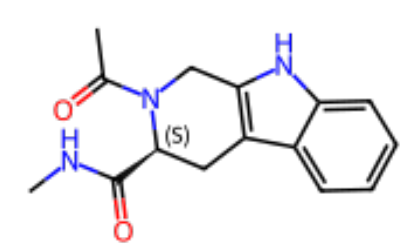
50	THP	CC(=O)N1[C@@H](CSC1)C(=O)NC	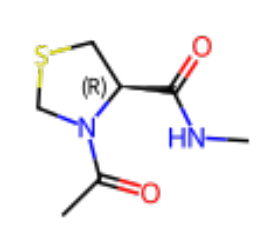
51	DHP	CC(=O)N1[C@@H](C=CC1)C(=O)NC	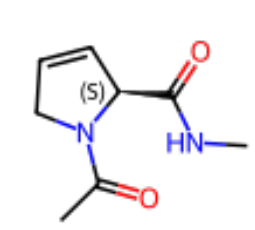

52	FPS	CC(=O)N1[C@@H](C[C@H](F)C1)C(=O)NC	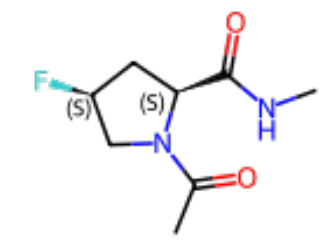
53	FPR	CC(=O)N1[C@@H](C[C@@H](F)C1)C(=O)NC	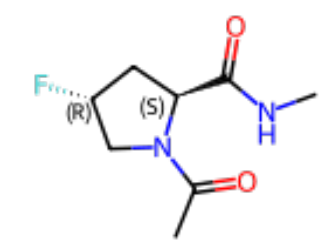
54	HPS	CC(=O)N1[C@@H](C[C@H](O)C1)C(=O)NC	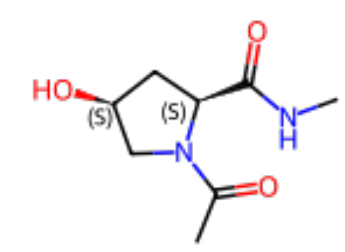
55	HPR	CC(=O)N1[C@@H](C[C@@H](O)C1)C(=O)NC	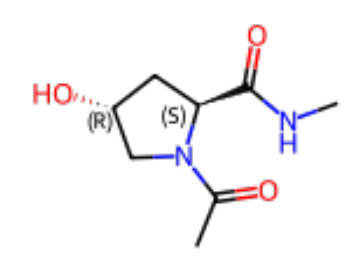
56	OXO	CC(=O)N1[C@@H](CCC1(=O))C(=O)NC	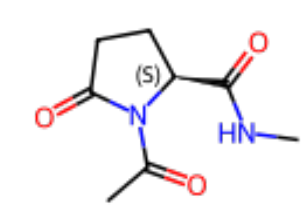
57	PPS	CC(=O)N1[C@@H](C[C@@H](c2ccccc2)C1)C(=O)NC	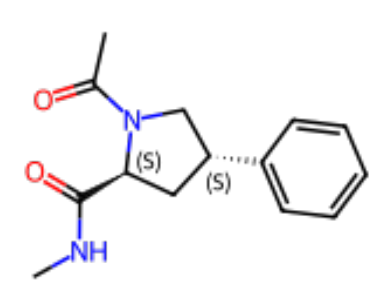
58	PPR	CC(=O)N1[C@@H](C[C@H](c2ccccc2)C1)C(=O)NC	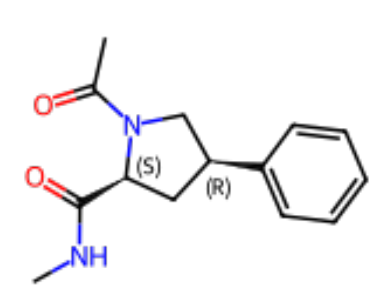

59	AMP	CC(=O)N1[C@@](C)(CCC1)C(=O)NC	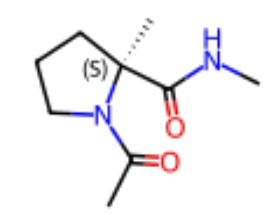
60	IDC	CC(=O)N1[C@@H](Cc(cccc2)c12)C(=O)NC	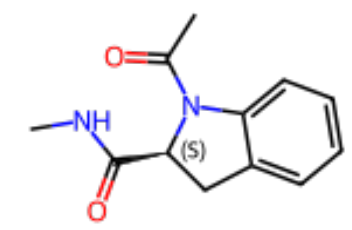
61	B3ALA	CC(=O)N[C@@H](C)CC(=O)NC	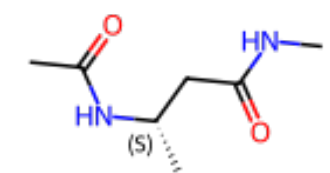
62	B3VAL	CC(=O)N[C@@H](C(C)C)CC(=O)NC	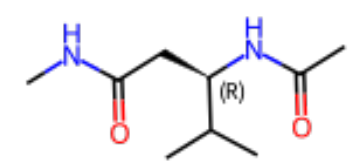
63	B3PHG	CC(=O)N[C@@H](c1ccccc1)CC(=O)NC	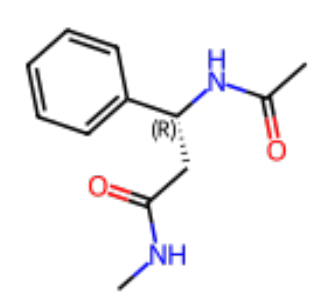
64	B3LEU	CC(=O)N[C@@H](CC(C)C)CC(=O)NC	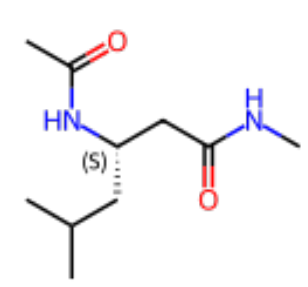
65	B3PHE	CC(=O)N[C@@H](Cc1ccccc1)CC(=O)NC	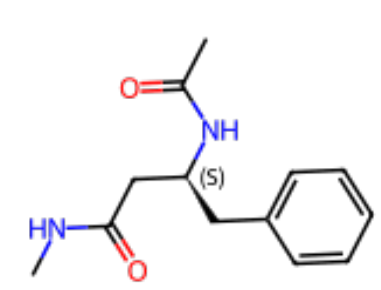

66	B2ALA	CC(=O)NC[C@@H](C)C(=O)NC	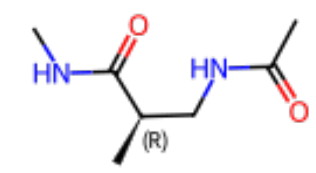
67	BAZE	CC(=O)N1CC(C1)C(=O)NC	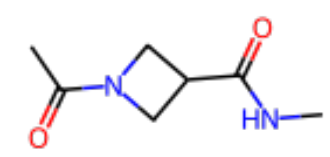
68	BPRO	CC(=O)N1[C@@H](CCC1)CC(=O)NC	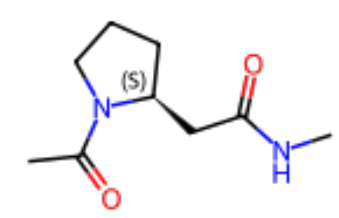
69	BPIP	CC(=O)N1[C@@H](CCCC1)CC(=O)NC	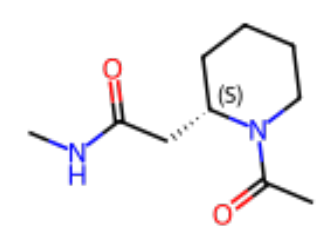
70	PRR	CC(=O)N1C[C@@H](CC1)C(=O)NC	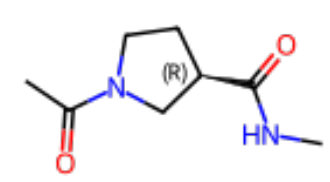
71	NIP	CC(=O)N1C[C@@H](CCC1)C(=O)NC	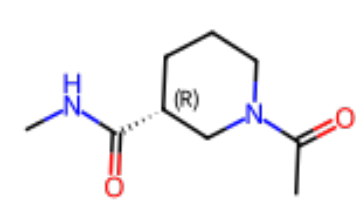
72	AGLS	CC(=O)N[C@@H](CC1)C(=O)N1[C@@H](C)C(=O)NC	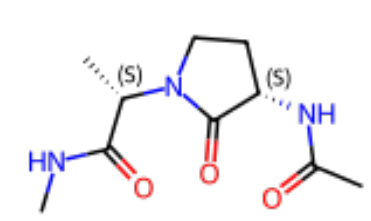

73	AGLR	CC(=O)N[C@@H](CC1)C(=O)N1[C@H](C)C(=O)NC	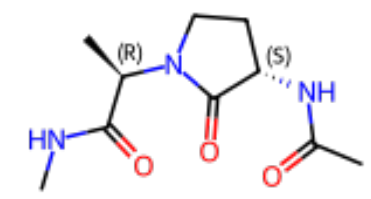
74	OZL	CC(=O)N[C@@H](C)c1nc(co1)C(=O)NC	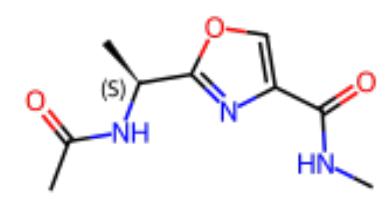
75	OZS	CC(=O)N[C@@H](C)C1=N[C@@H](CO1)C(=O)NC	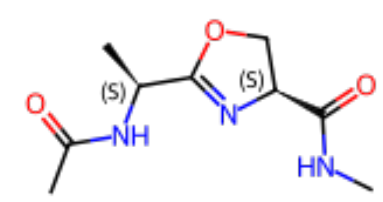
76	OZR	CC(=O)N[C@H](C)C1=N[C@@H](CO1)C(=O)NC	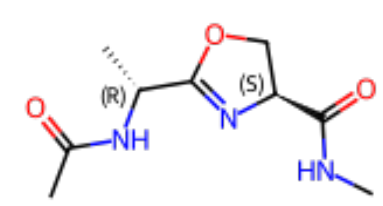
77	TZL	CC(=O)N[C@@H](C)c1nc(cs1)C(=O)NC	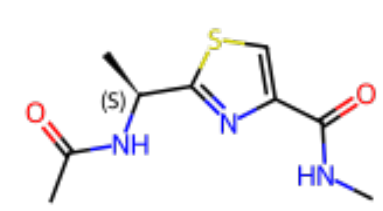
78	TZS	CC(=O)N[C@@H](C)C1=N[C@@H](CS1)C(=O)NC	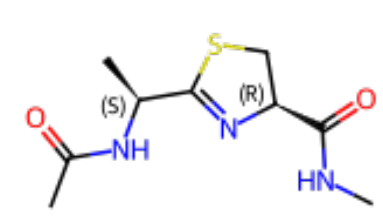
79	TZR	CC(=O)N[C@H](C)C1=N[C@@H](CS1)C(=O)NC	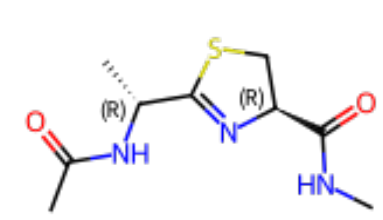

80	G4ALA	CC(=O)N[C@@H](C)CCC(=O)NC	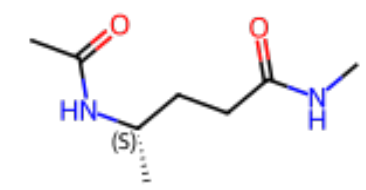
81	GPN	CC(=O)NCC1(CCCCC1)CC(=O)NC	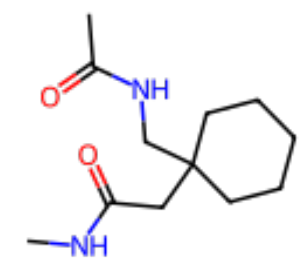
82	INIP	CC(=O)N1CCC(CC1)C(=O)NC	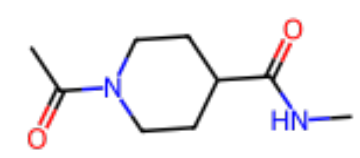
83	D4ALA	CC(=O)N[C@@H](C)CCCC(=O)NC	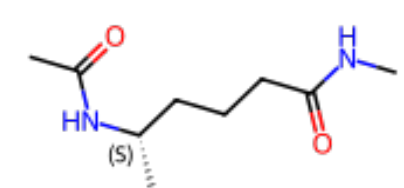
84	LARD	CC(=O)N[C@H](C(=O)N)CC(=O)NC	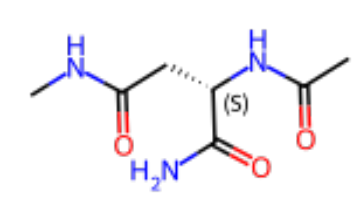
85	LARE	CC(=O)N[C@H](C(=O)N)CCC(=O)NC	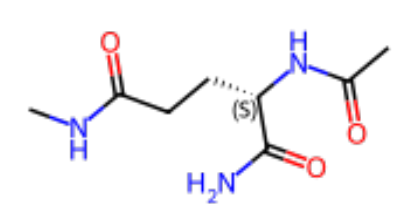
86	SUGA	CC(=O)N[C@H](C(=O)N)CSCC(=O)NC	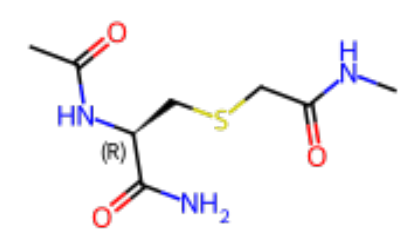

87	ACPC12C	CC(=O)N[C@@H]1[C@@H](C1)C(=O)NC	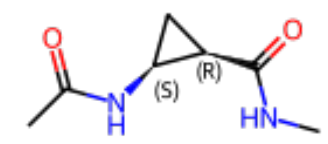
88	ACPC12T	CC(=O)N[C@@H]1[C@H](C1)C(=O)NC	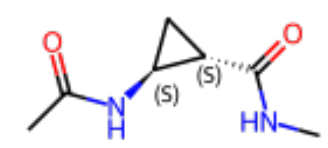
89	ACBC12C	CC(=O)N[C@@H]1[C@@H](CC1)C(=O)NC	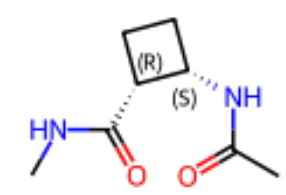
90	ACBC12T	CC(=O)N[C@@H]1[C@H](CC1)C(=O)NC	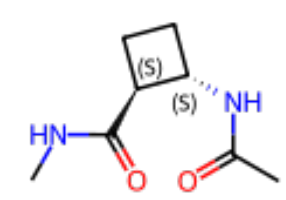
91	ACBC13C	CC(=O)N[C@@H]1C[C@@H](C1)C(=O)NC	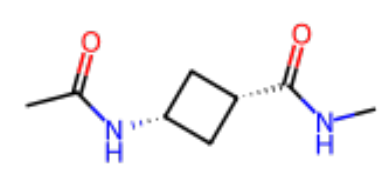
92	ACBC13T	CC(=O)N[C@@H]1C[C@H](C1)C(=O)NC	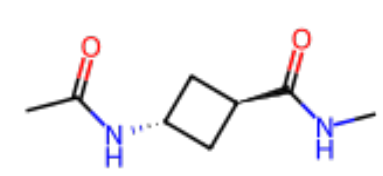
93	ACPenC12C	CC(=O)N[C@@H]1[C@@H](CCC1)C(=O)NC	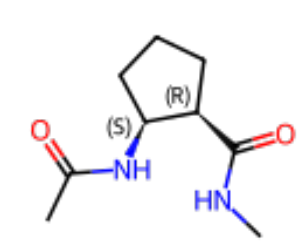

94	ACPenC12T	CC(=O)N[C@@H]1[C@H](CCC1)C(=O)NC	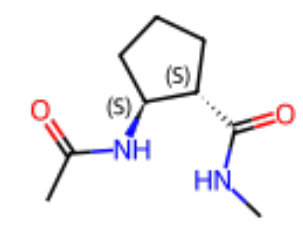
95	ACPenC13C	CC(=O)N[C@@H]1C[C@@H](CC1)C(=O)NC	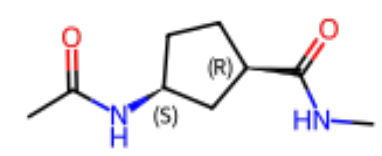
96	ACPeneC13C	CC(=O)N[C@@H]1C[C@@H](C=C1)C(=O)NC	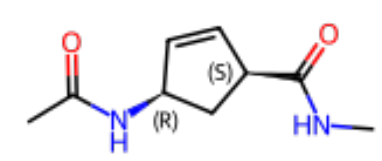
97	ACPenC13T	CC(=O)N[C@@H]1C[C@H](CC1)C(=O)NC	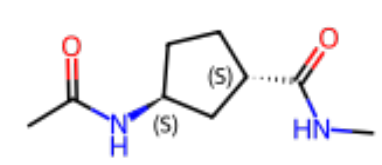
98	GAPC	CC(=O)N[C@H]1C[C@H](N(C(=O)C)C1)C(=O)NC	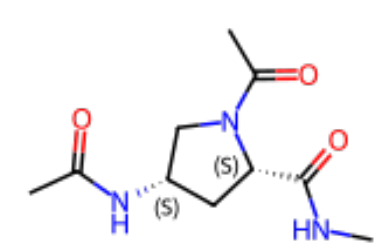
99	GAPT	CC(=O)N[C@@H]1C[C@H](N(C(=O)C)C1)C(=O)NC	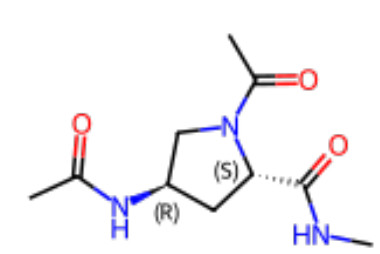
100	ACHC12C	CC(=O)N[C@@H]1[C@@H](CCCC1)C(=O)NC	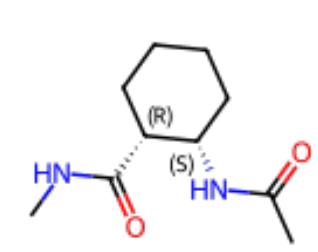

101	ACHC12T	CC(=O)N[C@@H]1[C@H](CCCC1)C(=O)NC	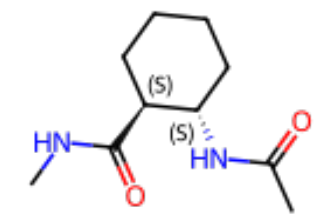
102	ACHC13C	CC(=O)N[C@@H]1C[C@@H](CCC1)C(=O)NC	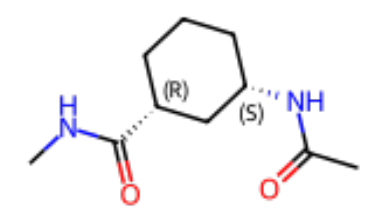
103	ACHC13T	CC(=O)N[C@@H]1C[C@H](CCC1)C(=O)NC	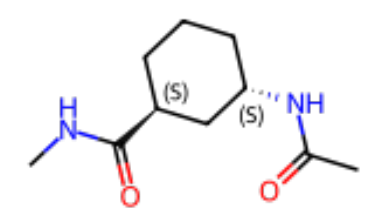
104	ACHC14C	CC(=O)N[C@@H]1CC[C@@H](CC1)C(=O)NC	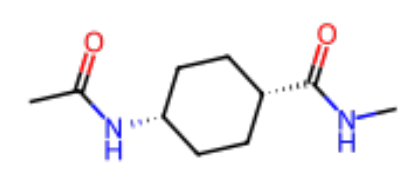
105	ACHC14T	CC(=O)N[C@@H]1CC[C@H](CC1)C(=O)NC	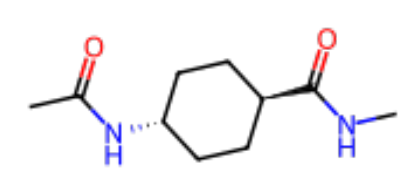
106	ACHA14C	CC(=O)N[C@@H]1CC[C@@H](CC1)CC(=O)NC	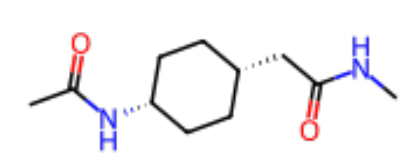
107	ACHA14T	CC(=O)N[C@@H]1CC[C@H](CC1)CC(=O)NC	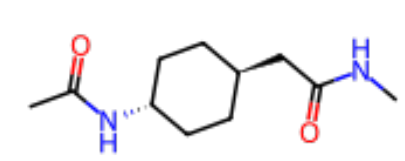

108	AMC14C	CC(=O)NC[C@@H]1CC[C@@H](CC1)C(=O)NC	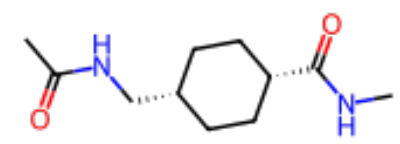
109	AMC14T	CC(=O)NC[C@@H]1CC[C@H](CC1)C(=O)NC	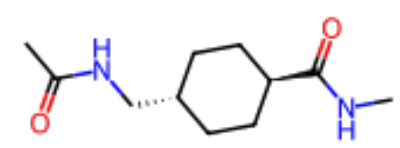
110	BEN2	CC(=O)Nc1c(cccc1)C(=O)NC	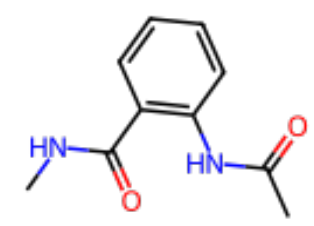
111	BEN3	CC(=O)Nc1cc(ccc1)C(=O)NC	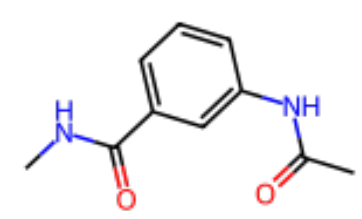
112	BEN4	CC(=O)Nc1ccc(cc1)C(=O)NC	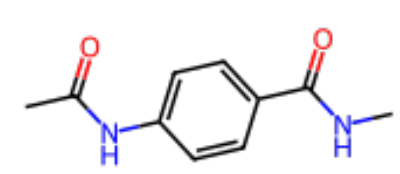
113	AMBEN2	CC(=O)NCc1c(cccc1)C(=O)NC	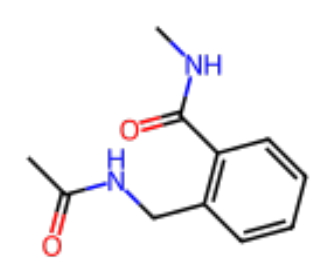
114	AMBEN3	CC(=O)NCc1cc(ccc1)C(=O)NC	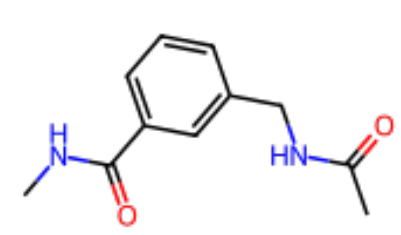

115	AMBEN4	CC(=O)NCc1ccc(cc1)C(=O)NC	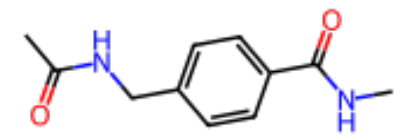
116	ACBEN2	CC(=O)Nc1c(cccc1)CC(=O)NC	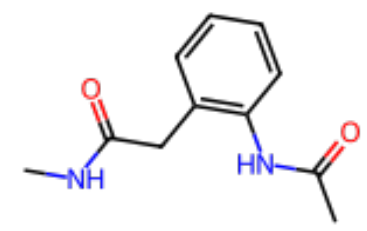
117	ACBEN3	CC(=O)Nc1cc(ccc1)CC(=O)NC	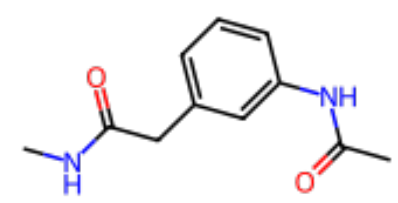
118	ACBEN4	CC(=O)Nc1ccc(cc1)CC(=O)NC	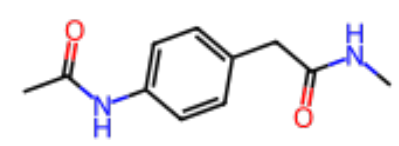
119	AMACBEN2	CC(=O)NCc1c(cccc1)CC(=O)NC	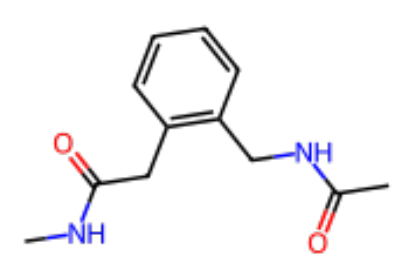
120	AMACBEN3	CC(=O)NCc1cc(ccc1)CC(=O)NC	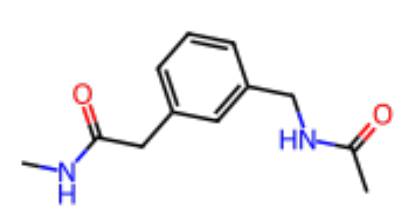
121	AMACBEN4	CC(=O)NCc1ccc(cc1)CC(=O)NC	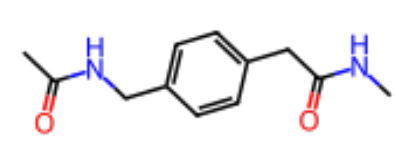

122	NMBEN2	CC(=O)N(C)c1c(cccc1)C(=O)NC	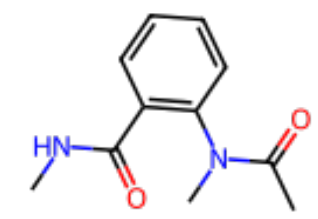
123	NMBEN3	CC(=O)N(C)c1cc(ccc1)C(=O)NC	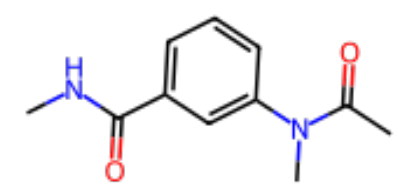
124	NMBEN4	CC(=O)N(C)c1ccc(cc1)C(=O)NC	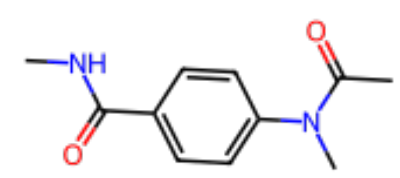
125	HUCP	CC(=O)NCc1nc(ccc1)C(=O)NC	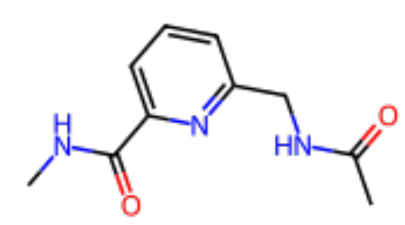
126	HUCQ	CC(=O)Nc1c2nc(ccc2ccc1)C(=O)NC	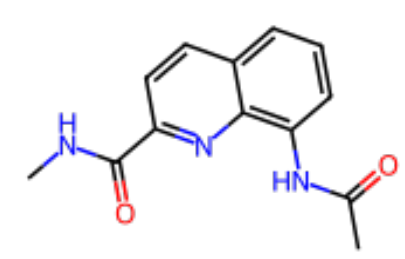
127	CLICK	CC(=O)NCc1cn(nn1)CC(=O)NC	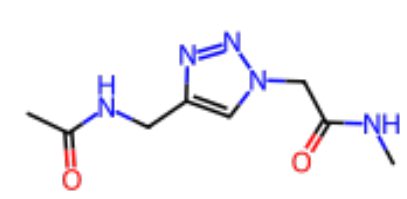
128	CLICKS	CC(=O)NCc1cn(nn1)[C@@H](C)C(=O)NC	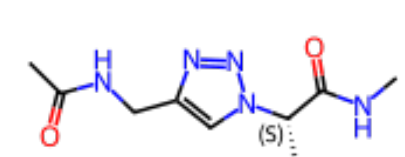

129	VIC	CC(=O)N[C@@H](CCNC(=O)CC1)C(=O)N[C@H]1C(=O)NC	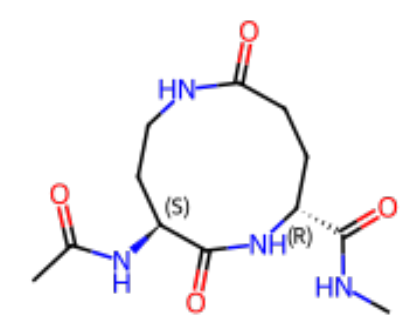
130	ABOC	CC(=O)NC(CC1)(CCC1C2)[C@H]2C(=O)NC	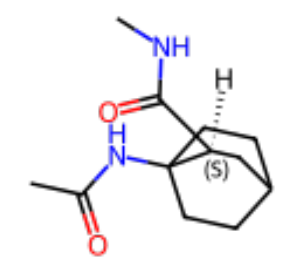
131	mALA	CC(=O)N[C@H](C)C(=O)NC	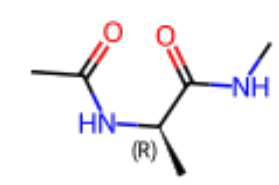
132	mABU	CC(=O)N[C@H](CC)C(=O)NC	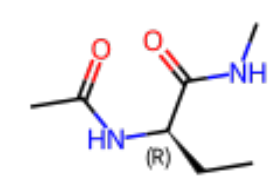
133	mSER	CC(=O)N[C@H](C[OH])C(=O)NC	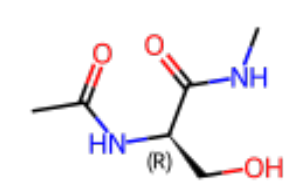
134	mASN	CC(=O)N[C@H](CC(=O)N)C(=O)NC	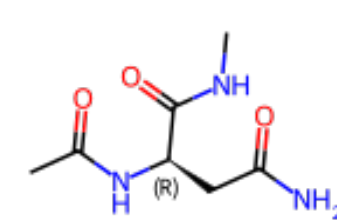
135	mCLA	CC(=O)N[C@H](C[Cl])C(=O)NC	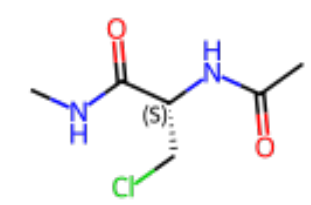

136	mVAL	CC(=O)N[C@H](C(C)C)C(=O)NC	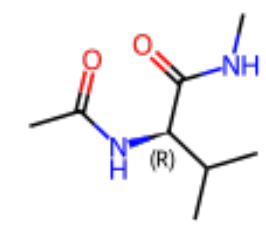
137	mTBG	CC(=O)N[C@H](C(C)(C)C)C(=O)NC	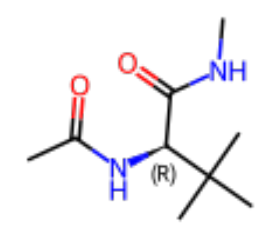
138	mNVL	CC(=O)N[C@H](CCC)C(=O)NC	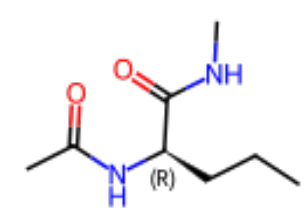
139	mLEU	CC(=O)N[C@H](CC(C)C)C(=O)NC	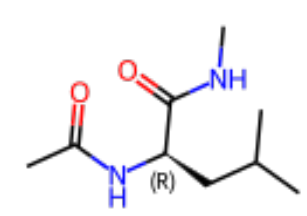
140	mTBA	CC(=O)N[C@H](CC(C)(C)C)C(=O)NC	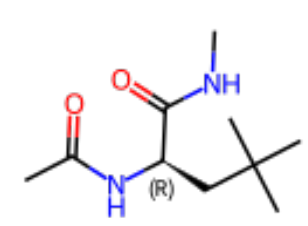
141	mPHG	CC(=O)N[C@H](c1ccccc1)C(=O)NC	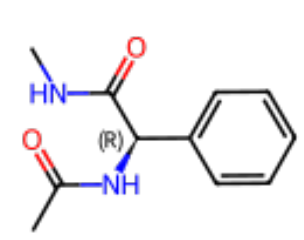
142	mPHE	CC(=O)N[C@H](Cc1ccccc1)C(=O)NC	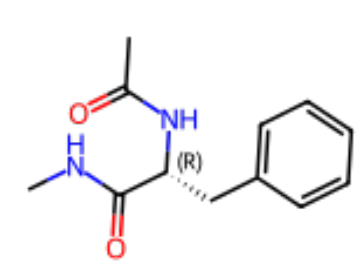

143	mFPA	CC(=O)N[C@H](Cc1c(F)c(F)c(F)c(F)c1(F))C(=O)NC	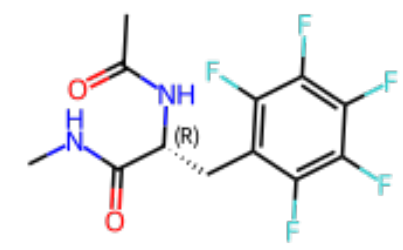
144	mNAP1	CC(=O)N[C@H](Cc1cccc(cccc2)c12)C(=O)NC	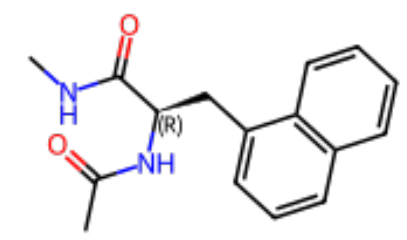
145	mNAP2	CC(=O)N[C@H](Cc1ccc(cccc2)c2c1)C(=O)NC	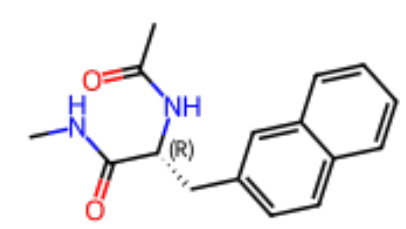
146	mANT9	CC(=O)N[C@H](Cc1c(cccc2)c2cc(cccc2)c12)C(=O)NC	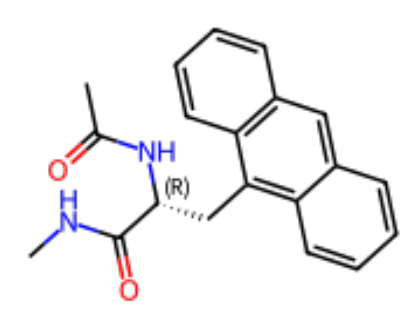
147	mPYR3	CC(=O)N[C@H](Cc1ccc(ccc3(ccc4))c(c3c4cc2)c12)C(=O)NC	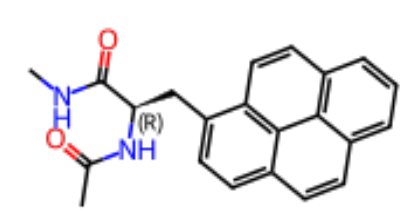
148	mPTAMBA	CC(=O)N([C@@H](c1ccccc1)C)CC(=O)NC	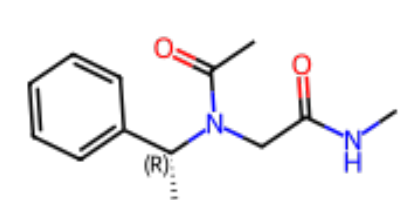
149	mNMALA	CC(=O)N(C)[C@H](C)C(=O)NC	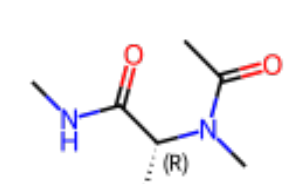

150	mNMABU	CC(=O)N(C)[C@H](CC)C(=O)NC	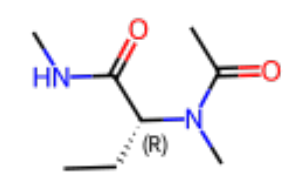
151	mNMVAL	CC(=O)N(C)[C@H](C(C)C)C(=O)NC	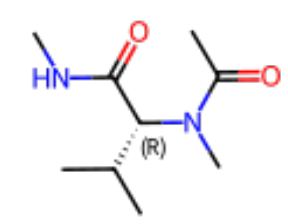
152	mNMPHG	CC(=O)N(C)[C@H](c1ccccc1)C(=O)NC	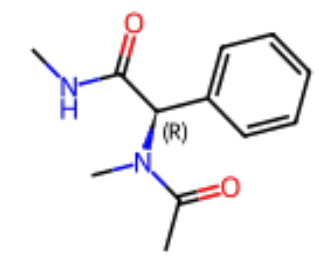
153	mNMLEU	CC(=O)N(C)[C@H](CC(C)C)C(=O)NC	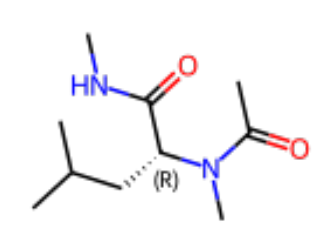
154	mNMPHE	CC(=O)N(C)[C@H](Cc1ccccc1)C(=O)NC	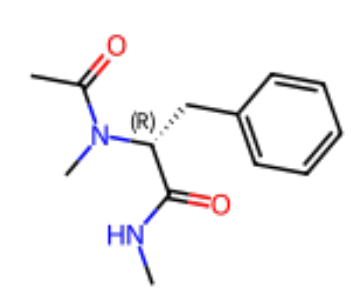
155	mNMSER	CC(=O)N(C)[C@H](C[OH])C(=O)NC	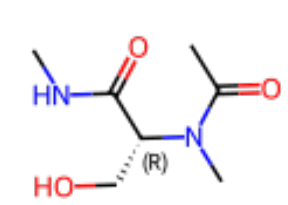
156	mNMASN	CC(=O)N(C)[C@H](CC(=O)N)C(=O)NC	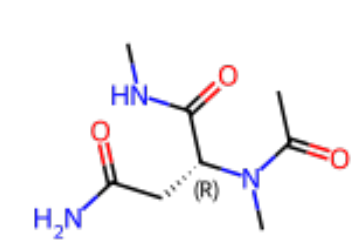

157	mAZE	CC(=O)N1[C@H](CC1)C(=O)NC	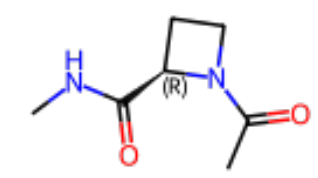
158	mPRO	CC(=O)N1[C@H](CCC1)C(=O)NC	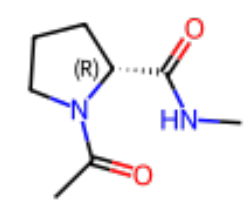
159	mPIP	CC(=O)N1[C@H](CCCC1)C(=O)NC	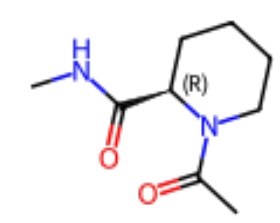
160	mTIQ	CC(=O)N1[C@H](c(cccc2)c2CC1)C(=O)NC	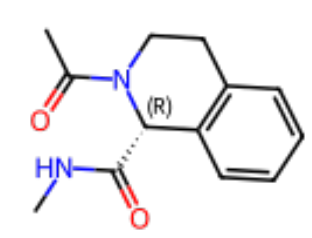
161	mTIC	CC(=O)N1[C@H](Cc(cccc2)c2C1)C(=O)NC	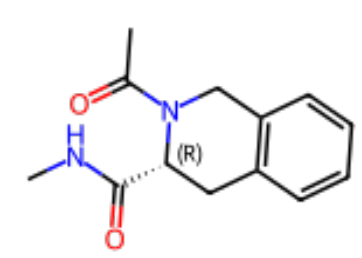
162	mNHM	CC(=O)N1[C@H](Cc2c3ccccc3[nH]c2C1)C(=O)NC	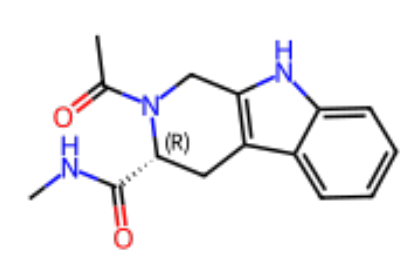
163	mTHP	CC(=O)N1[C@H](CSC1)C(=O)NC	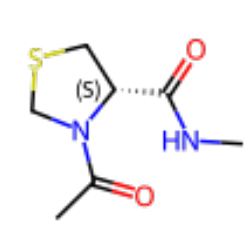

164	mDHP	CC(=O)N1[C@H](C=CC1)C(=O)NC	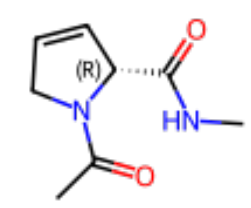
165	mFPS	CC(=O)N1[C@H](C[C@@H](F)C1)C(=O)NC	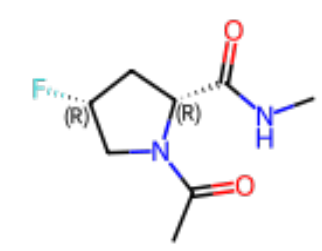
166	mFPR	CC(=O)N1[C@H](C[C@H](F)C1)C(=O)NC	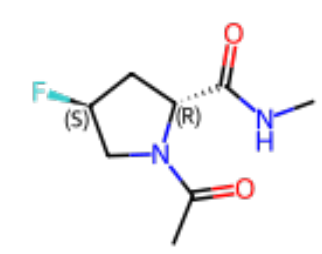
167	mHPS	CC(=O)N1[C@H](C[C@@H](O)C1)C(=O)NC	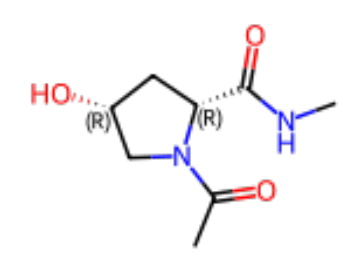
168	mHPR	CC(=O)N1[C@H](C[C@H](O)C1)C(=O)NC	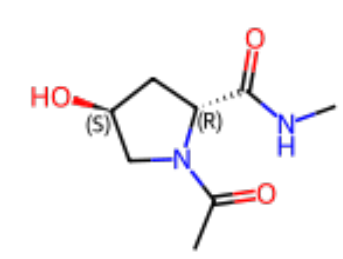
169	mOXO	CC(=O)N1[C@H](CCC1(=O))C(=O)NC	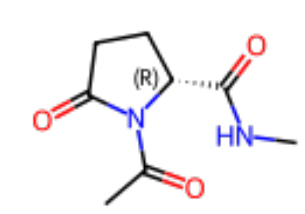
170	mPPS	CC(=O)N1[C@H](C[C@H](c2ccccc2)C1)C(=O)NC	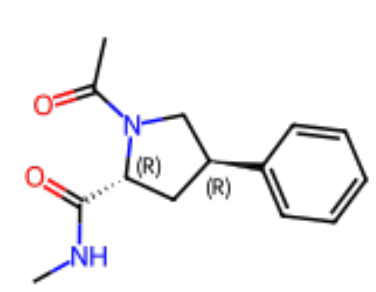

171	mPPR	CC(=O)N1[C@H](C[C@@H](c2ccccc2)C1)C(=O)NC	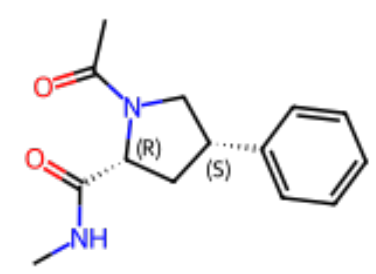
172	mAMP	CC(=O)N1[C@](C)(CCC1)C(=O)NC	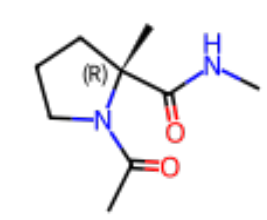
173	mIDC	CC(=O)N1[C@H](Cc(cccc2)c12)C(=O)NC	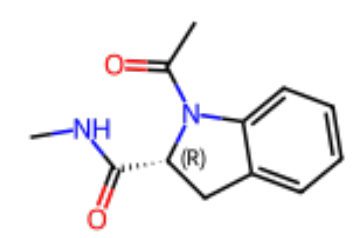
174	mB3ALA	CC(=O)N[C@H](C)CC(=O)NC	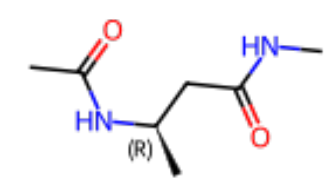
175	mB3VAL	CC(=O)N[C@H](C(C)C)CC(=O)NC	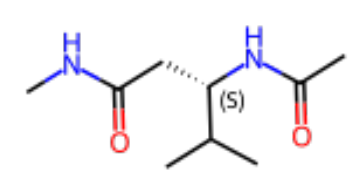
176	mB3PHG	CC(=O)N[C@H](c1ccccc1)CC(=O)NC	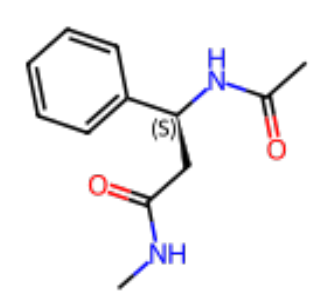
177	mB3LEU	CC(=O)N[C@H](CC(C)C)CC(=O)NC	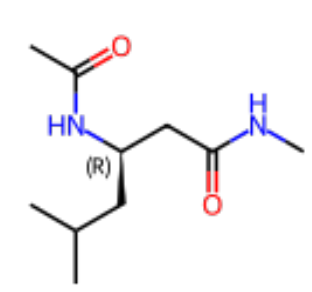

178	mB3PHE	CC(=O)N[C@H](Cc1ccccc1)CC(=O)NC	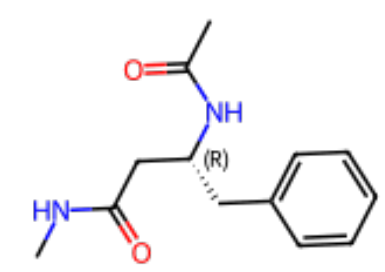
179	mB2ALA	CC(=O)NC[C@H](C)C(=O)NC	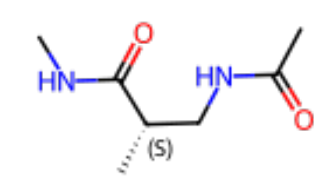
180	mBPRO	CC(=O)N1[C@H](CCC1)CC(=O)NC	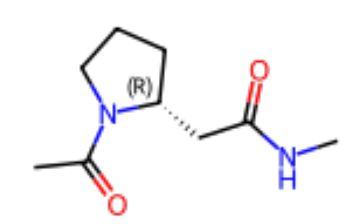
181	mBPIP	CC(=O)N1[C@H](CCCC1)CC(=O)NC	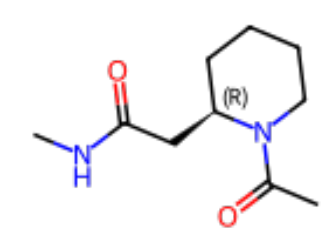
182	mPRR	CC(=O)N1C[C@H](CC1)C(=O)NC	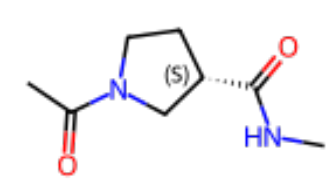
183	mNIP	CC(=O)N1C[C@H](CCC1)C(=O)NC	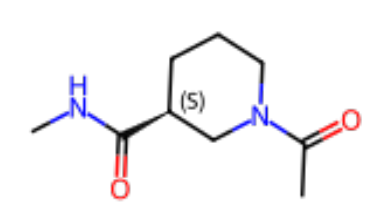
184	mAGLS	CC(=O)N[C@H](CC1)C(=O)N1[C@H](C)C(=O)NC	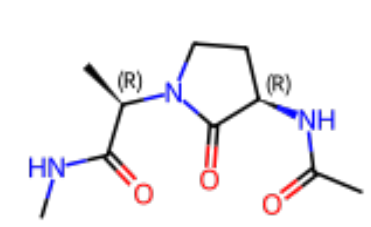

185	mAGLR	CC(=O)N[C@H](CC1)C(=O)N1[C@@H](C)C(=O)NC	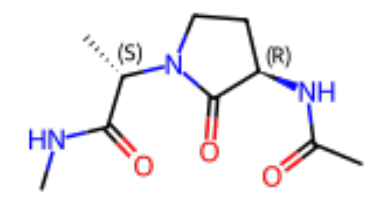
186	mOZL	CC(=O)N[C@H](C)c1nc(co1)C(=O)NC	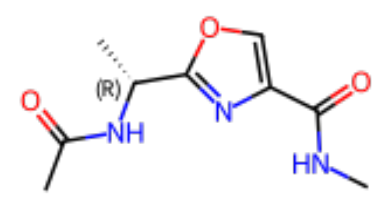
187	mOZS	CC(=O)N[C@H](C)C1=N[C@H](CO1)C(=O)NC	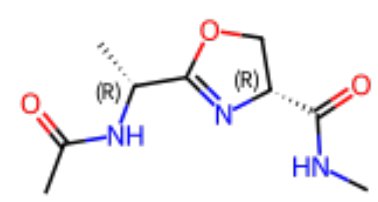
188	mOZR	CC(=O)N[C@@H](C)C1=N[C@H](CO1)C(=O)NC	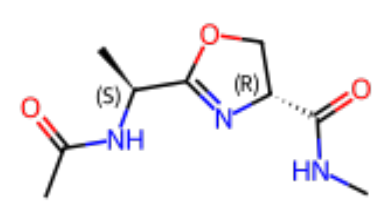
189	mTZL	CC(=O)N[C@H](C)c1nc(cs1)C(=O)NC	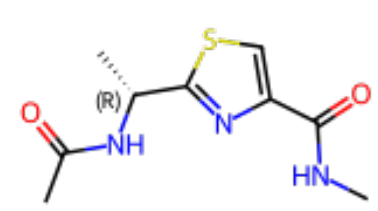
190	mTZS	CC(=O)N[C@H](C)C1=N[C@H](CS1)C(=O)NC	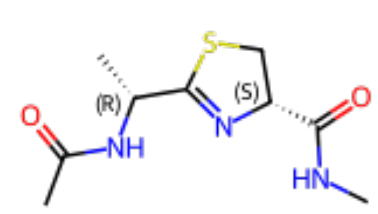
191	mTZR	CC(=O)N[C@@H](C)C1=N[C@H](CS1)C(=O)NC	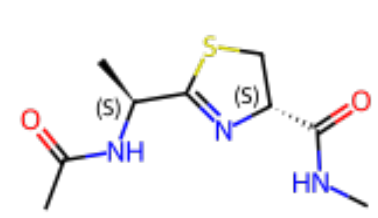

192	mG4ALA	CC(=O)N[C@H](C)CCC(=O)NC	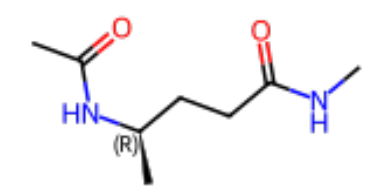
193	mD4ALA	CC(=O)N[C@H](C)CCCC(=O)NC	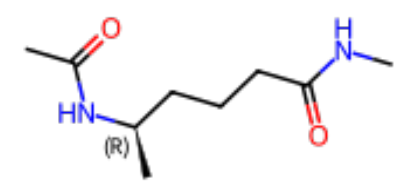
194	mLARD	CC(=O)N[C@@H](C(=O)N)CC(=O)NC	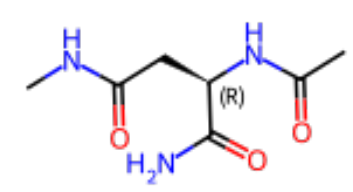
195	mLARE	CC(=O)N[C@@H](C(=O)N)CCC(=O)NC	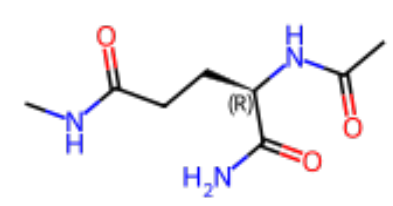
196	mSUGA	CC(=O)N[C@@H](C(=O)N)CSCC(=O)NC	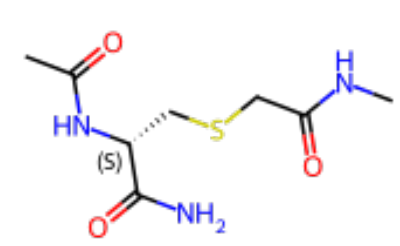
197	mACPC12C	CC(=O)N[C@H]1[C@H](C1)C(=O)NC	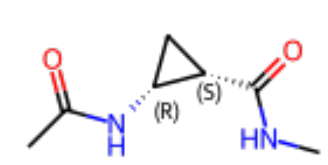
198	mACPC12T	CC(=O)N[C@H]1[C@@H](C1)C(=O)NC	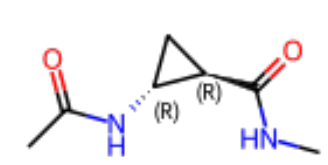

199	mACBC12C	CC(=O)N[C@H]1[C@H](CC1)C(=O)NC	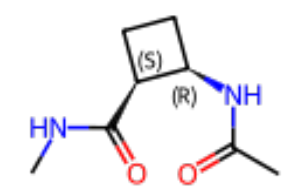
200	mACBC12T	CC(=O)N[C@H]1[C@@H](CC1)C(=O)NC	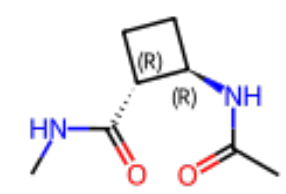
201	mACBC13C	CC(=O)N[C@H]1C[C@H](C1)C(=O)NC	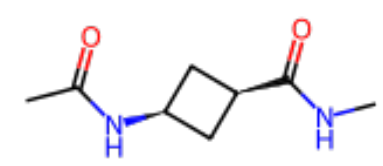
202	mACBC13T	CC(=O)N[C@H]1C[C@@H](C1)C(=O)NC	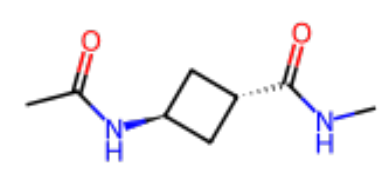
203	mACPenC12C	CC(=O)N[C@H]1[C@H](CCC1)C(=O)NC	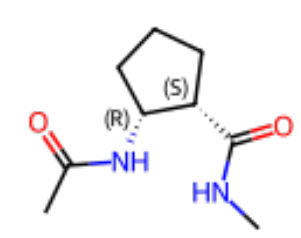
204	mACPenC12T	CC(=O)N[C@H]1[C@@H](CCC1)C(=O)NC	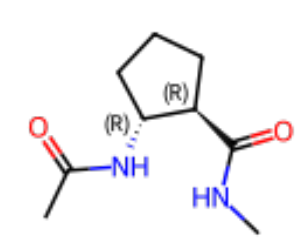
205	mACPenC13C	CC(=O)N[C@H]1C[C@H](CC1)C(=O)NC	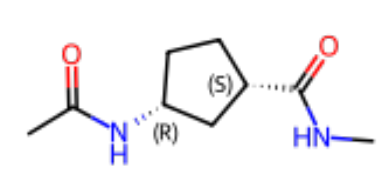

206	mACPeneC13C	CC(=O)N[C@H]1C[C@H](C=C1)C(=O)NC	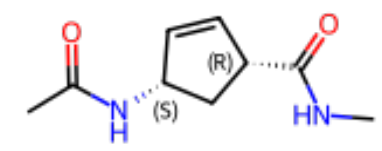
207	mACPenC13T	CC(=O)N[C@H]1C[C@@H](CC1)C(=O)NC	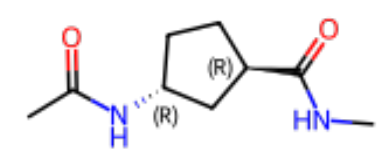
208	mGAPC	CC(=O)N[C@@H]1C[C@@H](N(C(=O)C)C1)C(=O)NC	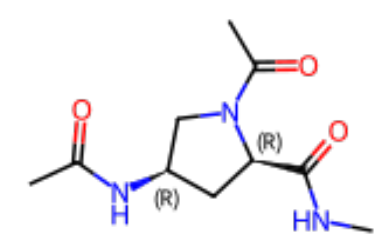
209	mGAPT	CC(=O)N[C@H]1C[C@@H](N(C(=O)C)C1)C(=O)NC	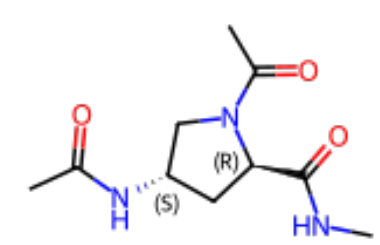
210	mACHC12C	CC(=O)N[C@H]1[C@H](CCCC1)C(=O)NC	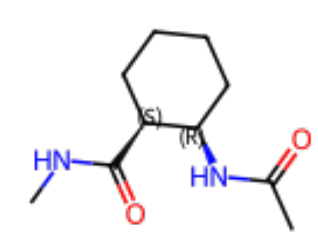
211	mACHC12T	CC(=O)N[C@H]1[C@@H](CCCC1)C(=O)NC	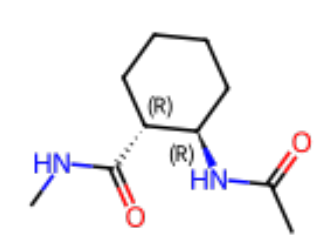
212	mACHC13C	CC(=O)N[C@H]1C[C@H](CCC1)C(=O)NC	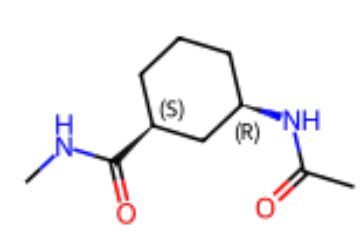

213	mACHC13T	CC(=O)N[C@H]1C[C@@H](CCC1)C(=O)NC	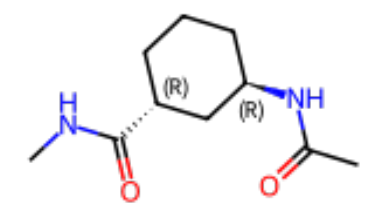
214	mACHC14C	CC(=O)N[C@H]1CC[C@H](CC1)C(=O)NC	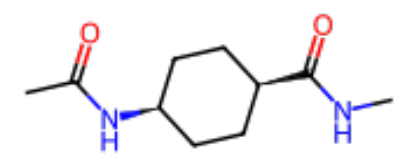
215	mACHC14T	CC(=O)N[C@H]1CC[C@@H](CC1)C(=O)NC	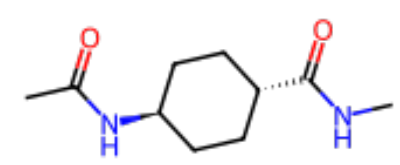
216	mACHA14C	CC(=O)N[C@H]1CC[C@H](CC1)CC(=O)NC	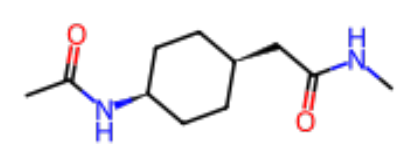
217	mACHA14T	CC(=O)N[C@H]1CC[C@@H](CC1)CC(=O)NC	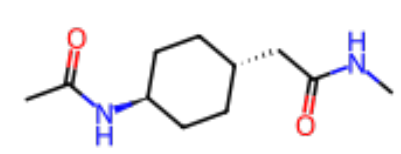
218	mAMC14C	CC(=O)NC[C@H]1CC[C@H](CC1)C(=O)NC	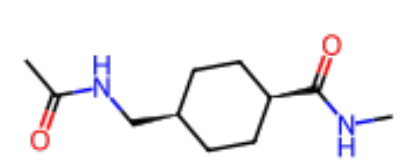
219	mAMC14T	CC(=O)NC[C@H]1CC[C@@H](CC1)C(=O)NC	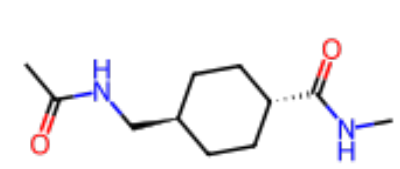

220	mCLICKS	CC(=O)NCc1cn(nn1)[C@H](C)C(=O)NC	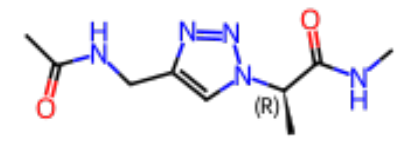
221	mVIC	CC(=O)N[C@H](CCNC(=O)CC1)C(=O)N[C@@H]1C(=O)NC	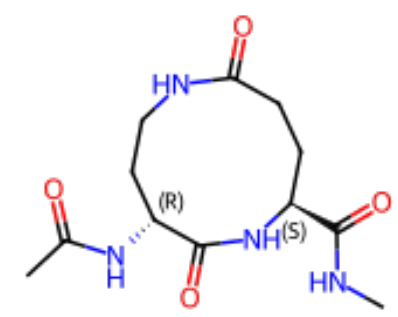
222	mABOC	CC(=O)NC(CC1)(CCC1C2)[C@@H]2C(=O)NC	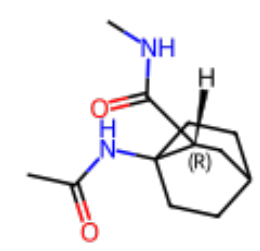

Table S2. Helical parameters for DPR5 - DPR10 models

Helices	**DPR5**	**DPR6**	**DPR7**	**DPR8**	**DPR9**	**DPR10**
amino acid[a] sequence $(AB)_n$	ALA B2ALA	ALA HPR	OZL INIP	GLN FPR	AZE PRO	AMBEN2 PRO
chirality A	S	S	S	S	S	achiral
chirality B	S	R, R	achiral	S, S	R	S
amino acid types A,B	α, β	α, α	δ, γ	α, α	α, α	δ, α
helix handedness	right	left	right	left	right	right
# residues per turn (RPT)	4.5	4.2	4.2	5.9	3.1	4.6
radius	~4 Å	~3 Å	~5 Å	~0 Å	~1 Å	~3 Å
pitch (rise per turn)	5.1 Å	6.8 Å	5 Å	13.3 Å	7.1 Å	5 Å
rotation angle per repeat (twist)[b]	160°	170°	170°	120°	235°	155°
hydrogen bond acceptor	B2ALA CO (*i*)	HPR sc OH (*i*)	INIP CO (*i*)	GLN sc CO (*i*)	NA	AMBEN2 CO (*i*)
hydrogen bond donor	ALA NH (*i* +3)	ALA NH (*i* -3)	OZL HN (*i* +5)	GLN NH (i+2)	NA	AMBEN2 NH (*i* -2)
2nd hbond acceptor	ALA CO (*i*)			FPR CO (*i*)		
2nd hbond donor	B2ALA HN (*i* -3)			GLN sc NH2 (*i* -1)		
# atoms in ring formed by hbond	16/18	14	25	10/10	none	17
peptide bond conformations ω A-B / B-A	trans / trans	trans / trans	trans / trans	trans / trans	trans / cis	trans / trans

[a]Amino acids are named as in the SMILES residue list (Table S1).
[b]The rotation angle per repeating dipeptide unit (twist) is calculated as 2 * 360 / RPT, where RPT is the number of residues per turn, and is rounded to the nearest 5°.

Table S3 - Dihedral angle parameters[a] for ideal models DPR1 - DPR10

Helix	3_{10}	α	π	DPR1[b]		DPR2		DPR3		DPR4	
(A B)$_n$	(A)$_n$	(A)$_n$	(A)$_n$	A	B	A	B	A	B	A	B
sequence	any	any	any	TYR	PIP	AMPA	PRO	ALA	NIP	ACHC	NIP
chirality	L	L	L	*S*	*R*	-	*S*	*S*	*S*	*S,R*	*S*
ϕ	-50°	-60°	-60°	-70°	110°	-130°	-60°	-120°	-100°	-150°	-100°
θ1	-	-	-	-	-	80°	-	-	180°	60°	180°
θ2	-	-	-	-	-	0°	-	-	-	-	-
θ3	-	-	-	-	-	70°	-	-	-	-	-
ψ	-30°	-50°	-70°	120°	-30°	-160°	-40°	120°	-150°	90°	-110°
(*i*, *i*-1) ω	180°	180°	180°	170°	180°	180°	180°	180°	-30°	180°	-30°

Helix	DPR5		DPR6		DPR7		DPR8		DPR9		DPR10	
(A B)$_n$	A	B	A	B	A	B	A	B	A	B	A	B
sequence	ALA	B2ALA	ALA	HPR	OZL	INIP	GLN	FPR	AZE	PRO	AMBEN2	PRO
chirality	*S*	*S*	*S*	*R,R*	*S*	-	*S*	*S,S*	*S*	*R*	-	*S*
ϕ	-120°	90°	-120°	70°	-60°	-100°	-120°	-80°	50°	-60°	-80°	-70°
θ1	-	70°	-	-	-40°	-60°	-	-	-	-	-70°	-
θ2	-	-	-	-	180°	180°	-	-	-	-	180°	-
θ3	-	-	-	-	180°	-	-	-	-	-	180°	-
ψ	130°	-140°	80°	-120°	30°	150°	110°	-10°	-150°	130°	-130°	-30°
(*i*, *i*-1) ω	180°	180°	180°	180°	170°	180°	180°	-170°	10°	180°	180°	180°

[a]All dihedral angles are rounded to the nearest 10°.

[b]These parameters are for the DPR1 model with L-Tyr, D-Pip repeats. The X-ray structure of the D-Tyr, L-Pip enantiomer had ϕ, ψ values of 60°, -138° and -95°, 29°, respectively, and a left-handed helix.

Table S4. X-ray data collection and refinement statistics

	DPR1
Wavelength	0.72932
Resolution range	21.47 - 0.86 Å (0.88 - 0.86 Å)
Space group	$P2_1$
Unit cell	13.89, 11.05, 22.56<br>90, 107.92, 90
Reflections collected	31943
Unique reflections	5333
Multiplicity	5.98
Completeness<br>Theta(max)= 24.953	94.3%
Mean I/sigma(I)	1.8
R-merge	0.309
Empirical formula	$C_{47} H_{61} N_8 O_{10}$ 19(O)
Formula weight	1202.04
Temperature	100 K
Volume	3294.6
Z	2
Density (calculated) g/cm^3	1.212
F(000)	1262.0
Absorption coefficient mm^{-1}	0.107
Refinement method	Full-matrix least-squares on F^2
Data / restraints / parameters	5333/ 759/ 196
Goodness-of-fit on F^2	1.184
Final R indices (all data)	0.2275
Largest diff. peak and hole	0.47 and -0.56 e.Å^{-3}

Table S5. DPR1 NOE summary table

NOESY data for 1x, 3x, and 5x DPR1 at 298 K (and 5x at 283 K). Key supporting NOEs that were not overlapped by intraresidue peaks are tabulated along with their distances determined from the model structures with NOE sum averaging used for Tyr Hβs, Hδs, and Hεs ($r_{sum}^{-6} = (r_1^{-6}) + (r_2^{-6})$). We identified nine sequential (i+1) NOEs, including five between the Tyr aromatic ring (*i*) and Pip side chain (*i*+1) protons in the 5x sample. Six of these NOEs were either weak or absent in the 3x polymer, confirming the proximity of these side chains in the 5x polymer. Additionally, four non-overlapping medium-range (*i*+3) NOEs, characteristic of this helical structure, further support the model. These include NOEs between the Pip Hβ3 and Hγ3 to the Tyr H^N that support the presence of *i*, *i*+3 hydrogen bonds. Most of these *i*+1 and *i*+3 NOEs became stronger upon cooling from 298 to 283 K.

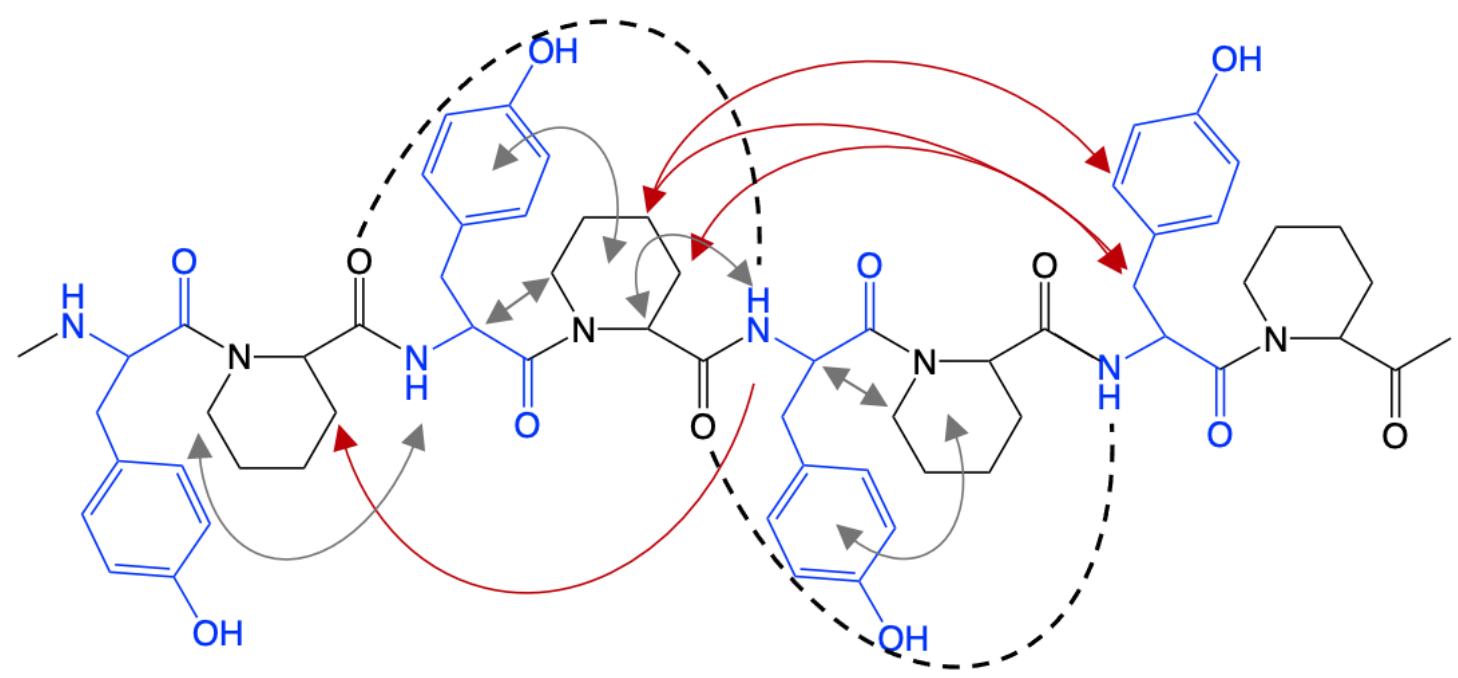

DPR1 NOE summary			NOE	sum ave.				
#	residue *i*	residue *n*	type	dist (Å)	1x	3x	5x	5x at 298 vs 283 K
1	Pip Hα	Tyr HN	*i* +1	3.0	NA	2nd and 3rd Tyr	yes	
2	Pip Hε2	Tyr HN	*i* +1	3.5	NA	3rd Tyr only	yes	stronger at 283K
3	Hε3	Tyr HN	*i* +1	2.5	NA	3rd Tyr only	yes	stronger at 283K
4	Tyr Hα	Pip Hε2	*i* +1	1.7	yes	yes	yes	confirms trans Pip
5		Pip Hε3	*i* +1	3.1	weak	OL	yes	
6	Tyr Hδs	Pip Hα	*i* +1	4.2	no	weak	yes	stronger at 283K
		Pip Hβ2	*i* +1	3.4	no	weak	OL	
7		Pip Hδ2	*i* +1	3.2	no	weak	yes	stronger at 283K
8		Pip Hε2	*i* +1	3.2	weak	weak	yes	stronger at 283K
9	Tyr Hεs	Pip Hβ2	*i* +1	3.8	no	no	yes	
10		Pip Hδ2	*i* +1	3.1	no	no	yes	stronger at 283K
		Pip Hε2	*i* +1	4.2	no	no	no	
11	Pip Hβ3	Tyr HN	*i* +3	3.9	NA	no	weak	stronger at 283K
	Pip Hγ3		*i* +3	2.8	NA	no	OL	
12	Pip Hγ2	Tyr Hδs	*i* +3	3.4	NA	weak	yes	stronger at 283K
	Pip Hγ3		*i* +3	3.8	NA	no	OL Hβs	
13	Pip Hβ3	Tyr Hβ2/3	*i* +3	2.6	NA	no	yes	stronger at 283K
	Pip Hγ2		*i* +3	2.4	NA	no	OL	
14	Pip Hγ3		*i* +3	1.9	NA	OL	weak	

Table S6. DPR1 ^3J(HN-Hα) scalar coupling constants at 298 and 283 K.

Tyr residues in 5x repeat are not assigned, except for the most N-terminal Tyr-1. The presence of small Tyr ^3J(HN-Hα) coupling constants in DPR1 5x supports the repeating type II β-turn structural motif. Smaller ^3J coupling constants at lower temperatures correspond to lower values of Tyr backbone dihedral angle φ, consistent with an increased population of type II β turns. The ^3J values in the 5x sample at 283 K (10 °C) were 4.4, 3.8, and 3.2 Hz and were notably smaller than those at 25 °C and significantly smaller than in the 1x unstructured control sample (8.4 Hz at 25 °C). The X-ray crystal structure has D-Tyr φ values of 58°, 60°, and 48° for the three repeats, corresponding to ^3J(HN-Hα) values of 2.5 -3, 3 - 3.5, and 2 - 2.5 Hz, respectively, as determined by the Karplus equation.[23] The observation of smaller ^3J(HN-Hα) coupling constants for longer-length DPR1 polymers and lower temperatures indicate a shift in population-average values towards those expected for the design model. Fraying at the N-terminus is indicated by the larger ^3J(HN-Hα) for Tyr-1 in 5x DPR1 at both temperatures. OL means that the amide protons of Tyr were overlapped, and the ^3J scalar coupling constant could not be measured.

DPR1		3J(HN-Hα) (Hz)	
**length**	**HN**	**298 K**	**283 K**
1x (*cis*)	Tyr-1	OL	5.9
1x (*trans*)	Tyr-1	8.4	OL
3x	Tyr-1	5.7	5.7
	Tyr-3	OL	6.8
	Tyr-5	4.6	4.6
5x	Tyr-1	4.8	4.4
	Tyr-NA	4.1	3.8
	Tyr-NA	OL	OL
	Tyr-NA	OL	OL
	Tyr-NA	3.7	3.2

Table S7. DPR1 amide temperature coefficients

Temperature coefficients (ΔδHN/ΔT) for Tyr HNs in 1x and 5x DPR1 were obtained by analyzing ^{1}H NMR spectra collected at different temperatures. They are determined by fitting the slope of chemical shift vs temperature for each non-overlapping amide proton. In 5x DPR1, these could only be measured for Tyr-1 and two other 'not-assigned' (NA) Tyr HNs. Typically, amide protons that are hydrogen bonded to solvent have large downfield shifts as temperatures are lowered, yielding large negative temperature coefficients, and amide protons that are in intramolecular hydrogen bonds are typically shifted less by temperature. In standard cases, coefficients more positive than -4.0 ppb/K are taken as evidence for intramolecular hydrogen bonds, e.g., a coefficient of -2 ppb/K indicates strong hydrogen bonding and 0 ppb/K even stronger. Contrary to expectations, only one of the coefficients in helical 5x DPR1 (-2.5 ppb/K) was more positive than in the 1x (trans conformation, -3.3 ppb/K). However, this standard interpretation is unreliable when there are significant population-weighted chemical shift changes with temperature.[24] Increasing the population of ordered helical conformations with enhanced hydrogen bonds at lower temperatures would lead to more downfield average shifts and more negative ΔδHN/ΔT values counteracting the positive coefficient effect. For 5x DPR1, CD, and other NMR analyses support this shift to more ordered conformations, and therefore, information about hydrogen bond strength cannot be interpreted in a simple manner from the temperature coefficients. Additionally, the variability among the three different temperature coefficients measured for 5x DPR1 shows that there are non-uniform influencing factors across the length of the polymer, which was also supported by other NMR evidence.

DPR1 1x	1x trans	1x cis	DPR1 5x		5x (all trans)	
H^N chemical shift (ppm)				H^N chemical shift (ppm)		
temp(K)	Tyr-1	Tyr-1	temp (K)	Tyr-1	Tyr-NA	Tyr-NA
288		7.102	283	8.086	7.810	7.710
298	6.643		293	8.004	7.764	7.690
308	6.605	6.985	303	7.920	7.715	7.660
318	6.570	6.930	313	7.833	OL	7.636
328	6.550	6.890	323	7.744	OL	OL
338	6.504	6.822	333	7.658	OL	OL
	-3.3	-5.5		-8.6	-4.7	-2.5
	$\Delta\delta H^N/\Delta T$ (ppb/K)			$\Delta\delta H^N/\Delta T$ (ppb/K)		

Table S8. DPR2 NOE summary

NOE summary for 1x, 3x, and 10x DPR2 at 298 K (and for 10x at 283 K). Schematic of DPR2 with selected intraresidue H^N-Hα2/3 TOCSY/NOESY correlations (gray arrows), *i*+2 and *i*+3 NOE peaks (red arrows), and hydrogen bond (black dashed line) shown (top). The NOE distances were measured from the model, and the sum average (r_{sum}) distance to methylene protons was calculated using R^{-6} averaging of the distances to each of the protons, where $r_{sum}^{-6} = (r_1^{-6}) + (r_2^{-6})$. For each *i*+2 predicted NOE with distances < 4 Å, the corresponding intraresidue distances are given. There are four NOEs identified in the 10x NOESY spectrum. Three are from the $Hα_2$s of AMPA (*i*) to protons in the AMPA (*i*+2), including the H^N and unassigned ring protons 5H-8H. There are also NOEs from these ring protons to Pro Hβs that are attributed to the *i*+3 NOE. These NOEs are stronger at the lower temperature (283 K). Many NOE cross-peaks from the H^Ns to shifted Hα2/3 that are not in the TOCSY (and therefore not from the same AMPA residue) can be observed and are attributed to NOEs to Hα2 and Hα3 of AMPA (*i*-2). Overlapped (OL) NOEs that could not be unambiguously assigned are marked.

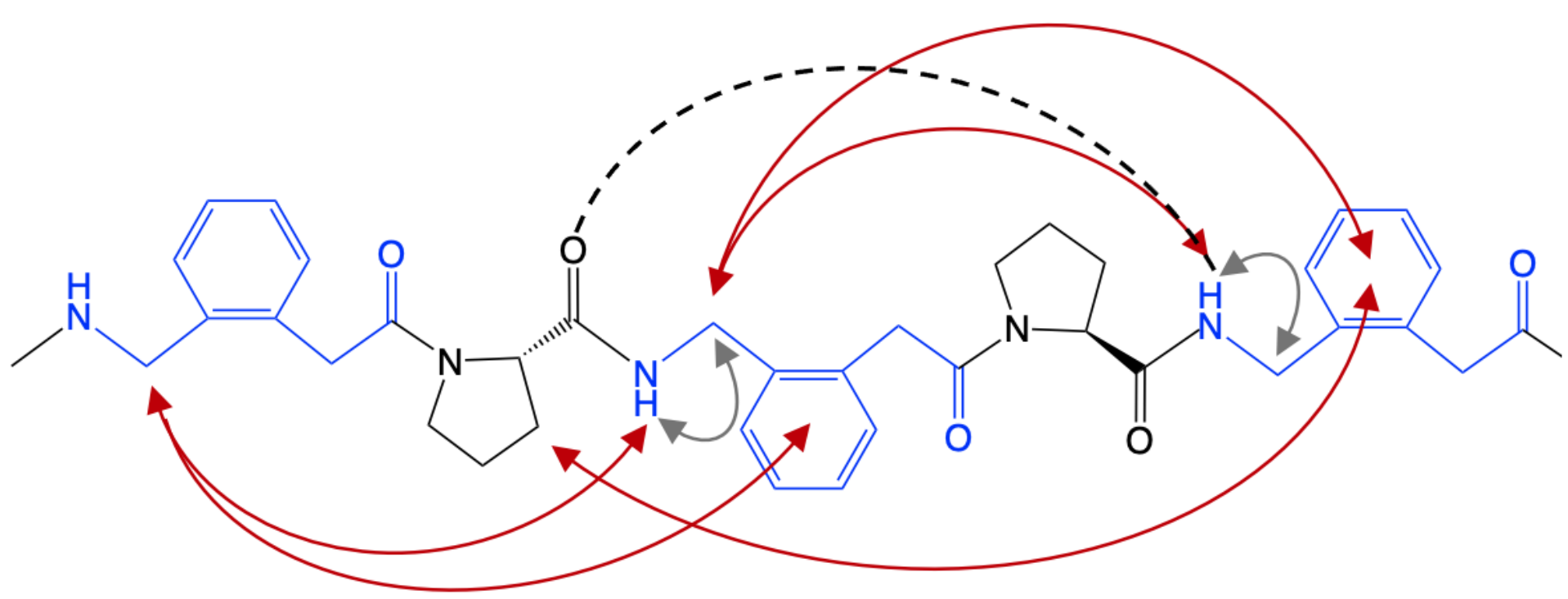

DPR2 NOE SUMMARY			NOE	sum ave.				
**#**	**residue *i***	**residue *n***	**type**	**dist (Å)**	**1x**	**3x**	**10x**	**10x 298 vs 283 K**
	AMPA Hα2/3	HN	intra	2.6	yes	yes	yes	
1	AMPA Hα2	AAB HN	*i*+2	2.6	NA	no	yes	stronger at 283 K
2	AMPA Hα3	AAB HN	*i*+2	2.6	NA	no	yes	stronger at 283 K
	AMPA Hα2/3	5H	intra	4.7	no	OL	yes	
3	AMPA Hα2/3	AAB 5H	*i*+2	3.6	NA	OL	yes (5H or 6H)	stronger at 283 K
	AMPA Hα2/3	6H	intra	5.5	no	OL	-	
3	AMPA Hα2/3	AAB 6H	*i*+2	3.6	NA	OL	yes (5H or 6H)	stronger at 283 K
	AMPA Hδ2/3	HN	intra	3.3	yes	yes	yes	
	AMPA Hδ2/3	AAB HN	*i*+2	3.6	NA	OL	OL	not observed
4	PRO HBs	AAB 7H	*i*+3	3.7	NA	no	yes	stronger at 283 K
	PRO HBs	AAB 8H	*i*+3	3.7	NA	no	yes	stronger at 283 K

Table S9. DPR2 amide temperature coefficients

We determined the temperature coefficients (ΔδHN/ΔT) for the AMPA HNs by analyzing ^{1}H NMR spectra of 1x DPR2 and the 2D ^{1}H-^{1}H TOCSY of 10x DPR2, focusing on the eight strongest and least-overlapped peaks in the HN region at different temperatures. We found that only half of the temperature coefficients in 10x DPR2 were more positive than in the 1x trans dipeptide. Our interpretation is that DPR2, like DPR1, also has a significant influence from population-weighted chemical shift changes at lower temperatures. These temperature-dependent changes in equilibrium made it so that the ΔδHN/ΔT values could not be used to provide evidence for enhanced hydrogen bonding in the 10x using the conventional interpretation. Also, the shift in equilibrium towards a more ordered helical structure was supported by temperature-dependent CD and NMR NOESY data, as was the case for DPR1.

DPR2	1x *cis*	1x *trans*			10x DPR2	10x *trans*				
**temp (K) **	**AAB NH**	**AAB NH**	**AAB NH**	**AAB NH**	**AAB NH**	**AAB NH**	**AAB NH**	**AAB NH**	**AAB NH**	**AAB NH**
273			8.55	8.26	8.20	8.18	8.02	7.67	7.61	7.38
283	7.47	7.26	8.45	8.22	8.17	8.13	7.96	7.65	7.59	7.36
288	7.42									
298	7.34	7.18	8.28	8.11	8.07	8.02	7.88	7.62	7.57	7.35
313		7.09	8.10	7.95	7.91	7.85	7.79	7.61	7.56	7.33
318		7.05								
**ΔδHN/ΔT**	**-8.1**	**-5.9**	**-11.4**	**-7.8**	**-7.4**	**-8.2**	**-5.7**	**-1.5**	**-1.2**	**-1.2**

Table S10. HPLC and Mass Spectrometry validation

Name	N-Term	Res1	Res2	Repeats	C-Term	Exp Mass	Obs Mass
DPR1-1x	ACE	TYR	mPIP	1	NH2	333.4	317.0+356.1+ 689.2
DPR1-3x	GLY	mTYR	PIP	3	NH2	897.0	897.5
DPR1-5x	ACE	TYR	mPIP	5	NH2	1430.7	1452.9+1431. 9+707.7
DPR2-1x	ACE	AMACBEN2	PRO	1	NH2	303.4	304.1
DPR2-3x	GLY	AMACBEN2	PRO	3	NH2	807.0	807.6
DPR2-10x	ACE	AMACBEN2	PRO	10	NH2	2502.0	1251.9+835.0+ 624.4
DPR3-1x	ACE	ALA	mNIP	1	NH2	241.3	227.1+242.1
DPR3-4x	GLY	ALA	mNIP	4	NH2	803.0	803.5
DPR3-10x	ACE	ALA	mNIP	10	NH2	1881.3	1881.3+941.3+ 627.9
DPR4-1x	ACE	ACHC12C	mNIP	1	NH2	295.4	296.2
DPR4-3x	GLY	ACHC12C	mNIP	3	NH2	783.0	783.4
DPR4-10x	ACE	ACHC12C	mNIP	10	NH2	2422.2	1211.6+808.5 +606.6

Name	Smiles
DPR1-1x	CC(=O)N[C@@H](Cc1ccc(O)cc1)C(=O)N1[C@H](CCCC1)C(=O)N
DPR1-3x	NCC(=O)N[C@H](Cc1ccc(O)cc1)C(=O)N1[C@@H](CCCC1)C(=O)N[C@H](Cc1ccc(O)cc1)C(=O)N1[C@@H](CCCC1)C(=O)N[C@H](Cc1ccc(O)cc1)C(=O)N1[C@@H](CCCC1)C(=O)N
DPR1-5x	CC(=O)N[C@@H](Cc1ccc(O)cc1)C(=O)N1[C@H](CCCC1)C(=O)N[C@@H](Cc1ccc(O)cc1)C(=O)N1[C@H](CCCC1)C(=O)N[C@@H](Cc1ccc(O)cc1)C(=O)N1[C@H](CCCC1)C(=O)N[C@@H](Cc1ccc(O)cc1)C(=O)N1[C@H](CCCC1)C(=O)N[C@@H](Cc1ccc(O)cc1)C(=O)N1[C@H](CCCC1)C(=O)N
DPR2-1x	CC(=O)NCc1c(cccc1)CC(=O)N1[C@@H](CCC1)C(=O)N
DPR2-3x	NCC(=O)NCc1c(cccc1)CC(=O)N1[C@@H](CCC1)C(=O)NCc1c(cccc1)CC(=O)N1[C@@H](CCC1)C(=O)NCc1c(cccc1)CC(=O)N1[C@@H](CCC1)C(=O)N

DPR2-10x	CC(=O)NCc1c(cccc1)CC(=O)N1[C@@H](CCC1)C(=O)NCc1c(cccc1)CC(=O)N1[C@@H](CCC1)C(=O)NCc1c(cccc1)CC(=O)N1[C@@H](CCC1)C(=O)NCc1c(cccc1)CC(=O)N1[C@@H](CCC1)C(=O)NCc1c(cccc1)CC(=O)N1[C@@H](CCC1)C(=O)NCc1c(cccc1)CC(=O)N1[C@@H](CCC1)C(=O)NCc1c(cccc1)CC(=O)N1[C@@H](CCC1)C(=O)NCc1c(cccc1)CC(=O)N1[C@@H](CCC1)C(=O)NCc1c(cccc1)CC(=O)N1[C@@H](CCC1)C(=O)NCc1c(cccc1)CC(=O)N1[C@@H](CCC1)C(=O)N
DPR3-1x	CC(=O)N[C@@H](C)C(=O)N1C[C@H](CCC1)C(=O)N
DPR3-4x	NCC(=O)N[C@@H](C)C(=O)N1C[C@H](CCC1)C(=O)N[C@@H](C)C(=O)N1C[C@H](CCC1)C(=O)N[C@@H](C)C(=O)N1C[C@H](CCC1)C(=O)N[C@@H](C)C(=O)N1C[C@H](CCC1)C(=O)N
DPR3-10x	CC(=O)N[C@@H](C)C(=O)N1C[C@H](CCC1)C(=O)N[C@@H](C)C(=O)N1C[C@H](CCC1)C(=O)N[C@@H](C)C(=O)N1C[C@H](CCC1)C(=O)N[C@@H](C)C(=O)N1C[C@H](CCC1)C(=O)N[C@@H](C)C(=O)N1C[C@H](CCC1)C(=O)N[C@@H](C)C(=O)N1C[C@H](CCC1)C(=O)N[C@@H](C)C(=O)N1C[C@H](CCC1)C(=O)N[C@@H](C)C(=O)N1C[C@H](CCC1)C(=O)N[C@@H](C)C(=O)N1C[C@H](CCC1)C(=O)N[C@@H](C)C(=O)N1C[C@H](CCC1)C(=O)N
DPR4-1x	CC(=O)N[C@@H]1[C@@H](CCCC1)C(=O)N1C[C@H](CCC1)C(=O)N
DPR4-3x	NCC(=O)N[C@@H]1[C@@H](CCCC1)C(=O)N1C[C@H](CCC1)C(=O)N[C@@H]1[C@@H](CCCC1)C(=O)N1C[C@H](CCC1)C(=O)N[C@@H]1[C@@H](CCCC1)C(=O)N1C[C@H](CCC1)C(=O)N
DPR4-10x	CC(=O)N[C@@H]1[C@@H](CCCC1)C(=O)N1C[C@H](CCC1)C(=O)N[C@@H]1[C@@H](CCCC1)C(=O)N1C[C@H](CCC1)C(=O)N[C@@H]1[C@@H](CCCC1)C(=O)N1C[C@H](CCC1)C(=O)N[C@@H]1[C@@H](CCCC1)C(=O)N1C[C@H](CCC1)C(=O)N[C@@H]1[C@@H](CCCC1)C(=O)N1C[C@H](CCC1)C(=O)N[C@@H]1[C@@H](CCCC1)C(=O)N1C[C@H](CCC1)C(=O)N[C@@H]1[C@@H](CCCC1)C(=O)N1C[C@H](CCC1)C(=O)N[C@@H]1[C@@H](CCCC1)C(=O)N1C[C@H](CCC1)C(=O)N[C@@H]1[C@@H](CCCC1)C(=O)N1C[C@H](CCC1)C(=O)N[C@@H]1[C@@H](CCCC1)C(=O)N1C[C@H](CCC1)C(=O)N

Supporting HPLC and MS traces are provided on the following pages.

DPR1 1x

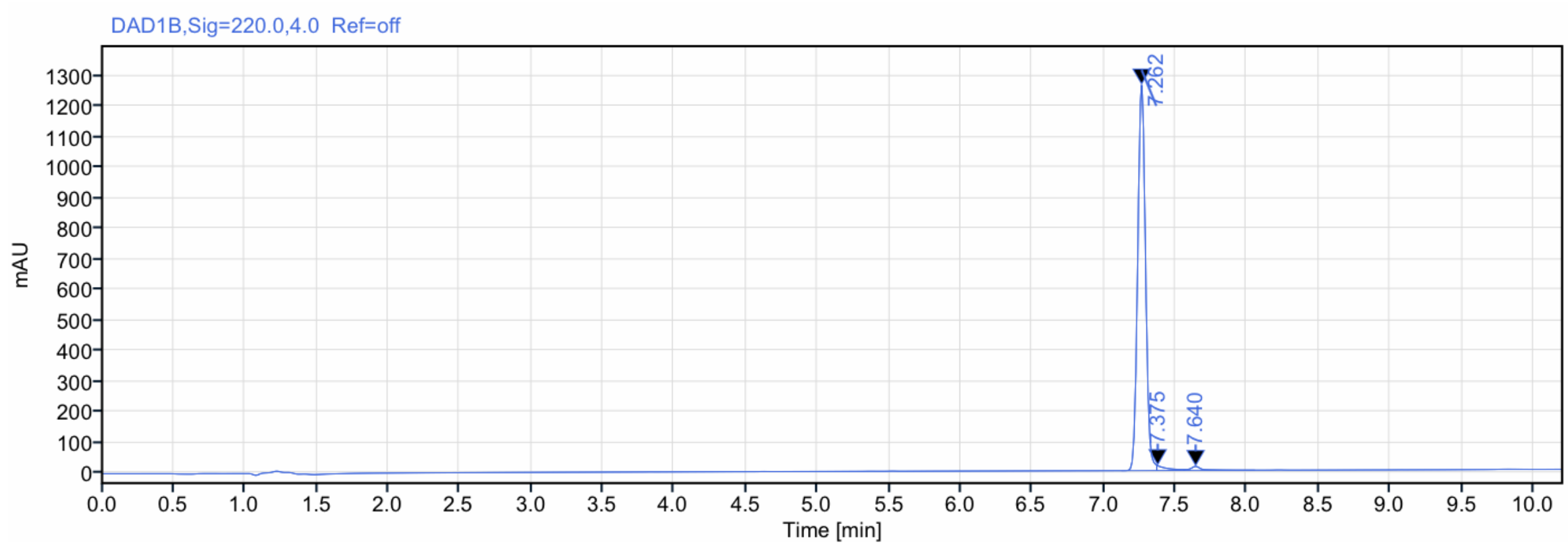

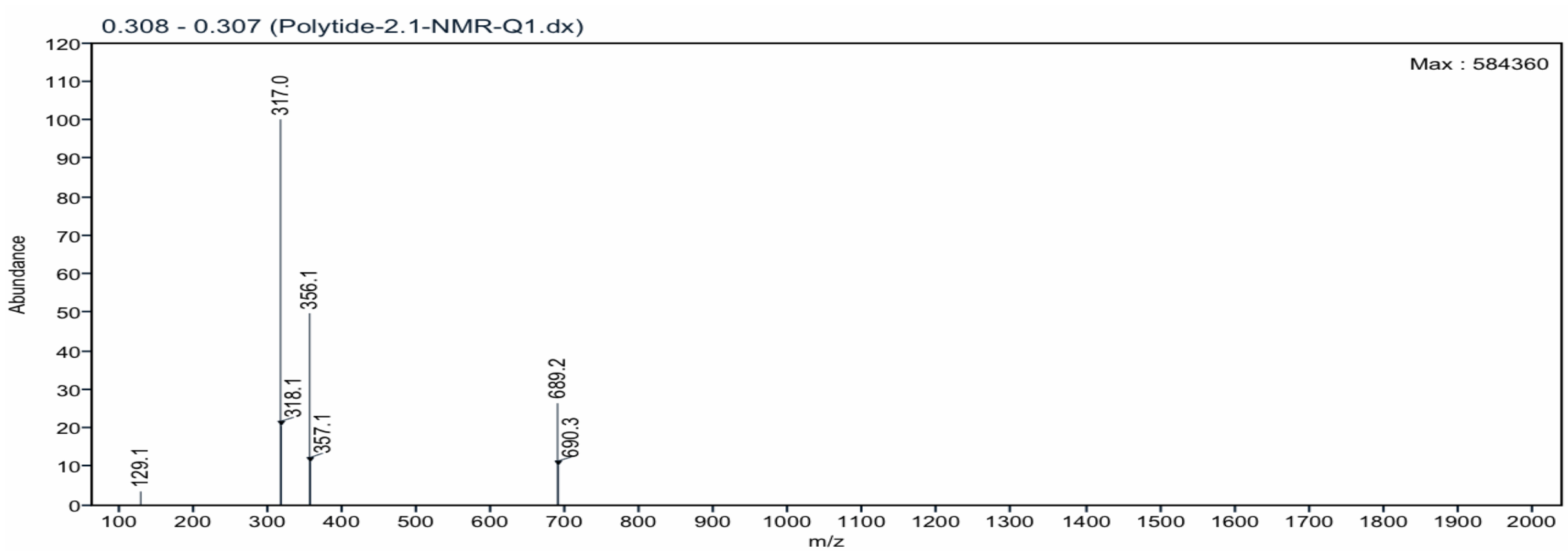

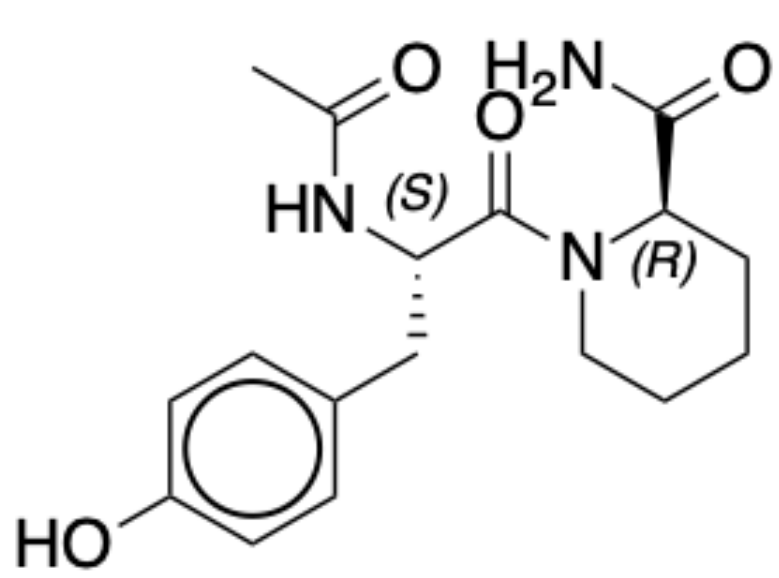

Molecular Weight: 333.39

DPR1 3x

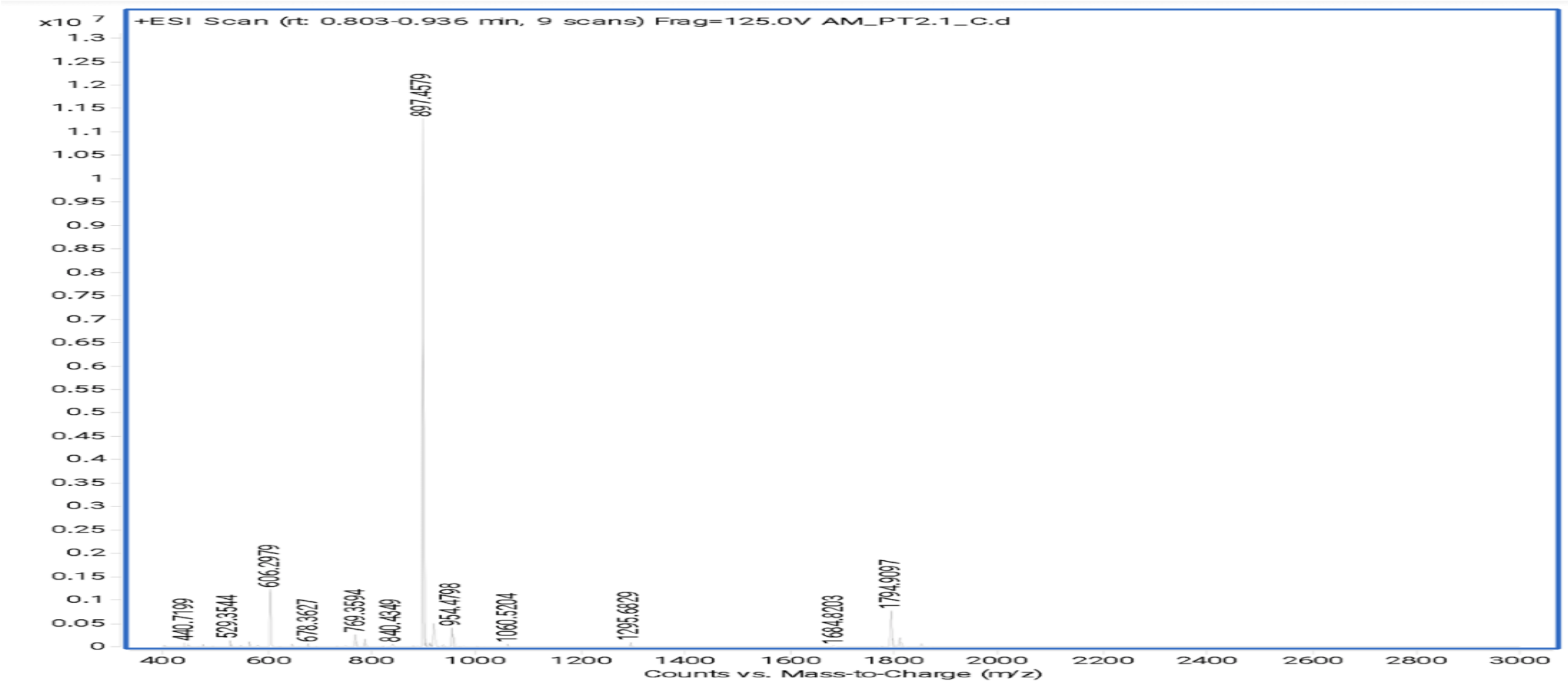

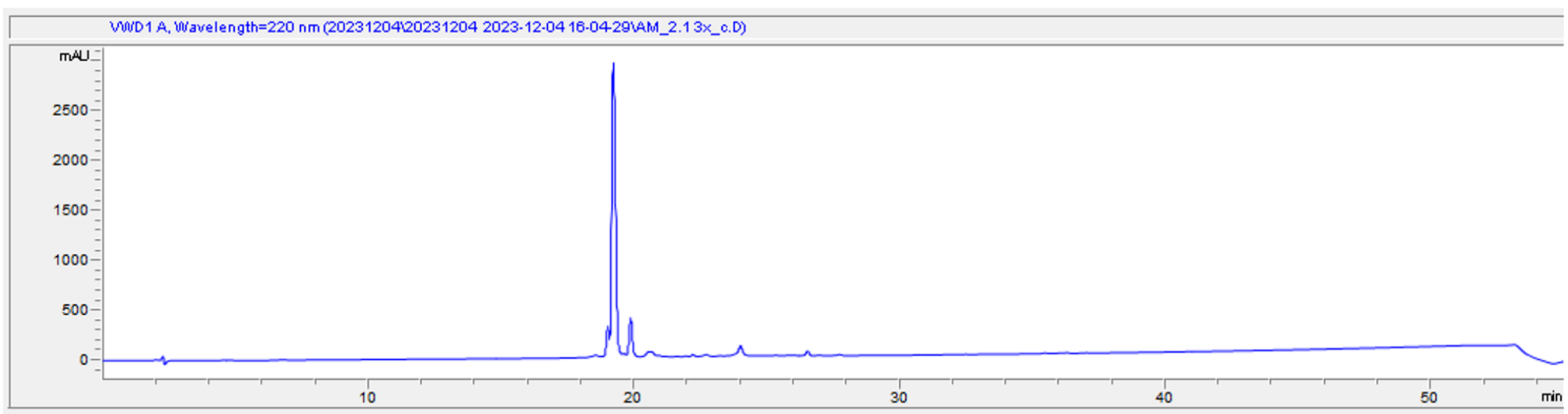

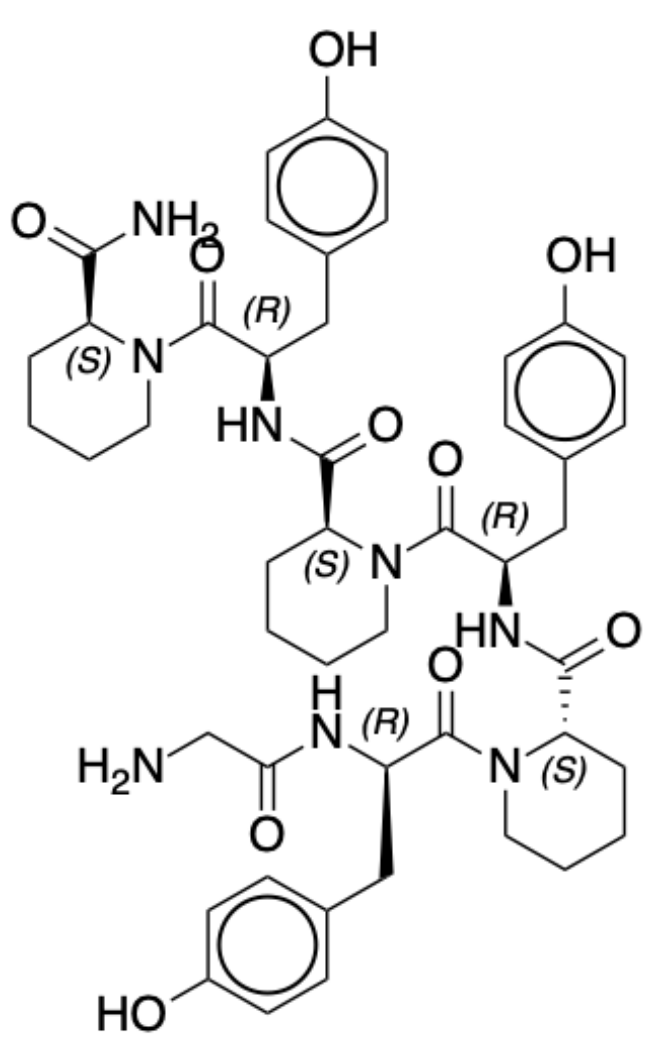

Molecular Weight: 897.04

DPR1 5x

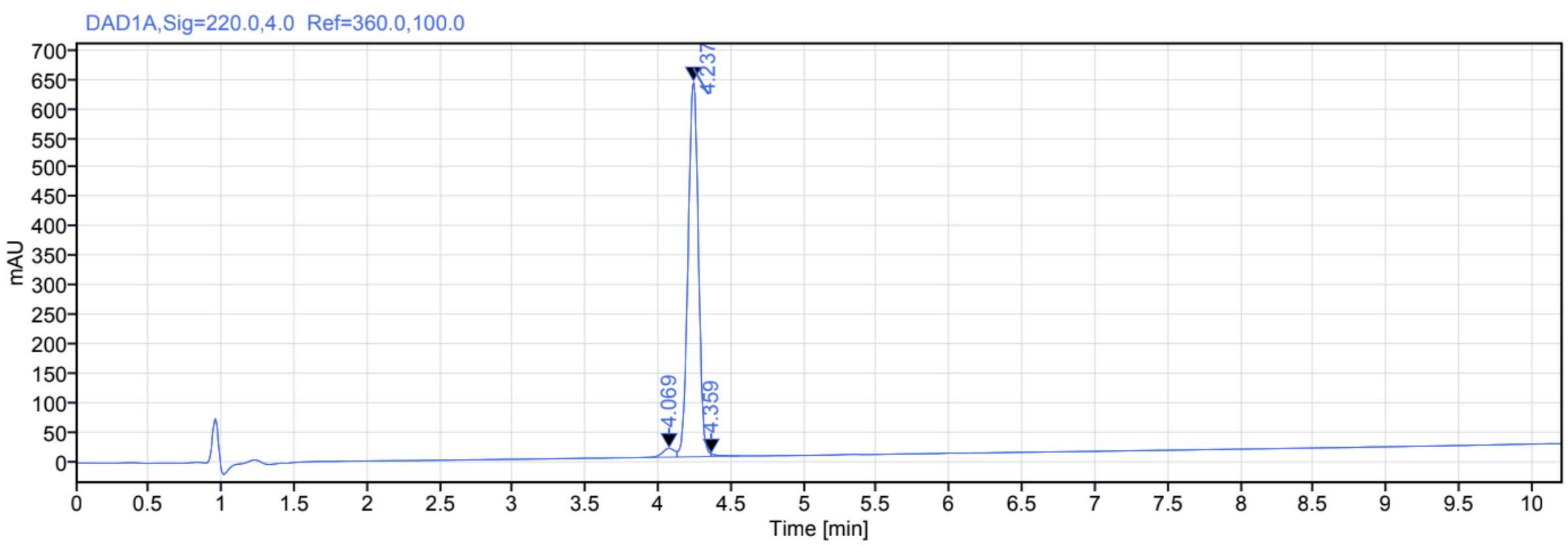

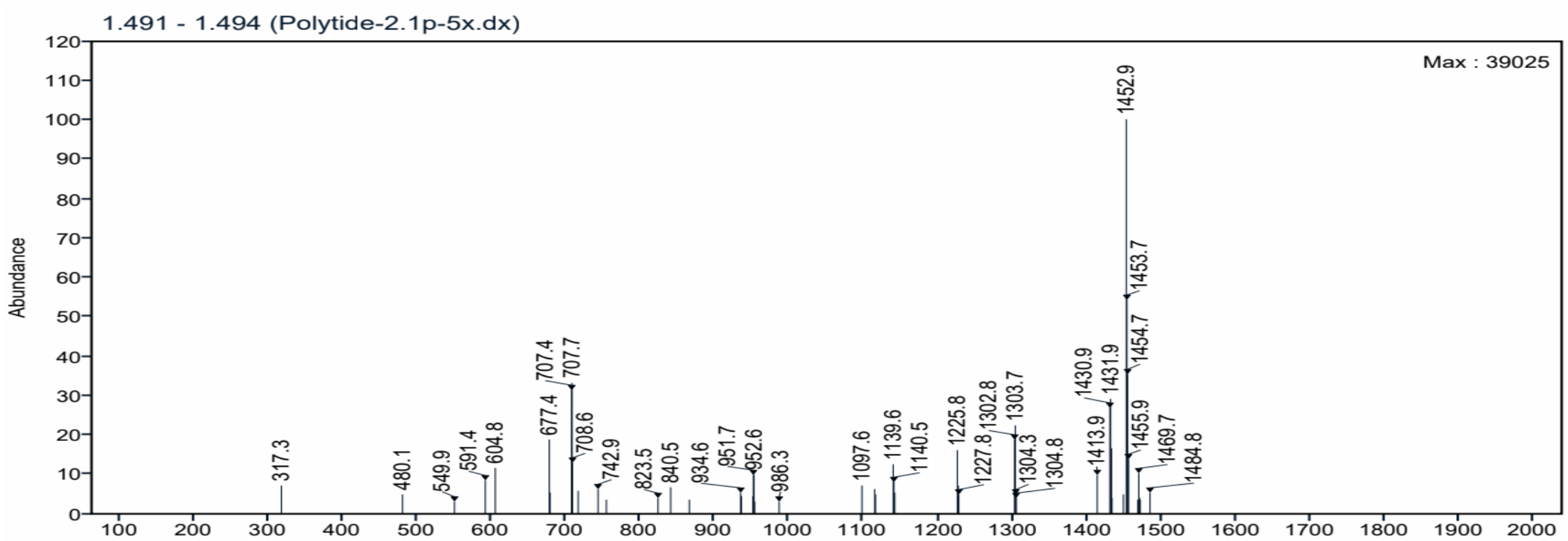

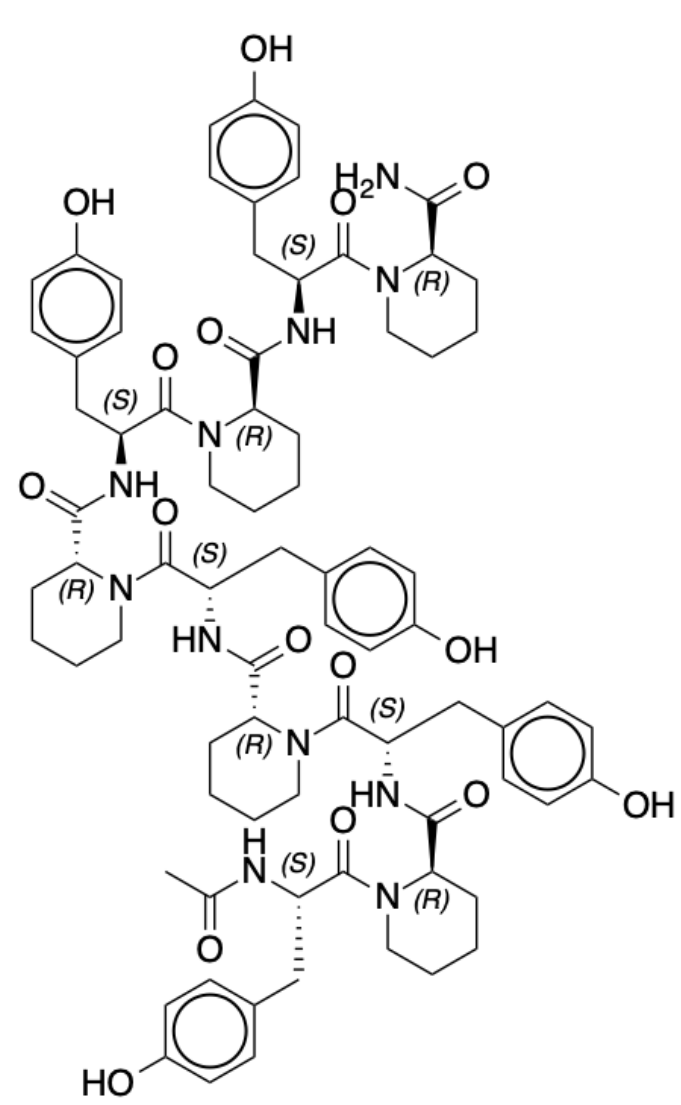

Molecular Weight: 1430.67

DPR2 1x

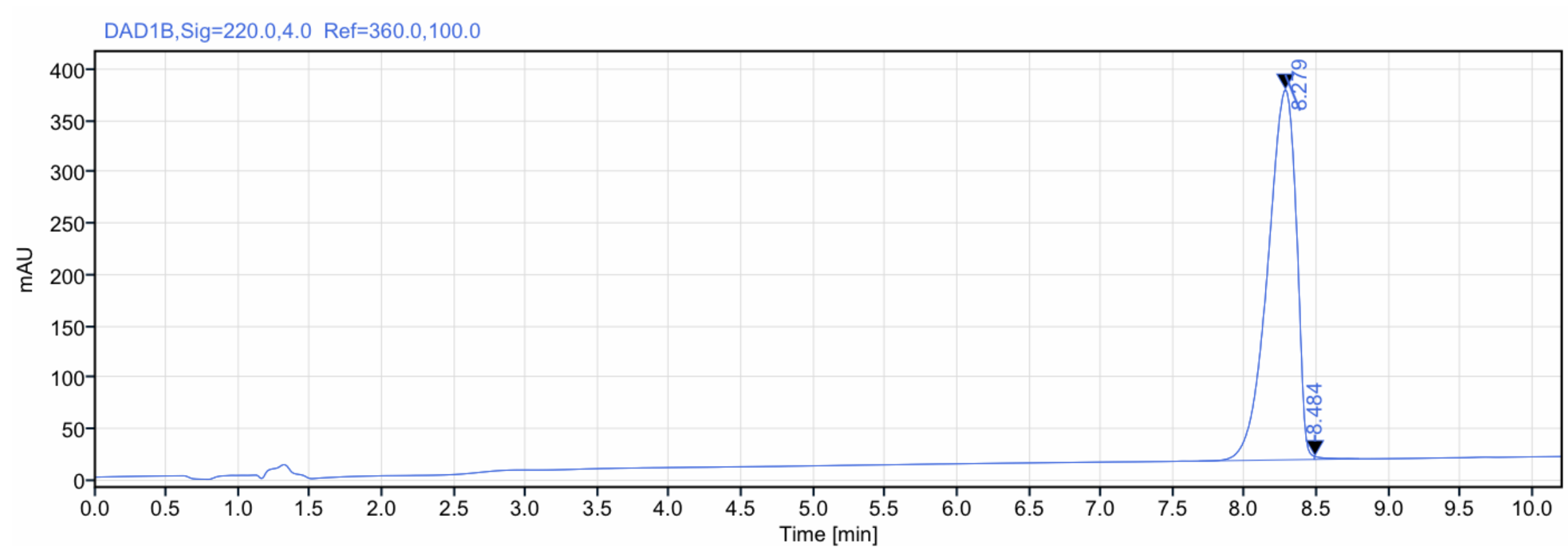

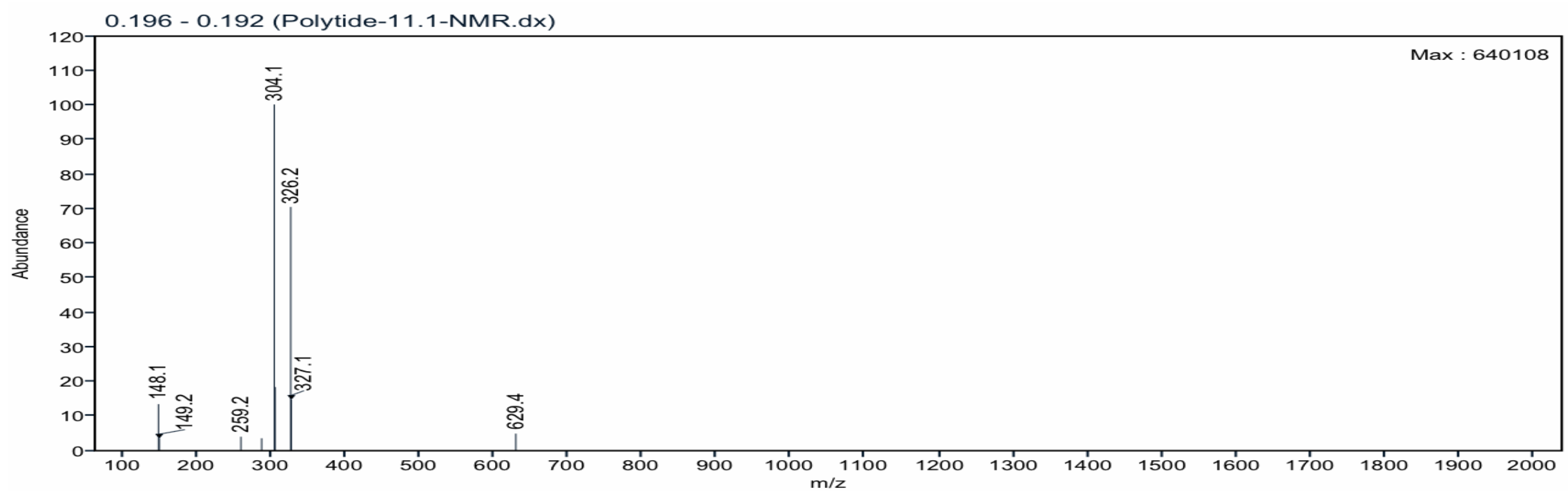

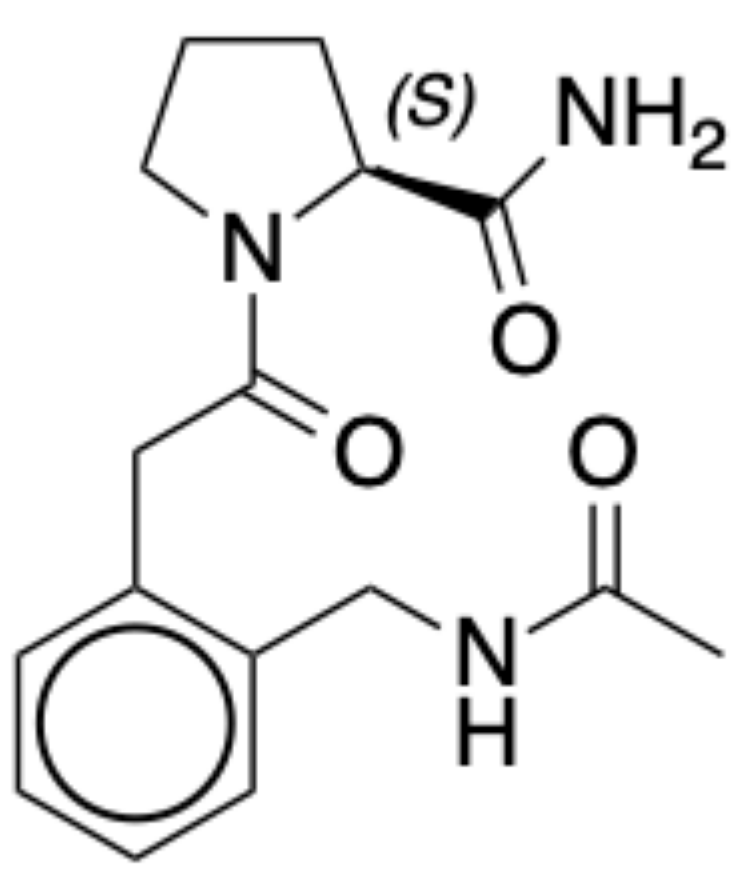

Molecular Weight: 303.36

DPR2 3x

User Chromatograms

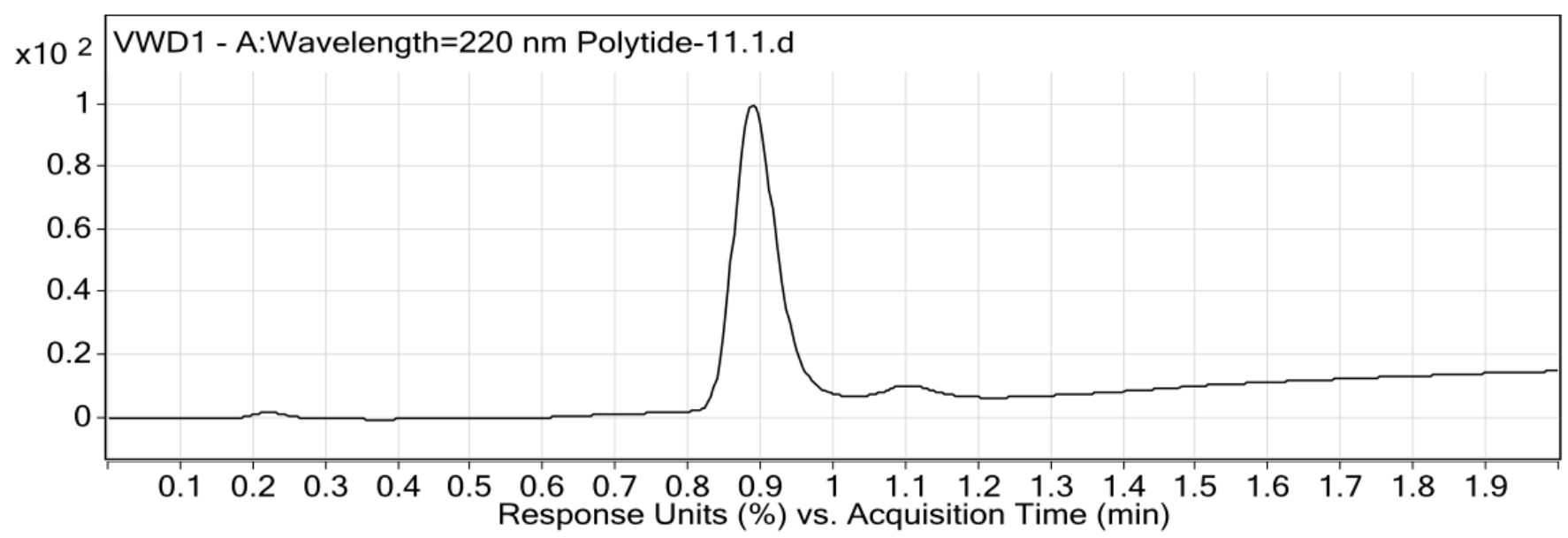

User Spectra

Fragmentor Voltage	Collision Energy	Ionization Mode
135	0	ESI

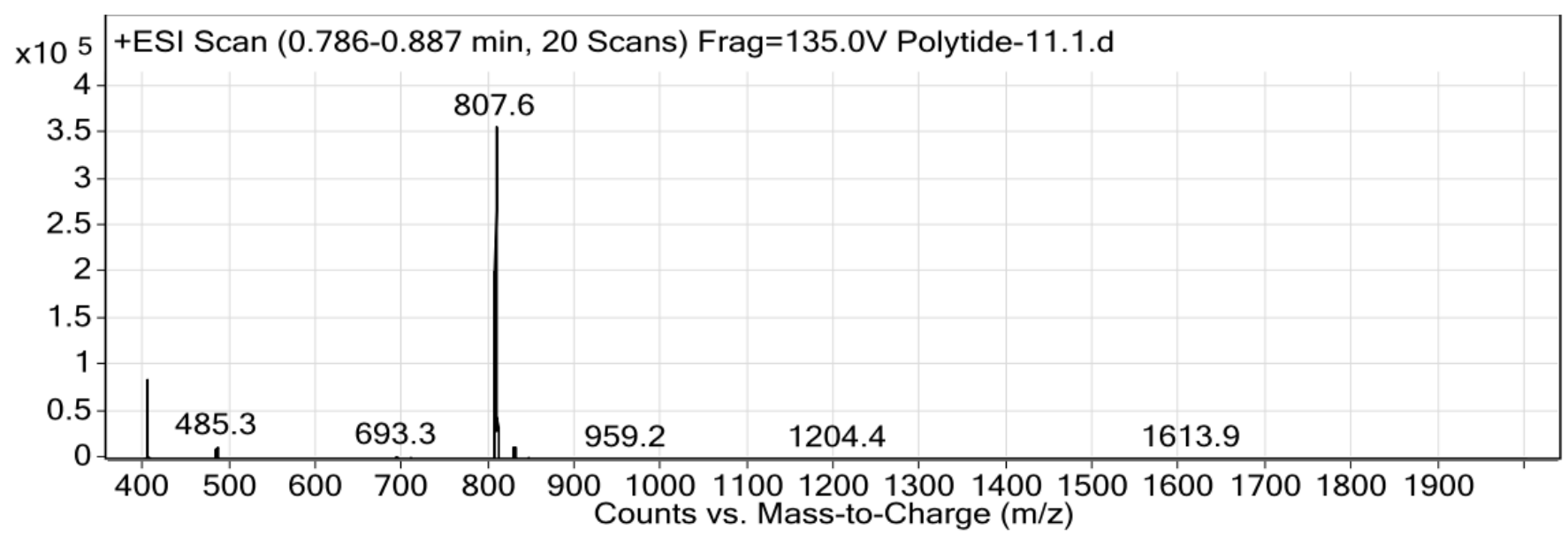

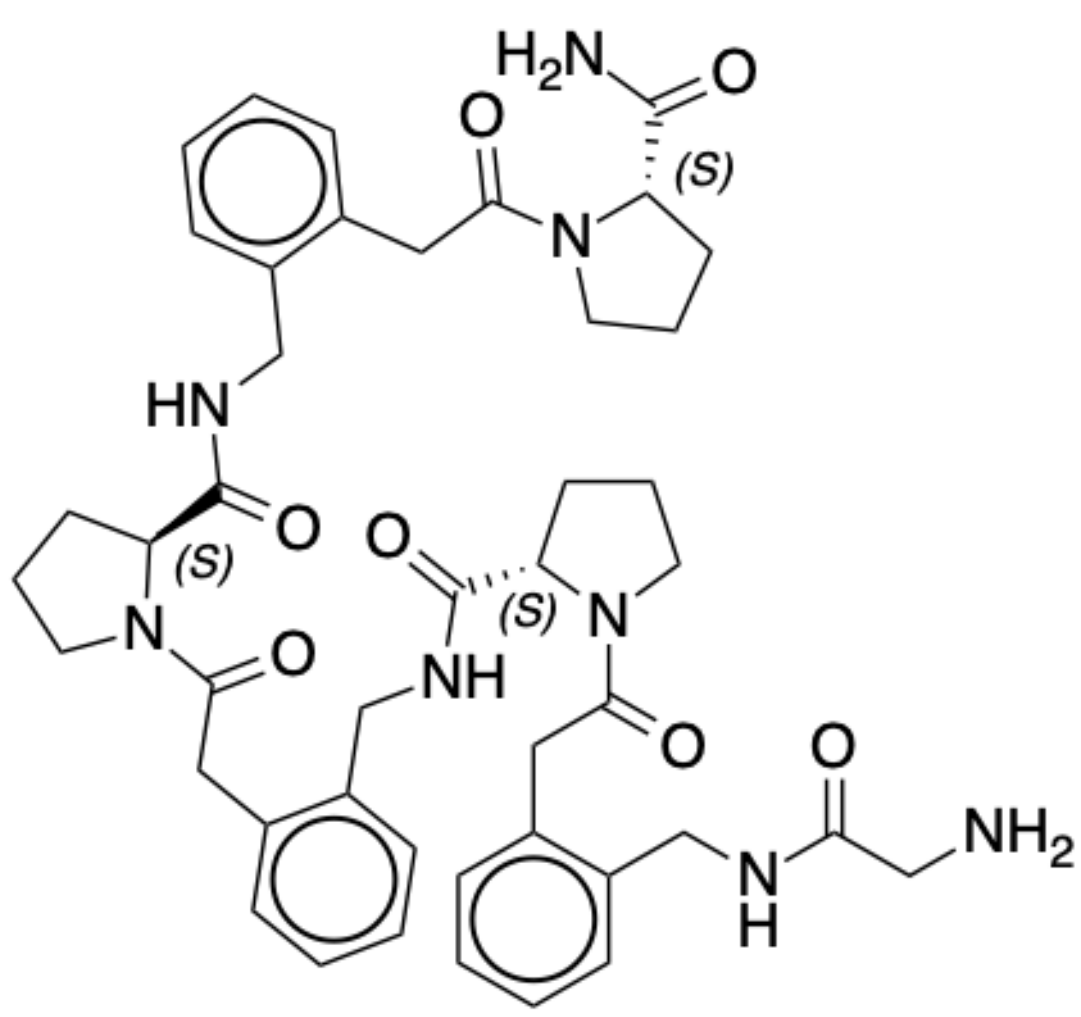

Molecular Weight: 806.97

DPR2 10x

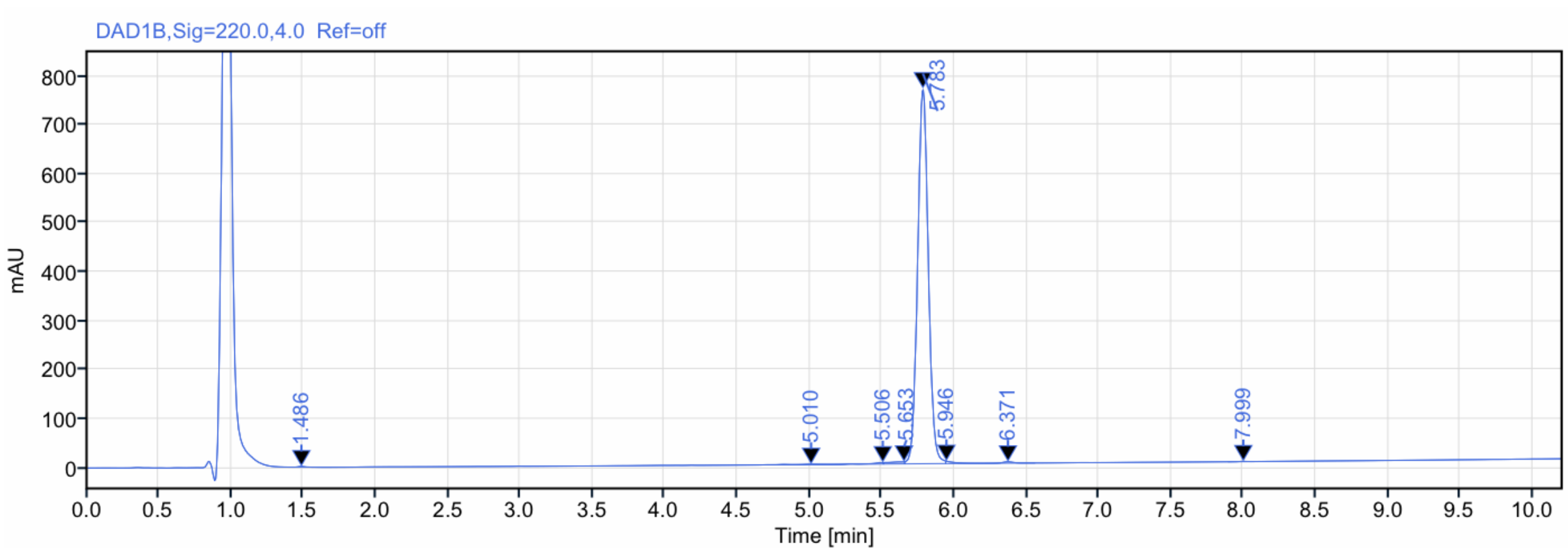

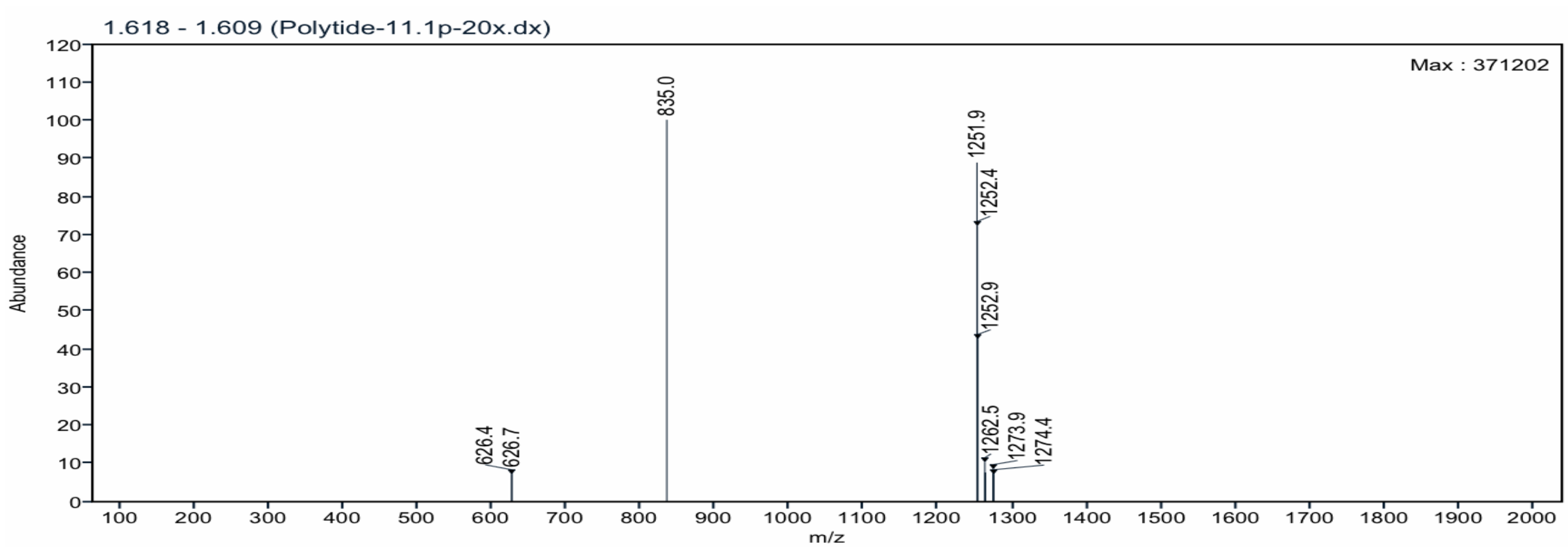

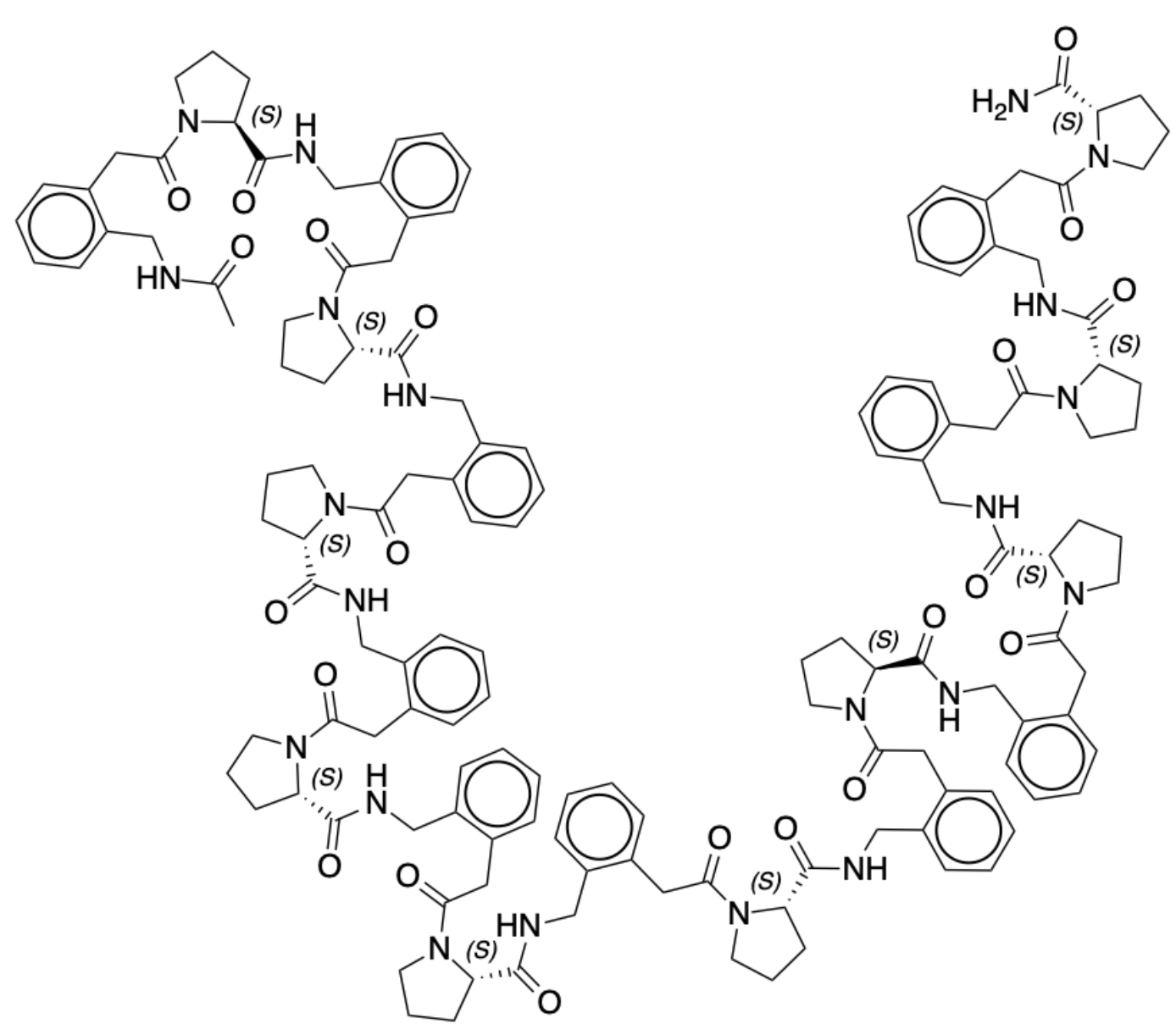

Molecular Weight: 2502.01

DPR3 1x

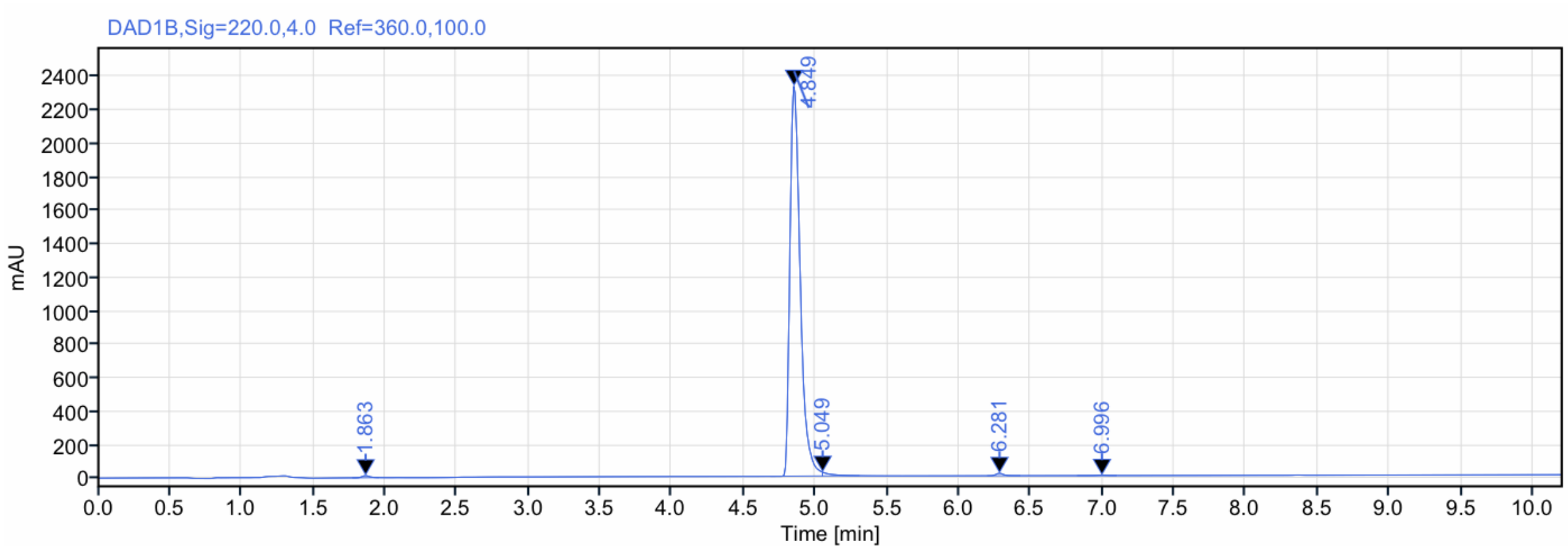

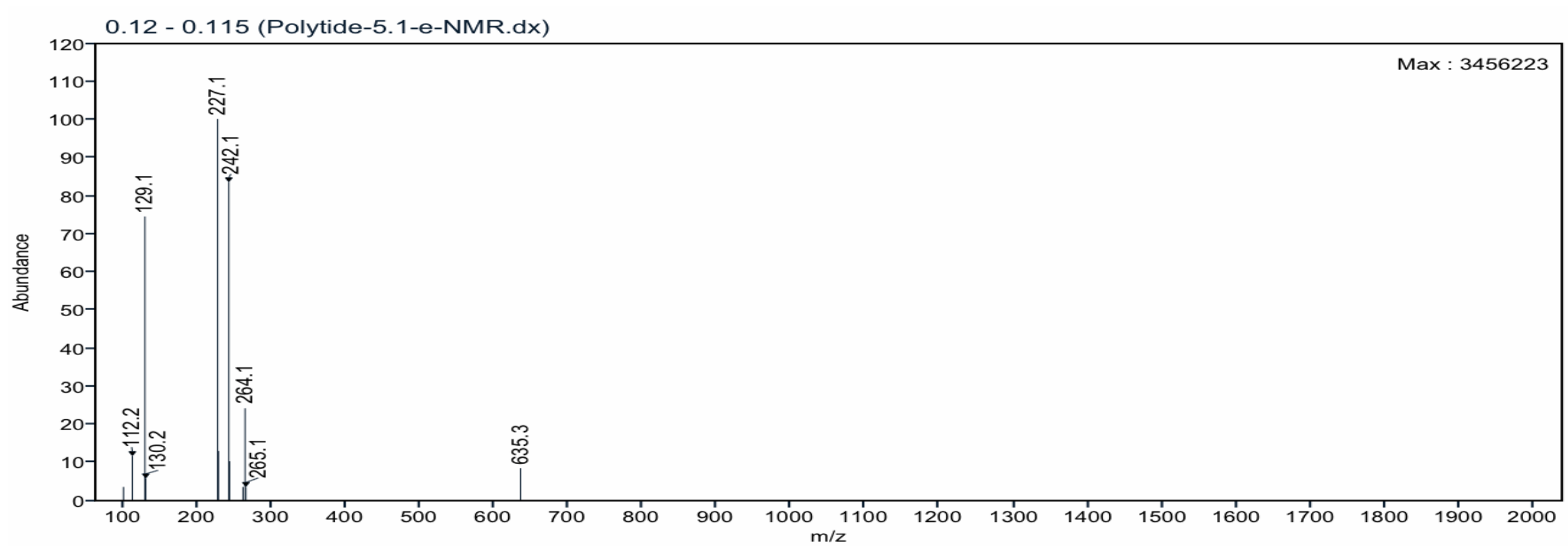

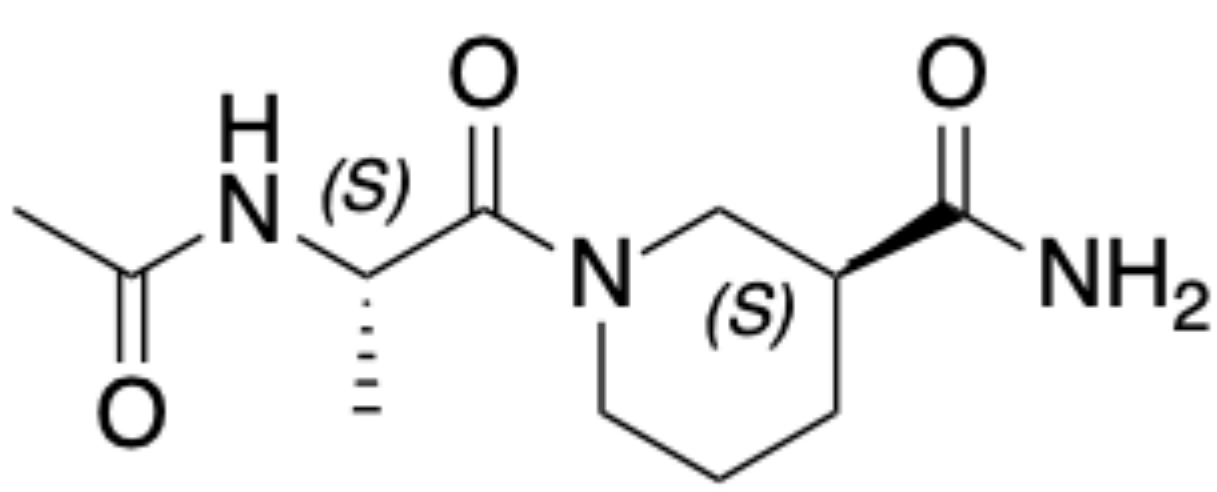

Molecular Weight: 241.29

DPR3 4x

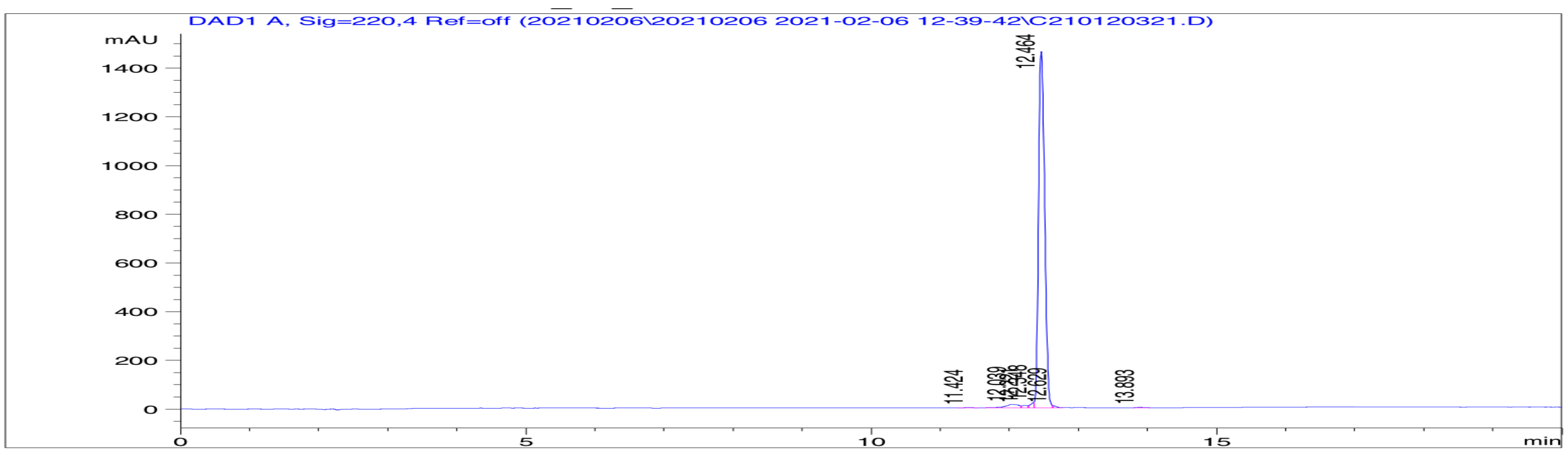

Sample Spectra

+ Scan (rt: 0.132-0.247 min)

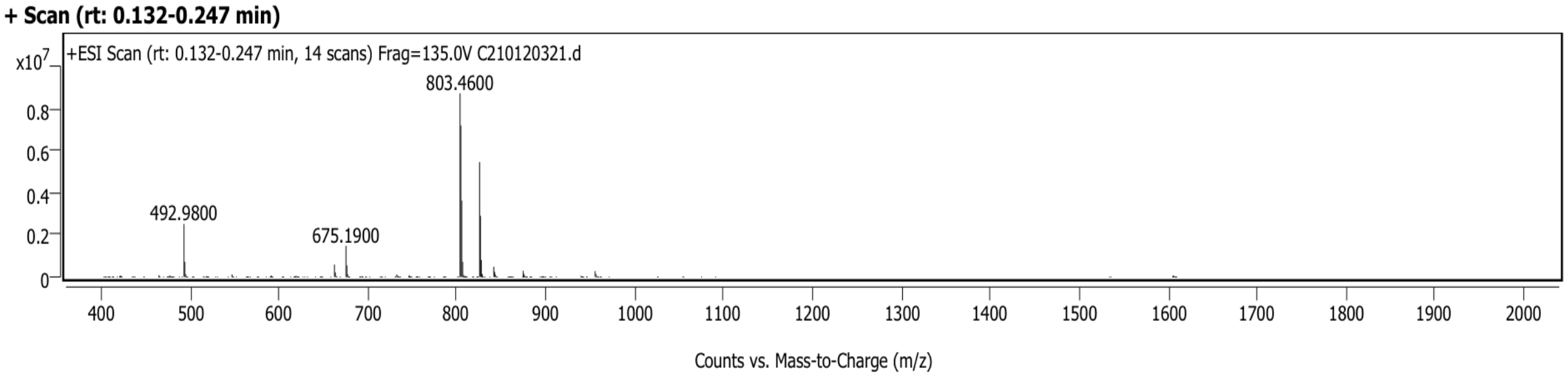

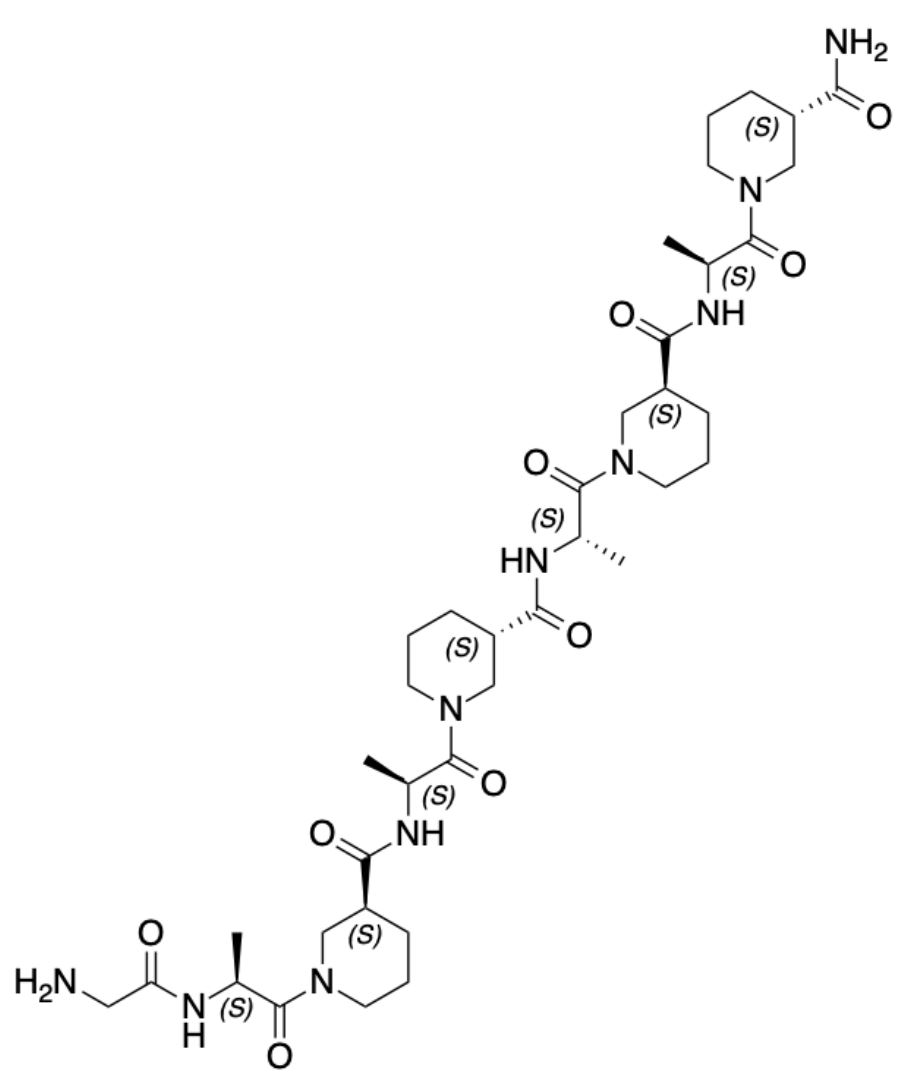

Molecular Weight: 802.98

DPR3 10x

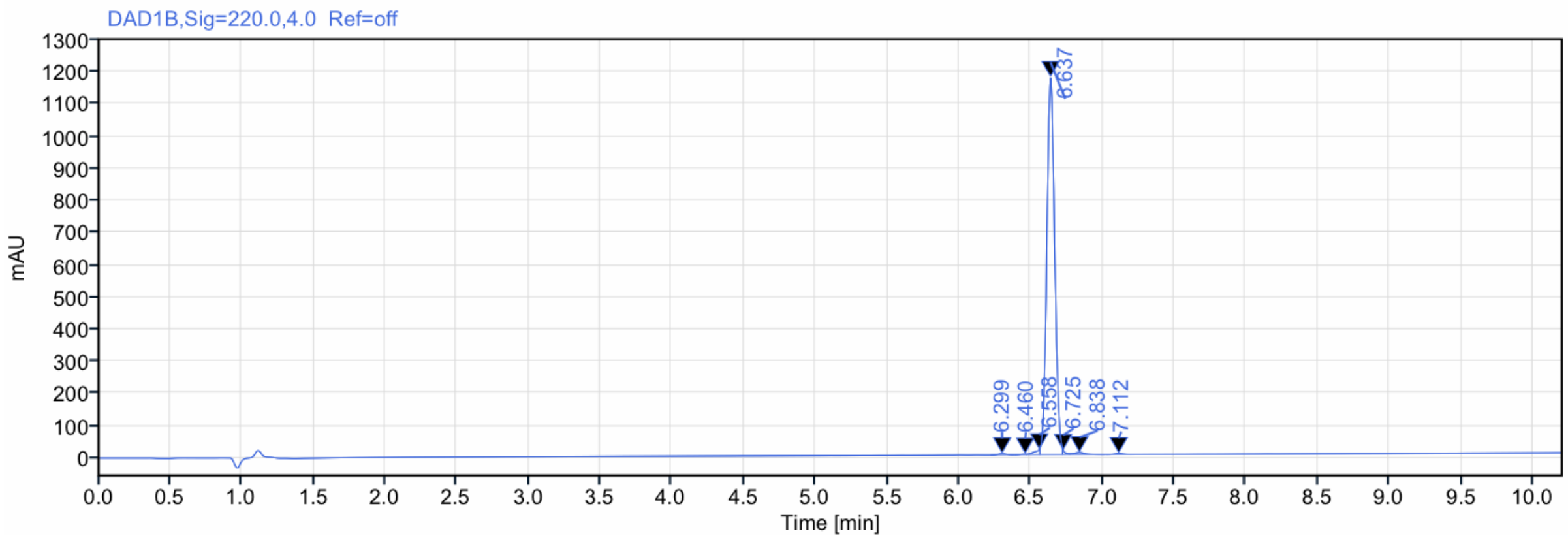

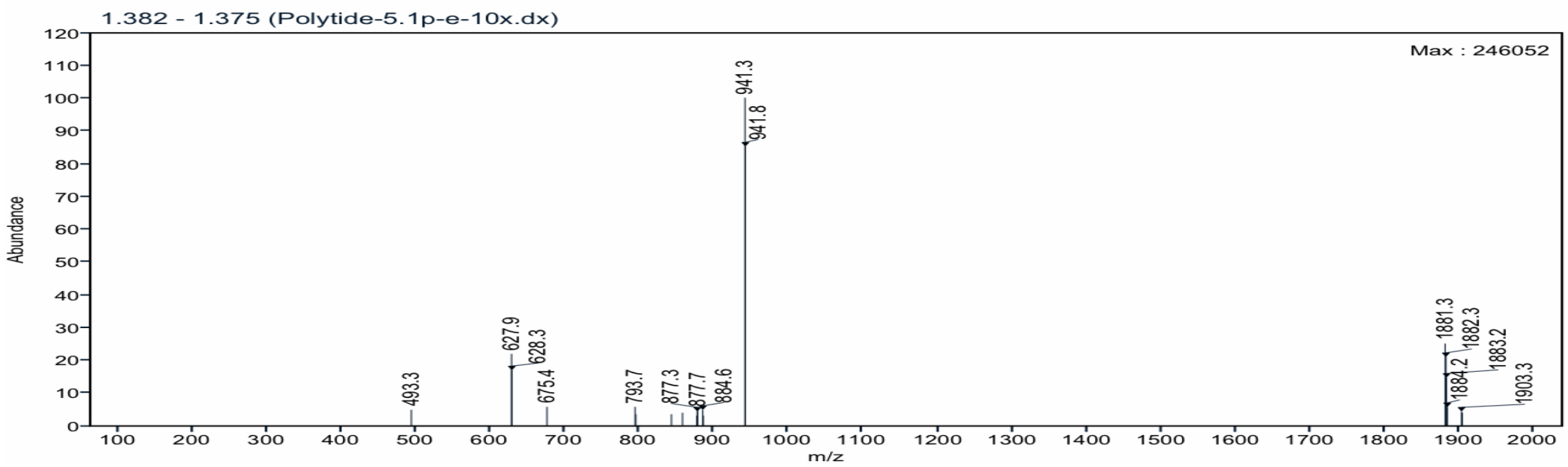

Chemical structure on the next page.

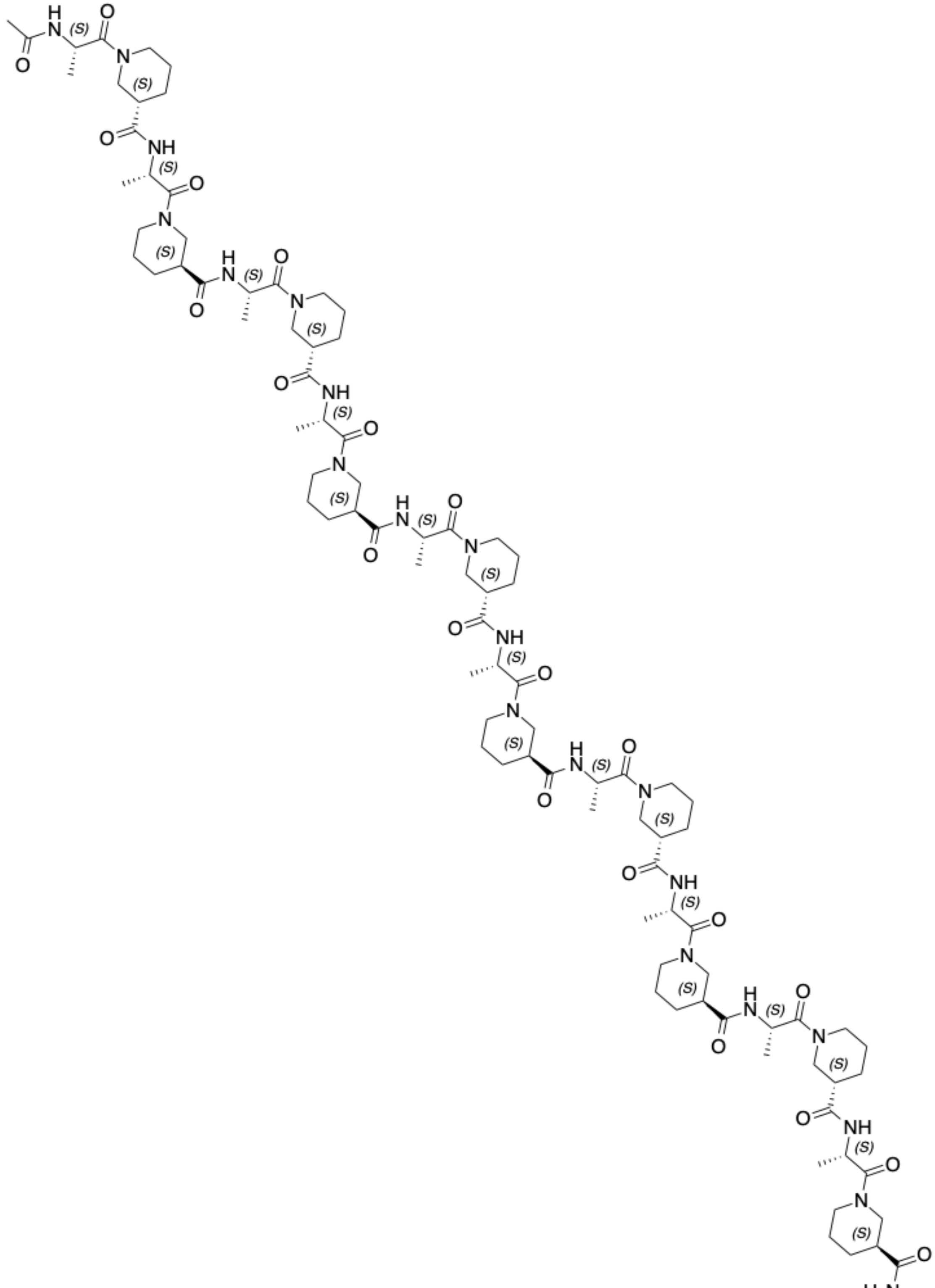

Molecular Weight: 1881.30

DPR4 1x

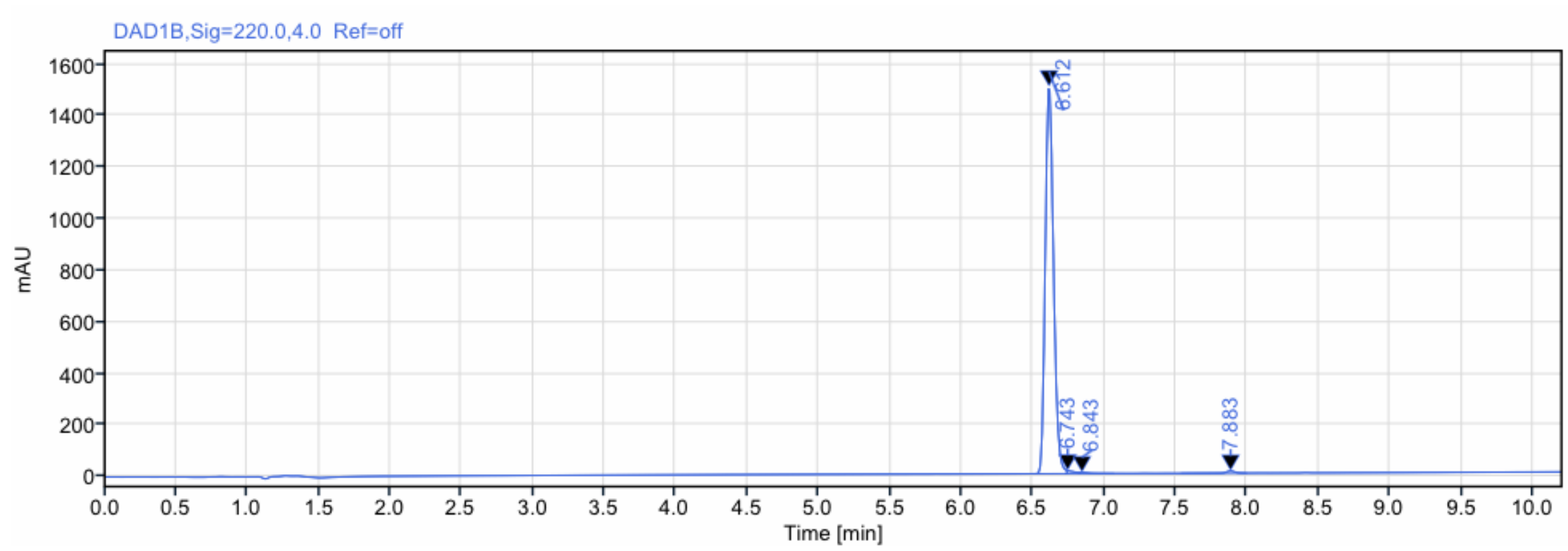

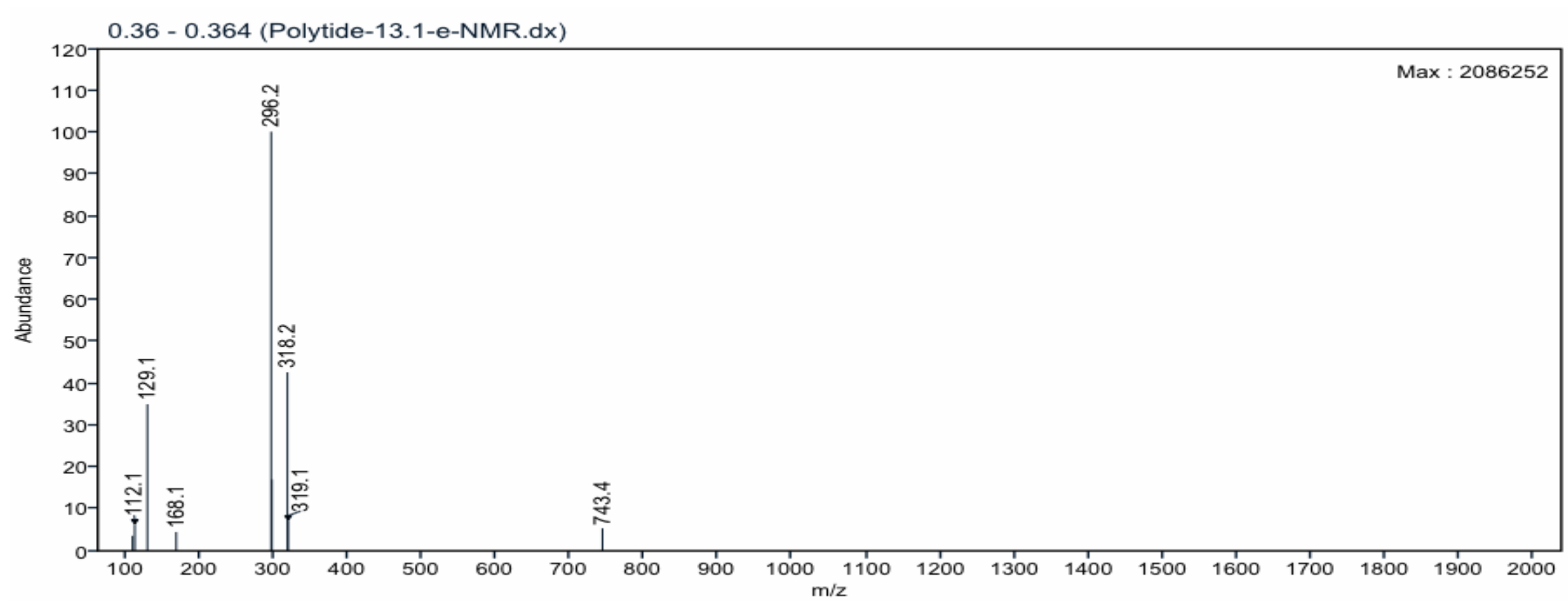

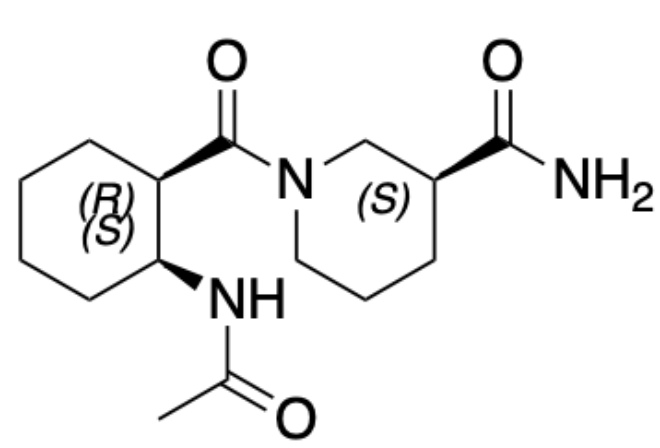

Molecular Weight: 295.38

DPR4 3x

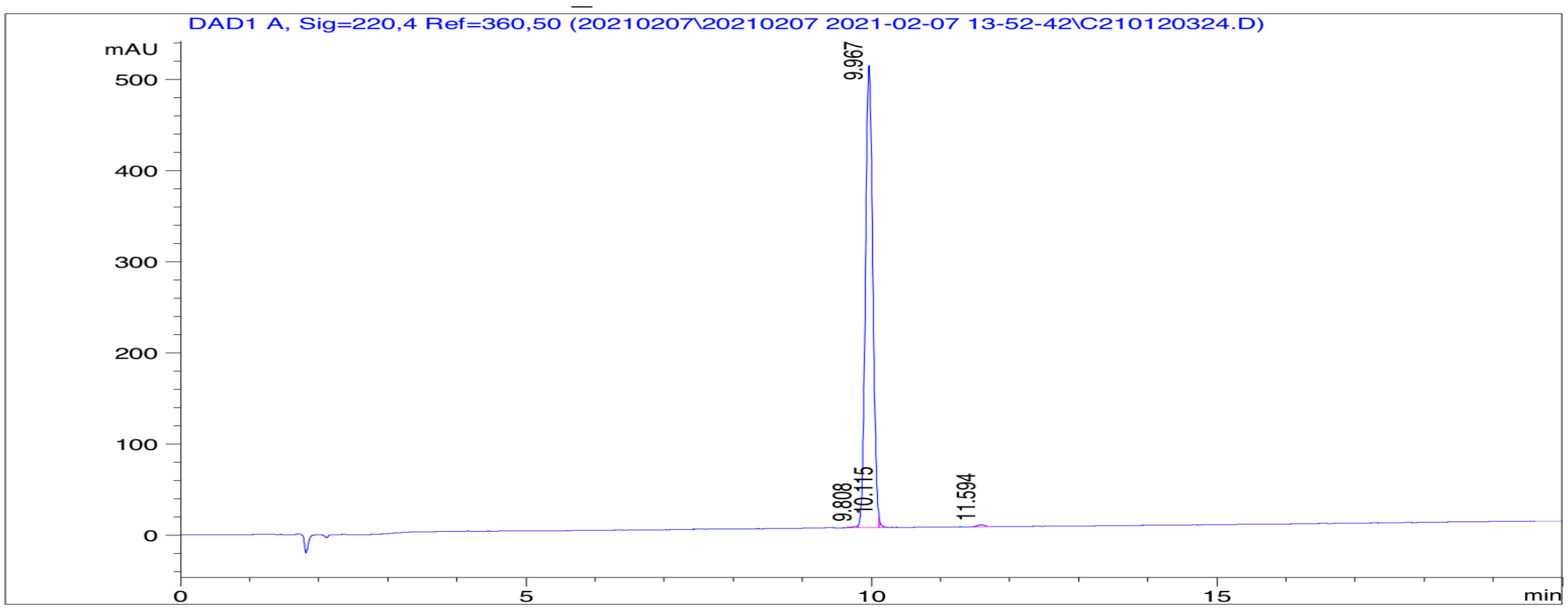

+ Scan (rt: 0.889-0.960 min)

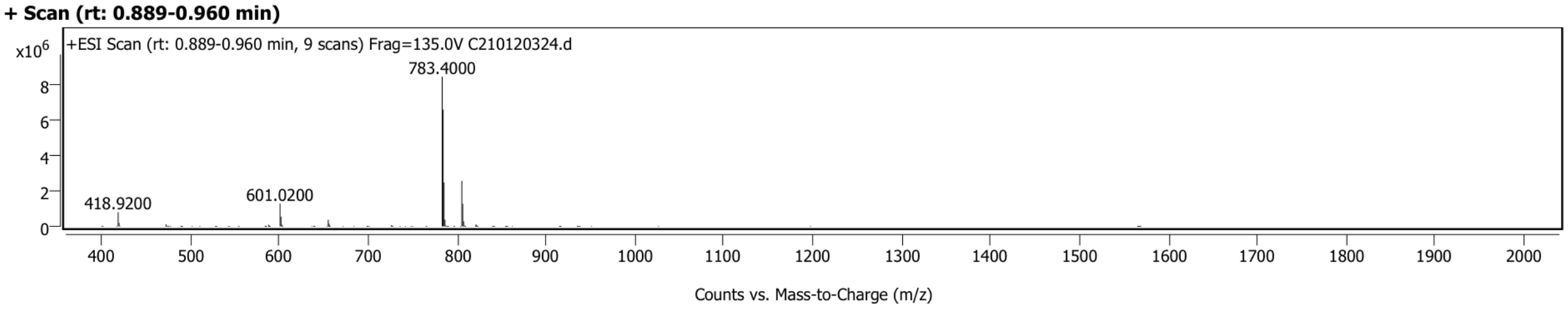

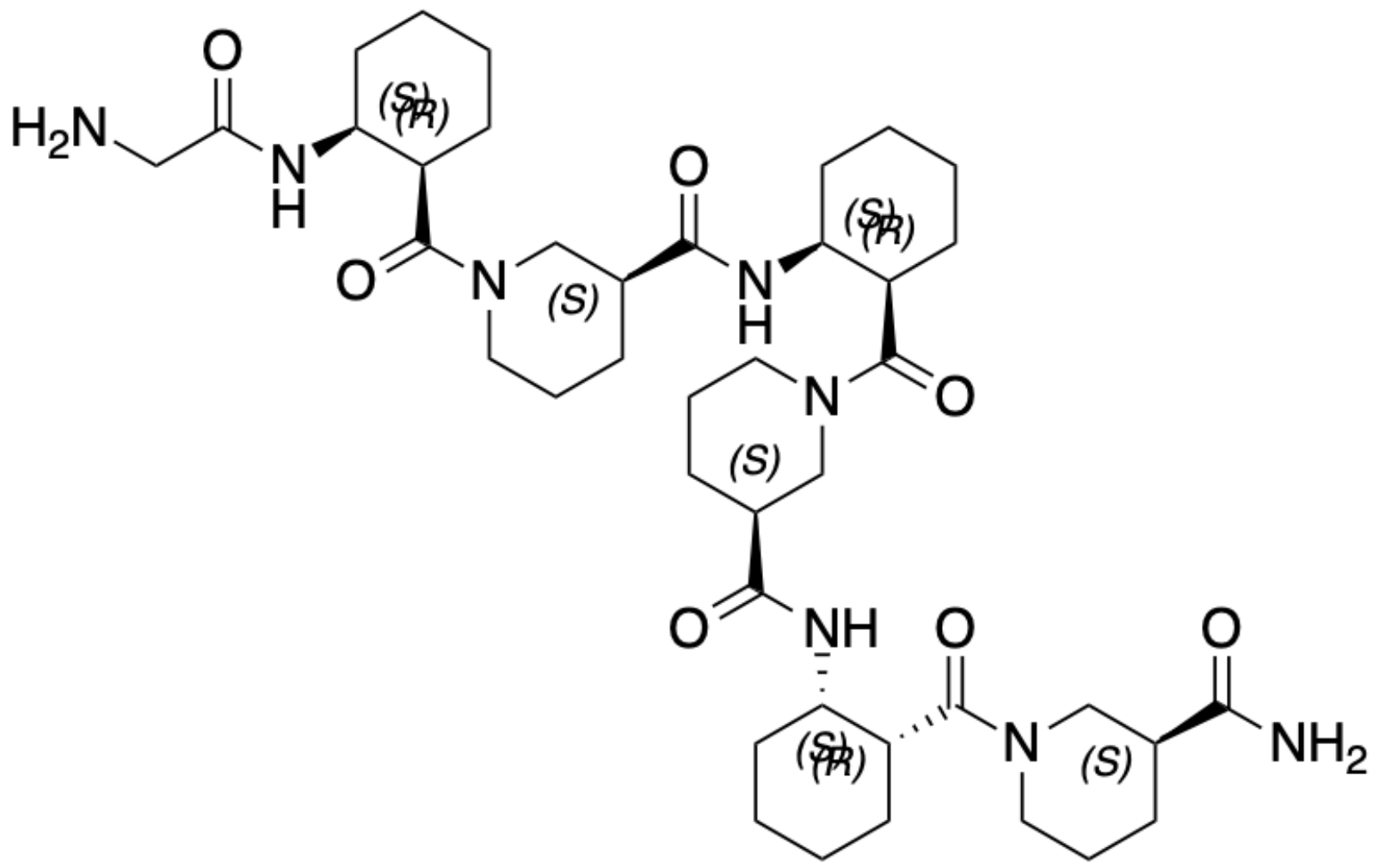

Molecular Weight: 783.03

DPR4 10x

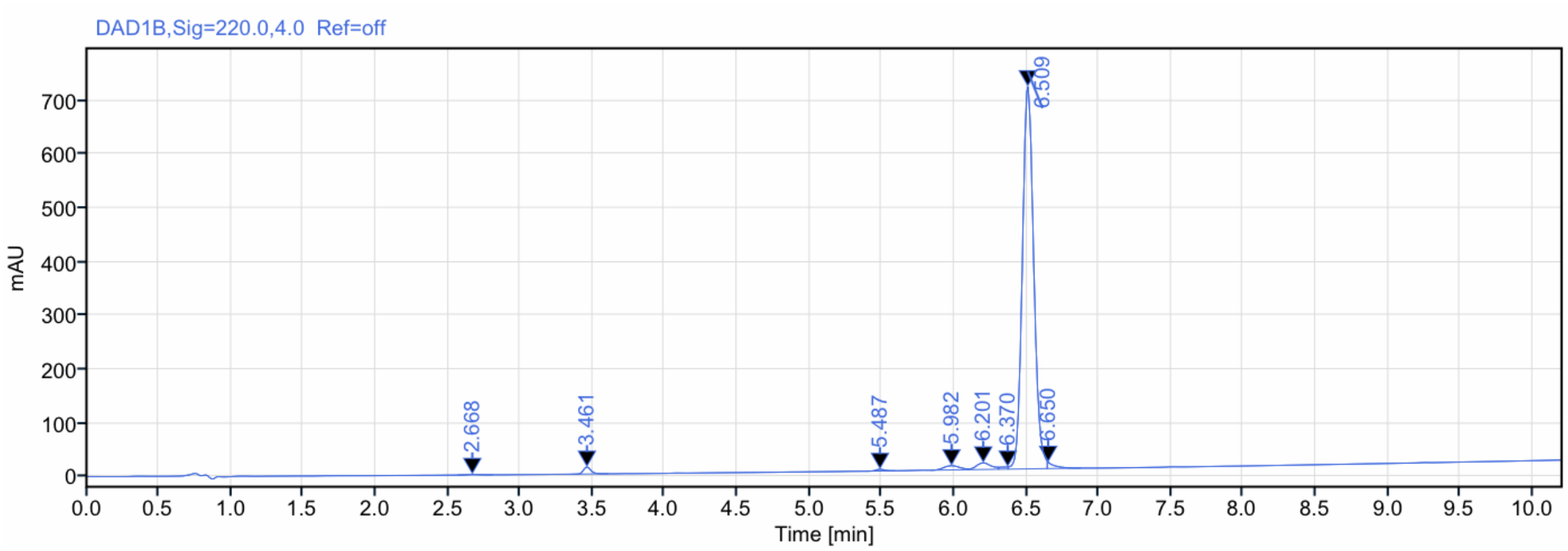

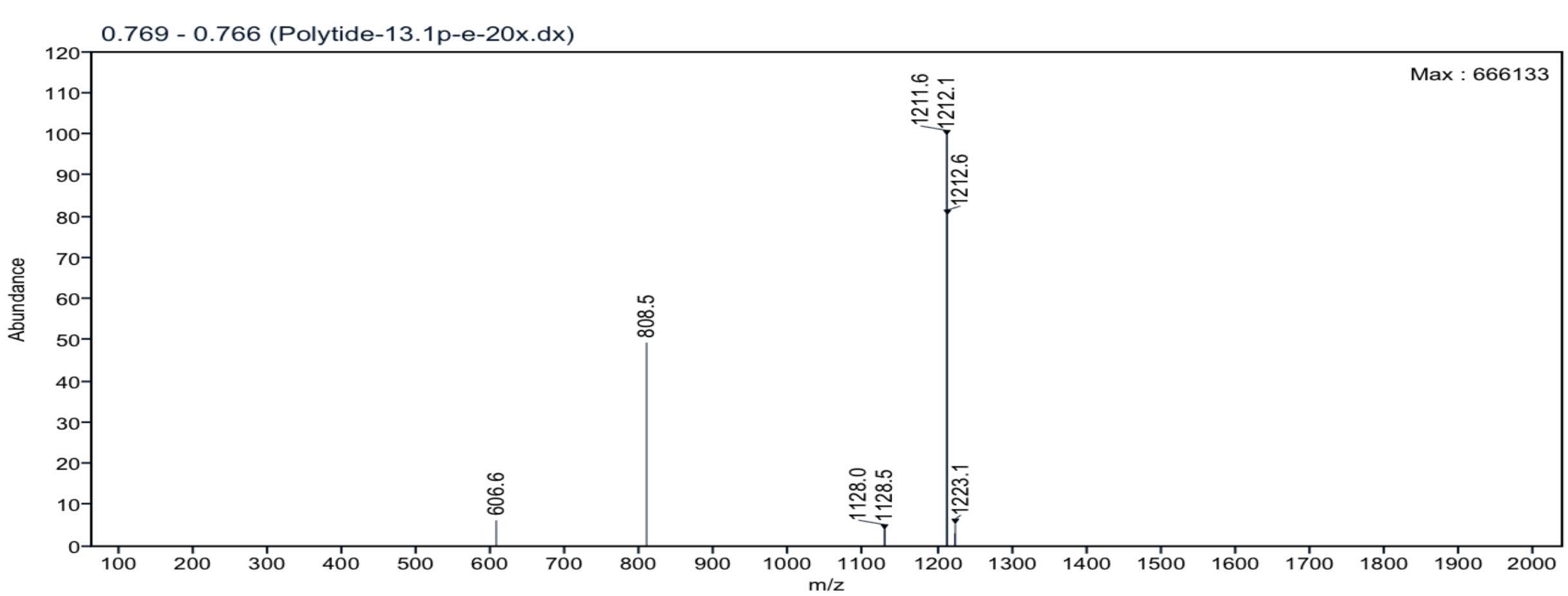

Chemical structure is on the next page.

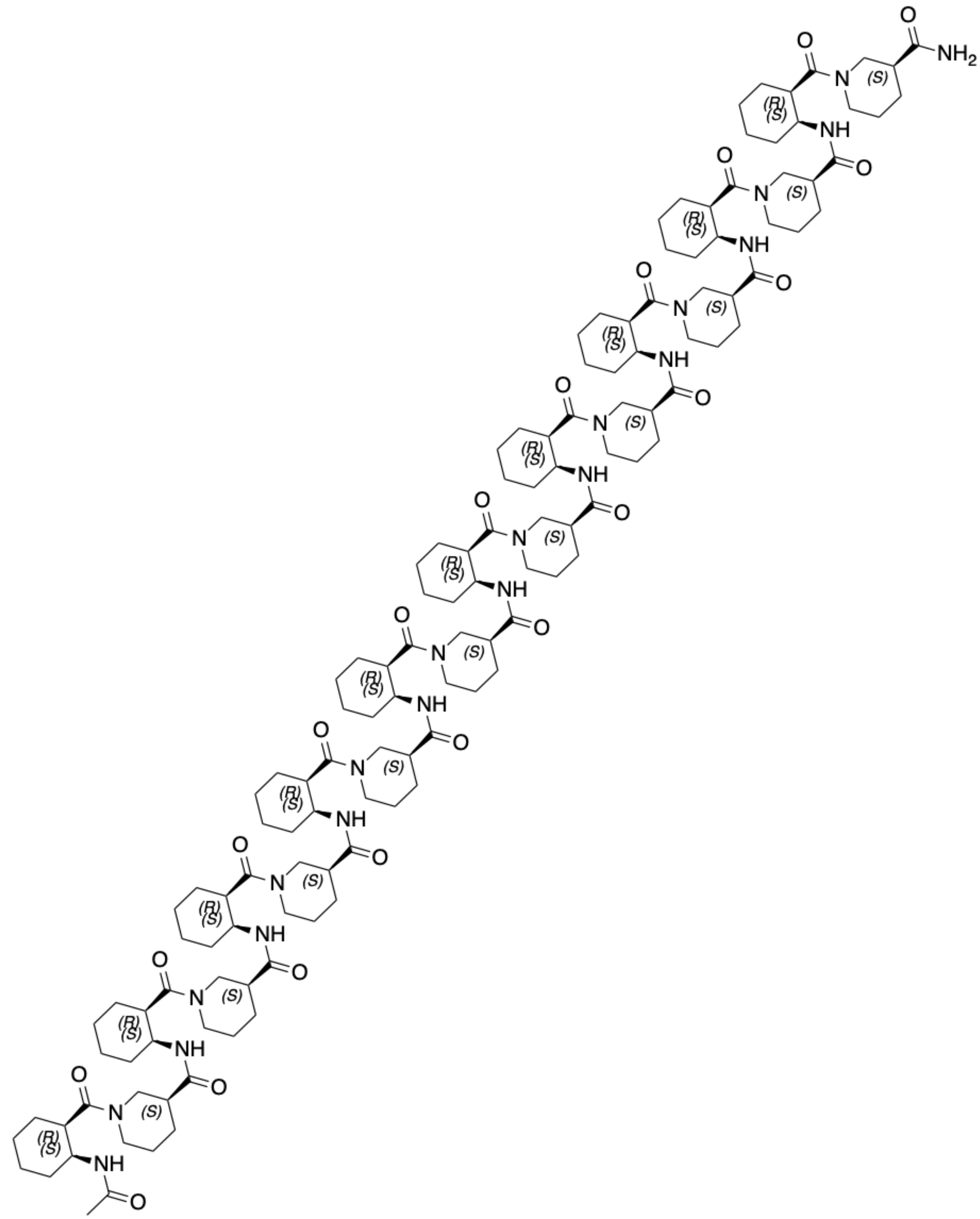

Molecular Weight: 2422.22

Table S11. Calculated Relative Energies of Monomer Conformations in the Design Structures Compared to Lowest Energy Monomer Conformation Sampled during Energy Landscape Generation

**DPR1**	**PHE (TYR)**	**PIP**
**dE (kcal/mol)**	0.66	0.97
**DPR2**	**AMACBEN2**	**PRO**
**dE (kcal/mol)**	0.50	0.13

Supplemental Code

Code S1. boltzmann_adaptive_sampler.py

```
from timeit import default_timer

import numpy as np
import scipy as sp
from scipy.spatial import Delaunay
from scipy.interpolate import NearestNDInterpolator
from scipy.interpolate import LinearNDInterpolator
# from sklearn.neighbors import KNeighborsRegressor


class BoltzmannAdaptiveSampler(object):
    def __init__(self, function, points, values, kt=3.0, clip=12.0, nthreads=1):
        self.function = function

        assert len(points) == len(values)

        self.delaunay = Delaunay(points, incremental=True, qhull_options='Q0 Q12 Qc QJ')
        self.values = values

        self.kt = kt
        self.clip = clip
        self.nthreads = nthreads

        self.dims = points.shape[-1]

        self.select_runtime = 0.0
        self.function_runtime = 0.0
        self.update_runtime = 0.0
        self.total_runtime = 0.0

        self.controller = float(-self.dims)


    def run(self):
        run_size = min([len(self.simplices), int((10**self.controller)*len(self.simplices)/(4*self.nthreads) + 1) *
(4*self.nthreads)])
        print(f'run_size: {run_size}, controller: {round(self.controller, 2)}')

        ###
        t = default_timer()
```

```
        selection = np.argpartition(self.losses, -run_size)[-run_size:]
        points = self.weighted_centers[selection]
        select_runtime = default_timer() - t

        ###
        t = default_timer()
        values = self.function(points)
        function_runtime = default_timer() - t

        ###
        t = default_timer()
        self.delaunay.add_points(points)
        self.values = np.concatenate([self.values, values])
        update_runtime = default_timer() - t

        ###
        total_runtime = select_runtime + function_runtime + update_runtime

        r = function_runtime / total_runtime; r_0 = 0.95; r_C = 1.0
        self.controller -= (r-r_0)/r_C

        self.select_runtime += select_runtime
        self.function_runtime += function_runtime
        self.update_runtime += update_runtime
        self.total_runtime += total_runtime

        print(f'select_runtime: {round(select_runtime, 2)} [s]')
        print(f'function_runtime : {round(function_runtime, 2)} [s]')
        print(f'update_runtime: {round(update_runtime, 2)} [s]')

        print(f'simplices: {len(self.simplices)}, points: {len(self.points)}')
        print(f'function runtime: {round(100 * self.function_runtime / self.total_runtime, 2)} [%]')


    @property
    def points(self):
        return self.delaunay.points


    @property
    def simplices(self):
        return self.delaunay.simplices


    @property
    def centers(self):
        return np.average(self.points[self.simplices], axis=1)

    @property
```

```
    def weighted_centers(self):
        weights = np.tile(np.linalg.norm(self.points[self.simplices] - self.centers[:,np.newaxis,:],
axis=2)[:,:,np.newaxis], (1,1,self.dims))
        return np.average(self.points[self.simplices], axis=1, weights=weights)


    @property
    def delta_values(self):
        return np.clip(self.values - np.min(self.values), 0.0, self.clip)


    @property
    def boltzmann_values(self):
        return np.exp(self.delta_values / -self.kt)


    @property
    def volumes(self):
        return np.abs(np.linalg.det(self.points[self.simplices][:,1:,:] - self.points[self.simplices][:,:1,:]) /
np.math.factorial(self.dims))


    @property
    def losses(self):
        losses = self.volumes * np.squeeze(np.mean(self.boltzmann_values[self.simplices], axis=1))

        return losses / np.sum(losses)


    @property
    def interpolator(self):
        # return NearestNDInterpolator(self.points, self.delta_values)
        return LinearNDInterpolator(self.points, self.delta_values)
        # interpolator = KNeighborsRegressor()
        # interpolator.fit(self.points, self.delta_values)


    def samples(self, size):
        selection = self.simplices[np.random.choice(len(self.simplices), size, p=self.losses)]
        weights = -np.log(np.random.random(selection.shape)) # * self.boltzmann_values[selection]
        points = np.average(self.points[selection], axis=1, weights=np.tile(weights[:,:,np.newaxis],
(1,1,self.dims)))

        return points



if __name__ == '__main__':
    import itertools
```

```
def function(xyz, wait=False):
    if wait:
        from time import sleep
        sleep_time = len(xyz)/10.0
        print(f'Sleep_time: {sleep_time}')
        sleep(sleep_time)

    return np.linalg.norm(xyz, axis=-1)


for dim in range(2,7):
    for step in range(3,11):
        print(dim, step)

        points = np.array(list(itertools.product(np.linspace(*(0,1), step, endpoint=True), repeat=dim)))
        values = function(points)

        t = default_timer()
        sampler = BoltzmannAdaptiveSampler(function, points, values, kt=0.1)
        print(default_timer() - t)

        t = default_timer()
        sampler.delaunay.add_points(np.random.random((100,dim)))
        print(default_timer() - t)
```

Code S2. run_residue_sampling.py

```
import argparse

import os
import sys
import itertools
import tqdm
from timeit import default_timer

from multiprocessing import Pool

from nerf import align

from rdkit.Chem.rdmolfiles import SDMolSupplier
from rdkit.Chem.rdMolTransforms import SetDihedralDeg

from ase import Atoms
from ase.units import kcal, mol
from ase.optimize import BFGS, GPMin, FIRE
from ase.constraints import FixInternals

import torch
from aimnet import load_AIMNetSMD, load_AIMNetSMD_ens, AIMNetCalculator
from dftd4 import D4_model

if torch.cuda.is_available():
    model = load_AIMNetSMD_ens().cuda()
else:
    model = load_AIMNetSMD_ens()

calculator = D4_model(xc='wB97x', calc=AIMNetCalculator(model))

import numpy as np
import scipy as sp
import h5py

from sampler import BoltzmannAdaptiveSampler
from params import residue_params

parser = argparse.ArgumentParser()
parser.add_argument('--residues', type=str, nargs='+')
parser.add_argument('--rotamers', type=int, nargs='+')
parser.add_argument('--number_of_threads', type=int)

args = parser.parse_args()

RESIDUES = args.residues
```

```
ROTAMERS = args.rotamers
NUMBER_OF_THREADS = args.number_of_threads

name = '-'.join(f'{residue}_{rotamer}' for residue, rotamer in zip(RESIDUES, ROTAMERS))
molecules = [SDMolSupplier(f'_rotamers/{residue}.sdf', True, False, False)[rotamer] for residue, rotamer
in zip(RESIDUES, ROTAMERS)]

molecule_dofs = [residue_params[residue]['dofs'] for residue in RESIDUES]
dofs = [dof for dofs in molecule_dofs for dof in dofs]

dims = len(dofs)
polymer_length = len(RESIDUES)

limit_dict = {
    1 : 100,
    2 : 1000,
    3 : 10000,
    4 : 100000,
    5 : 100000,
    6 : 100000,
}

limit = limit_dict[dims]
print(limit)


def build(dihedrals):
    for (molecule, dof), dihedral in zip([[molecule, dof] for molecule, dofs in zip(molecules, molecule_dofs)
for dof in dofs], dihedrals):
        SetDihedralDeg(molecule.GetConformer(), *dof, float(dihedral))

    _positions = [molecule.GetConformer().GetPositions() for molecule in molecules]
    _numbers = [np.array(residue_params[residue]['atoms']) for residue in RESIDUES]

    upper_connect_indices = [residue_params[residue]['upper_connect_indices'] for residue in RESIDUES]
    lower_connect_indices = [residue_params[residue]['lower_connect_indices'] for residue in RESIDUES]

    for i in range(polymer_length-1):
        _positions[i+1] = align(_positions[i], _positions[i+1], mapping=(upper_connect_indices[i],
lower_connect_indices[i+1]))

    selections = [residue_params[residue]['residue_indices'] for residue in RESIDUES]
    selections[0] = residue_params[RESIDUES[0]]['lower_indices'] + selections[0]
    selections[-1] = selections[-1] + residue_params[RESIDUES[-1]]['upper_indices']

    positions = np.concatenate([position[selection] for position, selection in zip(_positions, selections)])
    numbers = np.concatenate([number[selection] for number, selection in zip(_numbers, selections)])

    positions = np.concatenate([positions[np.where(numbers != 1)], positions[np.where(numbers == 1)]])
```

```
    numbers = np.concatenate([numbers[np.where(numbers != 1)], numbers[np.where(numbers == 1)]])

    atoms = Atoms(numbers=numbers, positions=positions, calculator=calculator)

    atom_map = {}
    for i, position in enumerate(_positions):
        for j, point in enumerate(position):
            delta = np.linalg.norm(positions - point, axis=-1)
            atom_map[i,j] = np.argmin(delta) if np.min(delta) < 0.1 and _numbers[i][j] ==
numbers[np.argmin(delta)] else None


    bonds = {tuple(atom_map[i,j] for j in bond) for i, residue in enumerate(RESIDUES) for bond in
residue_params[residue]['bonds'] if all([atom_map[i,j] is not None for j in bond])}
    dihedrals = [[atom_map[i,j] for j in dof] for i, dofs in enumerate(molecule_dofs) for dof in dofs]

    atoms.set_constraint(FixInternals(
        bonds=[[atoms.get_distance(*bond), bond] for bond in bonds],
        dihedrals=[[np.radians(atoms.get_dihedral(*dihedral)), dihedral] for dihedral in dihedrals],
        epsilon=0.03
    ))

    return atoms


def score(dihedrals):
    atoms = build(dihedrals)

    # atoms.write(f'_temp/{name}_premin.pdb', append=True)

    try:
        #if np.max(np.linalg.norm(np.nan_to_num(atoms.get_forces()), axis=-1)) * (1/(kcal/mol)) < 1000.0:
        opt = BFGS(atoms, logfile='/dev/null')
        opt.run(steps=99, fmax=0.005)
    except:
        print(f'FAILED MINIMIZE: {dihedrals}')

    # atoms.write(f'_temp/{name}_postmin.pdb', append=True)

    results = {
        'point' : dihedrals,
        'value' : np.nan_to_num(atoms.get_total_energy())[0] * (1/(kcal/mol)),
        'xyz' : atoms.positions
    }

    return results


def scores(dihedrals_array):
```

```
    with Pool(NUMBER_OF_THREADS) as p:
        results = np.array(list(tqdm.tqdm(p.imap(score, dihedrals_array), total=len(dihedrals_array))))

    points = np.array([r['point'] for r in results])
    values = np.array([r['value'] for r in results])
    xyzs = np.array([r['xyz'] for r in results])

    if os.path.exists(f'_ensembles5/{name}.hdf5'):
        with h5py.File(f'_ensembles5/{name}.hdf5', 'r') as hdf5_file:
            points = np.concatenate([hdf5_file['points'][:], points])
            values = np.concatenate([hdf5_file['values'][:], values])
            xyzs = np.concatenate([hdf5_file['xyzs'][:], xyzs])

    with h5py.File(f'_ensembles5/{name}.hdf5', 'w') as hdf5_file:
        hdf5_file.create_dataset('points', data=points, dtype=np.dtype('float32'))
        hdf5_file.create_dataset('values', data=values, dtype=np.dtype('float32'))
        hdf5_file.create_dataset('xyzs', data=xyzs, dtype=np.dtype('float32'))

    return np.array([r['value'] for r in results])

if os.path.exists(f'_ensembles5/{name}.hdf5'):
    with h5py.File(f'_ensembles5/{name}.hdf5', 'r') as hdf5_file:
        points = hdf5_file['points'][:]
        values = hdf5_file['values'][:]
else:
    points = np.array(list(itertools.product(np.linspace(*(-180, 180), 7, endpoint=True), repeat=dims)))
    values = scores(points)

sampler = BoltzmannAdaptiveSampler(scores, points, values, kt=3.0, clip=12.0,
nthreads=NUMBER_OF_THREADS)

print(len(sampler.points))

while len(sampler.points) < limit:
    sampler.run()
```

Code S3. run_polymer_sampling.py

```
import argparse

import os
import sys
import itertools
import random
import tqdm

from multiprocessing import Pool

from align import align

from rdkit import Chem

from ase import Atoms
from ase.units import kcal, mol
from ase.optimize import BFGS
from ase.constraints import FixInternals

import torch
from aimnet import load_AIMNetSMD, load_AIMNetSMD_ens, AIMNetCalculator
from dftd4 import D4_model

if torch.cuda.is_available():
    model = load_AIMNetSMD_ens().cuda()
else:
    model = load_AIMNetSMD_ens()

calculator = D4_model(xc='wB97x', calc=AIMNetCalculator(model))

import numpy as np
import scipy as sp
import networkx as nx
import h5py

from params import residue_params, mirror_residue_params
full_residue_params = {**residue_params, **mirror_residue_params}

parser = argparse.ArgumentParser()
parser.add_argument('--residues', type=str, nargs='+')
parser.add_argument('--polymer_repeats', type=int)
parser.add_argument('--run_size', type=int)

args = parser.parse_args()

RESIDUES = args.residues
```

```
POLYMER_REPEATS = args.polymer_repeats
RUN_SIZE = args.run_size

POINTS = [h5py.File(f'_residues/{residue}.hdf5', 'r')['points'][:] for residue in RESIDUES]
VALUES = [h5py.File(f'_residues/{residue}.hdf5', 'r')['values'][:] for residue in RESIDUES]
XYZS = [h5py.File(f'_residues/{residue}.hdf5', 'r')['xyzs'][:] for residue in RESIDUES]

name = '-'.join(f'{residue}' for residue in RESIDUES)

polymer_length = len(RESIDUES) * POLYMER_REPEATS

smiles = ''.join(
    [full_residue_params[RESIDUES[0]]['lower_smiles']] +
    [full_residue_params[residue]['residue_smiles'] for residue in RESIDUES * POLYMER_REPEATS] +
    [full_residue_params[RESIDUES[-1]]['upper_smiles']]
)

molecule = Chem.AddHs(Chem.MolFromSmiles(smiles))
bonds = [(bond.GetBeginAtomIdx(), bond.GetEndAtomIdx()) for bond in molecule.GetBonds()]
path_length_matrix = np.clip([[len(Chem.rdmolops.GetShortestPath(molecule, i, j)) if i != j else 0 for j in
range(molecule.GetNumAtoms())] for i in range(molecule.GetNumAtoms())], None, 10)

def build(ijk):
    _positions = [XYZS[i][index] for i, index in enumerate(ijk)] * POLYMER_REPEATS
    _charges = [np.array(full_residue_params[residue]['charges']) for residue in RESIDUES] *
POLYMER_REPEATS
    _radii = [np.array(full_residue_params[residue]['radii']) for residue in RESIDUES] *
POLYMER_REPEATS
    _residues = [np.full_like(full_residue_params[residue]['atoms'], i) for i, residue in
enumerate(RESIDUES * POLYMER_REPEATS)]
    _numbers = [np.array(full_residue_params[residue]['atoms']) for residue in RESIDUES] *
POLYMER_REPEATS

    upper_connect_indices = [full_residue_params[residue]['upper_connect_indices'] for residue in
RESIDUES] * POLYMER_REPEATS
    lower_connect_indices = [full_residue_params[residue]['lower_connect_indices'] for residue in
RESIDUES] * POLYMER_REPEATS

    for i in range(polymer_length-1):
        _positions[i+1] = align(_positions[i], _positions[i+1], mapping=(upper_connect_indices[i],
lower_connect_indices[i+1]))

    selections = [full_residue_params[residue]['residue_indices'] for residue in RESIDUES] *
POLYMER_REPEATS
    selections[0] = full_residue_params[RESIDUES[0]]['lower_indices'] + selections[0]
    selections[-1] = selections[-1] + full_residue_params[RESIDUES[-1]]['upper_indices']

    positions = np.concatenate([position[selection] for position, selection in zip(_positions, selections)])
    charges = np.concatenate([charge[selection] for charge, selection in zip(_charges, selections)])
```

```
    radii = np.concatenate([radius[selection] for radius, selection in zip(_radii, selections)])
    residues = np.concatenate([residue[selection] for residue, selection in zip(_residues, selections)])
    numbers = np.concatenate([number[selection] for number, selection in zip(_numbers, selections)])

    positions = np.concatenate([positions[np.where(numbers != 1)], positions[np.where(numbers == 1)]])
    charges = np.concatenate([charges[np.where(numbers != 1)], charges[np.where(numbers == 1)]])
    radii = np.concatenate([radii[np.where(numbers != 1)], radii[np.where(numbers == 1)]])
    residues = np.concatenate([residues[np.where(numbers != 1)], residues[np.where(numbers == 1)]])
    numbers = np.concatenate([numbers[np.where(numbers != 1)], numbers[np.where(numbers == 1)]])

    # atom_map = {}
    # for i, position in enumerate(_positions):
    #     for j, point in enumerate(position):
    #         delta = np.linalg.norm(positions - point, axis=-1)
    #         atom_map[i,j] = np.argmin(delta) if np.min(delta) < 0.1 and _numbers[i][j] ==
numbers[np.argmin(delta)] else None

    # bonds = [tuple(atom_map[i,j] for j in bond) for i, residue in enumerate(RESIDUES *
POLYMER_REPEATS) for bond in full_residue_params[residue]['bonds'] if all([atom_map[i,j] is not None
for j in bond])]
    # dihedrals = [[atom_map[i,j] for j in dof] for i, dofs in enumerate([full_residue_params[residue]['dofs']
for residue in RESIDUES] * POLYMER_REPEATS) for dof in dofs]

    atoms = Atoms(numbers=numbers, positions=positions, calculator=calculator)
    atoms.radii = radii
    atoms.charges = charges
    atoms.residues = residues
    atoms.bonds = bonds

    # atoms.set_constraint(FixInternals(
    #     bonds=[[atoms.get_distance(*bond), bond] for bond in bonds],
    #     dihedrals=[[np.radians(atoms.get_dihedral(*dihedral)), dihedral] for dihedral in dihedrals],
    #     epsilon=0.03
    # ))

    return atoms



def score(ijk):
    atoms = build(ijk)

    radius_matrix = atoms.radii[:,np.newaxis] + atoms.radii
    charge_matrix = atoms.charges[:,np.newaxis] * atoms.charges
    distance_matrix = np.linalg.norm(atoms.positions[:,np.newaxis,:] - atoms.positions, axis=-1)

    selection = np.triu(np.where(path_length_matrix >= 4, True, False), 1)
```

```
    crude_energy = (radius_matrix / distance_matrix)**12 - 2 * (radius_matrix / distance_matrix)**6 + 5 *
(path_length_matrix**2 * charge_matrix / distance_matrix**4)
    crude_energy = np.sum(crude_energy[selection]) / len(atoms)

    if True: # crude_energy < 0.0:
        # atoms.write(f'_test/{name}.pdb', append=True)

        # try:
        #     opt = BFGS(atoms)#, logfile='/dev/null')
        #     opt.run(steps=99, fmax=0.005)
        # except:
        #     print(f'FAILED MINIMIZE: {ijk}')

        # atoms.write(f'_test/{name}_postmin.pdb', append=True)

        energy = np.nan_to_num(atoms.get_total_energy())[0] * (1/(kcal/mol))

        return {
            'point' : ijk,
            'value' : energy,
            'crude_value' : crude_energy,
            'xyz' : atoms.positions
        }
    else:
        return None



def scores(ijks):
    results = [score(ijk) for ijk in tqdm.tqdm(ijks)]

    points = np.array([r['point'] for r in results if r is not None])
    values = np.array([r['value'] for r in results if r is not None])
    crude_values = np.array([r['crude_value'] for r in results if r is not None])
    xyzs = np.array([r['xyz'] for r in results if r is not None])

    if os.path.exists(f'_polymers2/{name}.hdf5'):
        with h5py.File(f'_polymers2/{name}.hdf5', 'r') as hdf5_file:
            points = np.concatenate([hdf5_file['points'][:], points])
            values = np.concatenate([hdf5_file['values'][:], values])
            crude_values = np.concatenate([hdf5_file['crude_values'][:], crude_values])
            xyzs = np.concatenate([hdf5_file['xyzs'][:], xyzs])

    with h5py.File(f'_polymers2/{name}.hdf5', 'w') as hdf5_file:
        hdf5_file.create_dataset('points', data=points, dtype=np.dtype('int32'))
        hdf5_file.create_dataset('values', data=values, dtype=np.dtype('float32'))
        hdf5_file.create_dataset('crude_values', data=crude_values, dtype=np.dtype('float32'))
        hdf5_file.create_dataset('xyzs', data=xyzs, dtype=np.dtype('float32'))
```

```
    return results

probabilities = [np.exp((values - np.min(values)) / -1.2) for values in VALUES]

for _ in range(100):
    ijks = np.transpose(np.array([np.random.choice(len(probability), size=RUN_SIZE, replace=True,
p=probability/np.sum(probability)) for probability in probabilities]))

    scores(ijks)


def distance_matrix(points1, points2):
    return np.sqrt(np.mean(np.power((points1 - points2 - 180.0) % 360.0 - 180.0, 2), axis=-1)) / 180.0

for _ in range(100):
    with h5py.File(f'_polymers2/{name}.hdf5', 'r') as hdf5_file:
        polymer_probabilities = np.exp( ( hdf5_file['values'][:] - np.min(hdf5_file['values'][:]) ) / (-
0.6*polymer_length) )
        sele = np.random.choice(len(polymer_probabilities),
p=polymer_probabilities/np.sum(polymer_probabilities))
        print(sele)
        low_point = hdf5_file['points'][:][sele]

    distance_matrix0 = distance_matrix(POINTS[0], POINTS[0][low_point[0]])
    distance_matrix1 = distance_matrix(POINTS[1], POINTS[1][low_point[1]])

    closest_points0 = np.argpartition(distance_matrix0, 10)[:10]
    closest_points1 = np.argpartition(distance_matrix1, 10)[:10]

    scores(np.array(list(itertools.product(closest_points0, closest_points1))))
```

Supplementary References

1 Di Blasio, B. et al. Structural characterization of the beta-bend ribbon spiral: crystallographic analysis of two long (L-Pro-Aib)n sequential peptides. J Am Chem Soc 114, 6273-6278 (1992). https://doi.org:10.1021/ja00042a001

2 Choi, S. H., Guzei, I. A., Spencer, L. C. & Gellman, S. H. Crystallographic characterization of helical secondary structures in alpha/beta-peptides with 1:1 residue alternation. J Am Chem Soc 130, 6544-6550 (2008). https://doi.org:10.1021/ja800355p

3 Basuroy, K., Dinesh, B., Shamala, N. & Balaram, P. Structural characterization of backbone-expanded helices in hybrid peptides: (alphagamma)n and (alphabeta)n sequences with unconstrained beta and gamma homologues of L-Val. Angew Chem Int Ed Engl 51, 8736-8739 (2012). https://doi.org:10.1002/anie.201204436

4 Yoon, H., Lee, J., Kang, P. & Choi, S. H. Promotion of 11/9-helical folding in α/β-peptides containing β2-homoalanine residue. Peptide Science 114, e24244 (2022). https://doi.org:https://doi.org/10.1002/pep2.24244

5 Delsuc, N., Godde, F., Kauffmann, B., Léger, J.-M. & Huc, I. The Herringbone Helix: A Noncanonical Folding in Aromatic−Aliphatic Peptides. J Am Chem Soc 129, 11348-11349 (2007). https://doi.org:10.1021/ja074285s

6 Prabhakaran, P. et al. Sequence-specific unusual (1-->2)-type helical turns in alpha/beta-hybrid peptides. J Am Chem Soc 130, 17743-17754 (2008). https://doi.org:10.1021/ja804297f

7 Karle, I. L. & Balaram, P. Structural characteristics of alpha-helical peptide molecules containing Aib residues. Biochemistry 29, 6747-6756 (1990). https://doi.org:10.1021/bi00481a001

8 Topol, I. A. et al. alpha- and 3(10)-helix interconversion: a quantum-chemical study on polyalanine systems in the gas phase and in aqueous solvent. J Am Chem Soc 123, 6054-6060 (2001). https://doi.org:10.1021/ja0038934

9 Andersson, A., Yatsyna, V., Linares, M., Rijs, A. & Zhaunerchyk, V. Indication of 3(10)-helix structure in gas-phase neutral pentaalanine. J Phys Chem A 127, 938-945 (2023). https://doi.org:10.1021/acs.jpca.2c07863

10 Rossi, M., Scheffler, M. & Blum, V. Impact of vibrational entropy on the stability of unsolvated peptide helices with increasing length. J Phys Chem B 117, 5574-5584 (2013). https://doi.org:10.1021/jp402087e

11 Bender, B. J. et al. A practical guide to large-scale docking. Nat Protoc 16, 4799-4832 (2021). https://doi.org:10.1038/s41596-021-00597-z

12 Baldauf, C., Gunther, R. & Hofmann, H. J. Theoretical prediction of the basic helix types in alpha,beta-hybrid peptides. Biopolymers 84, 408-413 (2006). https://doi.org:10.1002/bip.20493

13 Legrand, B. et al. Unprecedented chain-length-dependent conformational conversion between 11/9 and 18/16 helix in alpha/beta-hybrid peptides. Angew Chem Int Ed Engl 53, 13131-13135 (2014). https://doi.org:10.1002/anie.201407329

14 Yokum, T. S., Gauthier, T. J., Hammer, R. P. & McLaughlin, M. L. Solvent Effects on the 310-/α-Helix Equilibrium in Short Amphipathic Peptides Rich in α,α-Disubstituted Amino Acids. Journal of the American Chemical Society 119, 1167-1168 (1997). https://doi.org:10.1021/ja962875c

15 Sharma, G. V. et al. Hybrid helices: motifs for secondary structure scaffolds in foldamers. J Am Chem Soc 131, 17335-17344 (2009). https://doi.org:10.1021/ja907074u

16 Serrano-Andrés, L. & Fülscher, M. P. Theoretical Study of the Electronic Spectroscopy of Peptides. 2. Glycine and N-Acetylglycine. J Am Chem Soc 118, 12200-12206 (1996). https://doi.org:10.1021/ja9619972

17 Bulheller, B. M., Rodger, A. & Hirst, J. D. Circular and linear dichroism of proteins. Phys Chem Chem Phys 9, 2020-2035 (2007). https://doi.org:10.1039/b615870f

18 Ohmae, E., Tanaka, S., Miyashita, Y., Katayanagi, K. & Matsuo, K. Vacuum-Ultraviolet Circular Dichroism Spectra of Escherichia coli Dihydrofolate Reductase and Its Mutants: Contributions of Phenylalanine and Tyrosine Side Chains and Exciton Coupling of Two Tryptophan Side Chains. J Phys Chem B 119, 13002-13008 (2015). https://doi.org:10.1021/acs.jpcb.5b07480

19 Ianeselli, A. et al. Atomic Detail of Protein Folding Revealed by an Ab Initio Reappraisal of Circular Dichroism. J Am Chem Soc 140, 3674-3682 (2018). https://doi.org:10.1021/jacs.7b12399

20 Tejero, R., Snyder, D., Mao, B., Aramini, J. M. & Montelione, G. T. PDBStat: a universal restraint converter and restraint analysis software package for protein NMR. J Biomol NMR 56, 337-351 (2013). https://doi.org:10.1007/s10858-013-9753-7

21 Lee, W., Tonelli, M. & Markley, J. L. NMRFAM-SPARKY: enhanced software for biomolecular NMR spectroscopy. Bioinformatics 31, 1325-1327 (2015). https://doi.org:10.1093/bioinformatics/btu830

22 Ho, B. K. & Brasseur, R. The Ramachandran plots of glycine and pre-proline. BMC Struct Biol 5, 14 (2005). https://doi.org:10.1186/1472-6807-5-14

23 Vogeli, B., Ying, J., Grishaev, A. & Bax, A. Limits on variations in protein backbone dynamics from precise measurements of scalar couplings. J Am Chem Soc 129, 9377-9385 (2007). https://doi.org:10.1021/ja070324o

24 Trainor, K., Palumbo, J. A., MacKenzie, D. W. S. & Meiering, E. M. Temperature dependence of NMR chemical shifts: Tracking and statistical analysis. Protein Sci 29, 306-314 (2020). https://doi.org:10.1002/pro.3785
